# A Deep-Learning Neural Network Potential Accelerated First-Principles Study on the Structural Changes Modulated by Methylation and Solvation in 27 Protonated Tripeptides

**DOI:** 10.1021/acs.jpca.5c06004

**Published:** 2025-11-18

**Authors:** Dong Cao Hieu, Po-Jen Hsu, Jer-Lai Kuo

**Affiliations:** † Institute of Atomic and Molecular Sciences, 71556 Academia Sinica, Taipei 10617, Taiwan; ‡ Molecular Science and Technology Program, Taiwan International Graduate Program, Academia Sinica, Taipei 11529, Taiwan; § International Graduate Program of Molecular Science and Technology (NTU-MST), National Taiwan University, Taipei 10617, Taiwan; ∥ Department of Chemistry, National Tsing Hua University, Hsinchu 30013, Taiwan

## Abstract

Exploring low-energy conformers of tripeptides with different side chains using first-principles methods is important not only to interpret, validate, and predict experimental infrared (IR) spectra in the gas phase but also to understand how the relative stability of different conformations can be modulated by the interplay of basic molecular interactions. In this work, we identified low-energy conformers of 27 protonated tripeptides at M06–2X/6–311+G­(d,p) by employing a deep-learning-based neural network potential (DL-NNP) to speed up the structure search. Our methodology also demonstrates a seamless transition from gas phase to implicit-solvent models using the polarizable continuum model (PCM), achieving a mean absolute error (MAE) of energies less than 1.1 and 2.1 kJ/mol, respectively. We found that the number of distinct minima below 25 kJ/mol for a given tripeptide ranges from 10 to 59 in the gas phase and 60–361 in PCM-water. Analysis of the structures of these low-energy minima reveals how methylation modulates molecular interactions through both electronic and steric effects. Finally, the low-energy conformers of methylated tripeptides identified in this study provide valuable insights to compare with available experimental data and to stimulate future experimental and theoretical investigations.

## Graphical abstract

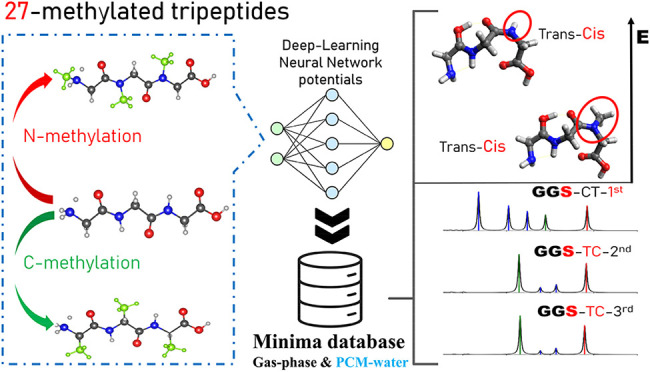

## Introduction

The biological activity of peptides is intrinsically linked to their three-dimensional (3D) structures.^1,2^ The relative stability of different conformations of any given peptide is known to be influenced by the interplay of several factors such as intramolecular hydrogen-bond (H-bond) interactions,^3–7^ protonation sides,^8,9^ and steric effects.^10,11^ From a physical science point of view, all of these factors can be fine-tuned by changing the side chains^12–15^ and the conditions of the solvation.^16–18^ Methylation of a peptide is one of the most minor modifications, but through the combination of state-of-the-art gas phase spectroscopy and first-principles calculations,^15,19^ physical chemists were able to investigate the effects of methylation on the structures of several dipeptides^20,21^ and tripeptides^22^ by comparing experimental vibrational spectra and structures optimized by various first-principles methods.^6,23–28^

Over the past decade, advancements in experimental techniques and the improved accuracy and efficiency of first-principles methods have provided powerful tools for examining the structures and relative stabilities of different peptide conformations. For example, Johnson’s group employed IRPD (infrared predissociation) and IR–IR double resonance spectroscopy to investigate the effect of *N*-methylation by examining four types of protonated dipeptides composed of glycine (G) and sarcosine (S).^20,21^ Using MP2/6–311+G­(d,p) calculations, they found that *cis* and *trans* conformers could coexist under their experimental conditions. In 2017, Luo’s group studied protonated triglycine (GGG) using IRMPD, X-ray spectroscopies, and density functional theory (DFT) calculations at the B3LYP/6–311++G­(d,p) level. They identified that a nonproline *cis*-peptide bond conformation is the global minimum of protonated triglycine.^26^ In 2022, Garand’s group examined eight types of tripeptides consisting of glycine (G) and alanine (A) to study the effect of Cα-methylation by comparing IR-IR double resonance spectroscopy with vibrational spectra of low-energy conformers within the 0–25 kJ/mol range calculated by CAM-B3LYP/def2-TZVP/GD3BJ level. They concluded that Cα-methylation increases the local proton affinities of functional groups, thereby tuning the preference for different protonation sites in these tripeptides.^22^

Various studies on the effects of solvation on peptide structural changes have been conducted using either explicit or implicit water models. Boyarkin’s group investigated the influence of microhydration on protonated glycine (G) and triglycine (GGG) with explicit water using IRPD spectroscopy. They suggested that the intrinsic structures of these biomolecules could become kinetically trapped under the cryogenic conditions in their experimental setup and may induce the formation of additional conformers than the global minima. Furthermore, they concluded that upon the addition of 4 to 5 water molecules, the embedded amino acid and peptide could adopt its native-like structure.^29^ Building on this concept, Garand’s group further investigated protonated GGG and AAA with 1 or 2 water molecules. They demonstrated that the addition of water solvent molecules can change the stability preference toward O-protonated conformers over N-terminal protonated conformers in both types of tripeptides. Additionally, the Cα-methylation effect observed in gas-phase tripeptides,^22^ which alters proton affinities of functional groups and thereby modulates intramolecular hydrogen bond strengths, remains significant in microsolvated tripeptides.^30^ In addition to explicit inclusion of water molecules, another commonly used approach for modeling peptides under full-solvation conditions is to employ an implicit solvent method, such as the Polarizable Continuum Model (PCM),^31–35^ developed by Tomasi et al.^36^ using first-principles methods. For instance, a theoretical study on four types of protonated tripeptides at the M06–2X/6–311++G­(d,p) (denoted as M06–2X) level using implicit PCM or explicit solvent (5 water molecules) showed that the secondary structure, protonation sites, intramolecular H-bonds, and relative energy ranking are preserved in both solvation approaches. However, differences in the energy gaps between conformers in the two conditions result in quantitatively different Boltzmann-weighted populations.^37^

Motivated by these studies, we present a systematic investigation of 27 tripeptides to examine how N- and Cα-methylation (Figure 1a) modulate their structural characteristics in both the gas phase and PCM using first-principles methods. Based on the above studies, extensive structural searches for each methylated tripeptide are essential using DFT methods. However, the conventional structural search process involving several first-principles methods not only becomes computationally too expensive but also comes with a high risk of overlooking crucial low-energy structures due to multiple layers of reoptimizations. To overcome these challenges, we utilize deep-learning neural network potentials (DL-NNPs), which have been shown to accelerate the structural searching of peptides and sugars^38–42^ at first-principles accuracy. Specifically, we designed an iterative training scheme to develop a set of robust DL-NNP models that are capable of reproducing the energy landscape of M06–2X for all 27 tripeptides (Figure 1b).

**Figure 1:**
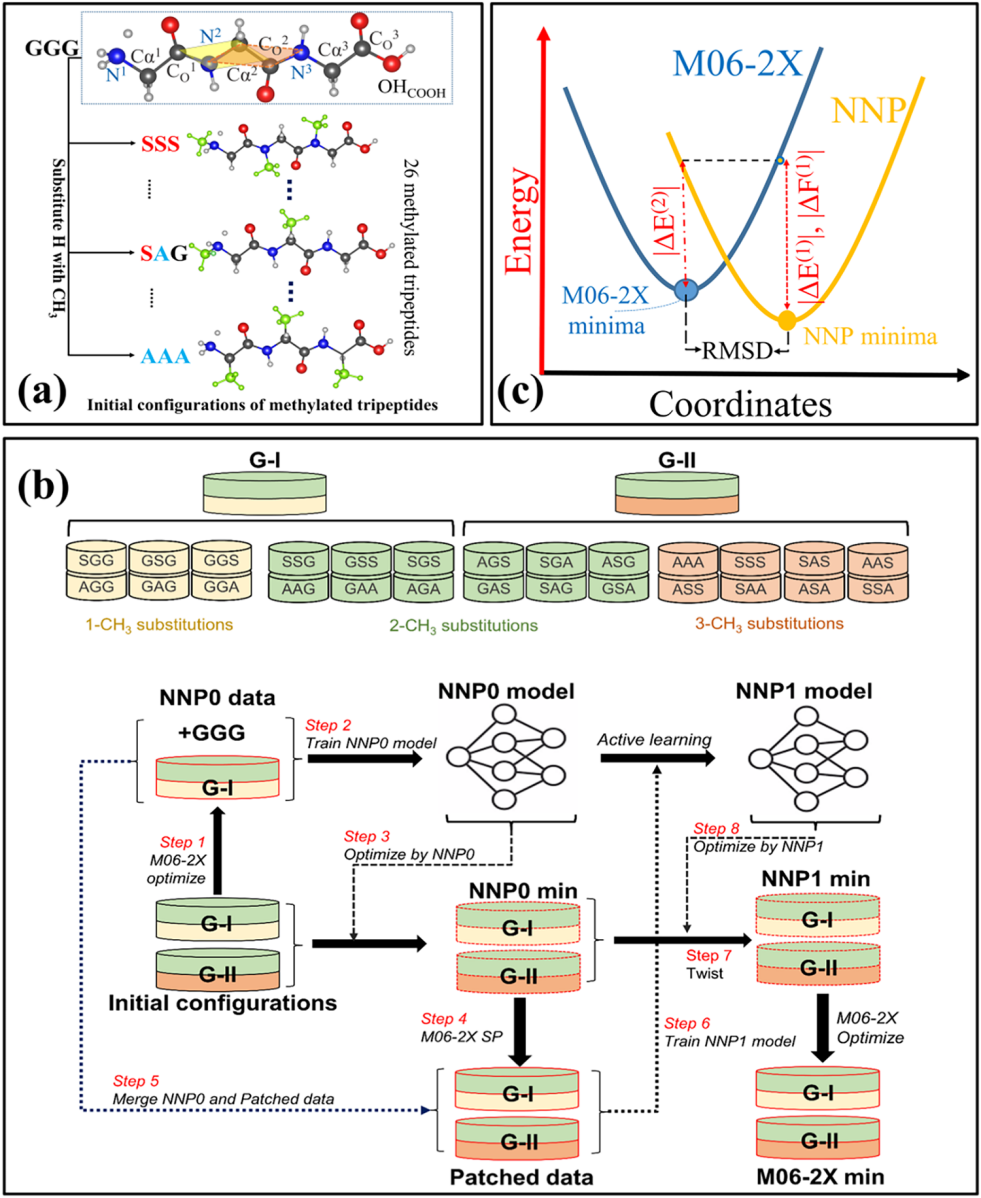
Illustration of the procedure to generate structures of 26 methylated tripeptides by replacing an H atom at either Cα or N-position with a methyl group on distinct conformers of protonated triglycine is shown in (a). Classification of 26 methylated tripeptides into Group I (GI, binary peptides with ≤2 methyl substitutions) and Group II (G-II) are shown in (b). Active learning workflow for training NNP0 and NNP1 models with extensive conformational search. Low-energy conformers (<50 kJ/mol) from NNP1 were reoptimized using the M06–2X/6- 311+G­(d,p) method. (c) Definition of the accuracy metrics: Δ*E* ^(1)^, Δ*F* ^(1)^, Δ*E* ^(2)^, and RMSD between NNP1 and M06–2X/6–311+G­(d,p) minimum energy structures. Results are summarized in Tables 1, 2, S2, and S3.

While the structural difference between the gas phase and water using PCM (denoted as PCM-water) is well documented, it is not straightforward to transfer knowledge from the gas phase to PCM-water. The accuracy of the NNP optimization will be evaluated by predicting the NNP model on an independent test set and comparing the NNP and M06–2X minima (Figure S1 of the ESI). To assess the effects of methylation and water solvent on the tripeptides, we will use the zero-point corrected relative energy (abbreviated as ZPE) diagram derived from stable conformers at the M06–2X level. Additionally, we simulate the IR spectra differences of all 27 tripeptides. Finally, by providing a comprehensive data set of methylated tripeptides with first-principles accuracy, we hope to facilitate experimental studies that could confirm our results.

## Methodology

### Generating Initial Configurations of Methylated Tripeptides from the Database of Triglycine

We used the structural database of protonated triglycine reported in our previous work,^42^ which contains 945 distinct minima evaluated at the M06–2X level. The initial configurations of the methylated tripeptides were generated by replacing an H atom at either Cα or N-position with a methyl group (Figure 1a). While preserving the diverse backbone structures from the GGG minima, this simple replacement scheme achieves an average success rate of approximately 92% (see Table S1 of the ESI). It ensures a diverse set of initial configurations for methylated tripeptides. The same M06–2X level of theory is chosen for the methylated systems based on the benchmark tests against DLPNO–CCSD­(T)/aug-cc-pvTZ on neutral and protonated poly glycine.^42^

### The Parameters for Training NNP Models Based on the SchNET Architecture

The NNP models were created using the SchNet neural network architecture,^42^ with settings identical to those in previous works on various peptides and sugars.^38–42^ Specifically, interatomic distances are expanded using 25 Gaussian radial basis functions with a cutoff distance of 15 Å. The neural network employs 4 message-passing interaction layers, where each atom is represented by a 128-dimensional feature vector that encodes its local chemical environment based on element type and geometric arrangement of neighboring atoms. To train the NNP models for energy and force predictions, we utilize a loss function^43^ with a trade-off parameter, ρ = 0.01, which balances the contributions of energy and force errors. During training, we use the Adam optimizer and the Warm-Restart learning rate scheduler with a learning rate of 0.001 and 1400 training epochs.^44^

### Generation of Test and Training Sets for NNP0 and NNP1 (Gas Phase)

The training procedure for NNP is summarized in Figure 1b. For the training set, we limited it to only 12 types of binary methylated tripeptides (the G-I database) as the computational cost for G-I tripeptides (adding 1–2 methyl groups) is lower than for G-II tripeptides (adding 2–3 methyl groups). In the following, we demonstrate that we can rely on a training set of tripeptides extracted from the G-I model to predict tripeptides with reasonable accuracy in both G-I and G-II.

To ensure that all possible *Cis*/*Trans* peptide bond and protonation sites configurations are included in both training and test sets, we partition all initial structures of a tripeptide into 12 groups (4 *cis*/*trans* configurations times 3 protonation states) as shown in Table S1 of the ESI. For each type of methylated tripeptide, we sequentially select 200 structures from these 12 groups. Next, these selected initial structures are optimized at the M06–2X level using the Gaussian 16 package.^45^ We then collect all snapshots from the optimization trajectories^38–42^ and remove duplicate snapshots using our structure screening algorithm.^46^ As a result, we obtain 67,030 distinct snapshots for all tripeptides, which will serve as the test set to evaluate the performance of the NNP models in the gas phase.

Repeating the same procedure in the previous paragraph for 12 tripeptides in G-I, we obtained 37,858 distinct structures. Our training set for the NNP0 model contains these 37,858 data points and 6000 data points extracted from our previous study on GGG.^42^ The generating G-I training set and training of NNP0 were shown as Step 1 and Step 2 in Figure 1b. The accuracy of the NNP models can be progressively improved through a multistep patching process (shown in Steps 3 to 6 in Figure 1b). We incorporated the NNP0 model to perform geometry optimization for all initial configurations of methylated tripeptides (Step 3). The mean absolute errors in energy and force (E-MAE and F-MAE) for NNP0 minima were evaluated using single-point M06–2X calculations (Step 4). Those NNP0 minima with errors exceeding 1.5 kJ/mol for energy or 1.5 kJ/mol·Å for forces were added to the above-mentioned NNP0 training data to train the NNP1 model (the active learning protocol from Steps 5 to 6). The number of data points added to the NNP0 training data set is shown in the ninth line of Table S1.

### Generation of Test and Training Sets for NNP0w and NNP1w (PCM-Water)

The gas phase NNP1 model can be transferred to generate NNP models to describe the structures and forces under solvation (denoted as NNPw) via PCM-water calculations. We selected all NNP1 gas phase minima within the 0–50 kJ/mol energy range and reoptimized them using the PCM-water option in Gaussian 16. This test set comprises 17,965 snapshots (PCM-test set) collected from these optimizations. The training procedure for NNP models in PCM is similar and is summarized in Figure S1 of the ESI.

The NNP0w training data was generated by recalculating the energies and forces of the NNP0 training data using the PCM-water option (Step 1w in Figure S1 of the ESI). The training parameters and patching scheme for NNP0w and NNP1w are identical to those used for the gas phase NNP models. However, two key modifications were made during training of the NNP0w model and during patching of the NNP1w model. First, the NNP0w model was updated from the NNP1 model using a transfer learning scheme (Step 2w in Figure S1 of the ESI), allowing fine-tuning of the gas phase model with solvated data while leveraging prior knowledge. Second, to obtain a more diverse set of NNP0w minima for the patching step, we performed NNP0w optimizations on the initial configurations and the M06–2X/6–311+G­(d,p) data in the gas phase (Step 3w).

### Searching Low-Energy Minima of 26 Methylated Tripeptides Using NNP and M06–2X

Our primary application of the NNP1 and NNP1w models is efficient, high-accuracy conformational searches for all methylated tripeptides. Compared to DFT geometry optimizations at the M06–2X level, our NNP models are approximately 400 times faster while maintaining comparable accuracy (1–2 kJ/mol error). The advantage makes NNPs superior to both computationally expensive DFT methods and less accurate DFTB approaches for extensive conformational sampling. The conformation search algorithm is to randomly twist the following single bonds on the NNP0 or NNP0w minima in the angle range of −0.3π to 0.3π: N^1^–Cα^1^, Cα^1^– C_O_^1^, N^2^–Cα^2^, Cα^2^– C_O_^2^, N^3^–Cα^3^, Cα^3^–C_O_^3^, and C_O_^3^–OH_COOH_ (Figure 1b). Subsequently, we obtained 30,000 structural candidates for the geometry optimization using the NNP1 or NNP1w models (Step 7). We repeated the single-bond twisting for the newly identified NNP1 or NNP1w minima (Table S2 in SI) that are within 0–50 kJ/mol energy range until no new minima in this energy range were found. Then, all distinct minima with a relative energy less than 50 kJ/mol were further reoptimized at the M06–2X level. These searching and reoptimization processes were applied to all methylated tripeptides in both the gas phase and in PCM-water.

## Results and Discussions

### Evaluation of the Prediction Accuracy of NNP Models

#### NNP0 and NNP1 Models for Methylated Tripeptides in the Gas Phase

From Figure 2, we can see that the NNP0 model achieved an average E-MAE of 1.54 kJ/mol on the G-I test set. Remarkably, despite containing no G-II peptides in its training data, the NNP0 model demonstrated reasonable transferability to the G-II test set with an E-MAE of 2.07 kJ/mol (Figure 2b). Based on the NNP0 prediction on the gas phase test set, we do not need to include all 27 types of tripeptides in the training set, as long as similar atomic environments are included, extracted from the binary peptides in G-I. Our evaluation highlights the extrapolation capability of NNP models, which can be applied to methylated tetra-peptides or other peptides with similar functional groups. After patching the NNP0 model, the resulting NNP1 model has even lower E-MAEs on both the G-I (1.00 kJ/mol) and G-II (1.14 kJ/mol) test sets (Figure 2c,2d). The accuracy of NNP1 is comparable to that of our model for gas-phase triglycine, as reported in our previous study.^42^ Therefore, NNP1 is sufficiently precise to perform structural searches for 27 tripeptides. The structural search procedure using NNP1 consisted of two components: (1) systematic optimization of all initial configurations presented in Figure 1, and (2) optimization of twisted-structural candidates generated through the approach described in the searching low-energy minima section.

**Figure 2:**
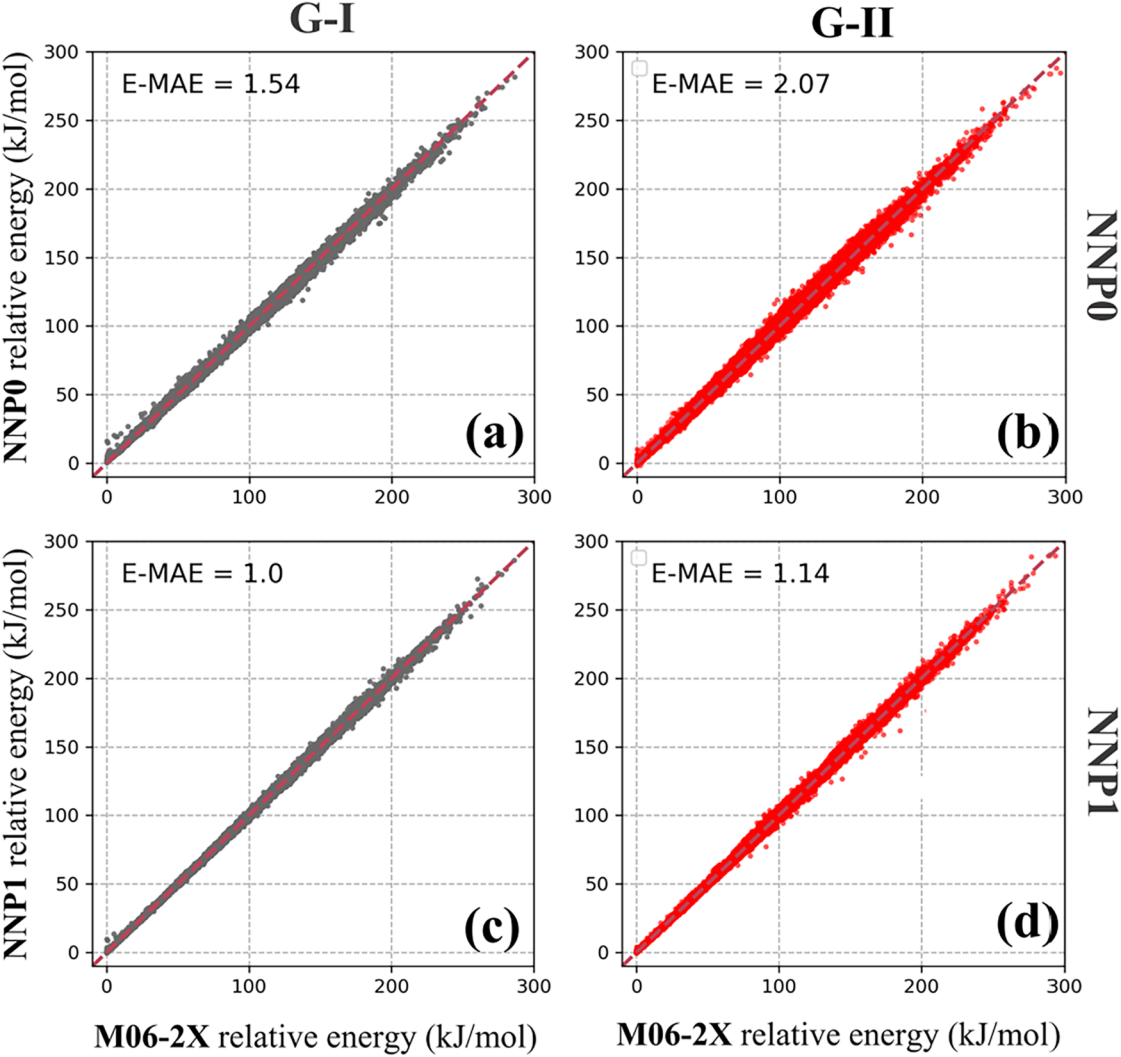
Correlation in the relative energies between M06–2X/6–311+G­(d,p) (*x*-axis) and NNP0 model­(*y*-axis) on the test sets of Group I (a) and Group II (b). The correlation between M06–2X/6- 311+G­(d,p) (*x*-axis) and NNP1 model (*y*-axis) on the test set of Group I (c) and Group II (d). The NNP0 model (without training G-II data) was able to predict precisely on the test set of G-II. The accuracy of NNP1 is improved by patching more data to the training set, as shown in Figure 1.

Given that the NNP1 model can approximate the potential energy surface at the M06–2X level of theory, it is essential to evaluate the accuracy of the local minima identified by NNP1 relative to those obtained from M06–2X calculations. As shown in Figure 1c, we analyze the success rate of reoptimization with M06–2X from the NNP1 minima, the energy differences (Δ*E* ^(1)^, Δ*E* ^(2)^), and gradient differences (Δ*F* ^(1)^). We also compute the root-mean-square deviation of the atomic positions between the superimposed NNP1 and M06–2X minima to quantify their structural differences (denoted as RMSD in Figure 1c). First, we found that the success rate of NNP1 optimizations on all methylated tripeptides is very high, with an average of 89% (third column of Table 1), which is comparable to the efficiency observed for poly glycine in our earlier study.^42^ This success rate was calculated as the ratio of the number of distinct minima evaluated at NNP1 to the number of distinct minima after reoptimization at M06–2X. The values of Δ*E* ^(1)^, Δ*F* ^(1)^, Δ*E* ^(2)^, and RMSD for methylated tripeptides are presented in the fourth, fifth, sixth, and seventh columns of Table 1, respectively. The average Δ*E* ^(1)^, Δ*F* ^(1)^, and Δ*E* ^(2)^ values for all tripeptides are generally small, namely 0.48 kJ/mol, 0.53 kJ/mol·Å, and 0.40 kJ/mol, respectively, indicating that the energy and forces of the NNP1 minima closely align with those of the M06–2X minima. Furthermore, the average RMSD between the NNP1 and M06–2X minima is only 0.06 Å. The small RMSDs are primarily due to subtle changes in hydrogen-atom orientation. The backbone structures remained unchanged during the M06–2X optimization. Our findings suggest that the NNP1 model is applicable for searching and optimizing methylated tripeptides with an accuracy comparable to the M06–2X level.

**Table 1:** Total Number of Distinct Low-Energy (Extensive Search Using the NNP1 Model (the Second Column) and Re-Optimization at M06-2X/6-311+G­(d,p) (the Third Column) for Tripeptides in G-I)^a^

name	NNP-1 min	M06–2X min	Δ*E* ^(1)^	Δ*F* ^(1)^	Δ*E* ^(2)^	RMSD
AGG	23	22(96%)	0.39	0.45	0.49	0.08
GAG	46	40(87%)	0.51	0.47	0.55	0.10
GGA	44	37(84%)	0.41	0.48	0.43	0.07
AAG	26	24(92%)	0.32	0.51	0.43	0.06
GAA	47	42(89%)	0.46	0.55	0.45	0.05
AGA	40	36(90%)	0.41	0.50	0.49	0.08
SGG	19	17(89%)	0.43	0.50	0.47	0.05
GSG	19	15(79%)	0.33	0.46	0.57	0.06
GGS	28	23(82%)	0.43	0.53	0.46	0.09
SSG	17	16(94%)	0.49	0.53	0.25	0.05
GSS	14	13(93%)	0.56	0.47	0.26	0.05
SGS	26	23(88%)	0.64	0.56	0.41	0.06

a The analysis of the structural similarity­(RMSD in Å) and the energy and gradient differences (Δ*E* ^1^, and Δ*F* ^1^ and Δ*E* ^2^) for the local minimadefined in Figure 1c.

#### NNP0w and NNP1w Models for Methylated Tripeptides in PCM-Water Solvent

The NNP1 model is specifically trained on gas-phase tripeptide configurations. As a result, its predictions exhibit significant deviations in both energy and forces when applied to the water-tripeptide test set, indicating limited transferability. These discrepancies arise from differences in the potential energy landscapes between gas-phase and solvated tripeptide systems, even when evaluated at identical geometries. From Figure S2a,b in the ESI, we can see that the E-MAEs are 239.0 and 230 kJ/mol for the test sets in G-I and G-II, respectively.

Nevertheless, we can quickly create the first robust model (NNP0w) to describe the structures of all 27 tripeptides in PCM-water. In particular, the NNP0w exhibits an E-MAE of 2.4 kJ/mol for both G-I and G-II (Figure S2c,d in the ESI). This result underscores the practical ability of NNP models to transfer knowledge between gas-phase and water-solvent structural data. This ability is beneficial, as it can be applied to various solvents, reducing the computational cost of generating training data. Through patching in the NNP0w model, the resulting NNP1w model further reduces the E-MAE on the test set to around 2.0 kJ/mol, as shown inFigure S2­(e),(f) in the ESI. Furthermore, the average Δ*E* ^(1)^, Δ*F* ^(1)^, Δ*E* ^(2)^, and RMSD values for all tripeptides in PCM-water demonstrate good accuracy, with minor mean deviations of 1.60 kJ/mol, 1.17 kJ/mol·Å, 0.89 kJ/mol, and 0.12 Å, respectively. Comprehensive statistical data for the 27 tripeptides are provided in Table S3 of the ESI.

### Comparison of the Low-Energy Minima of All 27 Tripeptides at M06–2X in the Gas Phase and the PCM-Water

We compile the number of distinct minima for all 27 types of tripeptides in the gas phase and in PCM-water, with zero-point-corrected relative energies under 25 kJ/mol at the M06–2X level, and present the results in Figure S3 of the ESI. The number of distinct low-energy conformers in water solvent is consistently higher than that in their gas phase counterparts. However, the number of distinct minima in the 0–25 kJ/mol window can range from 10 to 361, highlighting the potential risk of overlooking critical low-energy conformers (Figure S2 in ESI).

To analyze the stability of these conformers, we partition all distinct minima at the M06–2X level into four groups based on their peptide bond configurations: *trans*–*trans* (TT), *cis*–*trans* (CT), *trans*–*cis* (TC), and *cis*–*cis* (CC), in vacuum (Figure S3) and water solvent (Figure S4 of the ESI). Conformers with N-terminal, O1-amide, and O2-amide sites are shown in blue, red, and orange. In the gas phase, conformers containing both N-terminal and O1-amide species are observed in the low-energy energy range (Figure 4), which is consistent with experimental findings from Garand’s group^30^ on 8 types of tripeptides composed of alanine (A) and glycine (G), and the same notion is also applicable to all 27 tripeptides composed of A, G, and sarcosine (S). In most cases, the global minimum is the CT conformer with the N-terminal protonation site. Nonetheless, conformers with O1-amide protonation side and the TT backbone can be energetically competitive (i.e., the energy difference is within 5 kJ/mol).

In the implicit water solution, conformers with O-amide protonation sites are absent within the 0–25 kJ/mol energy window for all 27 tripeptides examined (Figure S4 of the ESI). This observation suggests that O-amide protonation is energetically less favorable than the N-terminal protonation in PCM-water. The shift in the preference for the protonation site with solvation is consistent with experimental findings from Garand’s group on the microsolvation of AAA and GGG with up to 2 water molecules.^22,30,47,48^ The reduced stability of O-protonated conformers can be attributed to the higher electronegativity of oxygen relative to nitrogen, which limits the delocalization of the positive charge upon protonation. Furthermore, the carbonyl oxygen serves as an H-bond acceptor rather than a protonated donor in the PCM-water model, which further destabilizes the O-protonated species. Surprisingly, TC conformers, typically considered less common in the gas phase, can emerge as the global minima for GGS, AGS, GAS, SGS, SAG, and SSS in PCM-water. However, we found that TC conformers are more stable water-PCM solvent than in the gas phase, as highlighted by the green arrows in Figures S5, S6, and S7 of the ESI.

The number of distinct stable conformers within 0–25 kJ/mol increases approximately 6-fold in aqueous solution (60–361) compared to the gas phase (10–59) in Figure S3 of the ESI. This increase can be attributed to several factors. First, the higher dielectric constant of water (ε ≈ 78.4) screens electrostatic interactions and stabilizes high-energy structures, thereby increasing the number of distinct minima. Second, the enhanced stability of TC conformers in PCM-water contributes to an overall increase in the total number of minima within this energy range, as indicated by the red numbers in Table S4 of the ESI.

In summary, the presence of a water solvent significantly affects protonation-state stability, *trans*/*cis* isomerization, and the number of stable conformers relative to the gas phase, particularly within 0–25 kJ/mol. These findings underscore the need to perform independent structural searches for short peptides across different environments to ensure that critical conformational states are not overlooked (Table 2).

**Table 2:** Total Number of Distinct Low-Energy (Extensive Search Using the NNP1 Model (the Second Column) and Re-Optimization at M06-2X/6-311+G­(d,p) (the Third Column) for Tripeptides in G-II)^a^

name	NNP1 min	M06–2X min	Δ*E* ^(1)^	Δ*F* ^(1)^	Δ*E* ^(2)^	RMSD
SSA	31	26(84%)	0.53	0.57	0.41	0.05
SAA	63	59(93%)	0.78	0.70	0.48	0.08
ASS	12	10(83%)	0.38	0.53	0.35	0.05
AAS	22	18(81%)	0.52	0.54	0.41	0.07
SAS	45	37(82%)	0.73	0.64	0.45	0.08
ASA	18	17(94%)	0.36	0.64	0.23	0.05
ASG	10	9(90%)	0.36	0.49	0.21	0.04
SAG	36	32(89%)	0.66	0.62	0.41	0.05
AGS	33	30(91%)	0.43	0.54	0.32	0.07
GAS	37	34(92%)	0.57	0.56	0.40	0.08
GSA	25	24(96%)	0.38	0.51	0.28	0.05
SGA	34	33(97%)	0.45	0.52	0.42	0.04
AAA	47	38(80%)	0.61	0.50	0.53	0.09
SSS	12	11(92%)	0.44	0.57	0.33	0.05

a The analysis of the structural similarity (RMSD in Å) and the energy and gradient differences (Δ*E* ^1^, and Δ*F* ^1^ and Δ*E* ^2^) for the local minimadefined in Figure 1c.

### Impact of Methylation on the Relative Stability of Protonation Sites and *Trans*/*Cis* Configuration

From Figure 3, we can see that the O-amide protonated conformers of AGG and SGG (belonging to mR^first^) are less stable than those of GGG, as indicated by the red arrows for O1- and O2-amide protonation. By comparing with other tripeptides in Figure 3, we can see a general trend that methylation at the first residue (denoted as mR^first^) favors N-protonated conformers over O-amide protonated ones due to its electronic effect, which enhances the proton affinity (PA) of N-terminal atoms. A similar trend was observed for other tripeptides in the water solvent (Figure S4 of the ESI). Following the approach in a previous study,^22^ we calculated the relative proton affinity (ΔPA) between mR^first^ tripeptides and GGG to further quantify the electronic effect. Specifically, ΔPA_mR1st_ is defined as PA_mR1st_–PA_GGG_, where PA_GGG_ is evaluated using the most stable linear form of GGG. The ΔPA values for Cα^1^- and N^1^-methylated tripeptides in the gas phase range from +13.1 to +27.4 kJ/mol and +50.0 to +67.2 kJ/mol, respectively (Table S5 of the ESI). These positive values confirm that mR^first^ was able to increase the N-terminal’s PA, with N^1^-methylation exerting a more pronounced effect than Cα^1^-methylation.

**Figure 3:**
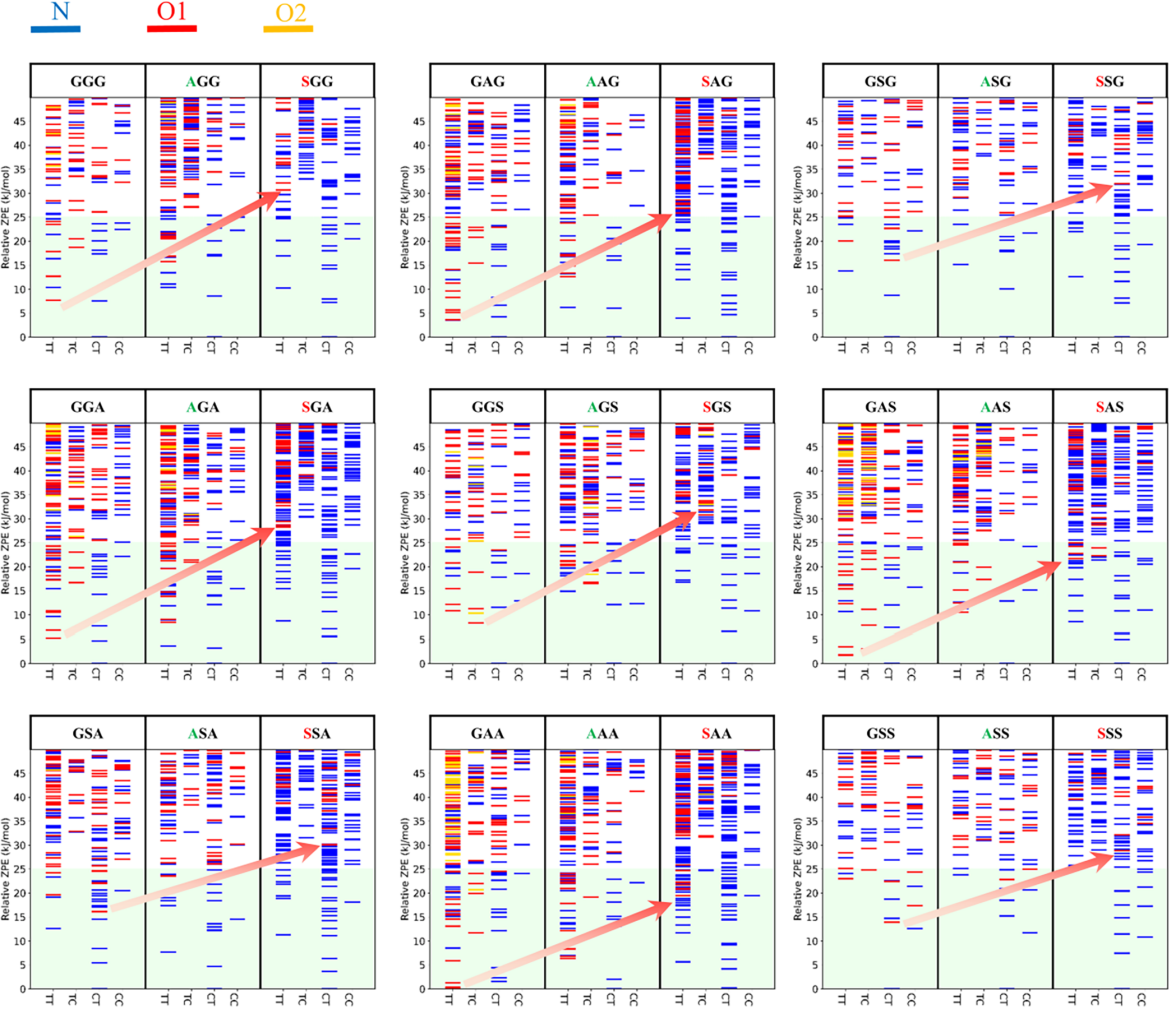
Zero-point corrected relative energy of the stable configurations of 27 tripeptides in gasphase at M06–2X/6–311+G­(d,p). Conformers with N-terminal, O1-amide and O2-amide site are shown in blue, red and orange. In general, N-protonated conformers were favored over Oprotonated conformers (with the exception of GAA). The red arrows show a trend that Cα^1^- and N^1^-methylation at first leads to further stabilization of the protonation site on the N-terminal site.

Furthermore, we analyze the impact of *N*-methylation on the third residue (NmR^third^, methyl group on R^3^ in Figure 1). By comparing GGG and GGS in Figure 4, we can see that *N*-methylation at the third residue consistently increases the relative stability of the *cis*-form at the second peptide bond, such as the TC and CC conformers, compared to their non-NmR^third^ counterparts. The steric effect can be observed by comparing the most stable TC and CC isomers in non-mR^third^ and NmR^third^ tripeptides. For example, in the gas phase, the TC and CC conformers of GGS (which has NmR^third^) are more stable than the corresponding forms in GGG (which is non-NmR^third^), as indicated by the green and cyan arrows in Figure 4. This effect can be consistently observed across nine comparisons in the gas phase (Figure 4) and in water solvent (Figure S8). This effect, attributed to steric interactions, has been discussed in the literature.^49–55^

**Figure 4:**
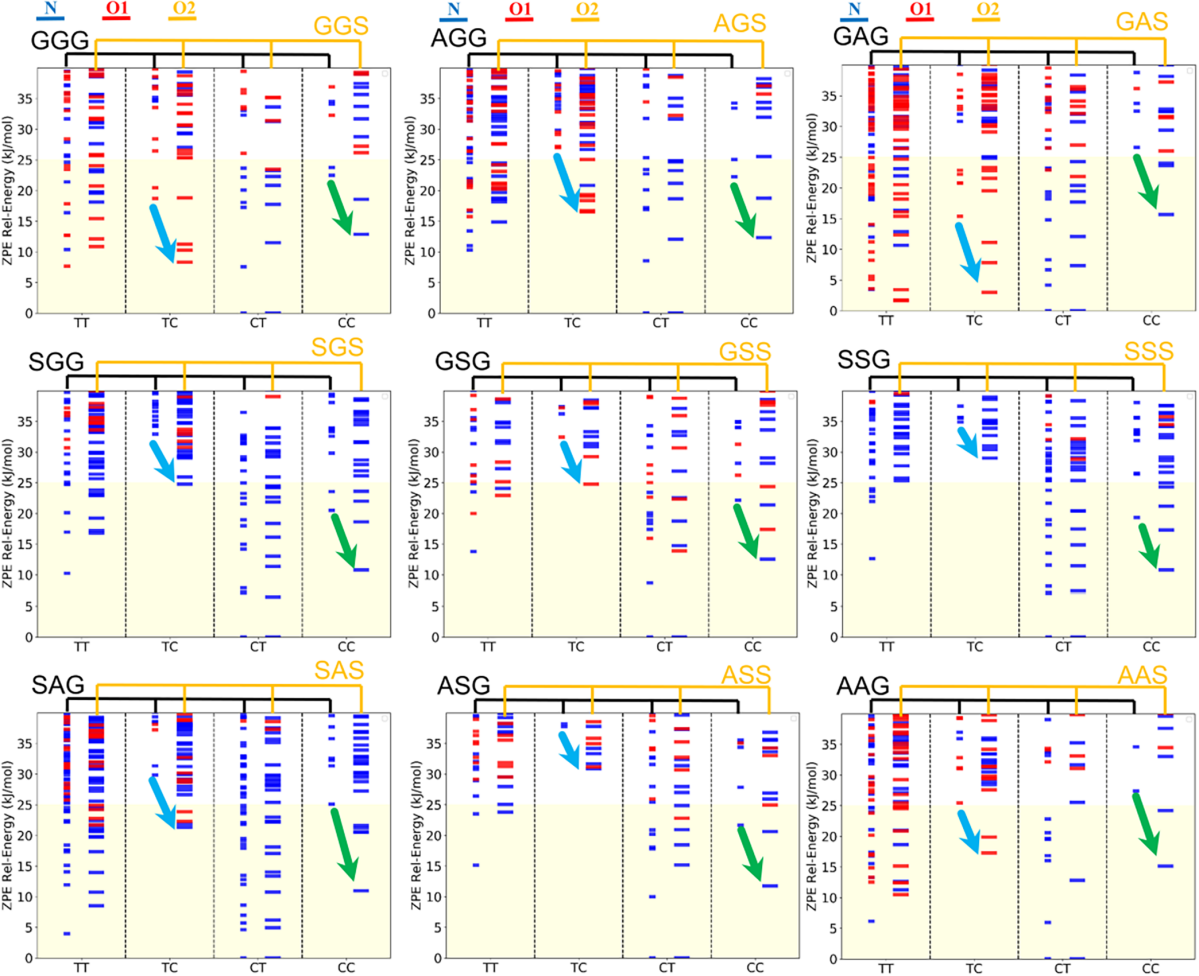
ZPE diagram compares nine pairs of non-NmR^third^ and NmR^third^ tripeptides in the gas phase.

Additionally, steric effects of *N*-methylation are observed when methylation occurs at the first and second residues. For the *N*-methylation of the first residue, the CT conformers represent the global minima in the gas phase. On the other hand, the stabilization of *N*-methylation is most evident in the CC conformers, which exhibit notably lower relative ZPE values than the nonmethylation conformers (Figure 5). However, the full extent of this stabilization (four comparisons between NmR1st and non-NmR1st) becomes apparent in PCM-water calculations, as shown in Figure S9.

**Figure 5:**
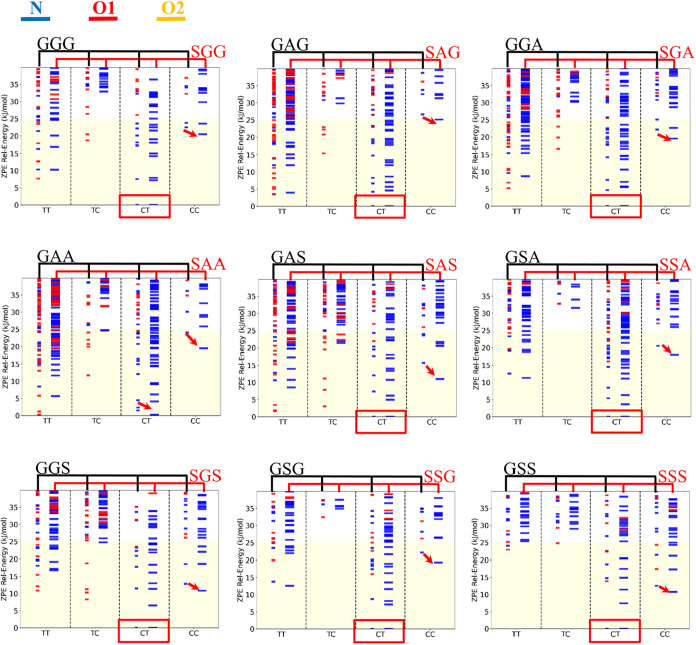
ZPE diagram compares nine pairs of non-NmR^first^ and NmR^first^ tripeptides in gasphase. The red boxes indicate two tripeptides that have CT as the global minimum.

We examine the *N*-methylation effect on the second residue by comparing only four pairs of non-NmR^second^ and NmR^second^ tripeptides without *N*-methylation at the first and third residue in gas phase and water, as shown in Figure 6. The NmR^second^ consistently yields lower relative ZPE values for both CC and CT conformers compared to their non-NmR^second^ counterparts. In Figure 6 for the gas phase, relative energies are referenced to the global minimum of each tripeptide pair. Because CT forms the global minimum (0 kJ/mol) in the four cases, CT relative energies are not directly comparable across different pairs. Therefore, we use the CC conformers, which maintain nonzero relative energies across all four pairs, to demonstrate the stability trend as a function of peptide composition. Overall, these findings demonstrate that *N*-methylation stabilizes *cis*-form peptide bonds both before and after the methylation site, suggesting that the steric and electronic effects of the *N*-methyl group propagate through the local peptide backbone.

**Figure 6:**
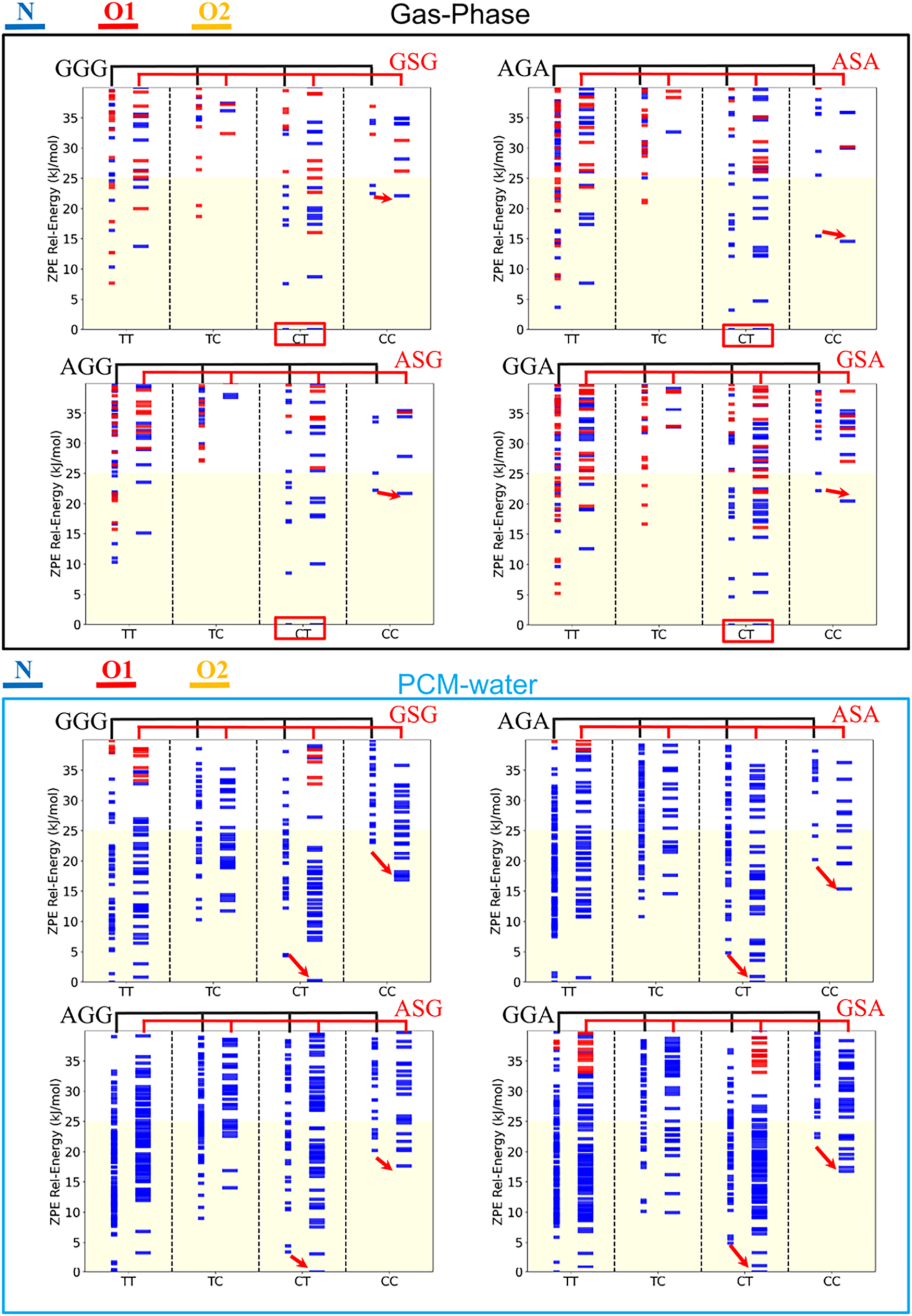
ZPE diagram compares four pairs of non-NmR^second^ and NmR^second^ tripeptides in gasphase and PCM-water. The red boxes indicate two tripeptides that have CT as the global minimum.

Methylation can influence the stability of protonation states and *cis*/*trans* isomers in both the gas phase and solvated environments, with the effects varying depending on the type and position of methylation (e.g., C- or N-methylation). A comprehensive understanding of these methylation-induced effects provides valuable insights into peptide structure–activity relationships, particularly in the context of functional group modifications and side-chain engineering.

### Vibrational Spectra of the Methylated Tripeptides in the Gas Phase

One of the primary applications of these low-energy conformers is to aid in the interpretation of IRPD spectra. Garand’s group reported that harmonic spectra at CAM-B3LYP/def2-TZVP/GD3BJ, with appropriate scaling factors, provide a pretty good description of peak positions. However, the energetic order of conformers with N-terminal and O-amide protonation sites is not well reproduced at the same level of theory.^22,30,47^ On the other hand, our previous work suggests that M06–2X/6–311+G­(d,p) offers a better relative energy than cam-B3LYP/def2TZVPP, and MP2/6–311+G­(d,p) based on comparison against the DLPNO–CCSD­(T)/aug-cc-pvTZ level of theory.^42^ Thus, we use M06–2X/6–311+G­(d,p) to simulate harmonic spectra of low-energy conformers of all 27 tripeptides.

For the eight types of tripeptides that consist of A and G, we found more low-energy conformers than those in Garand’s work^22^ (Figure S10). In their work, the low-energy minima at CAM-B3LYP/def2-TZVP/GD3BJ were selected for comparison with IR-IR double resonance to assist in the assignment of experimental spectra. Since M06–2X/6–311+G­(d,p) gives better relative energetics, our simulated results reproduce well the dominance of conformers with the O1-amide protonation site in GAA and GGA (Table S6). The low-energy conformers of the remaining 19 types of tripeptides remain to be tested (shown in Figure S11 of the ESI). In the following sections, we use two tripeptides (GGS and SSS, for which experimental IR spectra are not available) to highlight the comparison among TT, CT, TC, and CC low-lying energy minima in Figures 7 and 8.

**Figure 7:**
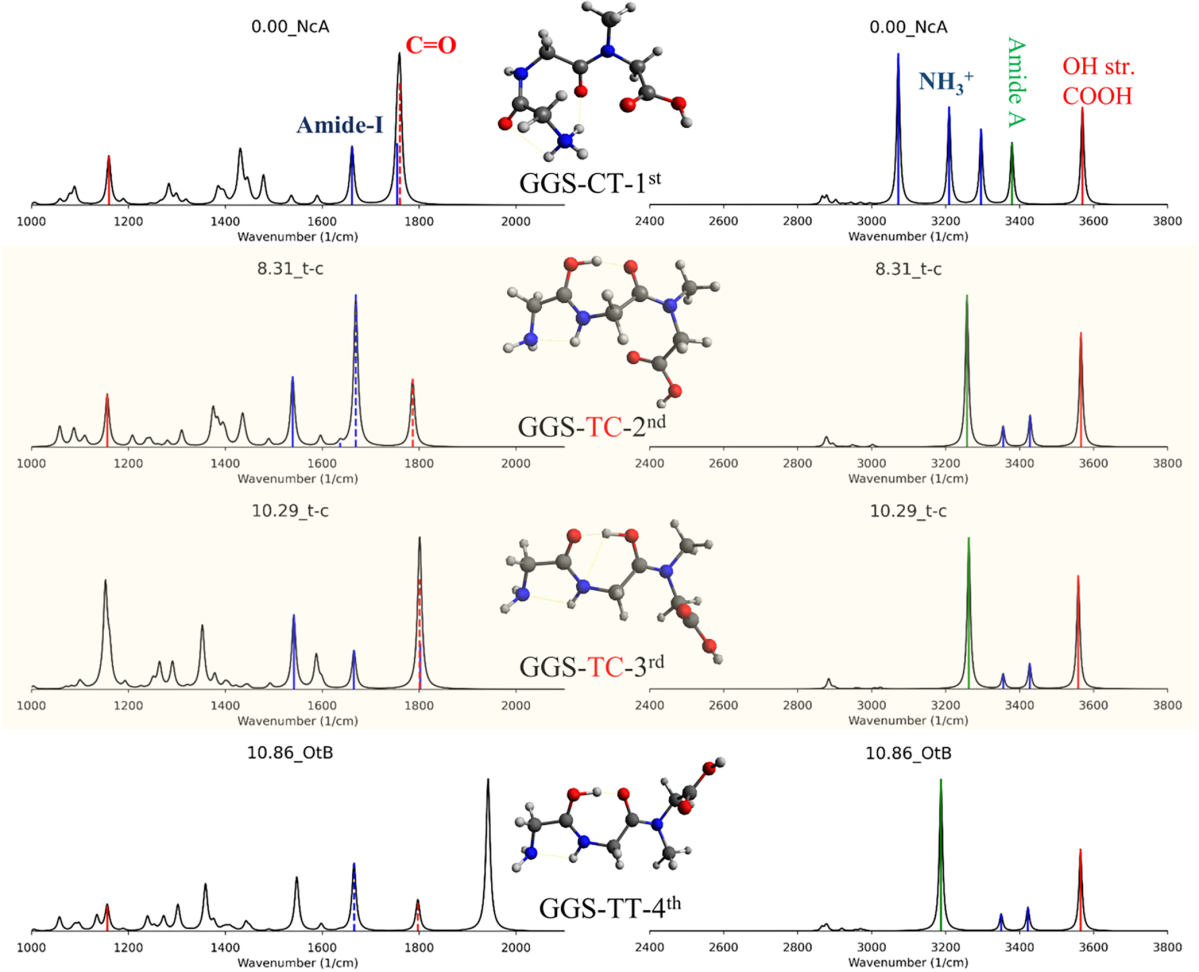
Scaled harmonic spectra of the four lowest-energy stable configurations of GGS computed at the M06–2X/6–311+G­(d,p) level. The harmonic frequencies in 1400–1900 and 2800–3800 cm^–1^ were scaled by 0.956 and 0.936 with a width of 5 cm^–1^.

**Figure 8:**
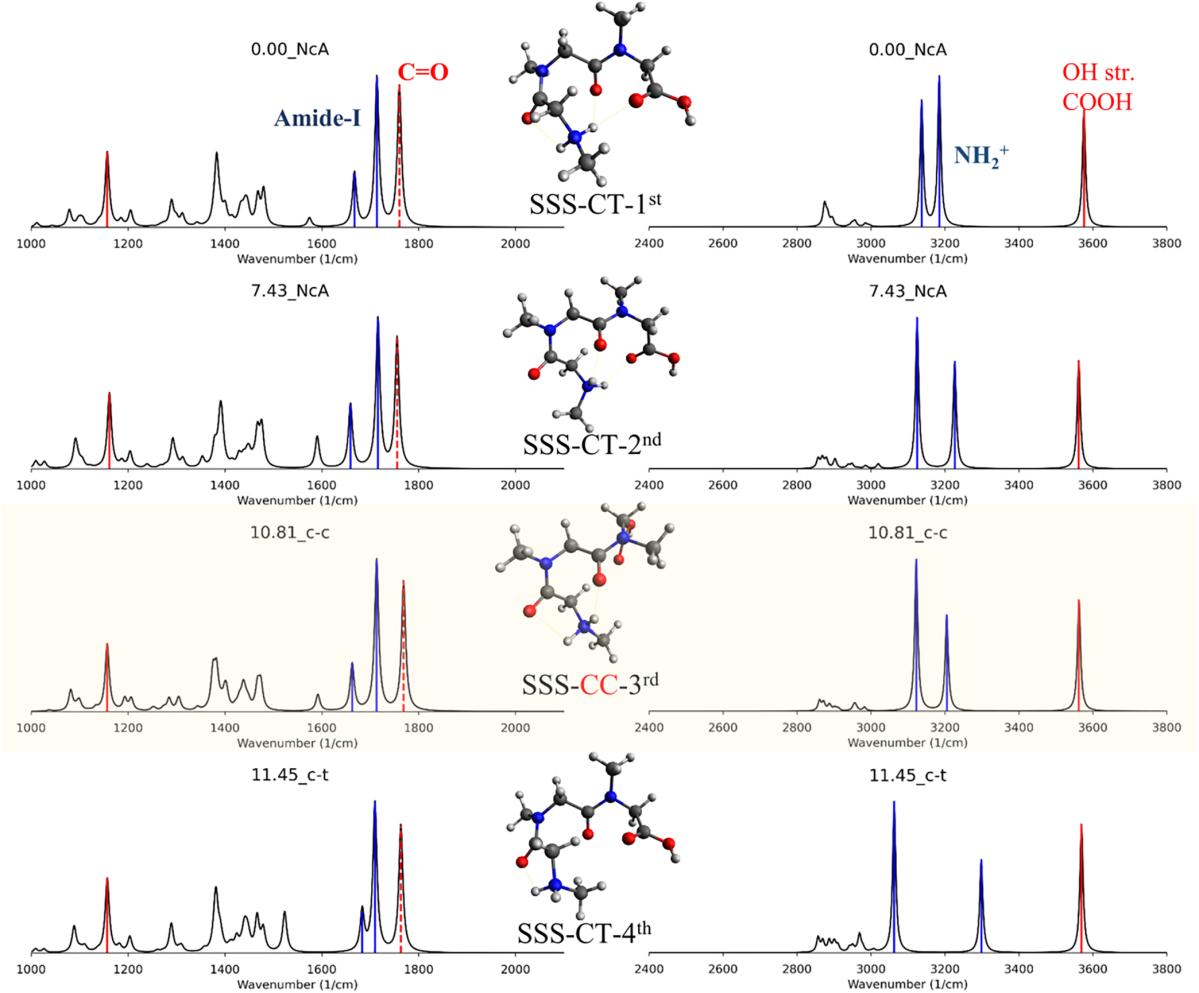
Scaled harmonic spectra of the four lowest-energy stable configurations of SSS computed at the M06–2X/6–311+G­(d,p) level. The same convention shown in Figure 7 is used to plot vibrational spectra.

Many studies compared measured and calculated IR spectra of TT and CT conformers to identify possible conformers within the 0–25 kJ/mol energy range.^22,30,47,56^ It has been suggested that experimental IRPD can identify conformers in this energy due to the kinetic trapping effect.^22,30,47,48^ Our structural databases indicate that TC and CC conformers can also fall within this low-energy range. For example, TC and CC conformers can be found in GGS and SSS, respectively, with relative energies of less than 15 kJ/mol. In particular, for these two tripeptides, we focus on two characteristic frequency regions: 1400–1900 and 2800–3800 cm^–1^. The harmonic frequencies in these two frequency regions were scaled by factors of 0.956 and 0.936, respectively, with a peak width of 5 cm^–1^.

For GGS, two TC conformers correspond to the second and third-lowest-energy minima, with their ZPE energies only 8.3–10.2 kJ/mol higher than the global CT minimum (Figure 7). Structurally, these two TC conformers share similar backbone geometries, differing only in the orientation of the carboxyl group, suggesting they are potentially interconvertible. In general, the spectra of the four minima in the low-energy frequency region of 1000–2000 cm^–1^, which contain fingerprint vibrational modes such as Amide-I, -II, -III, and C=O modes, are roughly similar except for the C=O mode of the CT conformers. Specifically, the C=O peak around 1758 cm^–1^ of the CT minima is lower in frequency than those of the TC (2nd-1786 and third-1800 cm^–1^) and TT (1794 cm^–1^) minima, since the CT backbone conformation can form a hydrogen bond between the H atom on the N-terminus and the O atom on the COOH group. Fortunately, CT can be distinguished from TT and TC conformers by analyzing the number of peaks and N–H modes in the 3000–3800 cm^–1^ region. The TC and TT conformers exhibit four strong peaks in this range, whereas the CT spectra have five peaks, as CT prefers the N-protonated site. Moreover, the N–H mode frequencies of the two CT (∼3261 cm^–1^) minima are much higher than those of the TT form (3185 cm^–1^), which may be due to the weaker hydrogen bond between N–H and the N-terminus in CT (2.1 Å) compared to the stronger interaction in TT minima (2.0 Å).

For SSS, we can see that the CC conformer represents the third most stable structure in Figure 8, with a relatively low ZPE of 10.8 kJ/mol. Similar to the GGS case, the CC conformer exhibits backbone vibrational modes in the 1000–2000 cm^–1^ region that closely resemble those of the CT minima. Unlike GGS, the CC spectra in the 3000–3800 cm^–1^ region were roughly similar to the spectra of the second stable CT conformer (CT-2nd). Only one N–H peak differs between the CT (3203 cm^–1^) and CC (3221 cm^–1^) spectra, with a difference of approximately 18 cm^–1^.

Overall, the 1000–2000 cm^–1^ region, which primarily reflects backbone vibrations, exhibits rough similarities between TC and CC conformers compared to TT and CT conformers. In the case of GGS, analyzing the 3000–3800 cm^–1^ region can help distinguish TC from CT conformers. However, distinguishing between CC and CT-second remains challenging in SSS, underscoring the need for *cis*- and *trans*-conformer searches to compare experimental and calculated spectra.

## Conclusions

In this work, we employed the DL-NNP framework to efficiently explore the conformational space of triglycine and 26 methylated tripeptides in both the gas phase at the first-principles level (NNP0 and NNP1) and in implicit water via PCM (NNP0w and NNP1w). The NNP models, trained on GGG data and 12 binary methylated tripeptides, can accurately predict the atomic environments of all 27 tripeptides. Furthermore, the NNP models demonstrated sufficient transferability, effectively adapting from gas-phase to water-solvent models, achieving average E-MAEs of 1 and 2 kJ/mol for NNP1 and NNP1w, respectively. Based on our experience with NNP methods, we expect that the NNP1 and NNP1w models can predict *N*-methylated alanine tripeptides with reasonable accuracy. These models are suitable for MD simulations, with accuracy further improvable through active learning approaches that incorporate additional high-energy configurations.

Regarding transferability to larger peptides, our models can serve as a foundation for studying larger systems in both explicit and implicit solvation. However, achieving high accuracy for larger peptides will likely require: (1) augmenting the training set with critical configurations from larger peptides, and (2) potentially employing more advanced NNP architectures to handle increased system complexity. Looking forward, these models will provide a foundation for developing more sophisticated approaches, including generative models for peptide conformational sampling.

These NNP models were used to search structurally distinct minima. Minima within an energy range of 0–50 kJ/mol using the NNP models were reoptimized by M06–2X. Subsequently, low-energy conformers within the 0–25 kJ/mol energy range, featuring different *trans*/*cis* isomers and protonation sites, were identified. The same procedure is repeated with NNP1w to identify low-energy conformers with PCM-water. The number of minima in the water-solvent environment is significantly higher than that of their counterparts in the gas phase. Furthermore, the O-amide protonation and TC conformers were found to be less stable in PCM-water than in the gas phase.

By analyzing the structures and relative energies of these low-energy conformers, we found that N-/Cα-methylation at the first residue favors N-terminal protonation and *N*-methylation at the third residue enhances the stability of CC and TC conformers. Vibrational spectra of all 27 tripeptides were simulated and compared to available experimental data on 8 types of tripeptides.^22^ The CC and TC conformers can be distinguished from TT and CT conformers by their characteristic N–H or NH^+^ vibrational features in the high-frequency region. We hope these low-energy conformers at M06–2X/6–311+G­(d,p) identified with the assistance of DL-NNP will inspire further validation through future experimental observations, ultimately providing a comprehensive and robust understanding of the conformational behavior of methylated tripeptides.

## Supplementary Material



## Notes

Active learning workflow for PCM-water; correlation plots comparing M06–2X and NNPw models; distribution of distinct M06–2X minima within 0–25 kJ/mol in the gas phase and PCM-water; zero-point corrected relative energies of 27 tripeptides in PCM-water; comparison of IR spectra and configurations with the 2022 literature; training statistics for NNPw models; total number of NNP1 and NNP1w minima (<50 kJ/mol); HSA calculations at 300 K and rescaled harmonic spectra at the M06–2X/6–311+G­(d,p) level; Cartesian coordinates of all M06–2X minima (PDF)
