# Supplementary material for: A Deep-Learning Neural Network Potential Accelerated First-Principles Study on the Structural Changes Modulated by Methylation and Solvation in 27 Protonated Tripeptides
Source: J Phys Chem A. 2025 Nov 18;129(47):10859–72. doi: 10.1021/acs.jpca.5c06004 (PMC12670389; doi:10.1021/acs.jpca.5c06004)
Supplement: Supplementary file 1 [file jp5c06004_si_001.pdf]

A Deep-Learning Neural Network Potential Accelerated First-Principles Study on the Structural Changes Modulated by Methylation and Solvation in 27 Protonated Tripeptides

Dong Cao Hieu,[a,b,c] Po-Jen Hsu,[a,] and Jer-Lai Kuo*,[a,c,d]

[a.] Institute of Atomic and Molecular Sciences, Academia Sinica, Taipei, 10617, Taiwan
[b.] Molecular Science and Technology Program, Taiwan International Graduate Program, Academia Sinica, Taipei, 11529, Taiwan
[c.] International Graduate Program of Molecular Science and Technology (NTU-MST), National Taiwan University, Taipei 10617, Taiwan
[d.] Department of Chemistry, National Tsing Hua University, Hsinchu 30013, Taiwan.

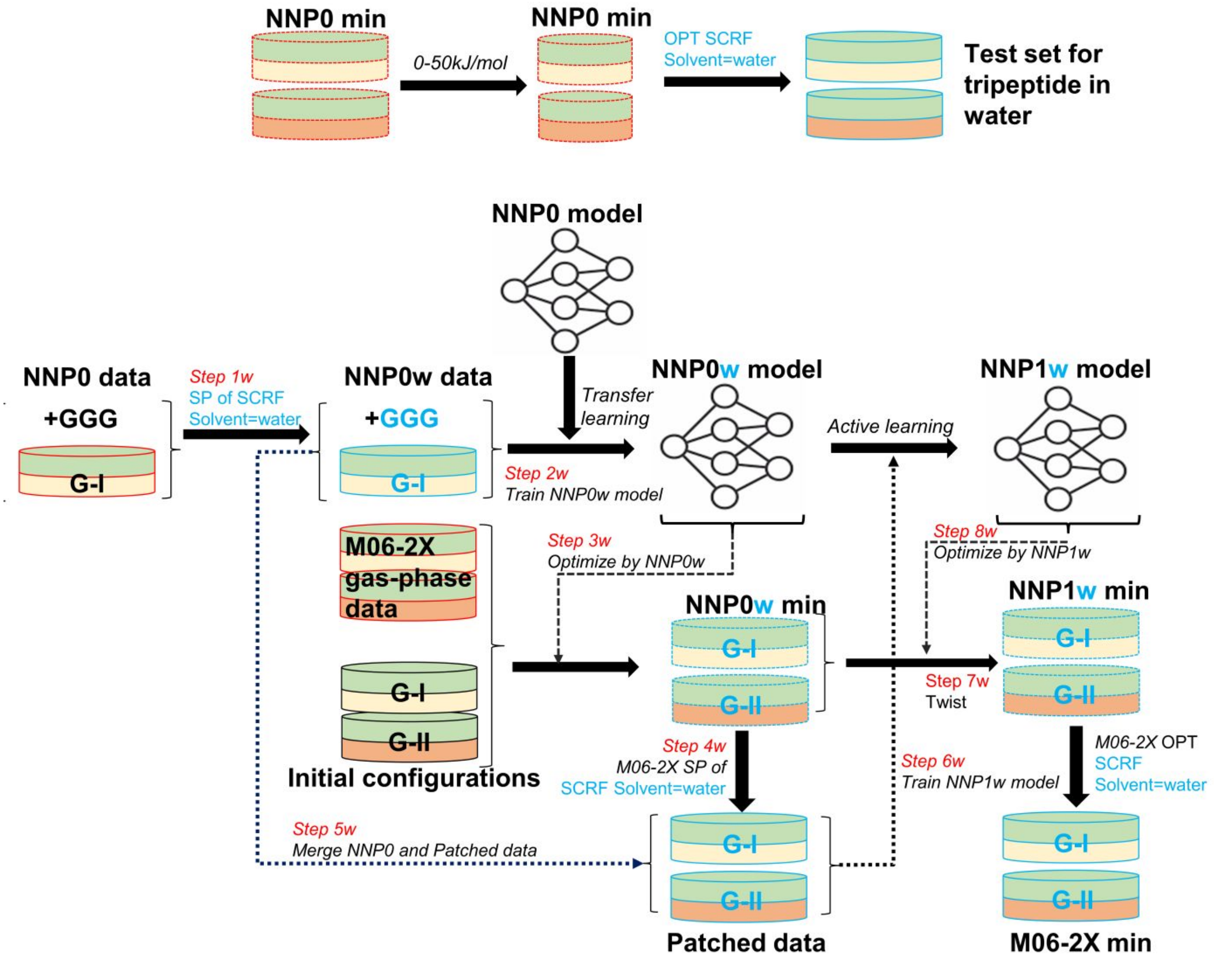

Figure S1: The active learning workflow employed for training the NNP0w and NNP1w models, followed by an extensive conformational search utilizing the NNP1w model. The classification of methylated tripeptides into Group I (G-I) and Group II (G-II) is the same as Figure 1.

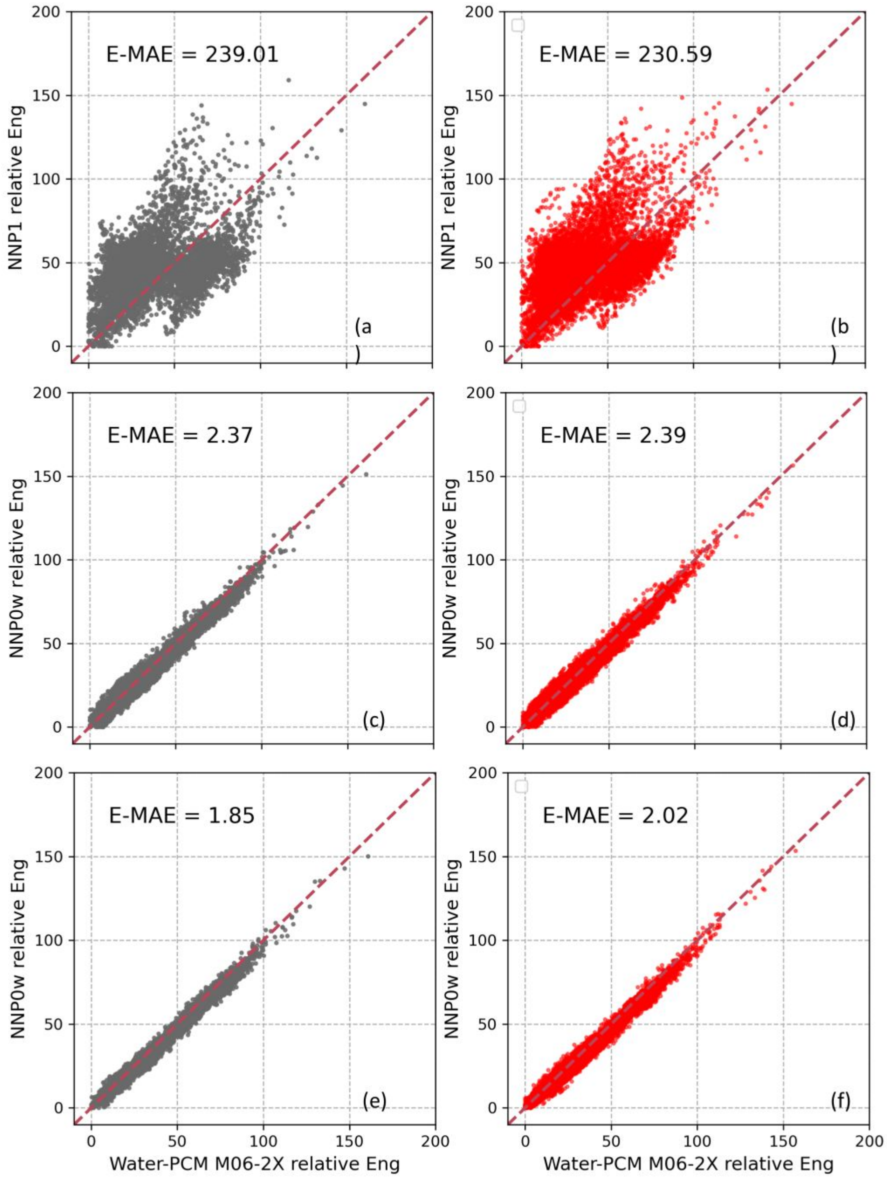

Figure S2: The correlation between water-PCM M06-2X relative energy (x-axis) and NNP1 relative energy (y-axis) on the water methylated tripeptides test set of Group I (a) and Group II (b). The correlation between PCM-water M06-2X relative energy (x-axis) and NNP0w relative energy (y-axis) on the test set of Group I (c) and Group II (d).

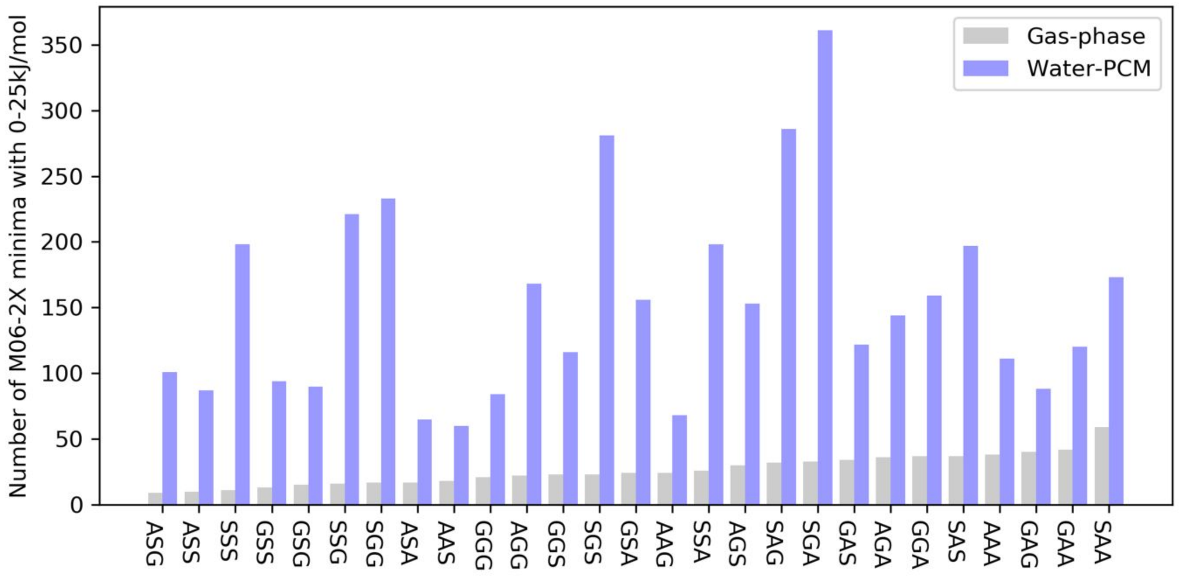

Figure S3: Number of distinct M06-2X minima within a relative energy range of 0–25kJ/mol in the gas phase and water-PCM.

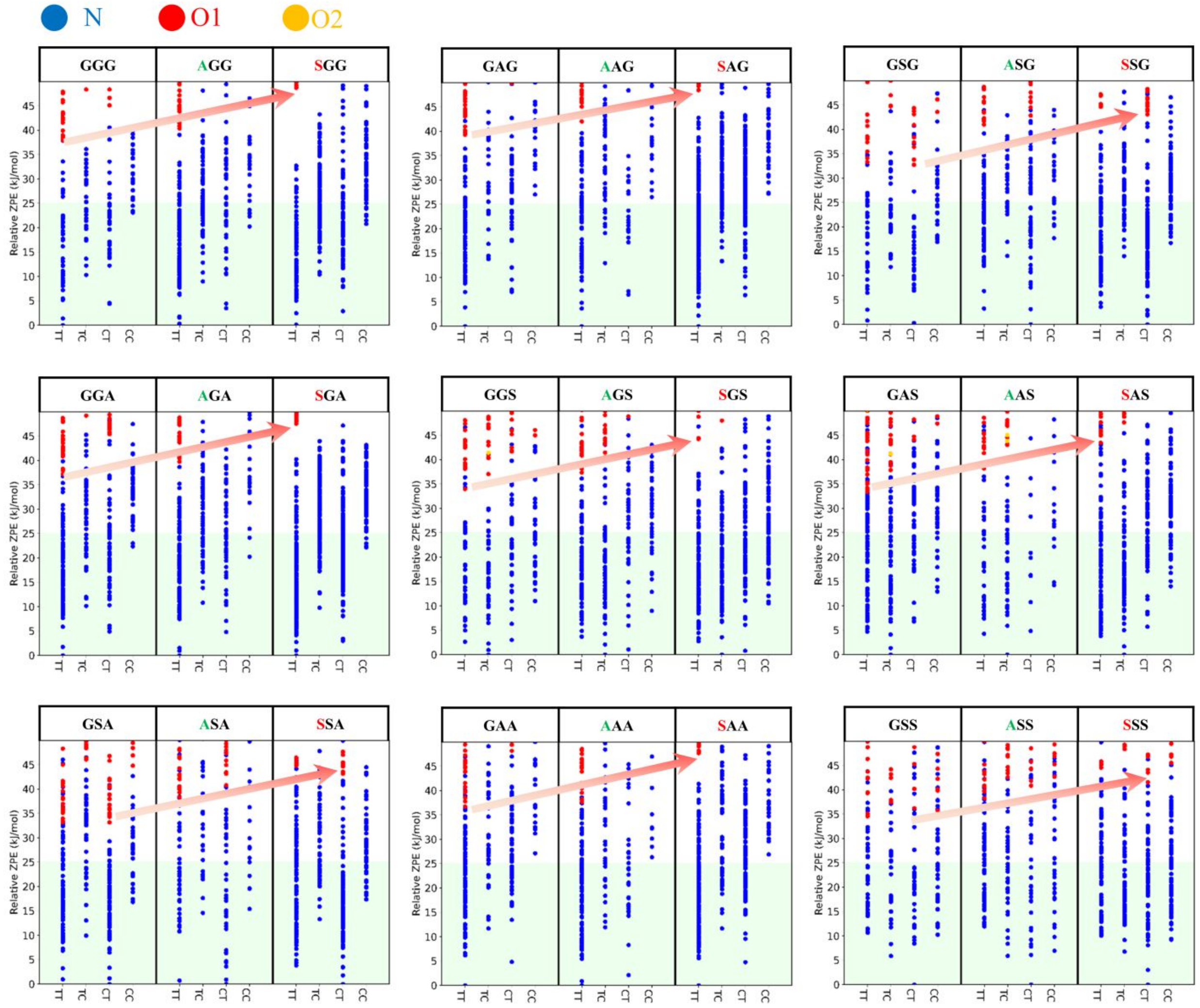

Figure S4: Zero-point corrected relative energy of the stable configurations of 27 tripeptides in PCM-water at M06-2X/6-311+G(d,p).

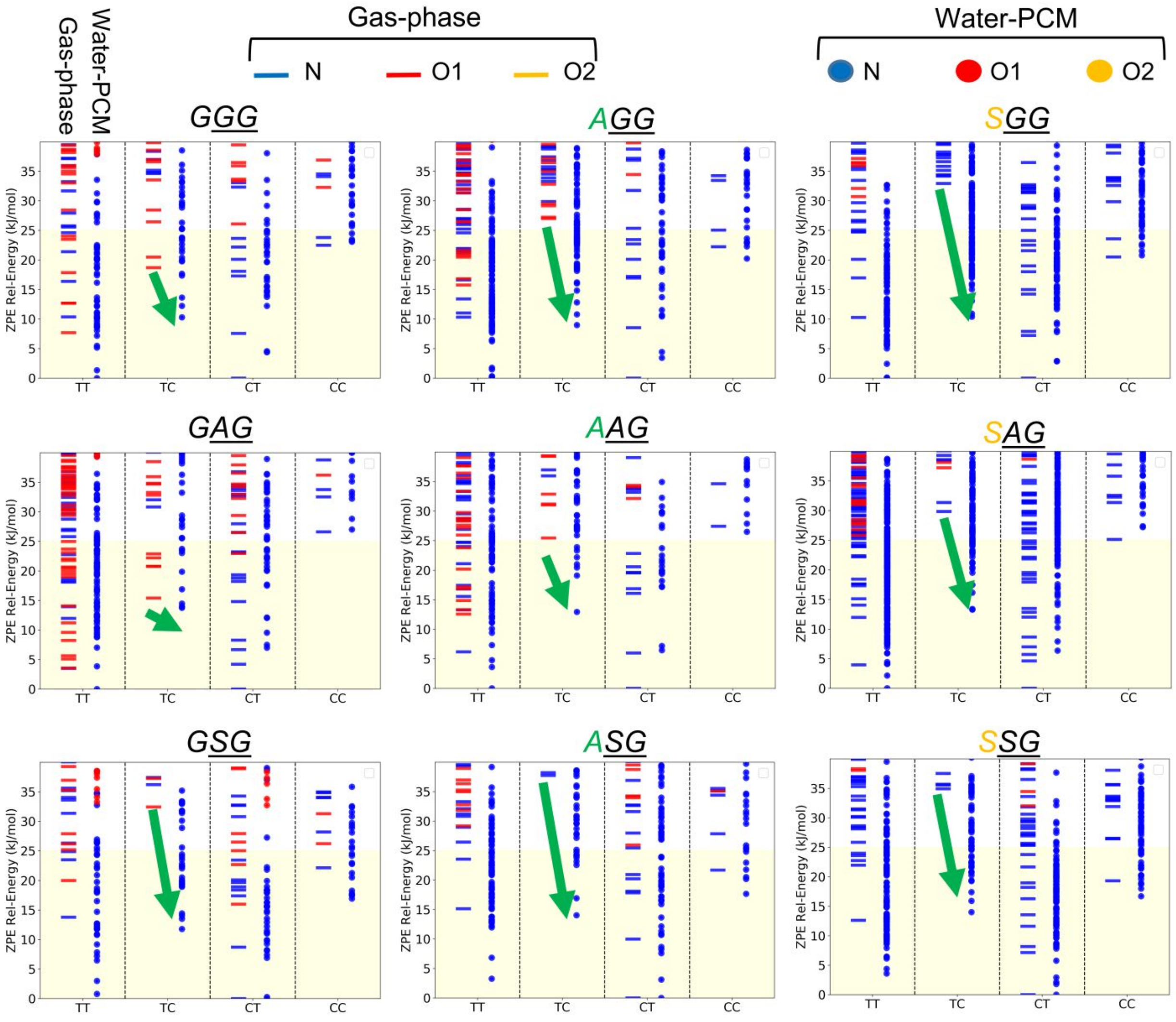

Figure S5: The ZPE diagram of nine methylated tripeptides in the gas phase (hline markers) and water-PCM (point markers).

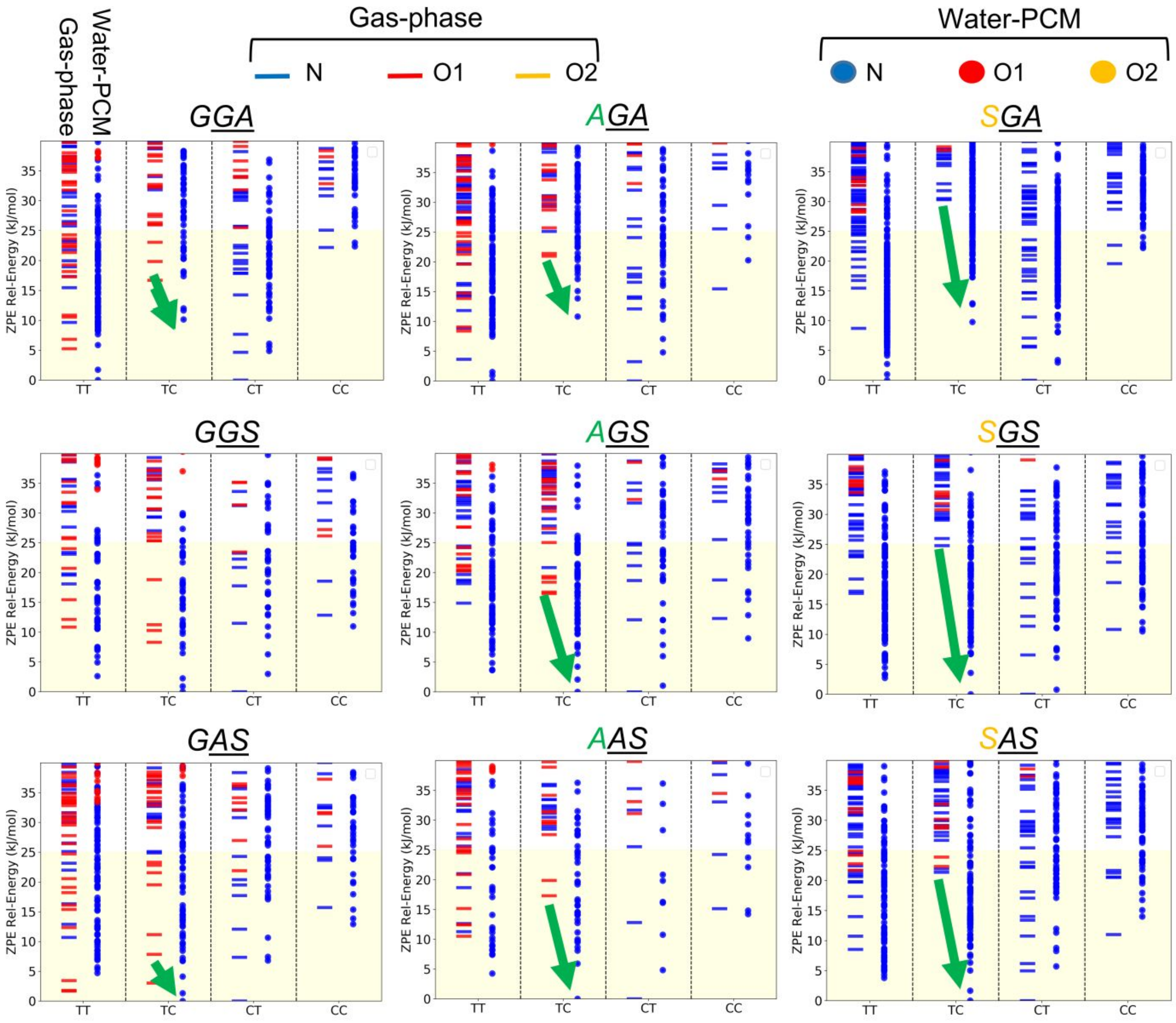

Figure S6: The ZPE diagram of nine methylated tripeptides in the gas phase (hline markers) and water-PCM (point markers).

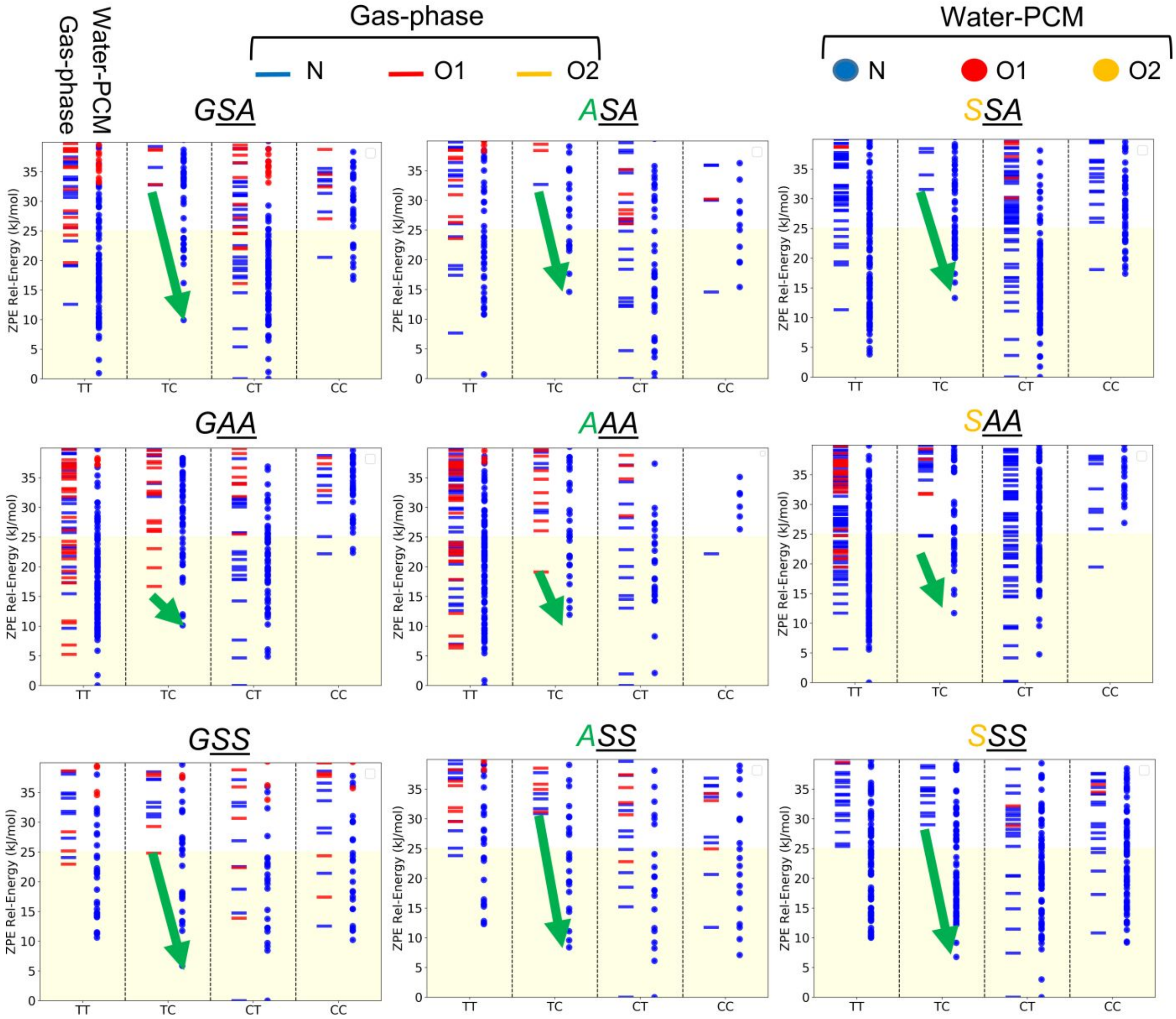

Figure S7: The ZPE diagram of nine methylated tripeptides in the gas phase (hline markers) and water-PCM (point markers).

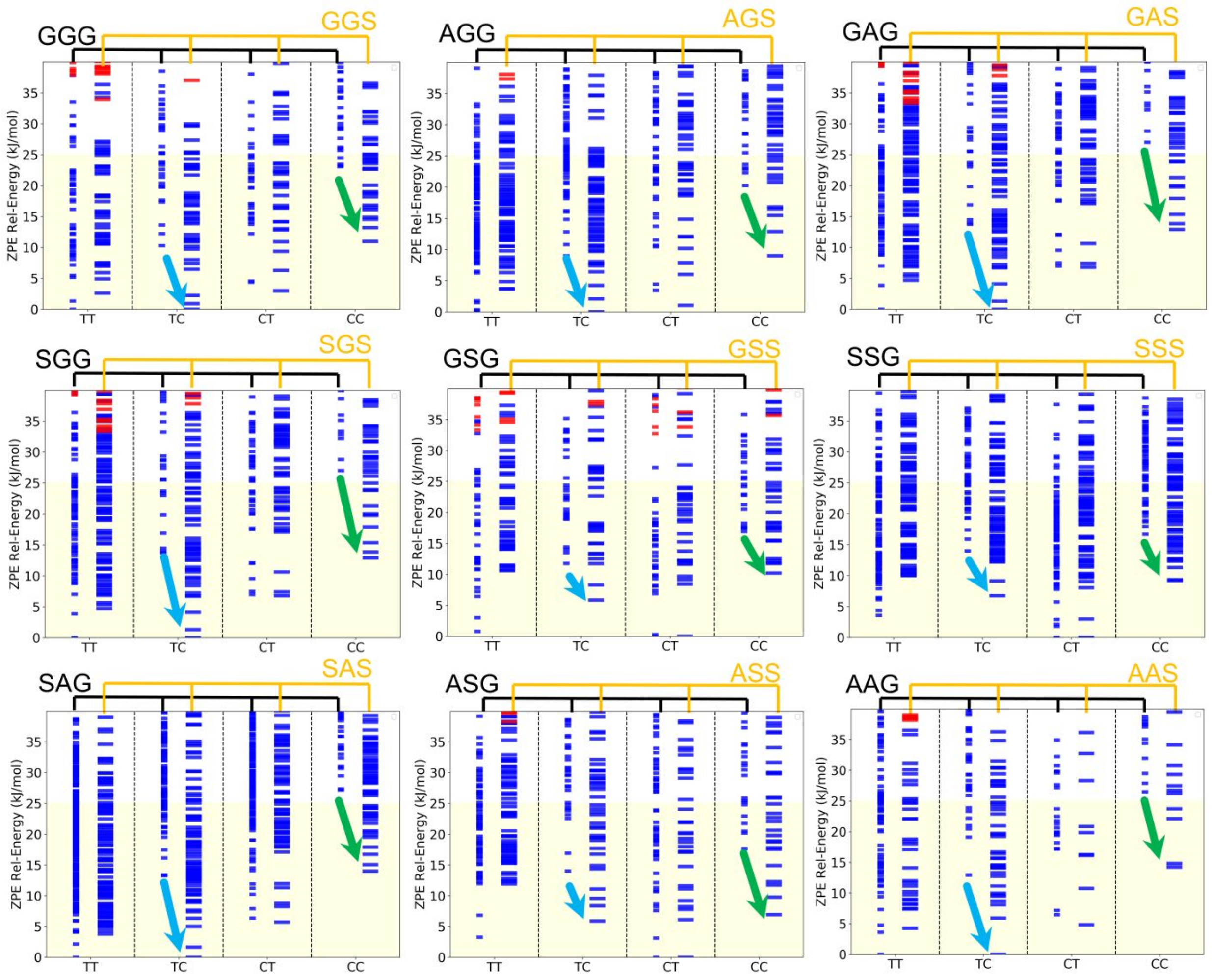

Figure S8: The ZPE diagram compares nine pairs of non-mR^{3rd} and mR^{3rd} tripeptides in PCM-water. The color-coding scheme for different protonation site is the same as Figure 3.

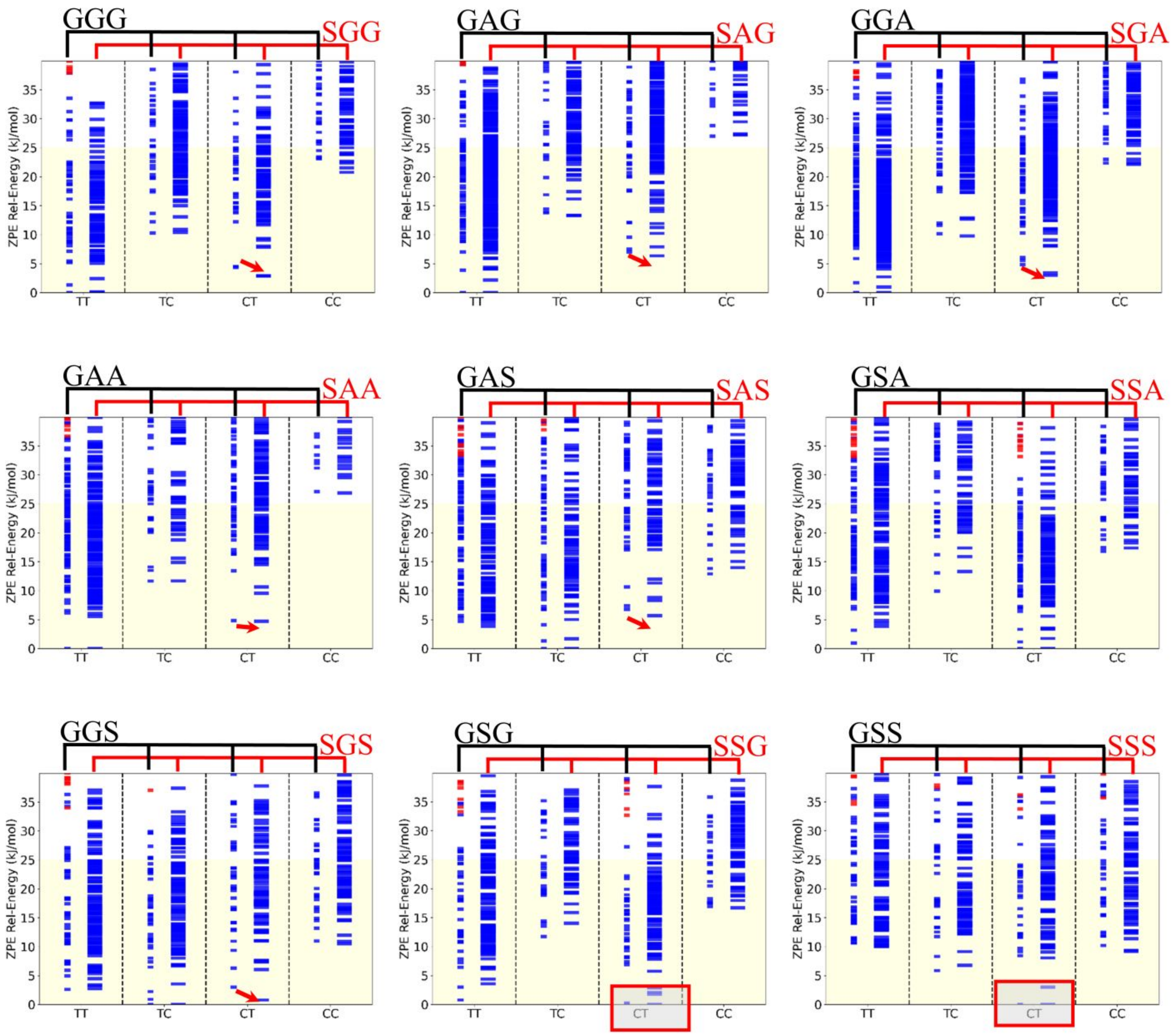

Figure S9: The ZPE diagram compares nine pairs of non-mR^{1st} and mR^{1st} tripeptides in PCM-water. The color-coding scheme for different protonation site is the same as Figure 3.

Figure S10: Comparison of IR spectra and configurations between the 2022 paper and our CAM-B3LYP/def2-TZVP/GD3BJ minima re-optimized from our M06-2X minima.

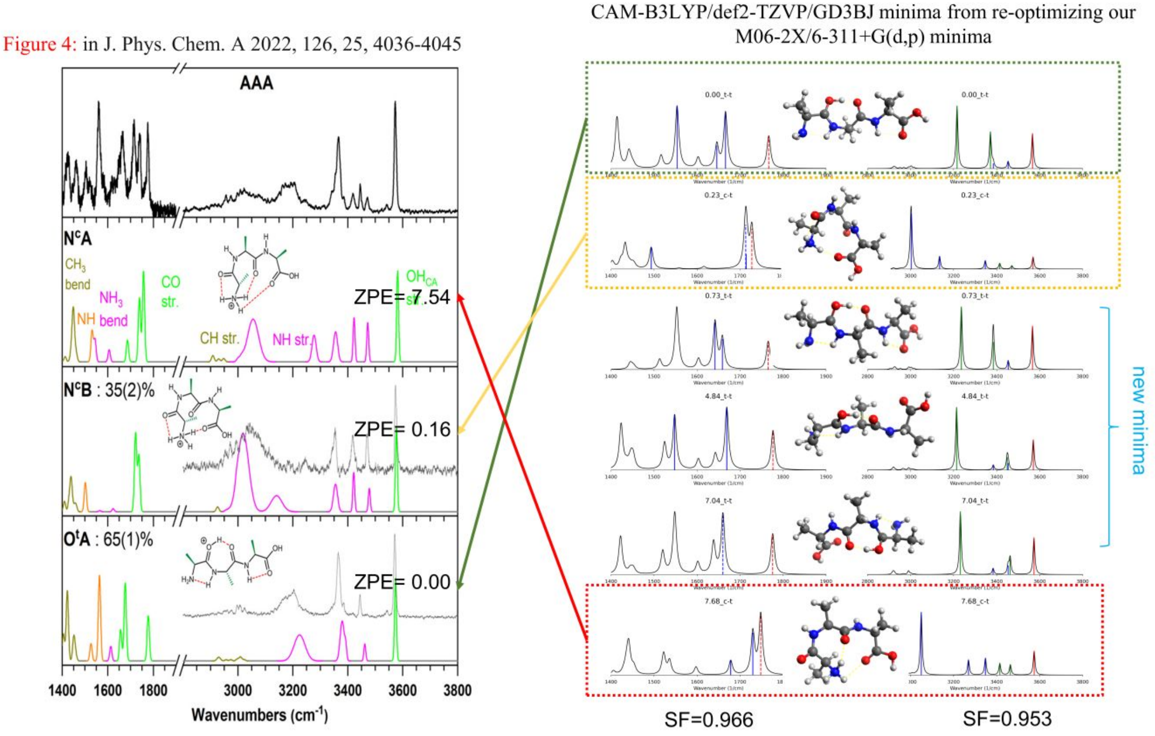

CAM-B3LYP/def2-TZVP/GD3BJ minima from re-optimizing our M06-2X/6-311+G(d,p) minima

Figure 5: in J. Phys. Chem. A 2022, 126, 25, 4036-4045

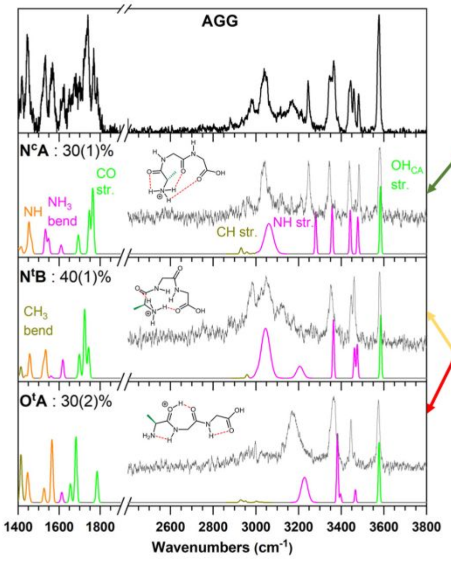

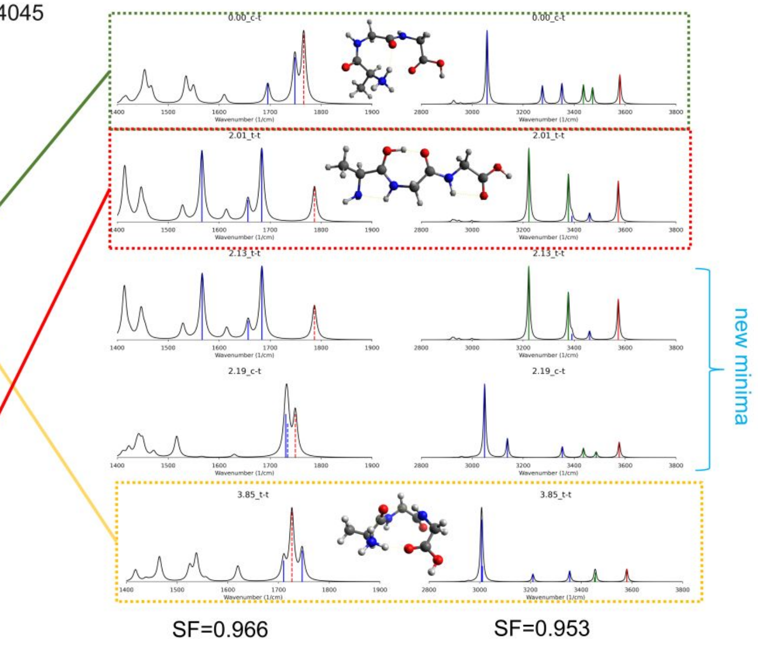

CAM-B3LYP/def2-TZVP/GD3BJ minima from re-optimizing our M06-2X/6-311+G(d,p) minima

Figure 6: in J. Phys. Chem. A 2022, 126, 25, 4036-4045

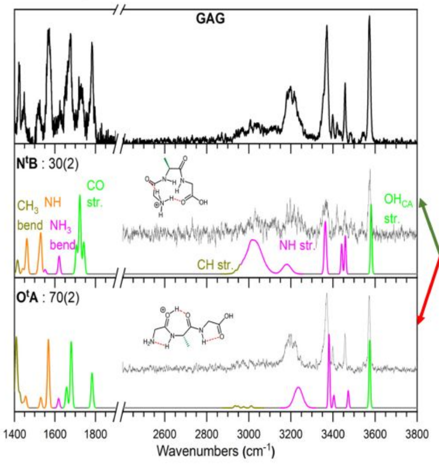

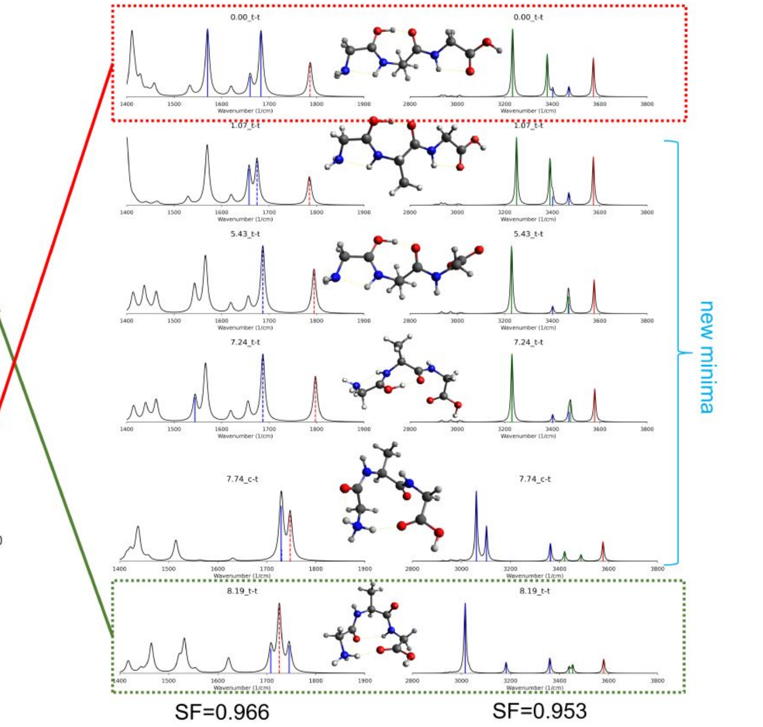

Figure 7: in J. Phys. Chem. A 2022, 126, 25, 4036-4045

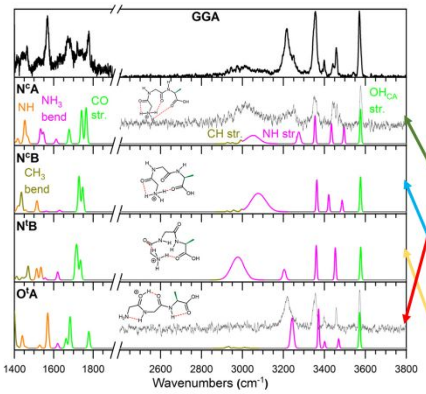

CAM-B3LYP/def2-TZVP/GD3BJ minima from re-optimizing our M06-2X/6-311+G(d,p) minima

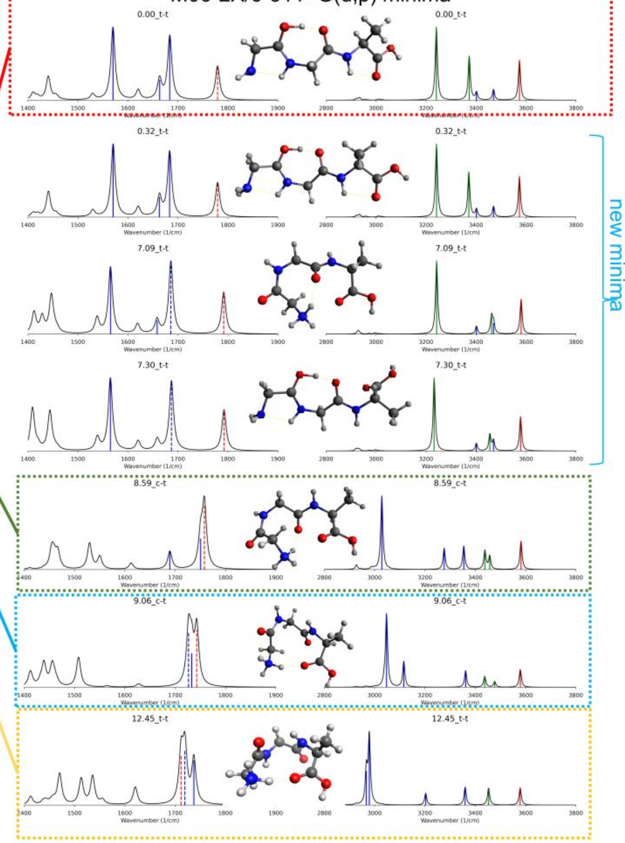

Figure 8: in J. Phys. Chem. A 2022, 126, 25, 4036-4045

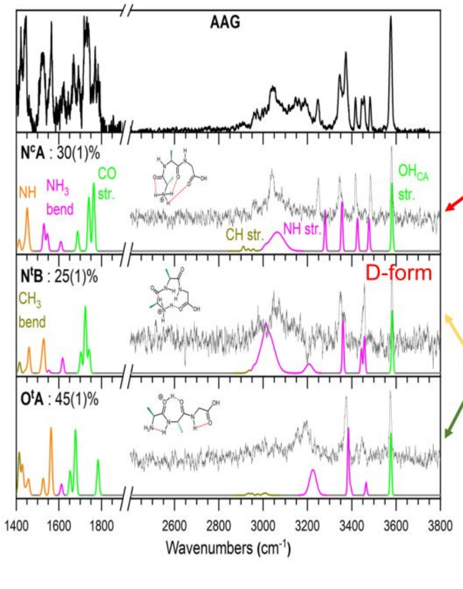

CAM-B3LYP/def2-TZVP/GD3BJ minima from re-optimizing our M06-2X/6-311+G(d,p) minima

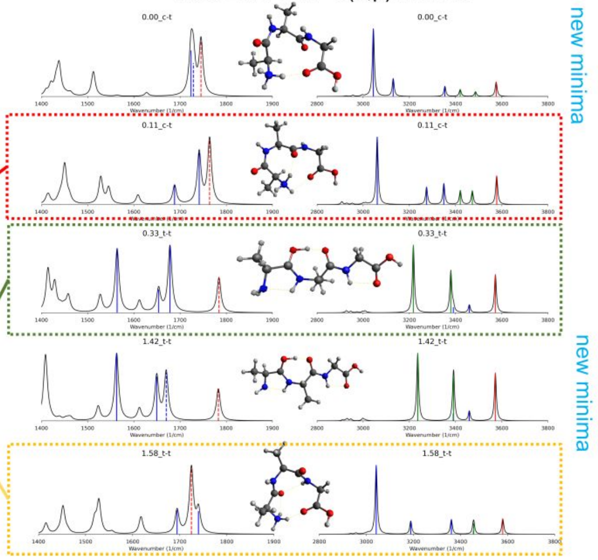

SF=0.966 SF=0.953

CAM-B3LYP/def2-TZVP/GD3BJ minima from re-optimizing our M06-2X/6-311+G(d,p) minima

Figure 9: in J. Phys. Chem. A 2022, 126, 25, 4036-4045

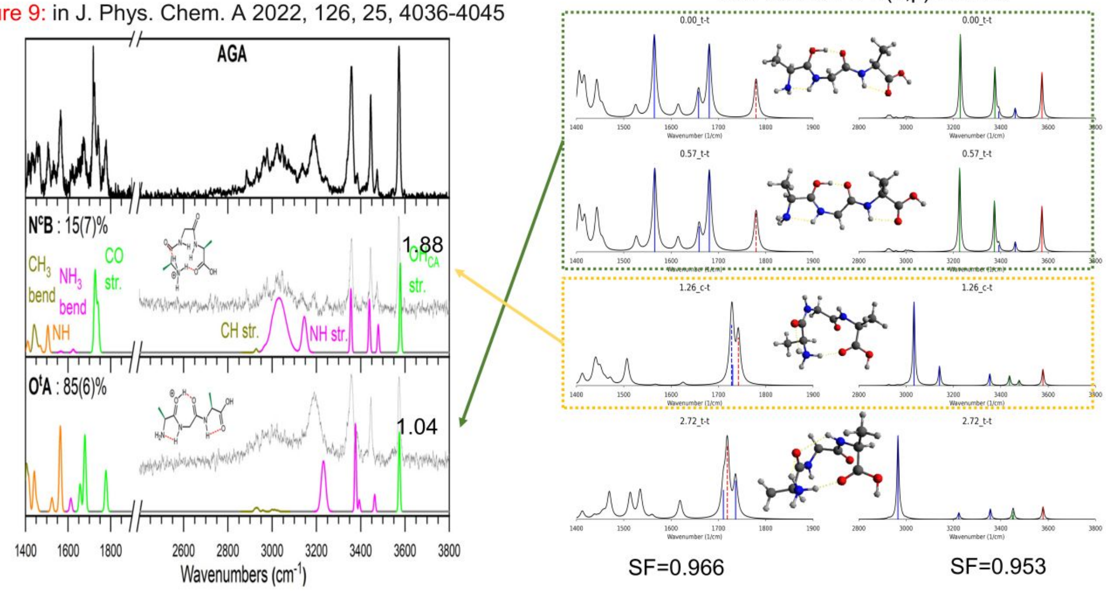

CAM-B3LYP/def2-TZVP/GD3BJ minima from re-optimizing our M06-2X/6-311+G(d,p) minima

Figure 10: in J. Phys. Chem. A 2022, 126, 25, 4036-4045

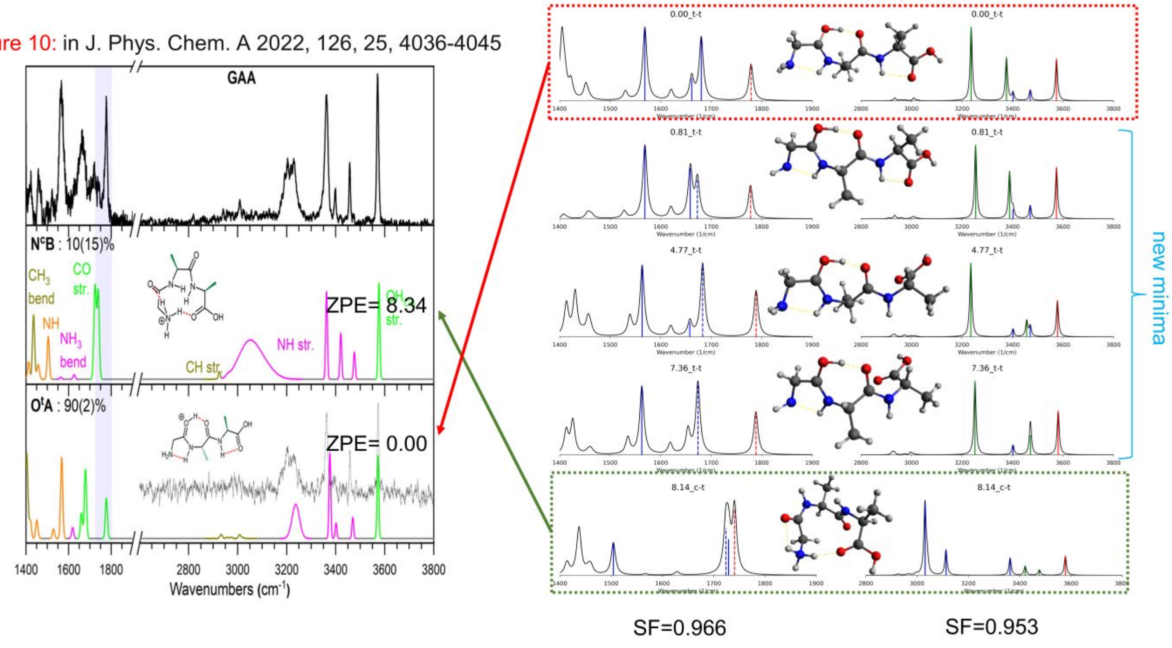

Table S1: The number of trials (second row), initial configurations (third row), successful rate (fourth row), test set (fifth row), training set and E/F-MAE of the NNP0 and NNP1 models for the G-I and G-II.

**G-I**		**GGG**	**AGG**	**GAG**	**GGA**	**AAG**	**AGA**	**GAA**	**SGG**	**GSG**	**GGS**	**SSG**	**SGS**	**GSS**
Number of trials			945*2	945*2	945*2	945*4	945*4	945*4	945*3	945*1	945*1	945*3	945*3	945*1
Number of Initial configurations		945	1846	1815	1839	3544	3590	3530	2738	898	899	2594	2598	853
Successful rate			97.6%	96.0%	97.3%	93.7%	95.0%	93.4%	96.6%	95.0%	95.1%	91.5%	91.6%	90.2%
Test set			2019	2170	2902	2561	2722	2456	2053	2377	2168	3160	2848	2968
NNP_0	Training data	4000	2327	2160	2289	2981	3076	2731	2056	2536	2212	3254	3232	2677
	E-MAE (kJ/mol)		1.52	1.67	1.49	1.84	1.71	2.01	1.29	1.31	1.11	1.49	1.39	1.42
	F-MAE (kJ/mol)		1.58	1.76	1.51	1.85	1.70	2.01	1.32	1.36	1.24	1.53	1.43	1.44
NNP_1	Training data	4000	+184	+337	+159	+344	+838	+206	+576	+521	+267	+341	+613	+381
	E-MAE (kJ/mol)		1.00	1.15	1.00	1.36	1.10	1.41	0.72	0.75	0.70	0.84	0.77	0.76
	F-MAE (kJ/mol)		1.26	1.47	1.22	1.45	1.27	1.59	0.85	0.89	0.80	0.96	0.88	0.89

G-II		AAA	AAS	ASA	ASG	AGS	ASS	SAA	GAS	SAG	SAS	GSA	SGA	SSA	SSS
Number of trials		945*8	945*4	945*4	945*2	945*2	945*2	945*12	945*2	945*6	945*6	945*2	945*6	945*6	945*3
Number of Initial configurations		6891	3369	3408	1754	1752	1660	10228	1721	5256	4984	1745	5329	5054	2471
Successful rate		91.1%	89.2%	90.2%	92.8%	92.7%	87.8%	90.2%	91.0%	92.7%	88.0%	92.3%	93.9%	89.1%	87.1%
Test set		2866	3204	3223	2933	3090	3446	2952	2781	2738	3262	2757	2826	3414	3378
NNP_0	Training data	0	0	0	0	0	0	0	0	0	0	0	0	0	0
	E-MAE (kJ/mol)	2.11	2.44	2.24	1.60	2.15	2.53	1.97	2.74	1.74	2.36	2.53	1.43	1.85	1.90
	F-MAE (kJ/mol)	2.10	2.51	2.45	1.64	2.29	2.43	1.98	2.66	1.79	2.37	2.12	1.50	1.85	1.69
NNP_1	Training data	+695	+1743	+1911	+695	+926	+1445	+1473	+910	+943	+1630	+966	+844	+2078	+334
	E-MAE (kJ/mol)	1.54	1.19	1.36	0.84	1.14	1.22	1.01	1.67	1.02	1.11	1.28	0.85	1.13	0.92
	F-MAE (kJ/mol)	1.60	1.38	1.37	1.02	1.37	1.34	1.10	1.74	1.07	1.29	1.49	0.96	1.11	0.99

Table S2: The total number of distinct NNP1 and NNP1w (<50 kJ mol^{-1}) minima.

**Name**	**NNP1 min**	**NNP1w min**	**Name**	**NNP1 min**	**NNP1w min**
SSA	263	464	GGG	119	261
SAA	339	635	AGG	177	349
ASS	99	223	GAG	196	273
AAS	147	185	GGA	238	393
SAS	301	601	AAG	146	231
ASA	118	276	GAA	238	378
ASG	104	235	AGA	221	357
SAG	308	674	SGG	114	429
AGS	172	307	GSG	102	360
GAS	189	391	GGS	115	242
GSA	155	323	SSG	152	382
SGA	338	696	GSS	77	185
AAA	188	575	SGS	223	457
SSS	163	355			

Table S3: The total number of distinct low-energy (<25 kJ mol^{-1}) minima after perform the extensive search using the NNP1w model (the second column) and of distinct M06-2X minima (the third column) for G-I and G-II groups. The analysis of the structural similarity (RMSD in Å) and the energy and gradient differences (ΔE^1, and ΔF^1 and ΔE^2) for the local minima derived from NNP1-w and M06-2X.

Name	NNP1-w min	M06-2X min	$\Delta E^{(1)}$	$\Delta F^{(1)}$	$\Delta E^{(2)}$	RMSD
GGG	99	84 (85%)	1.19	1.01	0.6	0.10
AGG	204	168(82%)	1.74	1.12	0.81	0.12
GAG	101	88(87%)	1.19	1.14	0.75	0.11
GGA	194	159(82%)	1.57	1.14	0.78	0.13
AAG	77	68(88%)	1.70	1.10	0.82	0.12
GAA	135	120(89%)	1.72	1.22	0.59	0.09
AGA	205	144(%)	1.62	1.15	0.80	0.13
SGG	275	233(85%)	1.69	1.11	0.68	0.12
GSG	110	90(82%)	1.79	1.14	0.81	0.12
GGS	133	116(87%)	1.59	1.16	0.75	0.11
SSG	282	221(78%)	1.47	1.13	0.91	0.14
GSS	116	94(81%)	1.70	1.24	1.06	0.13
SGS	327	281(86%)	1.47	1.15	0.82	0.11

Name	NNP1-w min	M06-2X min	$\Delta E^{(1)}$	$\Delta F^{(1)}$	$\Delta E^{(2)}$	RMSD
SSA	233	198(85%)	1.35	1.11	1.03	0.16
SAA	210	173(82%)	1.66	1.18	0.93	0.13
ASS	102	87(85%)	1.35	1.28	1.18	0.13
AAS	72	60(83%)	1.76	1.25	0.95	0.12
SAS	224	197(88%)	1.89	1.20	0.89	0.11
ASA	71	65(91%)	1.56	1.22	1.11	0.15
ASG	124	101(81%)	1.64	1.18	1.06	0.16
SAG	354	286(81%)	1.73	1.15	0.73	0.11
AGS	193	153(79%)	1.79	1.21	1.00	0.13
GAS	146	122(84%)	1.92	1.29	0.97	0.12
GSA	203	156(77%)	1.70	1.20	1.11	0.14
SGA	441	361(81%)	1.63	1.12	0.86	0.10
AAA	127	111(87%)	1.61	1.18	0.93	0.13
SSS	243	198(81%)	1.43	1.21	1.12	0.14

Table S4: Number of M06-2X minima per cis/trans isomer for 27 methylated tripeptides within the 0–25 kJ/mol range.

Tripeptide	Solvent	Number of M06-2X minima In 0-25kJ/mol CC	CT	TC	TT
GGG	Gas-phase	2	7	2	10
	Water-pcm	4	26	14	40
AGG	Gas-phase	1	7	0	14
	Water-pcm	5	22	35	106
SGG	Gas-phase	2	10	0	5
	Water-pcm	7	78	53	95

Tripeptide	Solvent	Number of M06-2X minima In 0-25kJ/mol CC	CT	TC	TT
GAG	Gas-phase	0	11	5	24
	Water-pcm	0	17	8	63
AAG	Gas-phase	0	8	0	16
	Water-pcm	0	14	7	47
SAG	Gas-phase	0	18	0	14
	Water-pcm	0	44	27	215

Tripeptide	Solvent	Number of M06-2X minima In 0-25kJ/mol CC	CT	TC	TT
GSG	Gas-phase	1	10	0	4
	Water-pcm	11	33	18	28
ASG	Gas-phase	1	7	0	2
	Water-pcm	6	31	8	56
SSG	Gas-phase	1	10	0	5
	Water-pcm	19	88	22	92

Tripeptide	Solvent	Number of M06-2X minima In 0-25kJ/mol CC	CT	TC	TT
GGA	Gas-phase	1	12	3	21
	Water-pcm	2	44	16	97
AGA	Gas-phase	1	10	2	23
	Water-pcm	2	27	19	96
SGA	Gas-phase	2	18	0	13
	Water-pcm	4	129	41	187

Tripeptide	Solvent	Number of M06-2X minima In 0-25kJ/mol CC	CT	TC	TT
GGS	Gas-phase	2	7	4	10
	Water-pcm	19	23	37	37
AGS	Gas-phase	2	7	6	15
	Water-pcm	11	20	59	63
SGS	Gas-phase	4	11	1	7
	Water-pcm	42	54	77	108

Tripeptide	Solvent	Number of M06-2X minima In 0-25kJ/mol CC	CT	TC	TT
GAS	Gas-phase	3	8	8	15
	Water-pcm	9	19	40	54
AAS	Gas-phase	2	2	2	12
	Water-pcm	4	6	28	22
SAS	Gas-phase	5	11	4	17
	Water-pcm	19	31	66	81

Tripeptide	Solvent	Number of M06-2X minima In 0-25kJ/mol CC	CT	TC	TT
GSA	Gas-phase	1	17	0	6
	Water-pcm	9	71	14	62
ASA	Gas-phase	1	10	0	6
	Water-pcm	5	26	8	26
SSA	Gas-phase	1	18	0	7
	Water-pcm	16	86	22	74

Tripeptide	Solvent	Number of M06-2X minima In 0-25kJ/mol CC	CT	TC	TT
GAA	Gas-phase	1	13	5	23
	Water-pcm	0	25	10	85
AAA	Gas-phase	1	8	1	28
	Water-pcm	0	18	11	82
SAA	Gas-phase	1	24	2	32
	Water-pcm	0	42	16	115

Tripeptide	Solvent	Number of M06-2X minima In 0-25kJ/mol CC	CT	TC	TT
GSS	Gas-phase	4	6	1	2
	Water-pcm	22	25	18	29
ASS	Gas-phase	3	6	0	1
	Water-pcm	15	18	19	35
SSS	Gas-phase	4	7	0	0
	Water-pcm	39	58	46	55

Table S5: The probability of N- and O-protonation from HSA calculations at 300 K and the relative proton affinity (PA), with the reference PA set to that of GGG, for C1- and N1-methylation.

AGG	96.4	3.6	15.1	SGG	100.0	0.0	52.4
AAG	91. 4	8.6	18.8	SSG	100.0	0.0	51.7
AGA	86.3	13.7	17.1	SGS	100.0	0.0	50.1
AAA	71.9	28.1	20.9	SSS	100.0	0.0	50.0
ASA	99.7	0.3	27.4	SAS	99.7	0.3	58.9
AAS	78.8	21.2	19.2	SSA	100.0	0.0	67.2
AGS	97.0	3.0	13.1	SGA	100.0	0.0	54.5
ASG	100.0	0.0	26.3	SAG	100.0	0.0	58.8
ASS	99.9	0.1	24.4	SAA	99.9	0.1	61.4

Table S6: The probability of N- and O-protonation from HSA calculations at 300 K and from the experimental work on 2022 (J. Phys. Chem. A 2022, 126, 25, 4036–4045).

**Tripeptides**	**N%<br>HSA**	**N%<br>Exp.**	**O%<br>HSA**	**O%<br>Exp.**
GGG	82.7	35	17.4	65
AGG	96.4	70	3.6	30
GAG	38.9	30	61.1	70
GGA	44.1	x	55.9	x
AAG	91. 4	45	8.6	55
AGA	86.3	15	13.7	85
GAA	16.9	10	83.1	90
AAA	71.9	35	28.1	65

Figure S11: Conformational Population Analysis of 27 Methylated Tripeptides Using HSA and rescaled harmonic spectra of low-energy conformers at M06-2X/6-311+G(d,p).

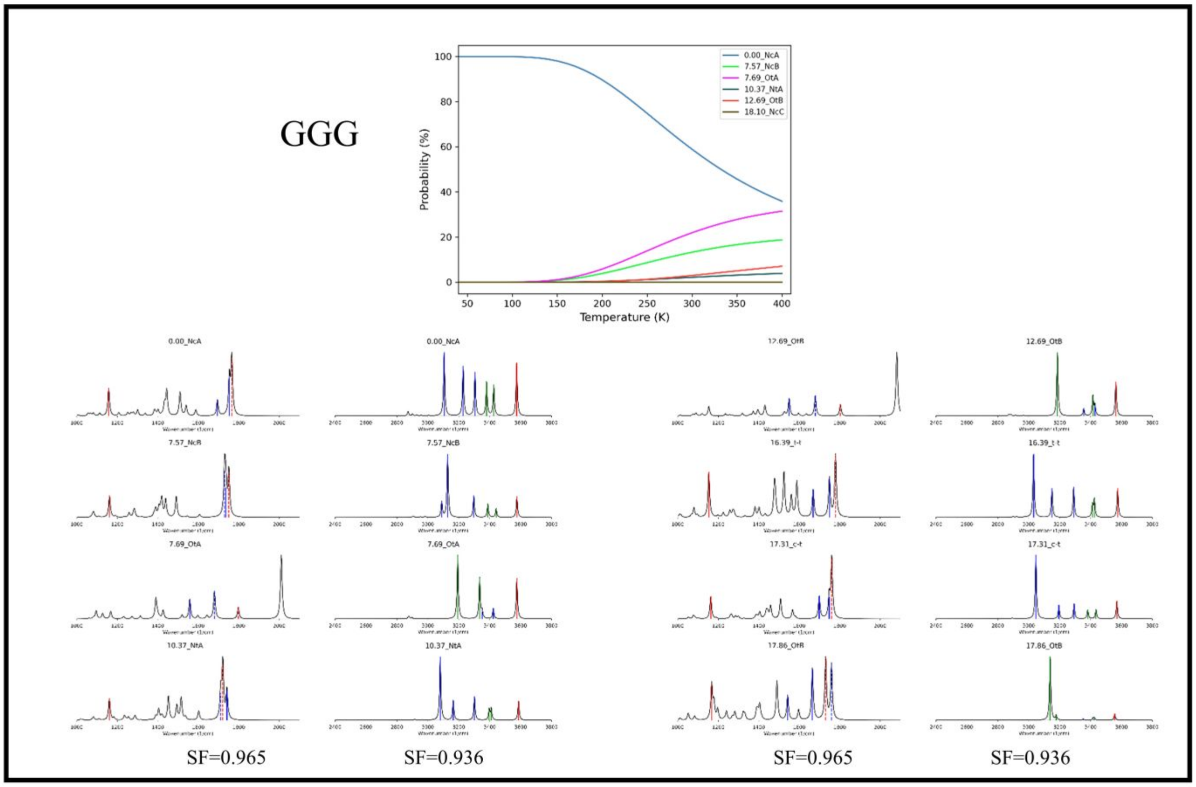

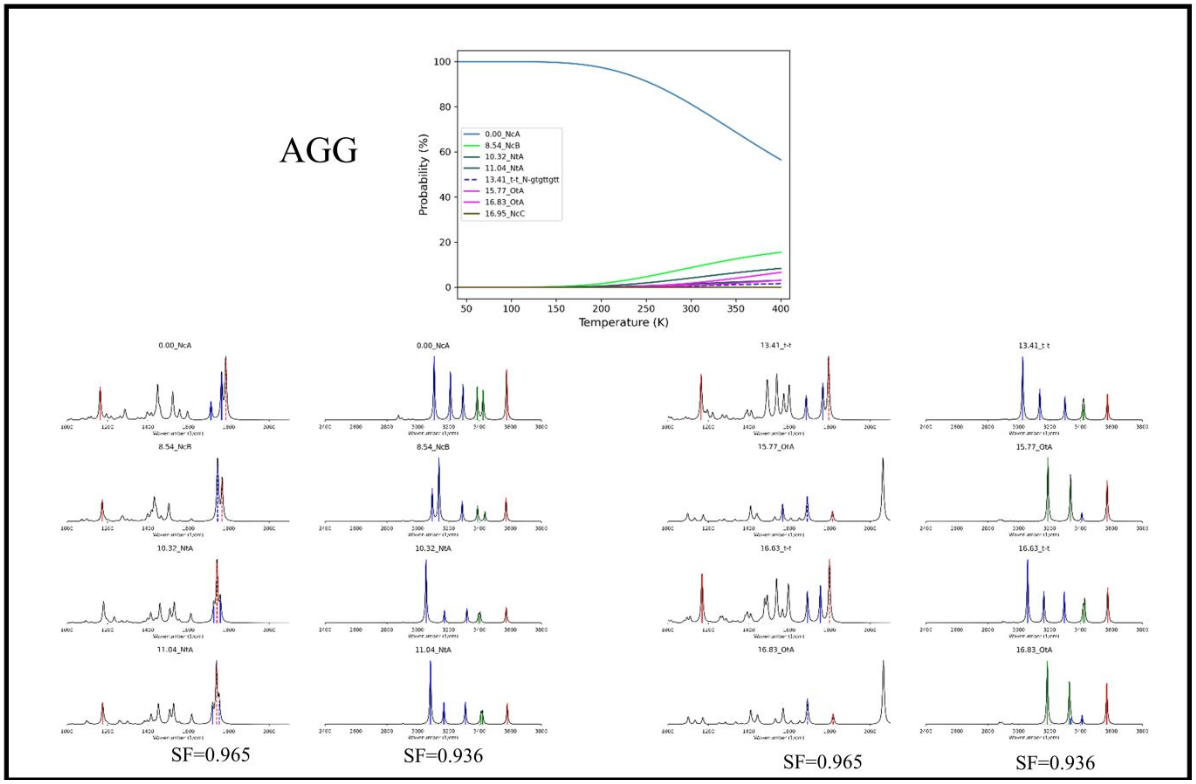

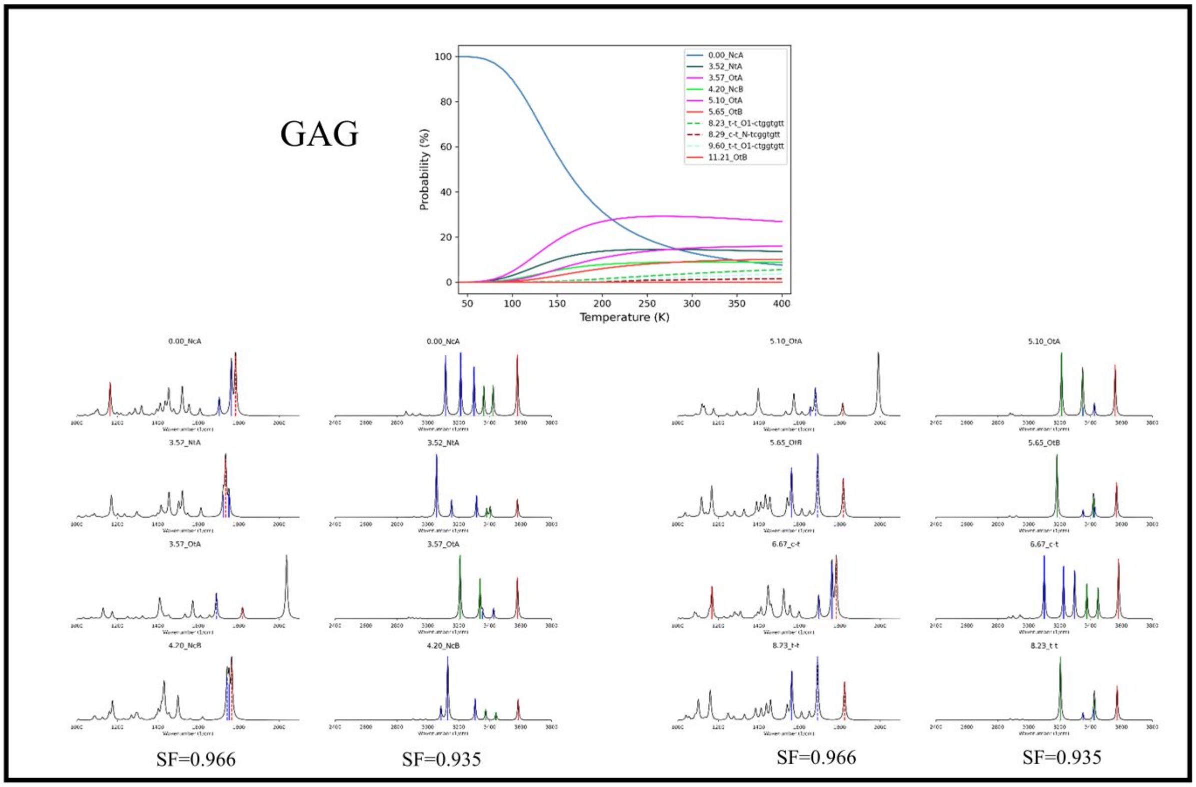
GAG
0.00_NcA
3.52_NtA
3.57_OtA
4.20_NcB
5.10_OtA
5.65_OtB
8.23_t-t_O1-ctggtgtt
8.29_c-t_N-tcggtgtt
9.60_t-t_O1-ctggtgtt
11.21_OtB
Probability (%)
Temperature (K)
SF=0.966
SF=0.935
SF=0.966
SF=0.935

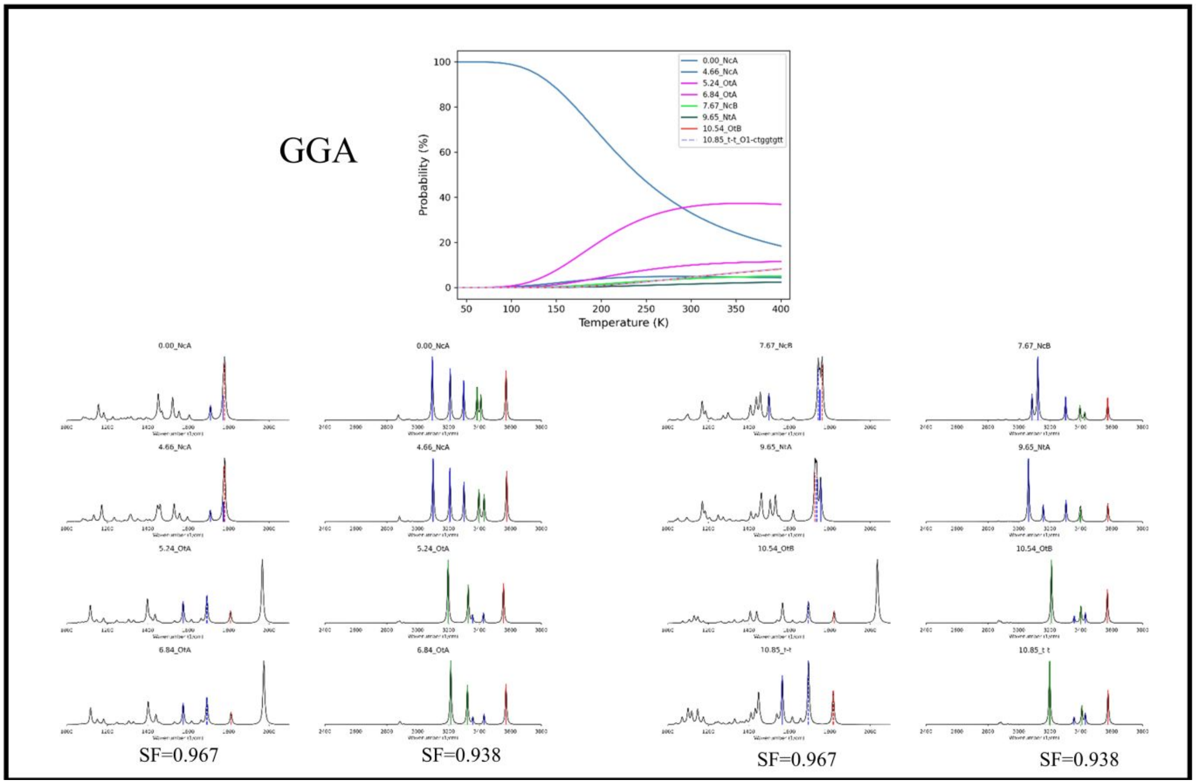
GGA
0.00_NcA
4.66_NcA
5.24_OtA
6.84_OtA
7.67_NcB
9.65_NtA
10.54_OtB
10.85_t-t_O1-ctggtgtt
Probability (%)
Temperature (K)
SF=0.967
SF=0.938
SF=0.967
SF=0.938

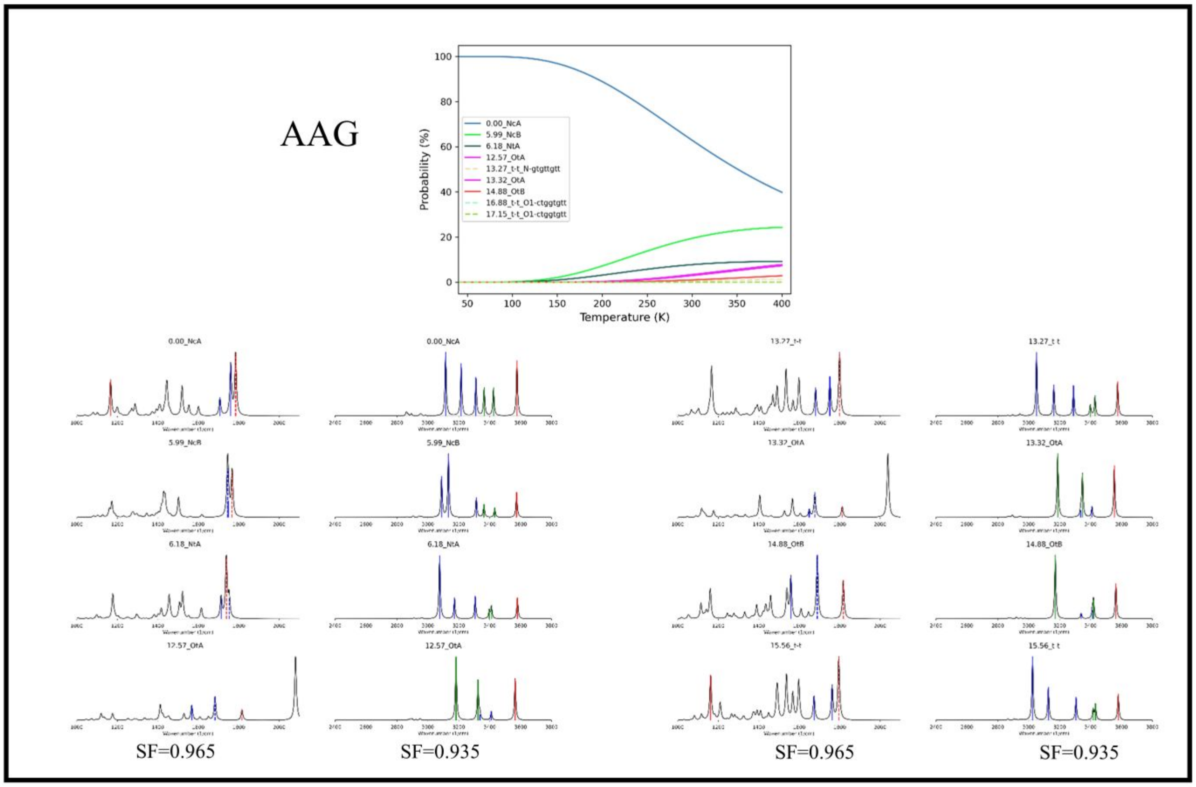
AAG
Probability (%)
Temperature (K)
0.00_NcA
5.99_NcB
6.18_NtA
12.57_OtA
13.27_t-t_N-gtgttgtt
13.32_OtA
14.88_OtB
16.88_t-t_O1-ctggtgtt
17.15_t-t_O1-ctggtgtt
SF=0.965
SF=0.935
SF=0.965
SF=0.935

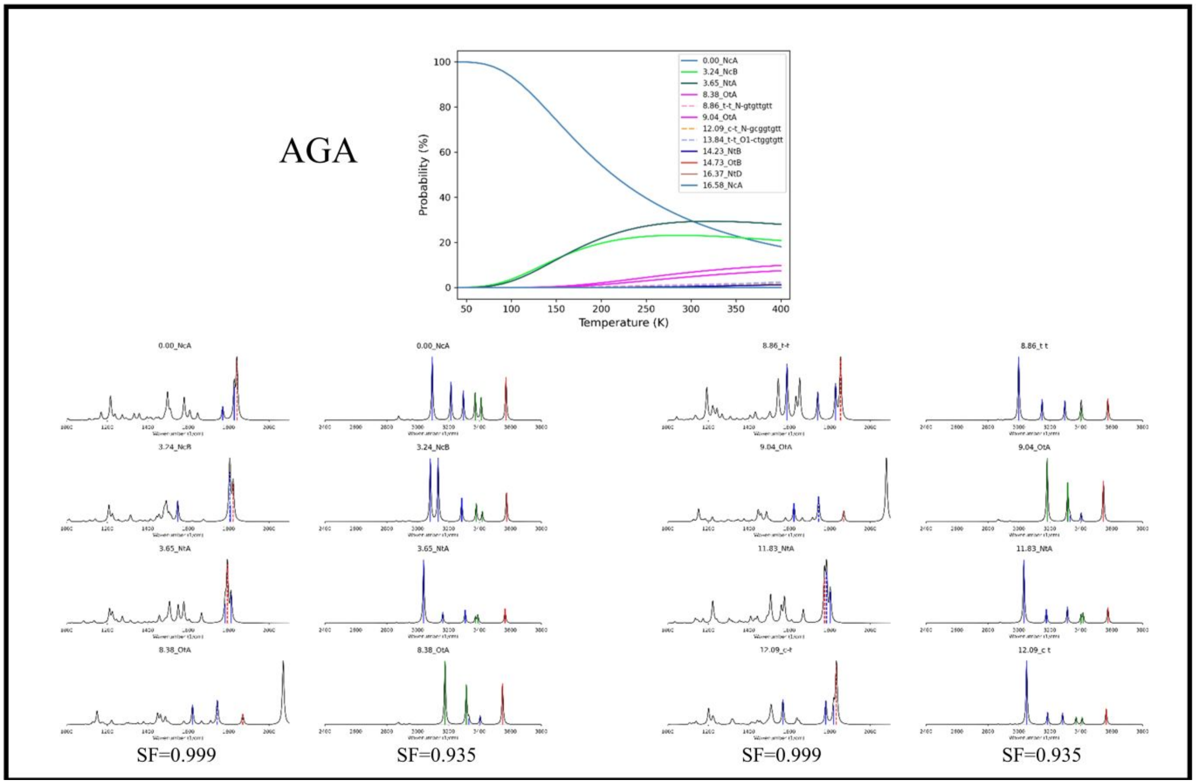
AGA
Probability (%)
Temperature (K)
0.00_NcA
3.24_NcB
3.65_NtA
8.38_OtA
8.86_t-t_N-gtgttgtt
9.04_OtA
12.09_c-t_N-gcggtgtt
13.84_t-t_O1-ctggtgtt
14.23_NtB
14.73_OtB
16.37_NtD
16.58_NcA
SF=0.999
SF=0.935
SF=0.999
SF=0.935

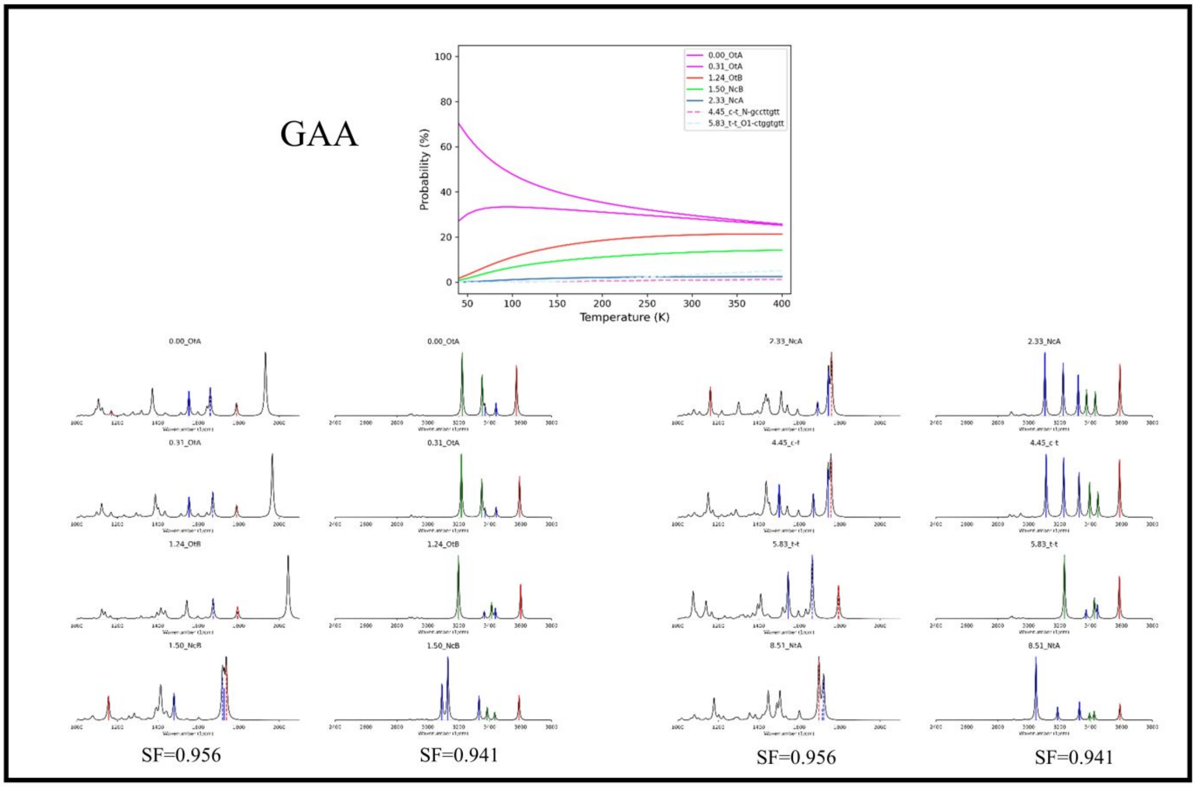
GAA
0.00_OtA
0.31_OtA
1.24_OtB
1.50_NcB
2.33_NcA
4.45_c-t_N-gccttgtt
5.83_t-t_O1-ctggtgtt
Probability (%)
Temperature (K)
4.45_c-t
5.83_t-t
8.51_NtA
SF=0.956
SF=0.941
SF=0.956
SF=0.941

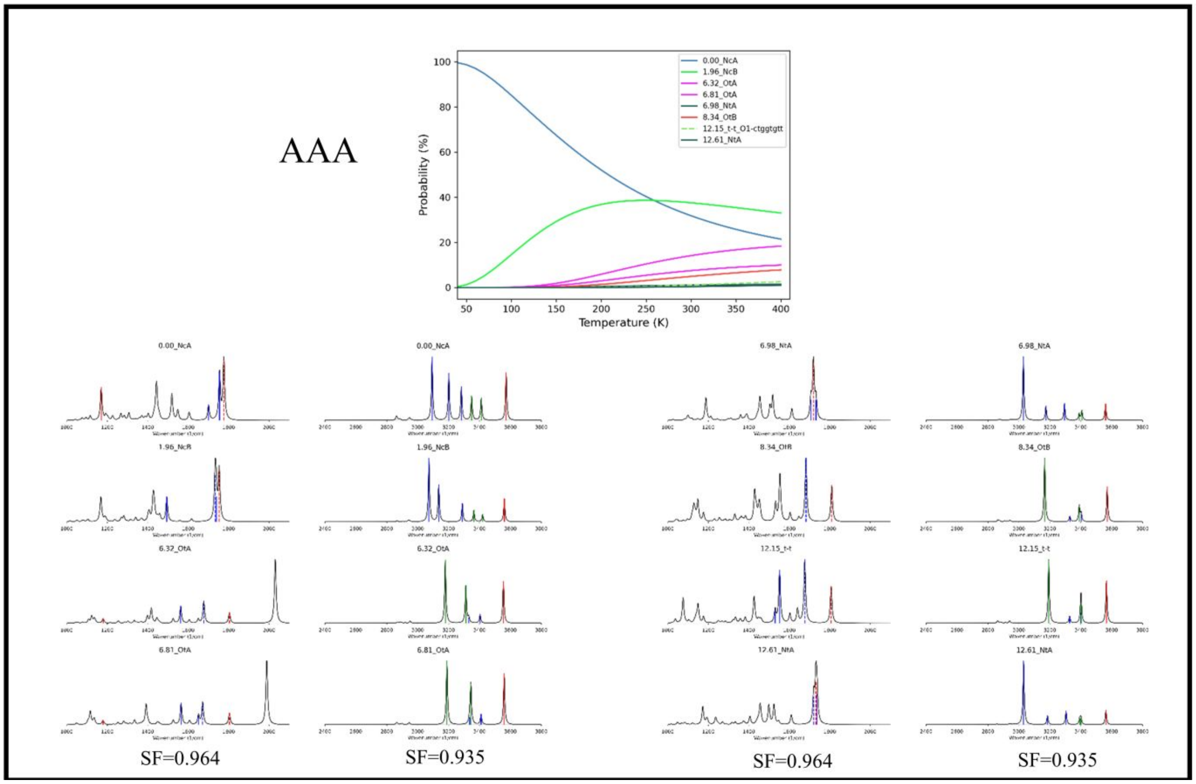
AAA
0.00_NcA
1.96_NcB
6.32_OtA
6.81_OtA
6.98_NtA
8.34_OtB
12.15_t-t_O1-ctggtgtt
12.61_NtA
Probability (%)
Temperature (K)
12.15_t-t
SF=0.964
SF=0.935
SF=0.964
SF=0.935

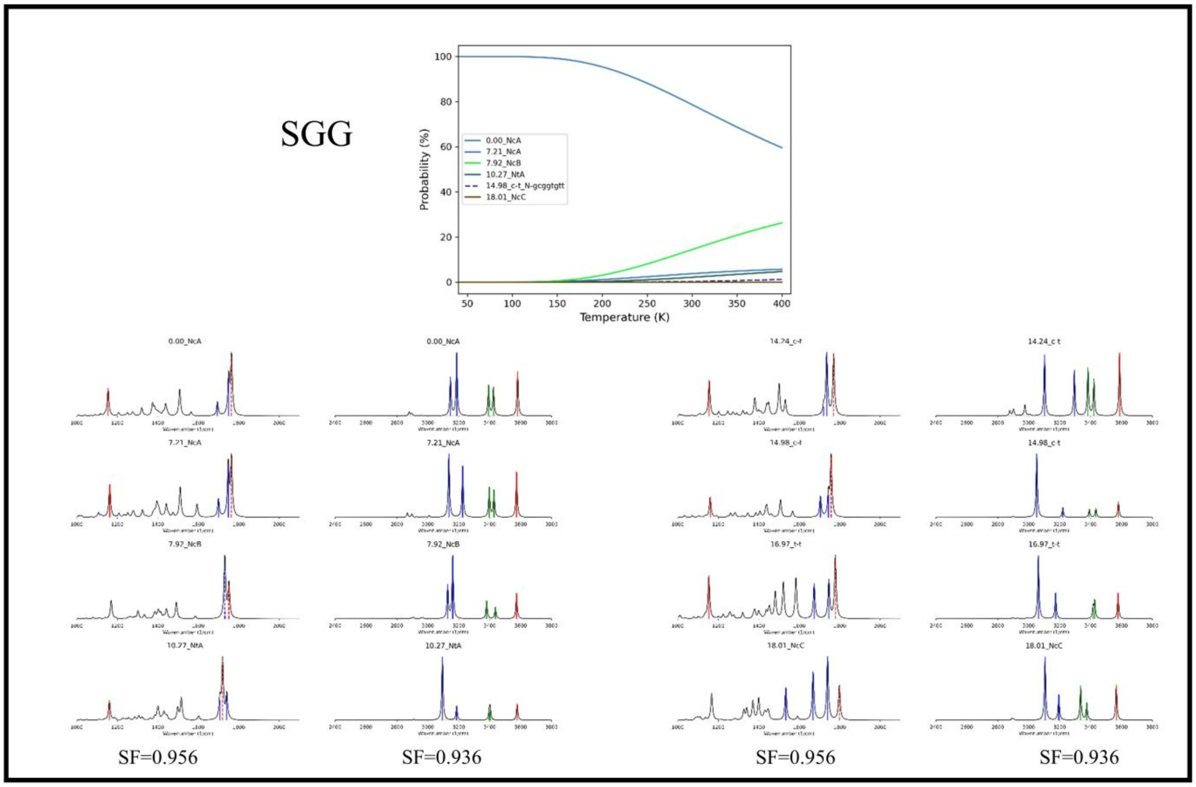
SGG
Probability (%)
Temperature (K)
0.00_NcA
7.21_NcA
7.92_NcB
10.27_NtA
14.98_c-t_N-gcggtgtt
18.01_NcC
0.00_NcA
0.00_NcA
14.24_c-t
14.24_c-t
7.21_NcA
7.21_NcA
14.98_c-t
14.98_c-t
7.92_NcB
7.92_NcB
16.97_t-t
16.97_t-t
10.27_NtA
10.27_NtA
18.01_NcC
18.01_NcC
SF=0.956
SF=0.936
SF=0.956
SF=0.936

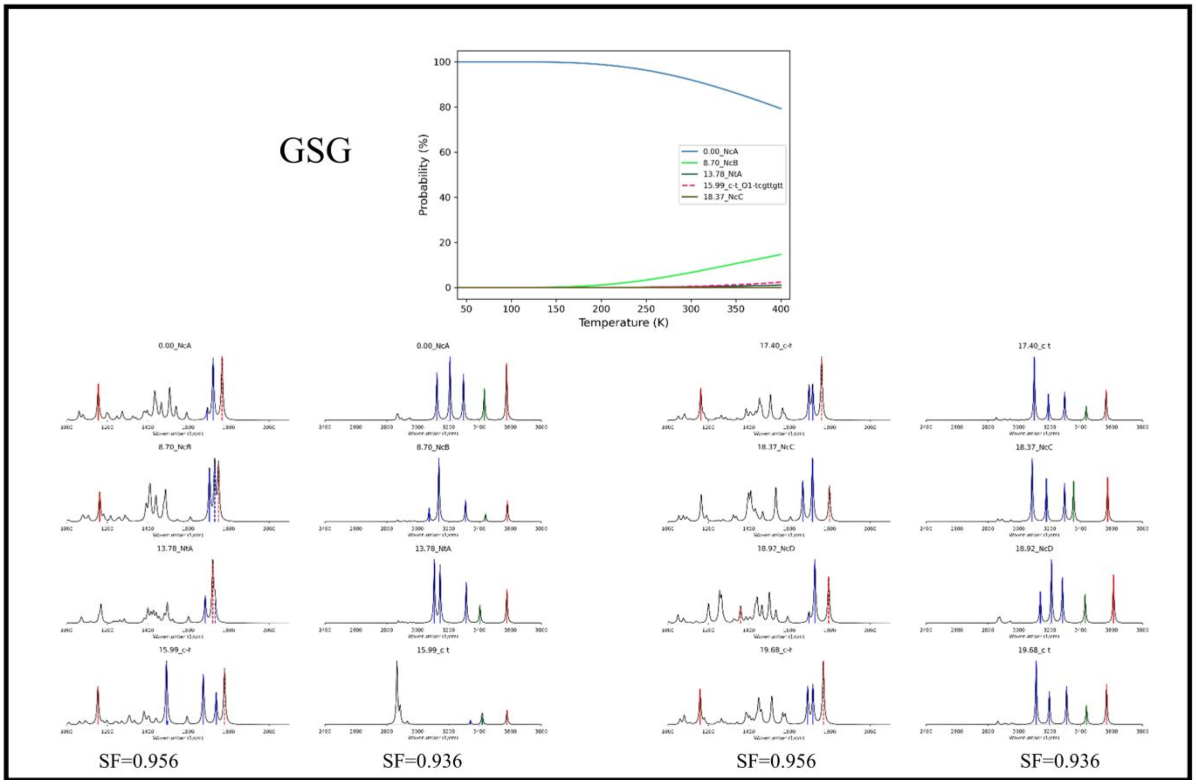
GSG
Probability (%)
Temperature (K)
0.00_NcA
8.70_NcB
13.78_NtA
15.99_c-t_O1-tcgttgtt
18.37_NcC
0.00_NcA
0.00_NcA
17.40_c-t
17.40_c-t
8.70_NcB
8.70_NcB
18.37_NcC
18.37_NcC
13.78_NtA
13.78_NtA
18.92_NcD
18.92_NcD
15.99_c-t
15.99_c-t
19.68_c-t
19.68_c-t
SF=0.956
SF=0.936
SF=0.956
SF=0.936

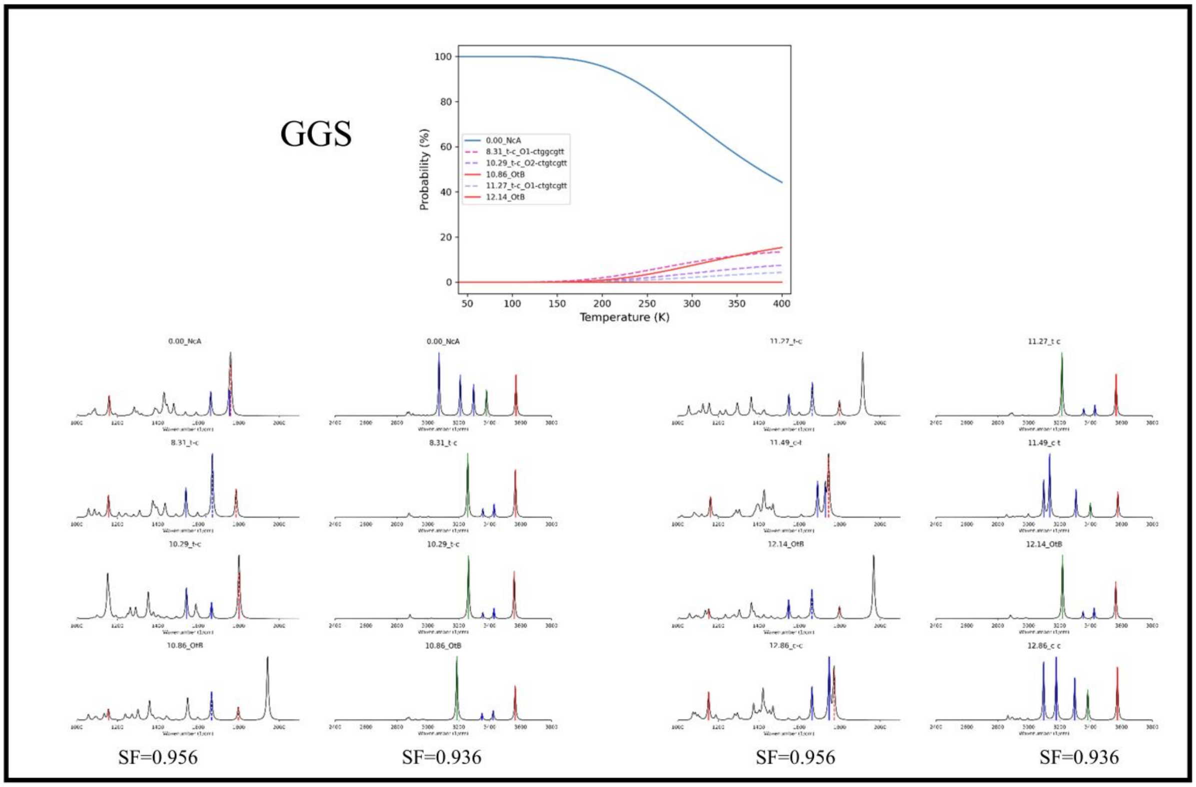
GGS
Probability (%)
Temperature (K)
0.00_NcA
8.31_t-c_O1-ctggcgtt
10.29_t-c_O2-ctgtcgtt
10.86_OtB
11.27_t-c_O1-ctgtcgtt
12.14_OtB
SF=0.956
SF=0.936
SF=0.956
SF=0.936

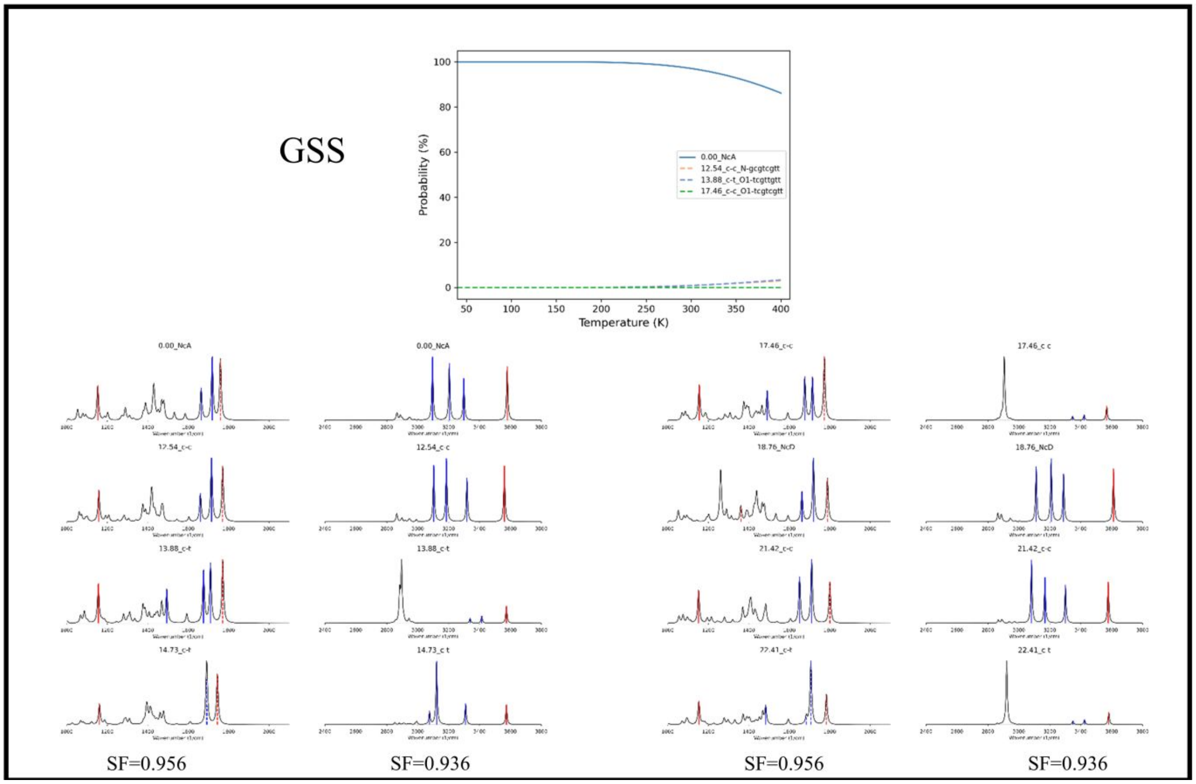
GSS
Probability (%)
Temperature (K)
0.00_NcA
12.54_c-c_N-gcgtcgtt
13.88_c-t_O1-tcgttgtt
17.46_c-c_O1-tcgtcgtt
SF=0.956
SF=0.936
SF=0.956
SF=0.936

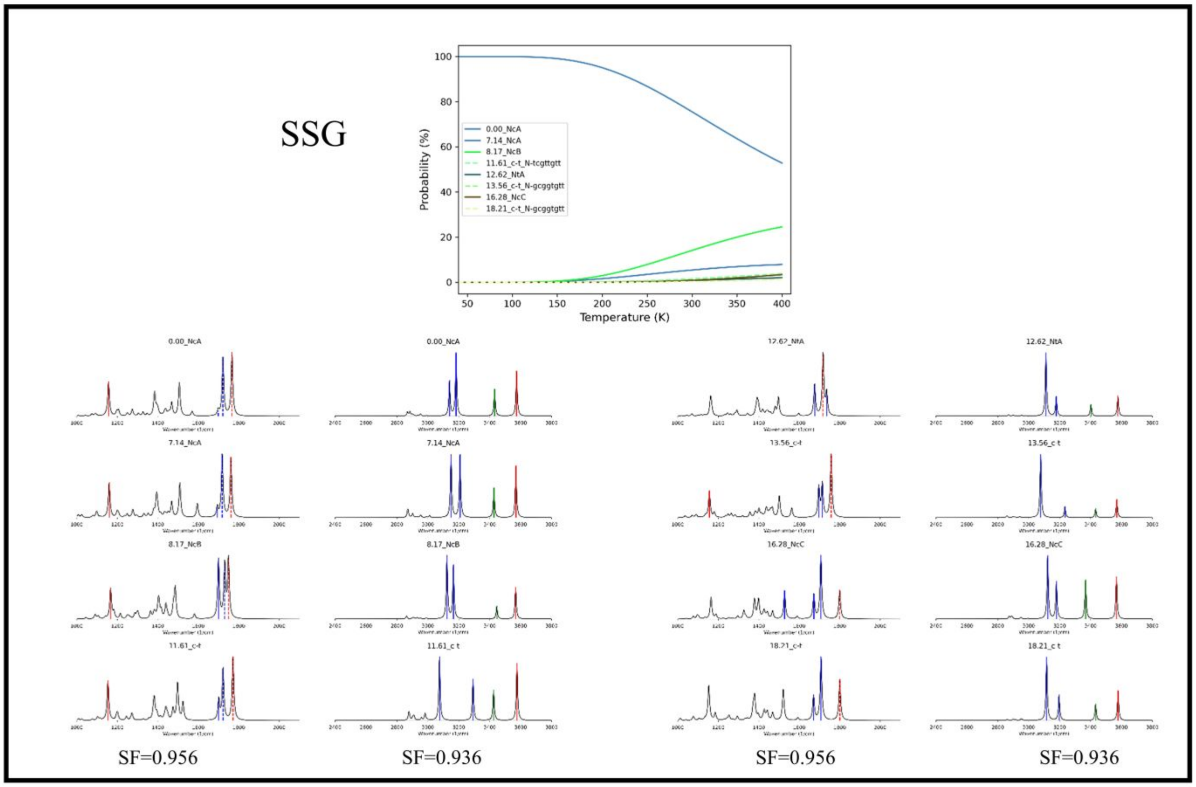
SSG
Probability (%)
Temperature (K)
0.00_NcA
7.14_NcA
8.17_NcB
11.61_c-t_N-tcgttgtt
12.62_NtA
13.56_c-t_N-gcggtgtt
16.28_NcC
18.21_c-t_N-gcggtgtt
SF=0.956
SF=0.936
SF=0.956
SF=0.936

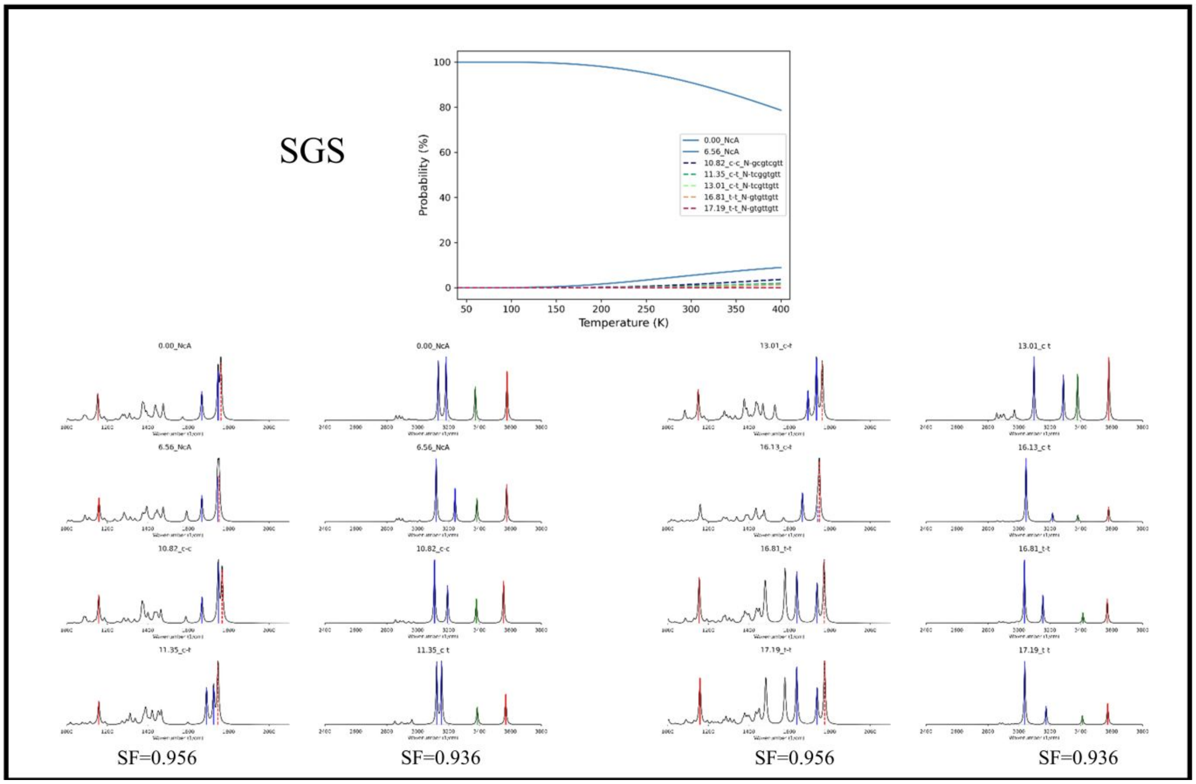
SGS
Probability (%)
Temperature (K)
0.00_NcA
6.56_NcA
10.82_c-c_N-gcgtcgtt
11.35_c-t_N-tcggtgtt
13.01_c-t_N-tcgttgtt
16.81_t-t_N-gtgttgtt
17.19_t-t_N-gtgttgtt
SF=0.956
SF=0.936
SF=0.956
SF=0.936

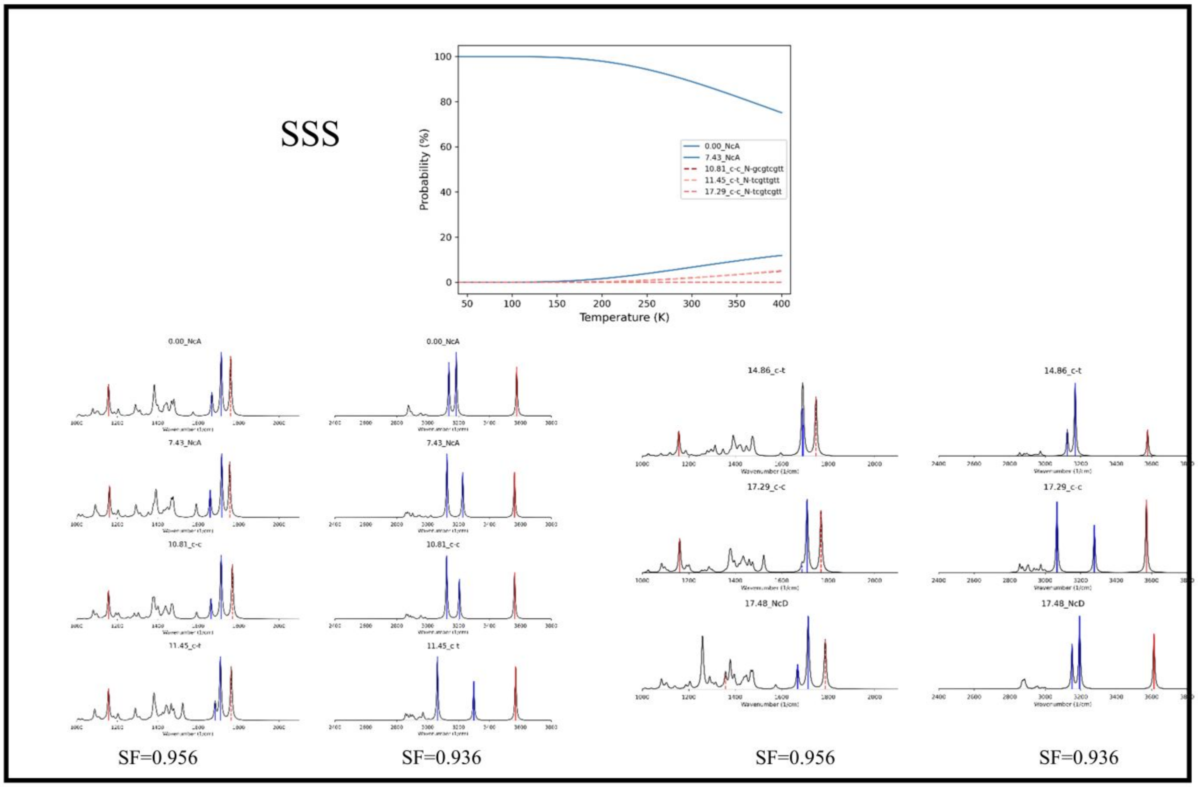
SSS
Probability (%)
Temperature (K)
0.00_NcA
7.43_NcA
10.81_c-c_N-gcgtcgtt
11.45_c-t_N-tcgttgtt
17.29_c-c_N-tcgtcgtt
0.00_NcA
7.43_NcA
10.81_c-c
11.45_c-t
14.86_c-t
17.29_c-c
17.48_NcD
Wavenumber (1/cm)
SF=0.956
SF=0.936
SF=0.956
SF=0.936

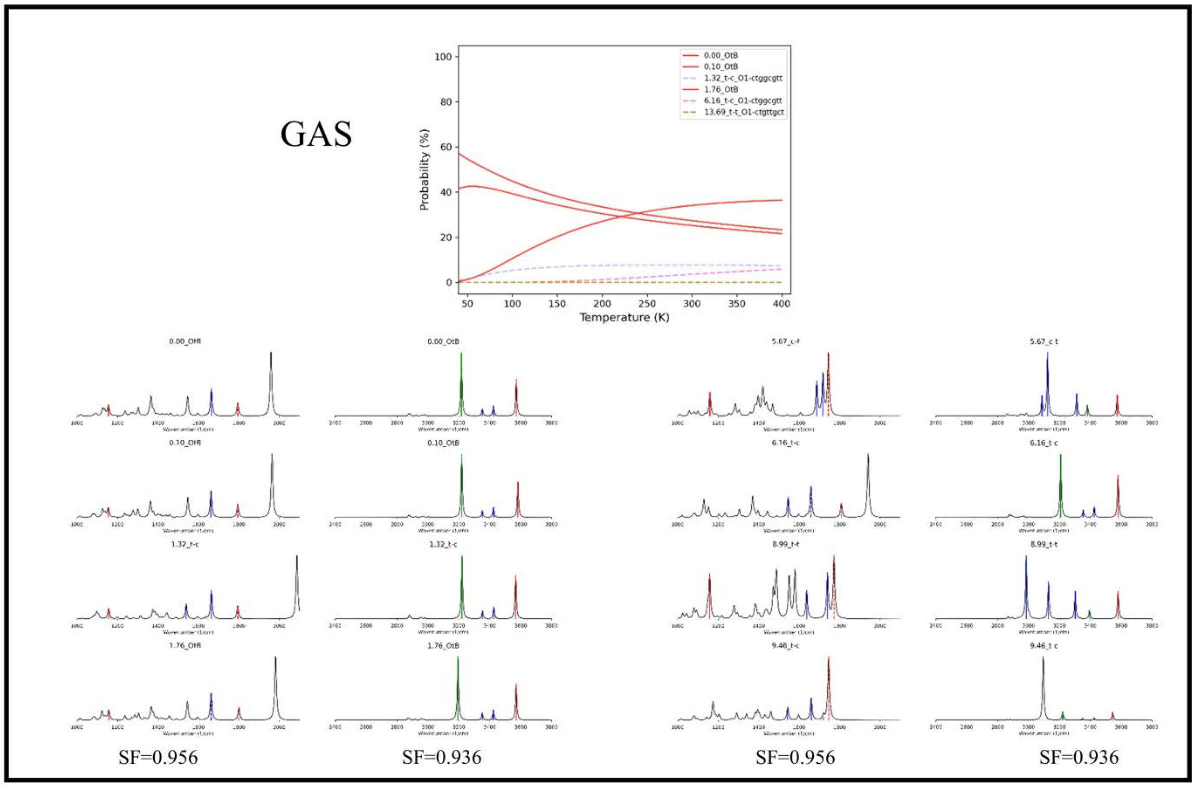
GAS
0.00_OtB
0.10_OtB
1.32_t-c_O1-ctggcgtt
1.76_OtB
6.16_t-c_O1-ctggcgtt
13.69_t-t_O1-ctgttgct
Probability (%)
Temperature (K)
SF=0.956
SF=0.936
SF=0.956
SF=0.936

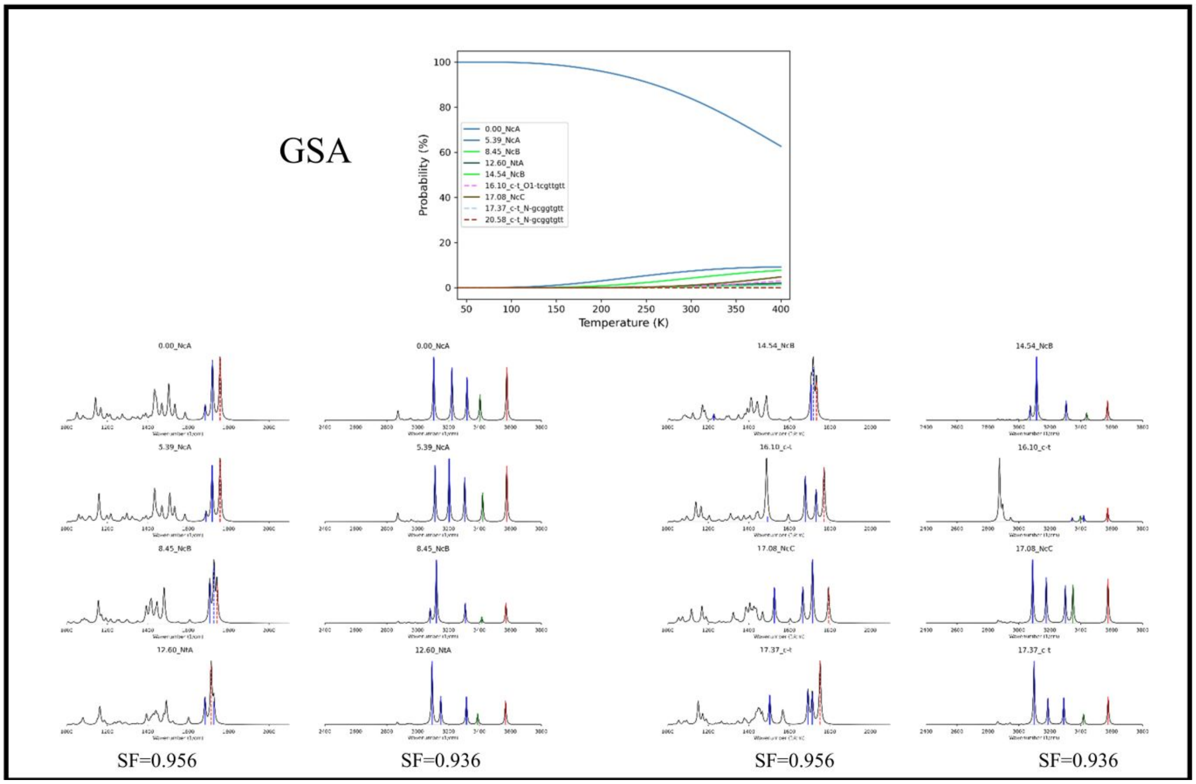
GSA
0.00_NcA
5.39_NcA
8.45_NcB
12.60_NtA
14.54_NcB
16.10_c-t_O1-tcgttgtt
17.08_NcC
17.37_c-t_N-gcggtgtt
20.58_c-t_N-gcggtgtt
Probability (%)
Temperature (K)
SF=0.956
SF=0.936
SF=0.956
SF=0.936

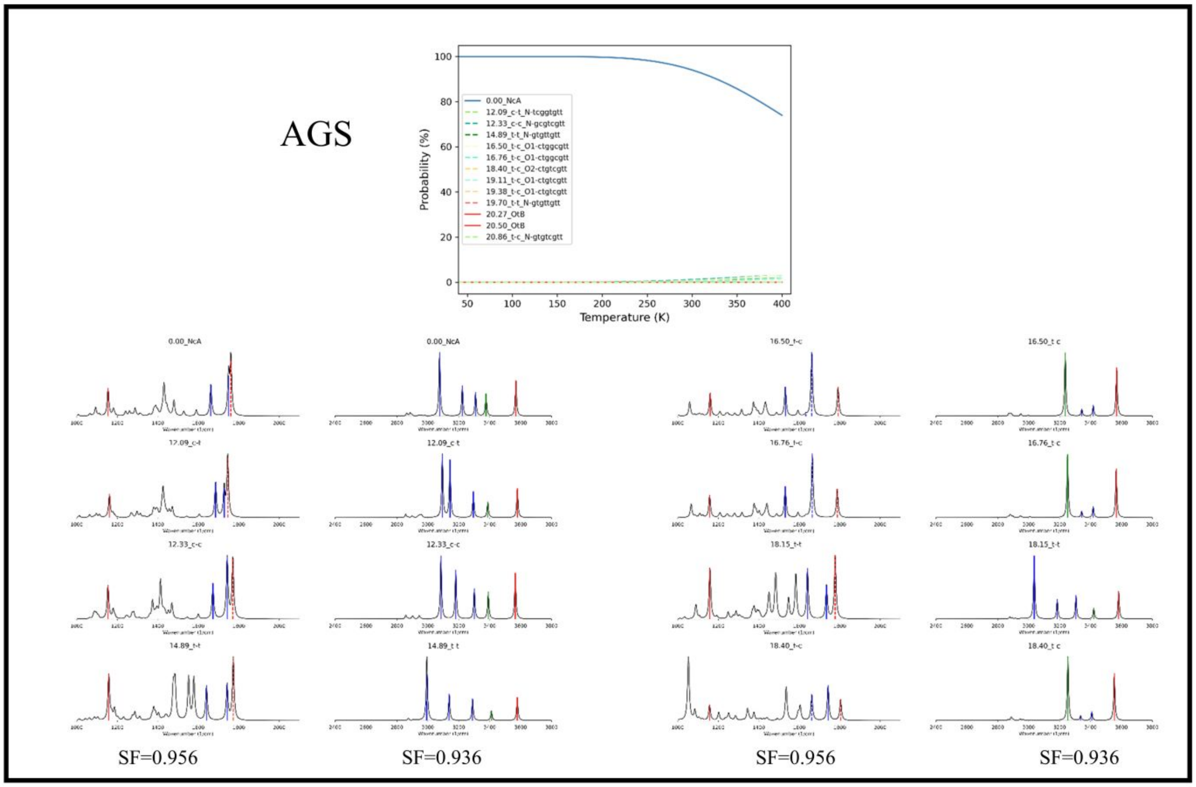

AGS
Probability (%)
Temperature (K)
0.00_NcA
12.09_c-t_N-tcggtgtt
12.33_c-c_N-gcgtcgtt
14.89_t-t_N-gtgttgtt
16.50_t-c_O1-ctggcgtt
16.76_t-c_O1-ctggcgtt
18.40_t-c_O2-ctgtcgtt
19.11_t-c_O1-ctgtcgtt
19.38_t-c_O1-ctgtcgtt
19.70_t-t_N-gtgttgtt
20.27_OtB
20.50_OtB
20.86_t-c_N-gtgtcgtt
0.00_NcA
12.09_c-t
12.33_c-c
14.89_t-t
16.50_t-c
16.76_t-c
18.15_t-t
18.40_t-c
SF=0.956
SF=0.936
SF=0.956
SF=0.936

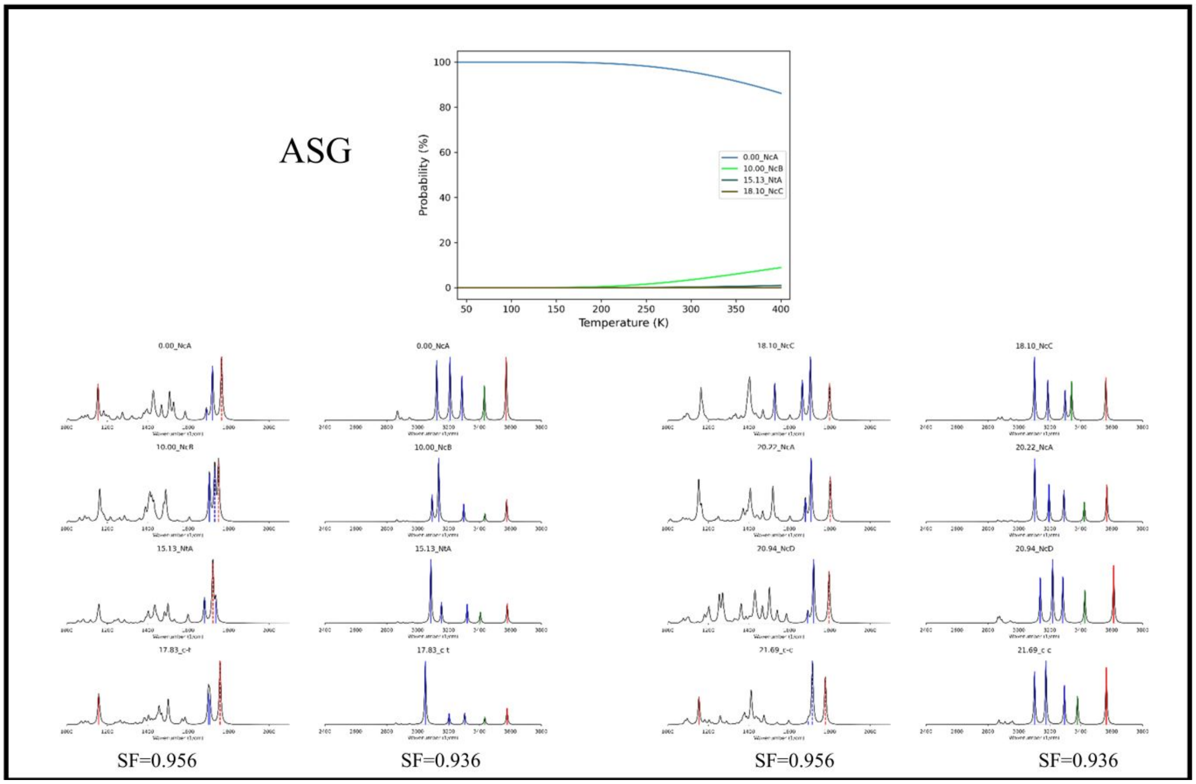

ASG
Probability (%)
Temperature (K)
0.00_NcA
10.00_NcB
15.13_NtA
18.10_NcC
0.00_NcA
10.00_NcB
15.13_NtA
17.83_c-t
18.10_NcC
20.22_NcA
20.94_NcD
21.69_c-c
SF=0.956
SF=0.936
SF=0.956
SF=0.936

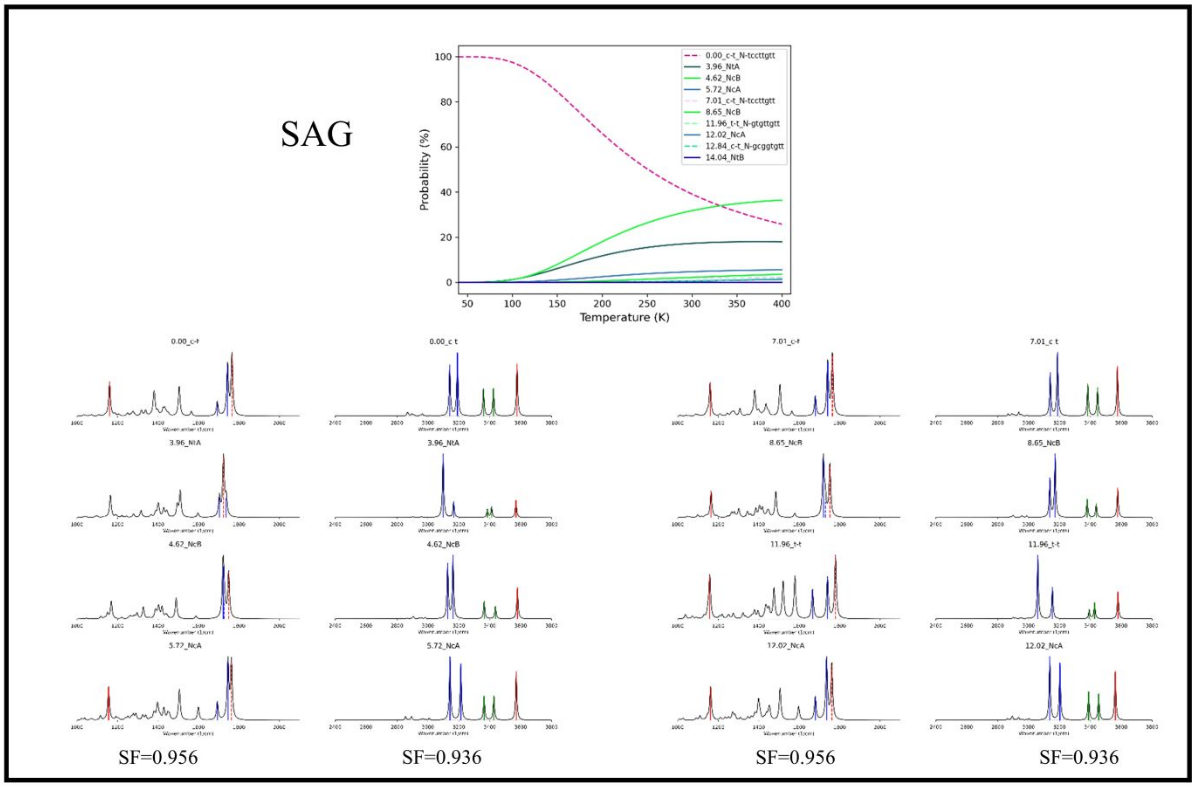
SAG
Probability (%)
Temperature (K)
0.00_c-t_N-tccttgtt
3.96_NtA
4.62_NcB
5.72_NcA
7.01_c-t_N-tccttgtt
8.65_NcB
11.96_t-t_N-gtgttgtt
12.02_NcA
12.84_c-t_N-gcggtgtt
14.04_NtB
SF=0.956
SF=0.936
SF=0.956
SF=0.936

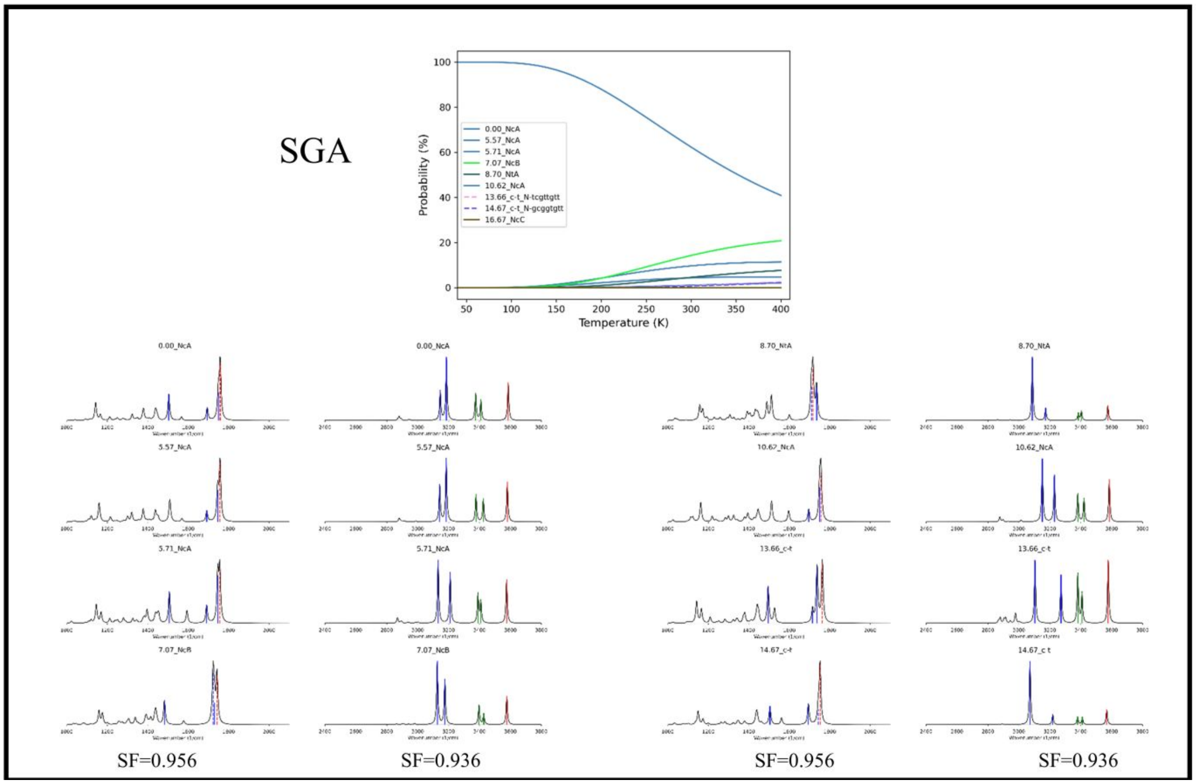
SGA
Probability (%)
Temperature (K)
0.00_NcA
5.57_NcA
5.71_NcA
7.07_NcB
8.70_NtA
10.62_NcA
13.66_c-t_N-tcgttgtt
14.67_c-t_N-gcggtgtt
16.67_NcC
SF=0.956
SF=0.936
SF=0.956
SF=0.936

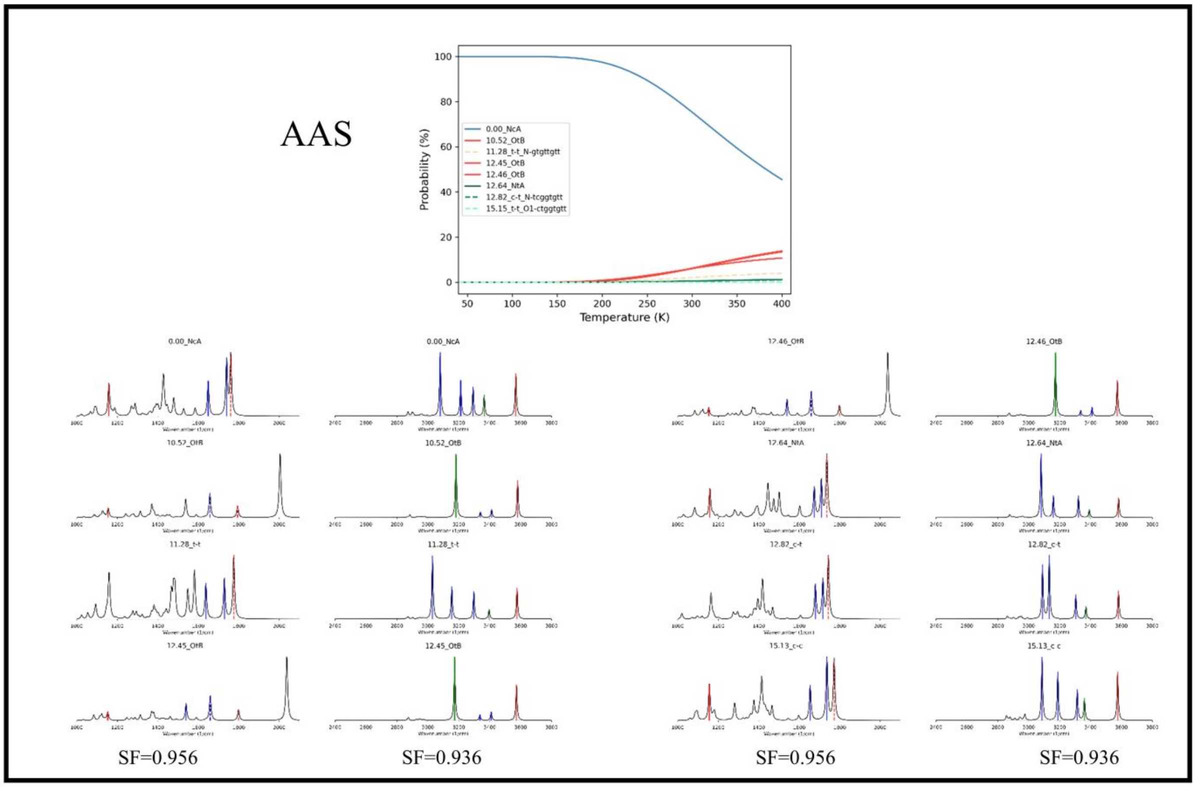
AAS
Probability (%)
Temperature (K)
0.00_NcA
10.52_OtB
11.28_t-t_N-gtgttgtt
12.45_OtB
12.46_OtB
12.64_NtA
12.82_c-t_N-tcggtgtt
15.15_t-t_O1-ctggtgtt
0.00_NcA
10.52_OtB
11.28_t-t
12.45_OtB
12.46_OtB
12.64_NtA
12.82_c-t
15.13_c-c
SF=0.956
SF=0.936
SF=0.956
SF=0.936

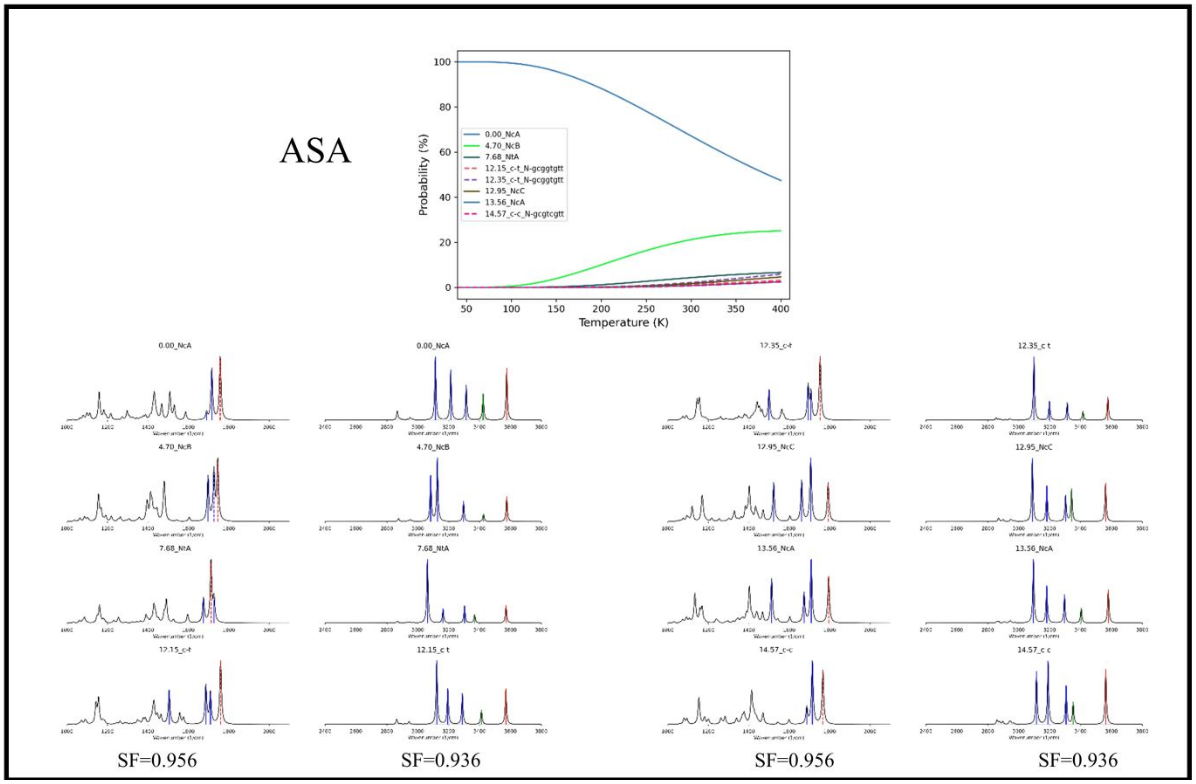
ASA
Probability (%)
Temperature (K)
0.00_NcA
4.70_NcB
7.68_NtA
12.15_c-t_N-gcggtgtt
12.35_c-t_N-gcggtgtt
12.95_NcC
13.56_NcA
14.57_c-c_N-gcgtcgtt
0.00_NcA
4.70_NcB
7.68_NtA
12.15_c-t
12.35_c-t
12.95_NcC
13.56_NcA
14.57_c-c
SF=0.956
SF=0.936
SF=0.956
SF=0.936

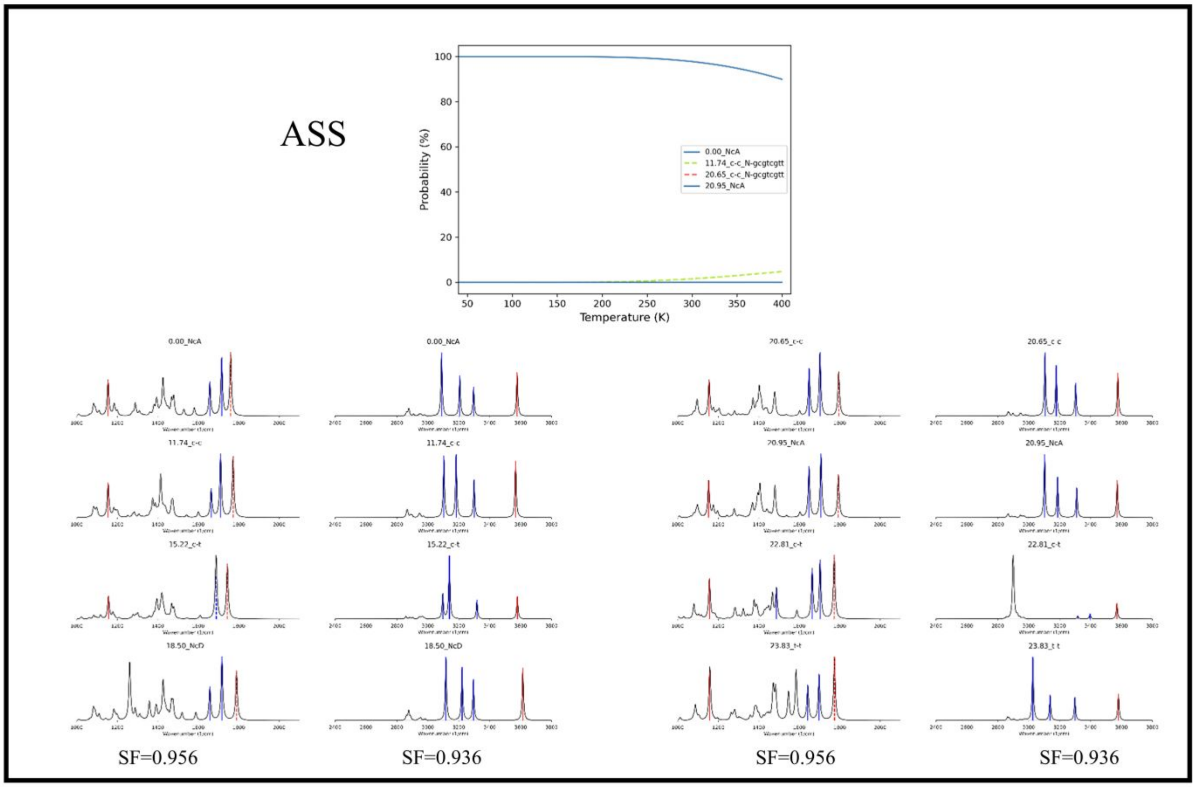
ASS
Probability (%)
Temperature (K)
0.00_NcA
11.74_c-c_N-gcgtcgtt
20.65_c-c_N-gcgtcgtt
20.95_NcA
0.00_NcA
11.74_c-c
15.22_c-t
18.50_NcD
20.65_c-c
20.95_NcA
22.81_c-t
23.83_t-t
SF=0.956
SF=0.936
SF=0.956
SF=0.936

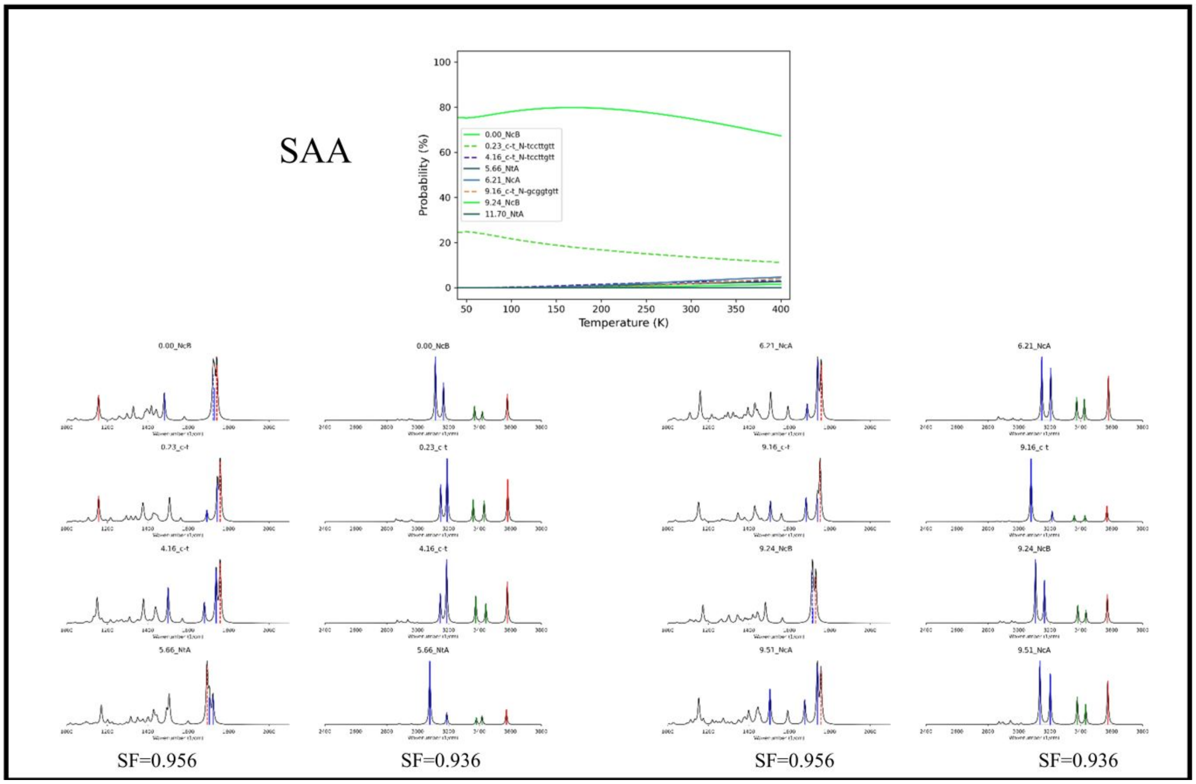
SAA
Probability (%)
Temperature (K)
0.00_NcB
0.23_c-t_N-tccttgtt
4.16_c-t_N-tccttgtt
5.66_NtA
6.21_NcA
9.16_c-t_N-gcggtgtt
9.24_NcB
11.70_NtA
0.00_NcB
0.23_c-t
4.16_c-t
5.66_NtA
6.21_NcA
9.16_c-t
9.24_NcB
9.51_NcA
SF=0.956
SF=0.936
SF=0.956
SF=0.936

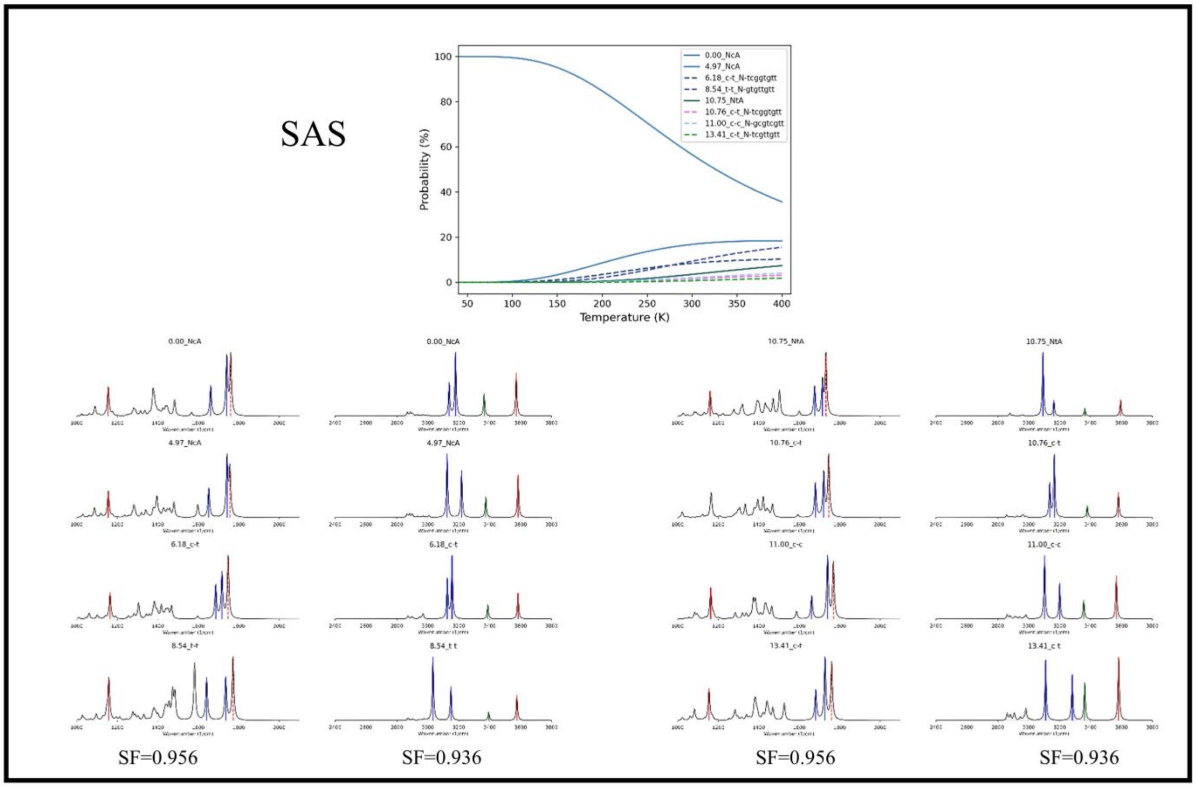
SAS
0.00_NcA
4.97_NcA
6.18_c-t_N-tcggtgtt
8.54_t-t_N-gtgttgtt
10.75_NtA
10.76_c-t_N-tcggtgtt
11.00_c-c_N-gcgtcgtt
13.41_c-t_N-tcgttgtt
Probability (%)
Temperature (K)
0.00_NcA
4.97_NcA
6.18_c-t
8.54_t-t
10.75_NtA
10.76_c-t
11.00_c-c
13.41_c-t
SF=0.956
SF=0.936
SF=0.956
SF=0.936

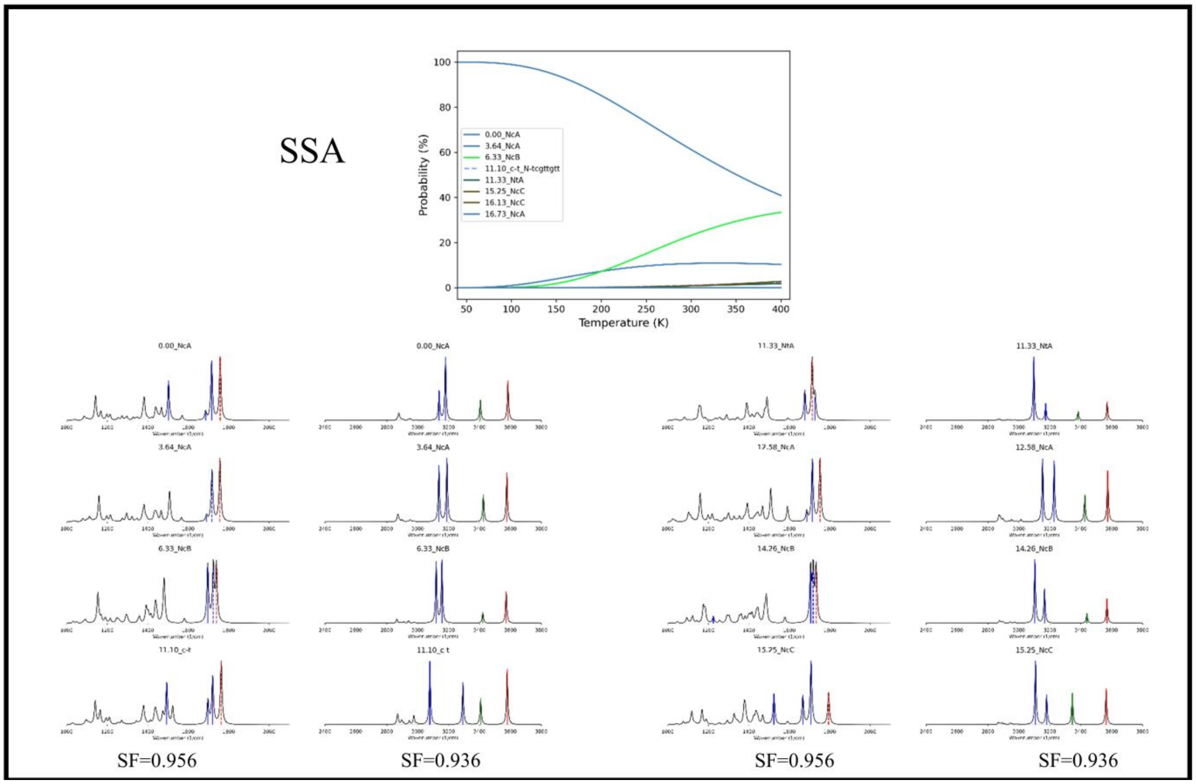
SSA
0.00_NcA
3.64_NcA
6.33_NcB
11.10_c-t_N-tcgttgtt
11.33_NtA
15.25_NcC
16.13_NcC
16.73_NcA
Probability (%)
Temperature (K)
0.00_NcA
3.64_NcA
6.33_NcB
11.10_c-t
11.33_NtA
12.58_NcA
14.26_NcB
15.25_NcC
SF=0.956
SF=0.936
SF=0.956
SF=0.936

Coordinate of M06-2X minima of 27 methylated tripeptide

```
AAA_0.00_NcA.xyz
34
0 eng= -818.705157126 zpe= -818.411451
N       -1.805490       -3.702287       -2.709279
C       -1.359414       -2.281522       -2.912848
C       -2.013725       -1.535490       -1.736771
N       -1.227608       -0.795928       -0.917977
C       0.198409        -0.993946       -0.722960
C       0.522396        -2.496163       -0.605710
N       1.813378        -2.819230       -0.814643
C       2.294189        -4.192651       -0.818359
C       1.622766        -4.988906       -1.935471
O       -3.215642       -1.635157       -1.623281
H       -1.479569       -4.309024       -3.464124
H       -2.829514       -3.714974       -2.633737
H       -1.741882       -0.382287       -0.148283
H       0.748600        -0.589965       -1.580421
O       -0.343966       -3.327374       -0.374581
H       2.466824        -2.085203       -1.044043
H       3.353432        -4.154855       -1.087405
O       2.084673        -6.231620       -1.962573
H       1.678572        -6.714847       -2.696519
O       0.799857        -4.565923       -2.711328
H       -1.388604       -4.037984       -1.824532
C       0.635453        -0.256981       0.544017
H       0.123056        -0.671227       1.415123
H       0.394941        0.803319        0.457430
H       1.709964        -0.351697       0.699549
C       2.164629        -4.887032       0.542550
H       2.610183        -5.879547       0.493146
H       1.119086        -4.973827       0.833945
H       2.696216        -4.300157       1.291458
H       -0.271825       -2.309325       -2.894112
C       -1.878271       -1.752410       -4.239683
H       -1.473766       -2.327995       -5.074490
H       -1.564741       -0.714867       -4.361872
H       -2.969024       -1.780046       -4.261516
```

```
AAA_1.96_NcB.xyz
34
0 eng= -818.704260932 zpe= -818.410705
C       -2.337124       -0.038301       0.907137
H       -1.456794       -0.495797       1.358281
C       -2.371447       -0.130107       -0.630165
O       -2.856710       0.799106        -1.246525
N       -2.246678       1.432870        1.179699
H       -2.774434       1.911560        0.431863
H       -1.251215       1.710109        1.127477
```

N	-1.926173	-1.270548	-1.194059
H	-2.078689	-1.343015	-2.193754
C	-1.095093	-2.272734	-0.527477
H	-1.619078	-2.679649	0.340957
C	0.191156	-1.647630	0.030384
O	0.589623	-1.923012	1.142504
N	0.838138	-0.768237	-0.777700
H	0.423971	-0.477021	-1.650996
C	1.998237	-0.064298	-0.282362
H	2.711332	-0.785746	0.123076
C	1.604641	0.851124	0.869796
O	2.652978	1.195032	1.591765
H	2.386157	1.796664	2.301994
O	0.482746	1.258100	1.086890
H	-2.623243	1.680313	2.095377
C	-0.774768	-3.405776	-1.496374
H	-0.145825	-4.143966	-0.999190
H	-0.238855	-3.031703	-2.372162
H	-1.692995	-3.895720	-1.823057
C	-3.629473	-0.609832	1.480550
H	-3.702976	-1.670067	1.236229
H	-4.497578	-0.102760	1.053267
H	-3.647427	-0.517178	2.568026
C	2.650858	0.759946	-1.391871
H	2.967820	0.098978	-2.198825
H	3.527227	1.280643	-1.007903
H	1.951250	1.498872	-1.790701

AAA_6.32_OtA.xyz
34
0 eng= -818.699463184 zpe= -818.409042

N	-0.677208	1.570172	-4.164831
C	-1.213049	0.224394	-4.324278
C	-0.406339	-0.745959	-3.478756
N	0.676115	-0.291660	-2.889259
C	1.486359	-0.932963	-1.839370
C	1.972059	-2.317776	-2.256039
N	3.232372	-2.617831	-2.004436
C	3.783948	-3.953372	-2.224346
C	5.077455	-4.001441	-1.427508
O	-0.811939	-1.948957	-3.411228
H	-1.296792	2.175924	-3.640642
H	-0.497307	2.010400	-5.059335
H	0.804762	0.707914	-3.077085
H	2.348398	-0.280056	-1.698016
O	1.179451	-3.147176	-2.739091
H	3.861576	-1.949149	-1.569172
H	3.088804	-4.691280	-1.814606
O	5.615053	-5.213220	-1.447083
H	6.445908	-5.201250	-0.949092

O	5.549230	-3.045470	-0.874226
H	-0.028526	-2.591237	-3.055878
C	0.697877	-1.049062	-0.529533
H	1.325879	-1.492803	0.244601
H	-0.184560	-1.677413	-0.662794
H	0.384748	-0.058808	-0.198666
C	4.034201	-4.242907	-3.706246
H	4.446569	-5.245250	-3.817743
H	4.739563	-3.521166	-4.122162
H	3.092643	-4.186971	-4.251226
H	-2.238275	0.140941	-3.947402
C	-1.180622	-0.251568	-5.779618
H	-0.167234	-0.171140	-6.181675
H	-1.845986	0.372133	-6.376846
H	-1.510308	-1.286674	-5.857169

AAA_6.81_OtA.xyz
34
0 eng= -818.699209133 zpe= -818.408859

C	-1.748813	0.962657	-3.907584
H	-1.597778	2.003322	-4.211631
C	-1.359512	0.912478	-2.442106
O	-1.908678	1.734733	-1.644747
N	-0.910247	0.041804	-4.663830
H	-0.242719	0.522796	-5.254395
H	-1.461476	-0.569626	-5.254377
N	-0.481910	0.009532	-2.064193
H	-0.135254	-0.544416	-2.853240
C	-0.192808	-0.422957	-0.685138
H	-1.152545	-0.670183	-0.214286
C	0.346736	0.752744	0.135781
O	-0.301889	1.816869	0.158545
N	1.452725	0.596407	0.834006
H	2.006465	-0.252882	0.774733
C	2.009782	1.654238	1.674831
H	2.015908	2.588020	1.106198
C	3.444821	1.240611	1.956364
O	4.102890	2.185575	2.613423
H	5.003827	1.879207	2.795639
O	3.901592	0.177187	1.634774
H	-1.318595	1.822717	-0.744359
C	0.688026	-1.661380	-0.731040
H	0.838499	-2.062630	0.271637
H	1.659514	-1.445498	-1.182203
H	0.198504	-2.439648	-1.317686
C	-3.239483	0.624996	-4.017070
H	-3.414965	-0.413823	-3.725592
H	-3.560826	0.758519	-5.050317
H	-3.834595	1.276636	-3.378572
C	1.221304	1.842193	2.973186

H 1.225300 0.921526 3.559599
H 0.194072 2.118907 2.738940
H 1.675365 2.638874 3.561547

AAA_6.98_NtA.xyz
34
0 eng= -818.702696864 zpe= -818.408793
N -0.958108 -0.620939 -3.925949
C -1.782129 -0.028034 -2.814471
C -2.138659 -1.267683 -1.971354
N -3.334571 -1.286568 -1.366603
C -3.911003 -2.569114 -0.926739
C -4.304647 -3.242036 -2.250001
N -3.414450 -4.135789 -2.749130
C -3.546550 -4.551919 -4.134801
C -3.621436 -3.301590 -5.015115
O -1.319422 -2.171593 -1.919616
H -0.479610 0.087037 -4.483138
H -1.586339 -1.196576 -4.519916
H -4.010814 -0.573159 -1.605222
H -3.124005 -3.123316 -0.414594
O -5.290049 -2.885289 -2.865109
H -2.516238 -4.216088 -2.294519
O -4.376437 -3.459662 -6.080653
H -4.367872 -2.649184 -6.610264
O -2.997078 -2.277717 -4.797844
H -0.283532 -1.271094 -3.500035
C -5.109039 -2.335010 -0.028181
H -5.515118 -3.291322 0.302121
H -5.894334 -1.810756 -0.575860
H -4.817598 -1.756678 0.848715
H -2.600761 -5.029160 -4.413442
C -4.690701 -5.536569 -4.359251
H -4.544424 -6.396613 -3.706381
H -4.703819 -5.877396 -5.393652
H -5.646547 -5.068520 -4.128725
H -1.109371 0.600757 -2.226236
C -2.939888 0.770394 -3.385872
H -3.637321 0.119218 -3.917819
H -2.578865 1.543374 -4.067098
H -3.467339 1.287913 -2.583043

AAA_8.34_OtB.xyz
34
0 eng= -818.698923913 zpe= -818.408276
N 0.321334 3.375547 -2.234164
C 0.616966 2.890468 -0.892223
C 1.581998 1.720593 -0.977840
N 1.869845 1.249742 -2.168611
C 2.936826 0.311364 -2.556404

C	2.898494	-0.985949	-1.761697
N	3.114459	-2.115729	-2.428410
C	3.496124	-3.330264	-1.719314
C	4.874945	-3.101769	-1.095219
O	5.211348	-4.124058	-0.314132
H	6.089142	-3.961431	0.061282
O	2.033734	1.250467	0.114876
H	0.733936	4.281502	-2.420260
H	-0.676178	3.450067	-2.394754
H	1.394530	1.790293	-2.898819
H	2.761181	0.092846	-3.610672
O	2.757158	-0.975945	-0.530949
H	3.320325	-2.068091	-3.417134
O	5.558368	-2.138591	-1.299382
H	2.447971	0.281785	-0.038230
C	4.325203	0.938983	-2.371163
H	4.478469	1.222789	-1.328294
H	4.419479	1.822197	-3.002959
H	5.097918	0.215664	-2.636384
H	2.786377	-3.493401	-0.905884
C	3.505396	-4.525288	-2.667309
H	3.794159	-5.421993	-2.121418
H	4.221786	-4.373323	-3.478979
H	2.511712	-4.677025	-3.089331
H	1.136749	3.641709	-0.287630
C	-0.638318	2.434488	-0.142420
H	-1.177566	1.685051	-0.727588
H	-1.291716	3.291832	0.019533
H	-0.378480	2.006374	0.824709

AAA_12.15_t-t_O1-ctggtgtt.xyz
34
0 eng= -818.697851493 zpe= -818.406823

N	-3.339472	0.196585	-2.533089
C	-2.486461	1.215550	-1.935740
C	-1.598108	0.573093	-0.888289
N	-1.813195	-0.682660	-0.581124
C	-1.239432	-1.433779	0.549118
C	0.282522	-1.477269	0.380952
N	0.901814	-2.645746	0.267716
C	2.292119	-2.696606	-0.164412
C	2.368536	-2.147313	-1.591876
O	-0.698698	1.294195	-0.344177
H	-3.059042	-0.034876	-3.478447
H	-4.310002	0.485515	-2.557849
H	-2.554995	-1.097972	-1.153569
H	-1.413986	-0.846281	1.457429
O	0.903764	-0.406085	0.363685
H	0.354302	-3.492175	0.201253
H	2.878885	-2.031903	0.473294

O	3.636053	-2.046765	-1.983116
H	3.663579	-1.701342	-2.887118
O	1.418456	-1.860446	-2.264363
H	0.043751	0.690219	0.100045
C	-1.942284	-2.776334	0.655819
H	-1.560388	-3.340507	1.507577
H	-1.816685	-3.364198	-0.257299
H	-3.009823	-2.623458	0.817821
C	2.828007	-4.122344	-0.086186
H	2.775663	-4.485946	0.940310
H	3.867325	-4.139993	-0.409729
H	2.255577	-4.790690	-0.734573
H	-1.799041	1.658697	-2.663468
C	-3.290302	2.333817	-1.264914
H	-2.631440	3.037433	-0.757595
H	-3.989443	1.914151	-0.537015
H	-3.856279	2.872107	-2.025589

AAA_12.61_NtA.xyz
34
0 eng= -818.700201073 zpe= -818.406649

N	0.044327	2.490826	0.325575
C	-0.725422	2.053304	-0.887562
C	-0.415622	0.549505	-0.967152
N	-1.395452	-0.232599	-1.445272
C	-1.425915	-1.695996	-1.223180
C	-1.663758	-1.828778	0.287799
N	-0.564950	-1.908503	1.084190
C	-0.742946	-1.647551	2.494048
C	-1.379066	-0.275960	2.697571
O	0.686882	0.180583	-0.595896
H	0.062911	3.505240	0.434628
H	0.999527	2.119852	0.227248
H	-2.303782	0.195667	-1.564350
H	-2.348906	-2.041768	-1.687981
O	-2.784913	-1.737272	0.743622
H	0.344139	-1.740780	0.674366
O	-2.091159	-0.224786	3.804472
H	-2.461068	0.662228	3.921115
O	-1.209000	0.687572	1.972507
H	-0.363804	2.029425	1.161705
C	-0.243482	-2.438708	-1.825775
H	-0.362143	-3.506821	-1.637864
H	0.713408	-2.111242	-1.424362
H	-0.228419	-2.281186	-2.904965
H	-1.432974	-2.380628	2.915231
C	0.596492	-1.692777	3.227628
H	0.454822	-1.498102	4.290602
H	1.285299	-0.948563	2.819488
H	1.039182	-2.682424	3.113212

H   -1.779588   2.236257   -0.674511
C   -0.245501   2.801080   -2.121949
H   0.815458   2.608488   -2.296352
H   -0.410207   3.875811   -2.023977
H   -0.799542   2.454959   -2.995037

AAA_13.03_c-t_N-gcggtgtt.xyz
34
0 eng= -818.700205336 zpe= -818.406487
N   -3.675921   1.316682   -0.562447
C   -3.028582   -0.036968   -0.688674
C   -2.657527   -0.135970   -2.179778
N   -1.348989   -0.269789   -2.520302
C   -0.194049   0.183927   -1.735852
C   -0.390815   1.680547   -1.442226
N   -0.006344   2.551882   -2.389194
C   -0.349362   3.959920   -2.318398
C   -1.864526   4.138945   -2.396380
O   -3.562135   -0.117609   -2.983234
H   -4.197149   1.403532   0.311694
H   -4.311742   1.462119   -1.357839
H   -1.216024   -0.329280   -3.524656
H   -0.194487   -0.311960   -0.764125
O   -0.965389   2.036561   -0.418750
H   0.438319   2.215008   -3.230404
H   -0.062756   4.337939   -1.330933
O   -2.187659   5.423946   -2.465192
H   -3.151025   5.514317   -2.499715
O   -2.677352   3.247268   -2.368127
H   -2.943038   2.045793   -0.608059
C   1.086818   -0.176502   -2.473613
H   1.956733   0.199122   -1.934677
H   1.104370   0.230366   -3.489111
H   1.169863   -1.260639   -2.554501
C   0.382020   4.741617   -3.406195
H   1.458290   4.610357   -3.291034
H   0.150147   5.801610   -3.326810
H   0.083828   4.398760   -4.400029
H   -2.169687   -0.017139   -0.023622
C   -4.019553   -1.128692   -0.321281
H   -3.543069   -2.102449   -0.440897
H   -4.889453   -1.094994   -0.978644
H   -4.332609   -1.033979   0.720571

AAA_13.48_t-t_N-gtgttgtt.xyz
34
0 eng= -818.69986242 zpe= -818.406316
N   0.571213   1.641112   -1.239217
C   -0.920784   1.777401   -1.462823
C   -1.460034   1.120756   -0.182000

N	-1.873236	1.951231	0.813400
C	-1.505300	1.555994	2.180337
C	0.016837	1.292058	2.130907
N	0.500139	0.215631	2.759107
C	1.915993	-0.100090	2.762595
C	2.425165	-0.415051	1.356839
O	3.713721	-0.740326	1.397313
H	4.023621	-0.938318	0.502196
H	-1.135425	2.846124	-1.508516
O	-1.316617	-0.072684	-0.051412
H	1.113083	2.030123	-2.012144
H	0.816294	0.644603	-1.110196
H	-1.814000	2.941582	0.625717
H	-1.617987	2.453020	2.794546
O	0.742955	2.076052	1.519518
H	-0.142933	-0.466002	3.135426
O	1.786422	-0.366867	0.336184
H	0.849745	2.079048	-0.335970
C	-2.379266	0.448232	2.752217
H	-2.138080	0.280328	3.804451
H	-2.271323	-0.478558	2.189232
H	-3.423298	0.756831	2.703169
C	-1.330830	1.050414	-2.725668
H	-1.117026	-0.015445	-2.630651
H	-0.819133	1.455807	-3.600714
H	-2.404100	1.169405	-2.877516
H	2.467098	0.793100	3.075261
C	2.196030	-1.247371	3.731877
H	1.854530	-0.973560	4.730467
H	3.263655	-1.452292	3.772805
H	1.680782	-2.157566	3.415539

AGA_0.00_NcA.xyz
31
0 eng= -779.396383143 zpe= -779.130568

N	-2.411552	0.042441	-2.693319
C	-2.579496	-1.259061	-1.961344
C	-1.946441	-2.289220	-2.914445
N	-0.972529	-3.102057	-2.434015
C	-0.148918	-2.784334	-1.289533
C	0.352151	-1.331222	-1.335920
N	0.845448	-0.857460	-0.177392
C	1.259248	0.527608	-0.006303
C	0.074506	1.465485	-0.233410
H	-2.060011	-1.130086	-1.013779
O	-2.377347	-2.335413	-4.044471
H	-2.846980	0.815681	-2.186449
H	-2.809967	-0.055925	-3.634553
H	-0.581180	-3.710260	-3.142490
H	0.712269	-3.454729	-1.292537

H	-0.684340	-2.959132	-0.351140
O	0.250521	-0.656424	-2.349274
H	0.868215	-1.465465	0.627702
H	1.526565	0.647849	1.047090
O	0.436764	2.725385	-0.033493
H	-0.328385	3.307094	-0.148619
O	-1.046684	1.132960	-0.534754
H	-1.398186	0.233780	-2.765209
C	2.471853	0.910802	-0.862776
H	3.297619	0.239414	-0.627785
H	2.774417	1.932154	-0.636777
H	2.237915	0.829223	-1.923102
C	-4.054488	-1.558703	-1.750909
H	-4.158535	-2.508589	-1.224948
H	-4.566501	-1.646572	-2.710593
H	-4.527991	-0.783684	-1.145185

AGA_3.24_NcB.xyz
31
0 eng= -779.394525601 zpe= -779.129334

N	-3.743877	0.573788	-0.607380
C	-3.319250	-0.693928	-1.285361
C	-1.836728	-0.456082	-1.627657
N	-1.045193	-1.542098	-1.747132
C	-1.381618	-2.867459	-1.246434
C	-1.679151	-2.872819	0.256690
N	-0.864057	-2.115639	1.034719
C	-1.158108	-1.970530	2.442030
C	-2.479713	-1.236025	2.625347
O	-1.473189	0.684429	-1.836891
H	-4.745231	0.746682	-0.703941
H	-3.203695	1.342358	-1.037518
H	-0.114515	-1.355429	-2.098724
H	-0.542006	-3.531917	-1.453806
H	-2.259286	-3.283297	-1.744273
O	-2.615478	-3.496390	0.707017
H	-0.153109	-1.532341	0.619230
O	-2.976333	-1.429811	3.830793
H	-3.801877	-0.934526	3.935841
O	-3.001596	-0.523303	1.793936
H	-3.501217	0.502179	0.395612
H	-3.483754	-1.500438	-0.571731
C	-4.111083	-0.905629	-2.571466
H	-3.761256	-1.808090	-3.074168
H	-3.972604	-0.063427	-3.253151
H	-5.173350	-1.035585	-2.356931
H	-1.284963	-2.960044	2.887286
C	-0.043417	-1.205969	3.155596
H	0.896034	-1.749271	3.051094
H	-0.271281	-1.112695	4.216670

H 0.074005 -0.204747 2.733341

AGA_3.65_NtA.xyz
31
0 eng= -779.394500337 zpe= -779.129179

N	-1.085438	1.692602	-1.392451
C	-0.787873	0.568325	-2.343921
C	-0.463065	-0.599628	-1.404677
N	-0.832850	-1.823941	-1.810213
C	-0.904821	-2.909355	-0.828339
C	-2.174341	-2.620431	-0.029400
N	-2.000753	-1.929641	1.129541
C	-3.156395	-1.289394	1.712478
C	-3.743101	-0.280384	0.731073
O	0.106499	-0.352222	-0.354870
H	-1.162802	2.594526	-1.863897
H	-0.323007	1.712459	-0.700510
H	-1.457163	-1.900074	-2.602265
H	0.003444	-2.906143	-0.228001
H	-1.007857	-3.857033	-1.351095
O	-3.269544	-2.874941	-0.482305
H	-1.085191	-1.547617	1.327716
O	-5.025284	-0.071651	0.948579
H	-5.366427	0.589331	0.328906
O	-3.106872	0.313895	-0.120005
H	-1.953328	1.468152	-0.868961
H	-1.700656	0.384223	-2.912308
C	0.389110	0.924336	-3.239263
H	1.279600	1.123943	-2.639114
H	0.164437	1.792838	-3.861324
H	0.608445	0.085223	-3.900228
H	-3.928265	-2.038692	1.895816
C	-2.787817	-0.574447	3.011685
H	-3.666875	-0.099756	3.446892
H	-2.026537	0.189109	2.831485
H	-2.395817	-1.299895	3.724445

AGA_8.38_OtA.xyz
31
0 eng= -779.389555204 zpe= -779.127378

N	-1.014546	-0.316143	-4.630823
C	-1.786132	0.714386	-3.948627
C	-1.364151	0.776968	-2.492469
N	-0.524349	-0.136395	-2.057304
C	-0.203826	-0.448845	-0.663842
C	0.331378	0.744330	0.115555
N	1.380289	0.550608	0.889816
C	1.936122	1.601213	1.741790
C	3.317144	1.112263	2.146768
O	-1.848454	1.698542	-1.765448

H	-0.340587	0.070949	-5.280198
H	-1.609981	-0.954358	-5.145197
H	-0.236997	-0.773431	-2.806697
H	0.505762	-1.273932	-0.667001
H	-1.112284	-0.783107	-0.150300
O	-0.255088	1.839831	0.050250
H	1.888477	-0.329458	0.891348
O	3.979660	2.037623	2.826336
H	4.842989	1.684141	3.087884
O	3.729478	0.013531	1.892068
H	-1.254743	1.830558	-0.876535
H	-1.586623	1.712977	-4.350454
C	-3.294831	0.450876	-3.998954
H	-3.518288	-0.548543	-3.616642
H	-3.633483	0.515113	-5.033219
H	-3.839495	1.185581	-3.407415
H	2.044329	2.512534	1.147648
C	1.062993	1.878744	2.967658
H	0.965680	0.979364	3.578534
H	0.075674	2.207819	2.645590
H	1.517203	2.666792	3.567473

AGA_8.86_t-t_N-gtgttgtt.xyz
31
0 eng= -779.392747511 zpe= -779.127194

N	-0.205154	1.441196	0.438003
C	-1.263163	1.236635	-0.627882
C	-0.603584	0.139365	-1.475773
N	-1.023070	-1.141299	-1.275288
C	0.049101	-2.125318	-1.280867
C	1.045828	-1.700496	-0.190607
N	2.348751	-1.858342	-0.447553
C	3.373222	-1.441127	0.491551
C	3.341251	0.071496	0.708621
O	0.371827	0.423950	-2.129676
H	-0.476570	2.154245	1.116661
H	0.690092	1.709054	-0.002244
H	-1.738796	-1.285685	-0.577255
H	0.513032	-2.166605	-2.265249
H	-0.360236	-3.105731	-1.036951
O	0.630822	-1.213492	0.859251
H	2.636543	-2.136366	-1.375282
O	4.308630	0.436215	1.543779
H	4.280279	1.395053	1.672000
O	2.555391	0.845796	0.223609
H	0.013497	0.542908	0.917965
H	-2.159966	0.885775	-0.114690
C	-1.496797	2.523562	-1.390203
H	-0.586431	2.818329	-1.914666
H	-1.818937	3.325876	-0.723684

H	-2.280805	2.365393	-2.131403
H	3.147759	-1.880876	1.468615
C	4.746384	-1.911015	0.015270
H	5.508973	-1.635919	0.740705
H	5.001151	-1.454811	-0.944412
H	4.743953	-2.995681	-0.095043

AGA_9.04_OtA.xyz
31
0 eng= -779.389247369 zpe= -779.127124

N	-0.154290	2.220722	-2.829946
C	-0.062346	2.480051	-1.398776
C	-0.041938	1.160370	-0.648581
N	-0.238841	0.059685	-1.339937
C	-0.019731	-1.318154	-0.897885
C	-0.795397	-1.688688	0.358126
N	-1.377092	-2.871255	0.389680
C	-2.037470	-3.392830	1.585657
C	-2.176521	-4.889371	1.357076
O	0.144904	1.187774	0.606899
H	0.702523	2.443538	-3.321845
H	-0.904354	2.749389	-3.259113
H	-0.363719	0.261070	-2.336927
H	1.042438	-1.459318	-0.667263
H	-0.274377	-1.972375	-1.729557
O	-0.807023	-0.914998	1.332496
H	-1.327157	-3.512583	-0.397211
O	-2.649859	-5.502442	2.432613
H	-2.745673	-6.448102	2.244899
O	-1.907148	-5.425892	0.317025
H	-0.186629	0.268649	1.058663
H	0.878776	2.971405	-1.128061
C	-1.228642	3.324266	-0.876657
H	-2.181767	2.856473	-1.136600
H	-1.187054	4.314052	-1.331489
H	-1.173801	3.435479	0.205386
H	-1.385181	-3.224592	2.446992
C	-3.400102	-2.740122	1.828752
H	-3.846717	-3.155010	2.731802
H	-4.068080	-2.925164	0.985485
H	-3.270526	-1.666743	1.961538

AGA_11.83_NtA.xyz
31
0 eng= -779.392234491 zpe= -779.126064

N	-2.832557	-1.239683	-1.702193
C	-2.575244	-1.206828	-0.219301
C	-1.084271	-1.582379	-0.135180
N	-0.679906	-2.295699	0.927122
C	0.579106	-3.039293	0.830616

C	0.257142	-4.206923	-0.101478
N	0.631928	-4.055444	-1.396864
C	0.051831	-4.936591	-2.395594
C	-1.472688	-4.895508	-2.256566
O	-0.355805	-1.227593	-1.047097
H	-3.734557	-0.835699	-1.954891
H	-2.768985	-2.227268	-2.017430
H	-1.381336	-2.702205	1.530053
H	0.845339	-3.417197	1.814748
H	1.358950	-2.377631	0.457792
O	-0.424040	-5.136262	0.282793
H	0.993945	-3.157546	-1.684340
O	-2.053407	-6.031329	-2.576990
H	-3.014266	-5.942763	-2.496124
O	-2.101907	-3.899442	-1.945025
H	-2.063134	-0.736633	-2.164674
H	-2.682613	-0.166268	0.096436
C	-3.552905	-2.115645	0.504171
H	-3.395019	-3.160084	0.224610
H	-4.583417	-1.835854	0.277033
H	-3.434361	-2.007271	1.583381
H	0.251501	-4.483561	-3.372685
C	0.639145	-6.344973	-2.367118
H	1.718085	-6.275981	-2.502382
H	0.220549	-6.949536	-3.170664
H	0.425787	-6.826766	-1.414180

AGA_12.09_c-t_N-gcggtgtt.xyz
31
0 eng= -779.391077103 zpe= -779.125964

C	-3.034654	-0.994318	-0.621654
H	-2.637212	-1.775829	0.019219
C	-2.251321	-0.795212	-1.930712
O	-2.563775	0.139271	-2.634470
N	-2.813794	0.298839	0.116884
H	-3.378420	0.346479	0.966789
H	-3.068144	1.075340	-0.507340
N	-1.267416	-1.676886	-2.236734
H	-0.814088	-1.476923	-3.120043
C	-0.572473	-2.541635	-1.286002
H	0.186699	-3.095189	-1.838330
H	-1.251089	-3.267409	-0.838157
C	0.043089	-1.700658	-0.162774
O	-0.550467	-1.521085	0.889108
N	1.221098	-1.106365	-0.440017
H	1.659694	-1.248481	-1.338151
C	1.825604	-0.156379	0.472221
H	1.877674	-0.611278	1.467509
C	0.937746	1.073237	0.635584
O	1.511878	1.972209	1.419210

H	0.929291	2.739419	1.517354
O	-0.155319	1.226428	0.141060
H	-1.812049	0.419573	0.360597
C	3.228936	0.218266	-0.000246
H	3.841745	-0.680538	-0.073135
H	3.694325	0.898980	0.709541
H	3.193880	0.708077	-0.976407
C	-4.514373	-1.181799	-0.912934
H	-4.657770	-2.113169	-1.462095
H	-4.892753	-0.364272	-1.528731
H	-5.088016	-1.248286	0.013715

AGA_13.84_t-t_O1-ctggtgtt.xyz
31
0 eng= -779.387855595 zpe= -779.125296

N	-1.971205	-0.032445	0.363280
C	-1.892286	0.214618	-1.070269
C	-1.302549	1.591543	-1.308355
N	-1.092969	2.358728	-0.264571
C	-0.765956	3.785401	-0.274787
C	0.520108	4.066171	-1.038860
N	1.473657	4.770488	-0.443314
C	2.784939	4.907810	-1.065393
C	3.438236	3.524116	-1.115232
O	-1.055564	1.937616	-2.508083
H	-1.297899	-0.722221	0.674244
H	-2.892667	-0.348380	0.641341
H	-1.330563	1.882575	0.611107
H	-0.710252	4.117260	0.759901
H	-1.570505	4.337373	-0.771529
O	0.629695	3.669593	-2.206616
H	1.391815	4.984292	0.540946
O	4.573873	3.572422	-1.806804
H	4.976532	2.691841	-1.816455
O	2.993677	2.542513	-0.591899
H	-0.376112	2.751927	-2.535467
H	-1.210808	-0.480450	-1.571236
C	-3.260802	0.151900	-1.756197
H	-3.962394	0.831897	-1.266150
H	-3.648895	-0.864363	-1.684731
H	-3.182835	0.422385	-2.808449
H	2.644335	5.242357	-2.095587
C	3.644149	5.902337	-0.291680
H	3.801688	5.564481	0.735916
H	3.165647	6.881926	-0.278250
H	4.616453	5.995769	-0.772508

AGA_13.89_c-t_N-gcggtgtt.xyz
31
0 eng= -779.390937979 zpe= -779.125279

N	-2.309990	-0.872923	-1.013634
C	-3.350173	-1.959942	-0.950121
C	-2.925087	-2.951158	-2.049043
N	-2.605427	-4.222186	-1.689296
C	-2.122094	-4.635348	-0.373373
C	-0.885958	-3.810246	-0.008701
N	0.288393	-4.241693	-0.500217
C	1.487342	-3.426454	-0.439011
C	1.299150	-2.151271	-1.259518
H	-3.291693	-2.366883	0.055673
O	-2.923611	-2.556090	-3.191990
H	-2.613528	-0.034898	-0.514362
H	-2.127459	-0.637993	-1.998222
H	-2.352430	-4.798883	-2.482805
H	-1.903936	-5.702072	-0.421334
H	-2.879745	-4.485400	0.395266
O	-0.994080	-2.761705	0.614947
H	0.322052	-5.091968	-1.044008
H	1.625036	-3.090239	0.594440
O	2.427095	-1.457053	-1.323948
H	2.281457	-0.647774	-1.835205
O	0.263169	-1.790319	-1.763563
H	-1.426460	-1.222213	-0.603966
C	2.702916	-4.231368	-0.890227
H	2.816552	-5.110603	-0.255617
H	3.603610	-3.626201	-0.811500
H	2.594597	-4.551189	-1.929521
C	-4.724033	-1.390687	-1.262872
H	-5.460397	-2.194885	-1.238338
H	-4.739393	-0.949126	-2.260264
H	-5.014612	-0.646810	-0.518210

GAG_0.00_NcA.xyz
28
0 eng= -740.087151396 zpe= -739.848978

N	-1.410536	-1.647200	-3.416054
C	-1.306253	-1.130722	-2.018863
C	-2.748780	-1.142178	-1.496180
N	-3.007901	-1.780910	-0.331309
C	-2.189604	-2.838823	0.236931
C	-1.727473	-3.806862	-0.866016
N	-0.666547	-4.578233	-0.555124
C	-0.083742	-5.461679	-1.536273
C	0.603644	-4.704015	-2.661399
H	-0.577497	-1.746027	-1.498173
H	-0.966822	-0.095982	-2.056612
O	-3.574426	-0.563327	-2.165247
H	-0.511353	-1.625767	-3.900799
H	-2.123432	-1.104863	-3.918054
H	-3.992730	-1.780441	-0.090054

O	-2.266379	-3.852820	-1.962014
H	-0.214938	-4.474230	0.341262
H	0.637836	-6.124793	-1.060279
H	-0.855717	-6.086355	-1.994469
O	1.274641	-5.539928	-3.436541
H	1.701151	-5.056338	-4.159233
O	0.534095	-3.512801	-2.844728
H	-1.714102	-2.634190	-3.352387
H	-1.299992	-2.405897	0.708588
C	-3.001825	-3.587528	1.295111
H	-2.402186	-4.364771	1.768587
H	-3.875890	-4.055498	0.837451
H	-3.330204	-2.891699	2.068085

GAG_3.52_NtA.xyz
28
0 eng= -740.084775909 zpe= -739.847639

N	-0.212573	0.835943	-1.275947
C	-1.407375	0.501812	-2.105682
C	-2.566790	0.487465	-1.103034
N	-3.593602	-0.324514	-1.377099
C	-4.542948	-0.708612	-0.315834
C	-3.739443	-1.721718	0.512780
N	-3.065479	-1.208352	1.580474
C	-2.007888	-2.005833	2.138434
C	-0.841814	-2.153165	1.173938
O	-0.048600	-3.146311	1.517996
H	0.690951	-3.218620	0.897316
H	-1.549983	1.282242	-2.853744
H	-1.243004	-0.461361	-2.585672
O	-2.470454	1.198680	-0.116015
H	0.599819	1.114830	-1.826028
H	0.004125	0.013973	-0.682521
H	-3.503963	-0.984160	-2.140193
O	-3.619959	-2.874057	0.159461
H	-3.035972	-0.204260	1.690165
H	-1.630321	-1.549966	3.056046
H	-2.369609	-3.004896	2.383650
O	-0.635369	-1.431536	0.216670
H	-0.497961	1.588831	-0.631096
H	-4.765752	0.190254	0.259892
C	-5.794577	-1.319503	-0.912483
H	-6.488868	-1.588229	-0.116144
H	-5.547460	-2.231433	-1.458945
H	-6.283463	-0.607822	-1.577972

GAG_3.57_OtA.xyz
28
0 eng= -740.081645699 zpe= -739.847619

N	-2.171524	1.222348	-3.997215

C	-2.051414	-0.161385	-4.416899
C	-0.988856	-0.884223	-3.620890
N	-0.330423	-0.193534	-2.718589
C	0.541505	-0.694620	-1.641650
C	1.666439	-1.571094	-2.182778
N	2.880815	-1.365703	-1.706722
C	4.004570	-2.213108	-2.065395
C	5.220185	-1.682769	-1.333625
H	-2.972386	-0.736479	-4.283298
H	-1.774203	-0.269040	-5.468934
O	-0.790629	-2.111037	-3.881981
H	-3.090987	1.438139	-3.633788
H	-1.974875	1.871052	-4.748551
H	-0.654975	0.777944	-2.684118
H	0.970163	0.193417	-1.175762
O	1.425114	-2.491615	-2.982273
H	3.089699	-0.608901	-1.061597
H	3.820813	-3.251038	-1.777581
H	4.182554	-2.194376	-3.143146
O	6.295727	-2.406338	-1.602904
H	7.055299	-2.045589	-1.121273
O	5.183395	-0.727500	-0.608868
H	0.147875	-2.435446	-3.476176
C	-0.263190	-1.491202	-0.607781
H	0.391999	-1.822746	0.199152
H	-0.718991	-2.370652	-1.066363
H	-1.046461	-0.862317	-0.184581

GAG_4.20_NcB.xyz
28
0 eng= -740.084462387 zpe= -739.84738

N	-1.981468	1.207030	1.831186
C	-1.525407	0.073725	0.977037
C	-2.149471	0.340221	-0.403511
N	-1.574777	-0.247299	-1.468543
C	-0.247281	-0.864617	-1.482333
C	0.827360	0.141067	-1.046961
N	0.761617	1.378445	-1.609478
C	1.628693	2.410618	-1.109442
C	1.245420	2.842302	0.295469
H	-0.438335	0.034642	1.002409
H	-1.941018	-0.857918	1.365828
O	-3.151144	1.027708	-0.447623
H	-2.890897	1.519162	1.446881
H	-1.298506	1.975649	1.727470
H	-2.078618	-0.141055	-2.341992
H	-0.202202	-1.674779	-0.750482
O	1.672863	-0.157466	-0.231489
H	-0.035630	1.632753	-2.172142
H	2.662016	2.059860	-1.076132

H	1.594024	3.286750	-1.758815
O	2.139457	3.666112	0.802967
H	1.879056	3.940854	1.694266
O	0.241992	2.491177	0.878063
H	-2.073334	0.957375	2.815549
C	0.047278	-1.422954	-2.870110
H	1.042220	-1.867407	-2.881540
H	0.015384	-0.634435	-3.625952
H	-0.680065	-2.193889	-3.128243

GAG_5.10_OtA.xyz
28
0 eng= -740.081067417 zpe= -739.847037

N	-1.121325	-0.065955	-4.662342
C	-1.817140	0.922965	-3.859839
C	-1.386940	0.853938	-2.413320
N	-0.455266	-0.015054	-2.090779
C	-0.084236	-0.458967	-0.734413
C	0.415167	0.727663	0.095176
N	1.546160	0.617138	0.762934
C	2.041624	1.680630	1.619418
C	3.355658	1.208084	2.205862
H	-1.625324	1.948046	-4.188423
H	-2.904606	0.802053	-3.858662
O	-1.942490	1.639097	-1.586338
H	-0.601171	0.353318	-5.422247
H	-1.749710	-0.752773	-5.059470
H	-0.130549	-0.550718	-2.901243
H	-1.006944	-0.777067	-0.233077
O	-0.281483	1.756745	0.163938
H	2.130073	-0.212282	0.711771
H	2.195541	2.601187	1.051544
H	1.332642	1.901197	2.421178
O	3.882331	2.122389	3.005125
H	4.717847	1.790876	3.367013
O	3.834271	0.135767	1.958865
H	-1.329869	1.735654	-0.702698
C	0.869883	-1.637902	-0.845053
H	1.075963	-2.059629	0.139262
H	1.810680	-1.347329	-1.318470
H	0.410924	-2.427980	-1.440604

GAG_5.65_OtB.xyz
28
0 eng= -740.081256487 zpe= -739.846827

N	-0.745572	1.953823	-2.423215
C	0.138253	2.333585	-1.336958
C	-0.597502	2.357845	-0.016425
N	-1.876801	2.066240	-0.028615
C	-2.761967	1.751295	1.106448

C	-2.741951	2.827376	2.181846
N	-3.906231	3.160769	2.734781
C	-3.918176	3.847777	4.011443
C	-3.415893	2.906778	5.102146
H	0.978330	1.645863	-1.202014
H	0.574934	3.327595	-1.464842
O	0.069760	2.670543	1.019089
H	-0.450381	1.102320	-2.883272
H	-0.823277	2.676779	-3.126985
H	-2.195289	1.851671	-0.979250
O	-1.675405	3.309988	2.583039
H	-4.741642	2.673889	2.443786
H	-3.271639	4.725115	3.973035
H	-4.929534	4.178592	4.245268
O	-3.385473	3.527225	6.275081
H	-3.063593	2.915198	6.953364
O	-3.099512	1.768110	4.907993
H	-0.583469	2.920674	1.820230
H	-3.764462	1.683074	0.681717
C	-2.375138	0.418223	1.761918
H	-1.349962	0.459144	2.134441
H	-2.464117	-0.389327	1.035231
H	-3.029677	0.218182	2.611704

GAG_6.67_c-t_N-gccttgtt.xyz
28
0 eng= -740.08462411 zpe= -739.846437

N	-0.430681	1.657605	-1.483604
C	-1.122858	0.440470	-2.002062
C	-0.655856	-0.690530	-1.076763
N	-0.102359	-1.799730	-1.619170
C	0.516582	-1.893179	-2.935931
C	1.313556	-0.605599	-3.231627
N	1.555838	-0.324679	-4.526520
C	2.279416	0.868983	-4.895085
C	1.479769	2.136524	-4.640673
O	2.106046	3.184895	-5.149482
H	1.599988	3.992503	-4.976933
H	-2.196398	0.566604	-1.863347
H	-0.889133	0.360194	-3.059431
O	-0.821272	-0.519108	0.110381
H	-0.714869	2.501574	-1.984541
H	0.582619	1.517990	-1.642624
H	0.199327	-2.464069	-0.917001
O	1.704137	0.125201	-2.331887
H	1.182463	-0.913445	-5.253741
H	3.203303	0.950275	-4.315430
H	2.551774	0.827815	-5.949247
O	0.433432	2.194174	-4.041741
H	-0.609077	1.737486	-0.475896

```
H       1.278441        -2.674012       -2.848337
C       -0.472800       -2.315029       -4.025764
H       0.039438        -2.616341       -4.942104
H       -1.187198       -1.524376       -4.265802
H       -1.032837       -3.181153       -3.674904
```

```
GAG_8.23_t-t_O1-ctggtgtt.xyz
28
0 eng= -740.080311011 zpe= -739.845845
N       -0.246239       1.093050        -4.373313
C       -1.366589       0.171241        -4.357902
C       -1.343563       -0.693298       -3.118624
N       -0.361301       -0.511865       -2.269715
C       -0.223109       -1.045207       -0.902724
C       -0.318530       -2.567722       -0.908444
N       0.645047        -3.257692       -0.305324
C       0.673934        -4.701989       -0.424291
C       0.994746        -5.100656       -1.861515
H       -2.338702       0.673126        -4.359430
H       -1.373436       -0.514471       -5.209310
O       -2.278035       -1.546542       -2.983366
H       -0.540661       2.060958        -4.390662
H       0.366901        0.939336        -5.163682
H       0.279553        0.219628        -2.593568
O       -1.302823       -3.127705       -1.406374
H       1.480886        -2.778528       -0.005300
H       1.426959        -5.112436       0.247615
H       -0.294641       -5.122060       -0.149764
O       0.989016        -6.423884       -1.981875
H       1.193952        -6.663120       -2.897607
O       1.219412        -4.323202       -2.743234
H       -2.024244       -2.270404       -2.256822
H       0.770986        -0.742181       -0.572624
C       -1.286499       -0.462190       0.031725
H       -1.147517       -0.854199       1.040411
H       -2.289026       -0.729984       -0.305850
H       -1.196955       0.623634        0.062522
```

```
GAG_8.29_c-t_N-tcggtgtt.xyz
28
0 eng= -740.08333287 zpe= -739.845821
N       0.552341        -0.251566       -3.741203
C       -0.756953       -0.545442       -3.089597
C       -1.062244       0.698426        -2.239054
N       -1.894621       0.579008        -1.188505
C       -2.378811       -0.660858       -0.564260
C       -1.182924       -1.562503       -0.199415
N       -0.227560       -0.985775       0.577336
C       0.988217        -1.706186       0.840351
C       1.854260        -1.843133       -0.399079
```

H	-1.525091	-0.623630	-3.860796
H	-0.676090	-1.483906	-2.545788
O	-0.562340	1.749816	-2.593009
H	1.302010	-0.548821	-3.095462
H	0.600104	0.776093	-3.855113
H	-2.140337	1.465415	-0.764584
O	-1.088405	-2.702715	-0.598886
H	-0.278561	-0.001948	0.791839
H	1.570691	-1.199611	1.611310
H	0.767229	-2.713876	1.199015
O	2.893781	-2.619699	-0.168421
H	3.445170	-2.693408	-0.960888
O	1.634522	-1.303826	-1.461720
H	0.665869	-0.719513	-4.639985
H	-2.835388	-0.343754	0.376768
C	-3.416668	-1.406573	-1.388925
H	-3.791044	-2.258967	-0.822489
H	-2.994535	-1.800196	-2.314152
H	-4.246794	-0.741086	-1.625914

GAG_9.60_t-t_O1-ctggtgtt.xyz
28
0 eng= -740.079997309 zpe= -739.845321

N	-1.168975	2.339258	-3.505243
C	-0.840119	1.089106	-2.846095
C	-2.043884	0.180581	-2.775628
N	-3.170453	0.613978	-3.285171
C	-4.506700	0.010295	-3.134286
C	-4.482970	-1.390157	-3.752910
N	-5.326042	-1.693994	-4.733970
C	-5.124770	-2.917057	-5.485166
C	-3.832755	-2.819505	-6.291334
O	-3.621803	-3.946281	-6.961505
H	-2.799893	-3.869338	-7.467953
H	-0.057281	0.522489	-3.357451
H	-0.499747	1.222066	-1.814800
O	-1.899298	-0.956064	-2.220449
H	-0.643380	2.471712	-4.359955
H	-1.008164	3.143325	-2.912404
H	-3.074029	1.543555	-3.705737
O	-3.682708	-2.221218	-3.305326
H	-5.898065	-0.966395	-5.135556
H	-5.053811	-3.769301	-4.808052
H	-5.966298	-3.081748	-6.157085
O	-3.120742	-1.856452	-6.309963
H	-2.666160	-1.613847	-2.523306
H	-4.674288	-0.150484	-2.063177
C	-5.547172	0.971324	-3.683338
H	-6.550628	0.566783	-3.544963
H	-5.379074	1.177726	-4.743635

```
H       -5.501767       1.915992        -3.140251

GAA_0.00_OtA.xyz
31
0 eng= -779.390312283 zpe= -779.128532
N       -0.976389       0.045451        -4.784757
C       -1.731327       1.014864        -4.012353
C       -1.378718       0.939602        -2.545096
N       -0.474960       0.059544        -2.175039
C       -0.189708       -0.391294       -0.800678
C       0.287460        0.786735        0.054581
N       1.384720        0.659039        0.770729
C       1.884816        1.719307        1.644321
C       3.328762        1.352533        1.945344
H       -1.549676       2.047620        -4.321354
H       -2.813740       0.865168        -4.069462
O       -1.968230       1.726610        -1.744757
H       -0.389981       0.483386        -5.483477
H       -1.572844       -0.622889       -5.255423
H       -0.106749       -0.476096       -2.966219
O       -0.402968       1.824909        0.087299
H       1.973259        -0.166152       0.704870
O       3.940408        2.307870        2.630963
H       4.848208        2.030345        2.824841
O       3.828499        0.311731        1.613655
H       -1.393902       1.815577        -0.826909
H       -1.145736       -0.688963       -0.351750
C       0.742409        -1.591015       -0.858650
H       0.888629        -2.011543       0.136721
H       1.712945        -1.324648       -1.284027
H       0.296830        -2.373514       -1.474068
H       1.866012        2.664667        1.095638
C       1.065191        1.848458        2.930547
H       1.091063        0.914714        3.495408
H       0.033338        2.094248        2.682357
H       1.479102        2.646539        3.546006

GAA_0.31_OtA.xyz
31
0 eng= -779.390756565 zpe= -779.128413
N       -1.076309       3.654567        -1.657245
C       0.031899        2.823102        -1.225582
C       -0.371967       1.923714        -0.079293
N       -1.597451       2.038994        0.380010
C       -2.325444       1.126770        1.279650
C       -1.572076       0.888775        2.584768
N       -2.271959       0.920930        3.702190
C       -1.701418       0.573063        5.002534
C       -2.888701       0.324764        5.919140
H       0.413663        2.162436        -2.009649
```

H	0.888000	3.404080	-0.872286
O	0.499879	1.123270	0.378193
H	-1.354972	3.463866	-2.611194
H	-0.867642	4.642114	-1.586190
H	-2.123473	2.750144	-0.137300
O	-0.361496	0.596571	2.571769
H	-3.268253	1.121589	3.700498
O	-2.501269	-0.128272	7.103110
H	-3.278991	-0.251366	7.667775
O	-4.026163	0.540088	5.599742
H	0.201009	0.757268	1.348945
H	-3.270236	1.626003	1.497252
C	-2.590267	-0.221635	0.599687
H	-1.652016	-0.723704	0.356523
H	-3.161662	-0.068835	-0.315968
H	-3.165230	-0.867199	1.265306
H	-1.133797	-0.355582	4.898815
C	-0.796849	1.676315	5.556257
H	-0.402378	1.370767	6.524882
H	-1.358104	2.604459	5.678477
H	0.036074	1.842074	4.874034

GAA_1.24_OtB.xyz
31
0 eng= -779.390201443 zpe= -779.12806

N	-1.233529	3.239212	-1.812661
C	-0.560519	2.110089	-1.197890
C	-0.109188	2.439371	0.207116
N	-0.340650	3.653013	0.650205
C	-0.262544	4.160902	2.030933
C	1.081322	3.882058	2.688444
N	1.605245	4.846101	3.438696
C	2.644643	4.537874	4.413294
C	2.034589	3.630264	5.484472
O	2.973031	3.194000	6.318945
H	2.563754	2.630936	6.992466
H	-1.192214	1.221119	-1.111951
H	0.334271	1.797428	-1.742628
O	0.475948	1.523015	0.863647
H	-2.197928	3.036559	-2.042546
H	-0.771367	3.549143	-2.657729
H	-0.823636	4.216902	-0.056498
O	1.618612	2.768755	2.599011
H	1.088282	5.706318	3.561680
O	0.867913	3.366572	5.562111
H	0.983192	1.935663	1.710368
H	-0.407552	5.239201	1.951561
C	-1.357256	3.544689	2.913265
H	-1.255006	2.458330	2.943604
H	-2.339586	3.804762	2.518746

H	-1.263494	3.916282	3.934853
H	3.433390	3.974469	3.910809
C	3.207412	5.817247	5.023066
H	3.982743	5.566338	5.745135
H	2.426069	6.378691	5.541986
H	3.643430	6.444403	4.245140

GAA_1.50_NcB.xyz
31
0 eng= -779.39292554 zpe= -779.127961

N	-3.001570	0.038161	0.734894
C	-2.503819	-0.888948	-0.320344
C	-3.621380	-0.928747	-1.376437
N	-3.281345	-1.297066	-2.624810
C	-1.914832	-1.343929	-3.148017
C	-1.214265	0.011794	-2.978053
N	-1.944579	1.115262	-3.285543
C	-1.391713	2.427646	-3.044902
C	-1.203964	2.653050	-1.549916
O	-0.389434	3.662368	-1.314341
H	-0.302958	3.808556	-0.361073
H	-1.537718	-0.529583	-0.669026
H	-2.398218	-1.890067	0.101778
O	-4.751975	-0.668802	-1.012843
H	-4.034456	-0.022349	0.721563
H	-2.716643	0.996334	0.468661
H	-4.059013	-1.416402	-3.264272
H	-1.316408	-2.056829	-2.575395
O	-0.074366	0.079968	-2.568641
H	-2.922006	1.031612	-3.523406
O	-1.755352	2.020729	-0.673306
H	-2.640951	-0.180231	1.663383
C	-1.937289	-1.768474	-4.612247
H	-0.919510	-1.790377	-5.001184
H	-2.520069	-1.066646	-5.213849
H	-2.367348	-2.766200	-4.710288
H	-0.397605	2.485375	-3.494435
C	-2.296686	3.516857	-3.620838
H	-2.401944	3.372098	-4.696207
H	-1.859895	4.499085	-3.445726
H	-3.286788	3.487066	-3.158865

GAA_2.33_NcA.xyz
31
0 eng= -779.393675375 zpe= -779.127643

N	-0.961079	-2.992781	-2.381843
C	-1.171165	-1.576893	-2.805409
C	-2.366246	-1.101523	-1.968549
N	-2.235592	0.030413	-1.239322
C	-0.972997	0.594594	-0.792290

```
C       -0.027371       -0.521671       -0.307560
N       1.276158        -0.186731       -0.264808
C       2.323571        -1.120788       0.121391
C       2.381432        -2.292501       -0.856166
O       3.364937        -3.119543       -0.531427
H       3.416181        -3.838056       -1.178371
H       -0.227888       -1.053365       -2.675846
H       -1.467238       -1.567975       -3.854083
O       -3.369500       -1.776924       -2.021817
H       -0.655207       -2.968798       -1.393571
H       -0.228085       -3.452693       -2.924580
H       -3.056460       0.238251        -0.681712
O       -0.441264       -1.635555       -0.017863
H       1.548754        0.740309        -0.556093
H       3.274532        -0.595113       -0.001132
O       1.658670        -2.456317       -1.809821
H       -1.854287       -3.494303       -2.451251
C       2.212517        -1.590020       1.577138
H       3.062589        -2.223466       1.826225
H       1.289106        -2.145098       1.735630
H       2.220967        -0.718509       2.231519
H       -0.489396       1.122887        -1.621851
C       -1.243417       1.582835        0.343500
H       -0.319992       2.057054        0.675409
H       -1.690353       1.063334        1.193957
H       -1.924247       2.363850        0.002420

GAA_4.45_c-t_N-gccttgtt.xyz
31
0 eng= -779.392872138 zpe= -779.126836
N       -2.319557       1.590484        1.411686
C       -1.996543       0.834707        0.165285
C       -2.981148       -0.341455       0.165960
N       -2.500987       -1.600226       0.047900
C       -1.143283       -2.029618       0.361622
C       -0.642978       -1.302835       1.628837
N       0.689149        -1.230359       1.798160
C       1.273415        -0.579074       2.955699
C       0.996891        0.922484        2.924830
O       1.550927        1.520809        3.969737
H       1.368755        2.471151        3.939979
H       -2.224767       1.465817        -0.693341
H       -0.934908       0.607633        0.191743
O       -4.154960       -0.061589       0.267460
H       -3.327630       1.784582        1.430298
H       -1.768776       2.446908        1.494044
H       -3.231754       -2.299408       0.098354
H       -1.230162       -3.078616       0.661234
O       -1.425080       -0.818720       2.436792
H       1.311795        -1.591844       1.091647
```

```
O       0.349317        1.503274        2.086773
H       -2.073889       0.974069        2.206384
C       -0.208086       -1.958649       -0.848959
H       0.725684        -2.496183       -0.671172
H       0.027287        -0.930036       -1.130999
H       -0.695534       -2.438507       -1.696915
H       0.772913        -0.950883       3.855845
C       2.770050        -0.874648       3.032417
H       2.927576        -1.951747       3.094153
H       3.198361        -0.409893       3.918043
H       3.290115        -0.487009       2.152987

GAA_5.83_t-t_O1-ctggtgtt.xyz
31
0 eng= -779.388932372 zpe= -779.126312
N       -0.389981       -0.412492       -4.172063
C       -0.949417       0.745055        -3.499637
C       -1.958242       0.333178        -2.454263
N       -2.187407       -0.947057       -2.292344
C       -3.265491       -1.562094       -1.497223
C       -3.085639       -1.149853       -0.032895
N       -2.912600       -2.076950       0.899694
C       -2.471526       -1.697228       2.235446
C       -1.070107       -1.090400       2.119346
O       -0.673533       -0.610718       3.294771
H       0.214148        -0.235541       3.202336
H       -0.199049       1.346567        -2.979412
H       -1.474036       1.426161        -4.176263
O       -2.533627       1.252463        -1.789095
H       0.608429        -0.493818       -4.028993
H       -0.562968       -0.402543       -5.168808
H       -1.607825       -1.527938       -2.905167
O       -3.118621       0.056920        0.247510
H       -2.810637       -3.043774       0.625181
O       -0.420030       -1.062732       1.112338
H       -2.932903       0.861210        -0.887228
H       -4.208825       -1.103040       -1.813844
C       -3.297007       -3.055199       -1.775991
H       -4.111947       -3.526597       -1.225237
H       -2.350692       -3.531384       -1.506305
H       -3.476961       -3.230172       -2.837293
H       -3.135212       -0.915724       2.611747
C       -2.482753       -2.903442       3.168611
H       -1.810547       -3.684813       2.804677
H       -3.492629       -3.306786       3.247151
H       -2.149636       -2.601101       4.159942

GAA_8.51_NtA.xyz
31
0 eng= -779.39117555 zpe= -779.125292
```

N	-0.827251	0.215165	-3.838439
C	-1.627800	0.467180	-2.604666
C	-2.277920	-0.887071	-2.300522
N	-3.479312	-0.861981	-1.709593
C	-4.362140	-2.041532	-1.778640
C	-4.834465	-2.017870	-3.240066
N	-4.122123	-2.786919	-4.103260
C	-4.240210	-2.524090	-5.527984
C	-3.981350	-1.033009	-5.768501
O	-4.704675	-0.525749	-6.741109
H	-4.488520	0.411068	-6.857311
H	-0.957647	0.768245	-1.799060
H	-2.351812	1.252357	-2.814977
O	-1.673923	-1.892740	-2.634047
H	-0.177765	0.969099	-4.062327
H	-1.497778	0.056224	-4.615840
H	-3.957556	0.027396	-1.631974
O	-5.690433	-1.239985	-3.609496
H	-3.290163	-3.242819	-3.757081
O	-3.141971	-0.384455	-5.165300
H	-0.324362	-0.673204	-3.700393
H	-3.750705	-2.919646	-1.568749
C	-5.510255	-1.907414	-0.799181
H	-6.150889	-2.787464	-0.859346
H	-6.118181	-1.035549	-1.047694
H	-5.131887	-1.818072	0.219226
H	-3.399081	-3.031302	-6.012637
C	-5.547584	-3.032942	-6.127145
H	-5.630035	-4.101225	-5.928467
H	-5.561808	-2.873010	-7.204561
H	-6.396850	-2.517415	-5.681360

GAA_11.73_t-c_O1-ctggcgtt.xyz
31
0 eng= -779.38667368 zpe= -779.124065

N	-1.198318	-4.688034	-4.172403
C	-1.810312	-4.905309	-2.875310
C	-0.814805	-4.693875	-1.758595
N	0.406459	-4.356658	-2.084056
C	1.492514	-3.900382	-1.202396
C	1.789933	-4.950604	-0.134781
N	3.039918	-5.364685	0.073087
C	4.207271	-5.196879	-0.783158
C	3.946950	-5.837990	-2.150725
H	-2.643324	-4.225656	-2.672666
H	-2.203366	-5.917512	-2.747684
O	-1.228153	-4.867442	-0.565137
H	-1.648381	-3.947843	-4.694710
H	-1.203424	-5.522675	-4.744274
H	0.540641	-4.332910	-3.098576

H	2.347775	-3.760541	-1.856935
O	0.866598	-5.336616	0.594435
H	3.123785	-6.035793	0.830273
O	5.073250	-5.996018	-2.835768
H	4.868417	-6.372000	-3.704852
O	2.853498	-6.113384	-2.564721
H	-0.409781	-5.008266	0.086591
C	1.143867	-2.574364	-0.519116
H	0.961551	-1.808623	-1.273090
H	1.977542	-2.254832	0.109184
H	0.260807	-2.672558	0.113495
H	4.380494	-4.130586	-0.968355
C	5.435526	-5.772948	-0.083389
H	5.577786	-5.278770	0.878024
H	6.320831	-5.615195	-0.695402
H	5.317862	-6.846922	0.078450

GAA_12.12_NcB.xyz
31
0 eng= -779.38954722 zpe= -779.123916

N	-1.237933	0.953650	-2.739429
C	-1.457468	-0.447703	-2.279575
C	-2.812848	-0.424984	-1.554436
N	-3.062799	-1.366113	-0.625000
C	-2.080646	-2.233216	0.041078
C	-0.918422	-1.381950	0.593065
N	-1.278183	-0.303083	1.332374
C	-0.266200	0.593820	1.863747
C	0.570368	1.136639	0.706017
H	-1.557520	-1.095602	-3.152001
H	-0.601021	-0.754713	-1.683165
O	-3.613285	0.424361	-1.897709
H	-0.751205	1.459802	-1.979651
H	-2.173422	1.369287	-2.887681
H	-4.018395	-1.363373	-0.289645
O	0.241045	-1.639359	0.342482
H	-2.252962	-0.093089	1.479295
O	1.787808	1.476241	1.074905
H	2.264233	1.854395	0.321227
O	0.150121	1.303628	-0.420532
H	-0.683011	1.010111	-3.593096
H	-2.606349	-2.647644	0.905356
C	-1.579325	-3.380745	-0.822819
H	-0.924613	-4.023630	-0.235239
H	-0.997760	-3.025044	-1.674046
H	-2.424964	-3.965489	-1.184752
H	-0.794955	1.471984	2.250210
C	0.561806	-0.028305	2.987024
H	-0.111219	-0.363018	3.776315
H	1.250498	0.706269	3.401670

H 1.133137 -0.878405 2.616158

AAG_0.00_NcA.xyz
31
0 eng= -779.398733325 zpe= -779.132987

N	-2.626258	-2.740682	-2.874107
C	-2.285524	-1.277281	-2.931088
C	-2.157273	-0.884497	-1.449228
N	-1.009106	-0.298015	-1.032374
C	0.278483	-0.411544	-1.695264
C	0.524752	-1.862331	-2.143902
N	1.461653	-2.024503	-3.100187
C	1.720014	-3.327483	-3.663466
C	0.556578	-3.838955	-4.498791
O	-3.110085	-1.105070	-0.735064
H	-2.774000	-3.124727	-3.809694
H	-3.457533	-2.862319	-2.283880
H	-0.996962	-0.120122	-0.034214
O	-0.106263	-2.803308	-1.686332
H	1.924967	-1.218525	-3.492319
H	2.615703	-3.296218	-4.283213
H	1.891875	-4.059923	-2.869793
O	0.892206	-4.961226	-5.115011
H	0.145931	-5.289975	-5.637266
O	-0.528777	-3.317625	-4.579137
H	-1.825389	-3.236002	-2.446937
H	-1.362864	-1.213537	-3.503921
C	-3.407577	-0.502761	-3.601750
H	-3.557599	-0.846481	-4.627114
H	-3.146869	0.555814	-3.635032
H	-4.334760	-0.604456	-3.035176
H	0.296198	0.230173	-2.583648
C	1.379351	0.038284	-0.732921
H	2.355215	0.009057	-1.217420
H	1.407198	-0.616739	0.140465
H	1.190324	1.062048	-0.407621

AAG_5.99_NcB.xyz
31
0 eng= -779.395738546 zpe= -779.130704

C	-2.328629	-0.036253	0.933947
H	-1.430707	-0.488160	1.353691
C	-2.383615	-0.078993	-0.604808
O	-2.888849	0.863586	-1.184125
N	-2.267731	1.427981	1.251327
H	-2.814176	1.914736	0.521339
H	-1.282095	1.734283	1.195137
N	-1.931124	-1.193179	-1.213327
H	-2.100574	-1.234678	-2.212062
C	-1.105767	-2.227811	-0.590148

H	-1.629013	-2.665697	0.263566
C	0.187129	-1.636501	-0.013072
O	0.573015	-1.934977	1.096555
N	0.858736	-0.759512	-0.807617
H	0.446348	-0.442187	-1.671174
C	2.000424	-0.070718	-0.270739
H	2.686807	-0.775942	0.201925
H	2.543528	0.442409	-1.066030
C	1.605606	0.957092	0.774942
O	2.665822	1.447720	1.384890
H	2.401681	2.107451	2.042723
O	0.469319	1.301829	1.015325
H	-2.641120	1.639047	2.177368
C	-0.798903	-3.322877	-1.606437
H	-0.173773	-4.086270	-1.143635
H	-0.264080	-2.917334	-2.468627
H	-1.723072	-3.791202	-1.947889
C	-3.597559	-0.653876	1.511282
H	-3.651155	-1.708097	1.237663
H	-4.485228	-0.155566	1.115141
H	-3.596981	-0.593753	2.601232

AAG_6.18_NtA.xyz
31
0 eng= -779.396389128 zpe= -779.130632

N	-0.555011	1.408608	-2.906656
C	-1.928010	0.876084	-2.596675
C	-1.988259	0.980394	-1.060272
N	-2.727513	0.073551	-0.409564
C	-2.527062	-0.147322	1.033204
C	-1.198846	-0.915122	1.088096
N	-0.087892	-0.157983	1.305086
C	1.188121	-0.752432	1.017669
C	1.370933	-1.017837	-0.467453
H	-2.642208	1.589645	-3.014999
O	-1.349739	1.873808	-0.525045
H	-0.411962	2.246370	-2.324307
H	-0.428138	1.623262	-3.895803
H	-3.083883	-0.723211	-0.920695
H	-2.441246	0.832422	1.504125
O	-1.143568	-2.100265	0.840376
H	-0.182355	0.847566	1.274781
H	1.996798	-0.095689	1.344507
H	1.296425	-1.702573	1.542130
O	2.383180	-1.831476	-0.691933
H	2.489075	-1.982566	-1.642226
O	0.697350	-0.527361	-1.352395
H	0.142103	0.711046	-2.588658
C	-3.673522	-0.950919	1.613765
H	-3.514900	-1.101923	2.681745

H -3.721496 -1.934478 1.142929
H -4.616802 -0.423492 1.470965
C -2.104863 -0.508816 -3.193821
H -1.440083 -1.229176 -2.711595
H -1.905689 -0.497319 -4.267149
H -3.138677 -0.836923 -3.075708

AAG_12.57_OtA.xyz
31
0 eng= -779.390385689 zpe= -779.1282
N -1.811139 0.857028 -4.849136
C -1.190636 -0.420943 -4.525096
C -1.260386 -0.651876 -3.025377
N -1.706233 0.329263 -2.274932
C -2.106875 0.294108 -0.857713
C -0.995317 -0.243036 0.037712
N -0.758842 0.403010 1.165658
C 0.189557 -0.085809 2.150691
C 0.165549 0.879696 3.317325
O 1.004203 0.502622 4.270060
H 0.964605 1.135976 5.002255
H -1.734147 -1.264937 -4.964003
O -0.860466 -1.775283 -2.583078
H -2.686828 0.749488 -5.346215
H -1.202644 1.436309 -5.415299
H -1.977169 1.134577 -2.849412
H -2.315169 1.330156 -0.588067
O -0.395603 -1.291535 -0.252337
H -1.240511 1.264000 1.408231
H -0.080909 -1.088120 2.491448
H 1.197893 -0.139339 1.733265
O -0.534579 1.853511 3.347493
H -0.677106 -1.722264 -1.533008
C -3.365037 -0.560340 -0.663459
H -3.664863 -0.548690 0.385562
H -3.179274 -1.594484 -0.959101
H -4.181281 -0.158011 -1.263366
C 0.274588 -0.493440 -4.964848
H 0.837578 0.345690 -4.547828
H 0.326450 -0.445532 -6.052618
H 0.733308 -1.424385 -4.634742

AAG_13.27_t-t_N-gtgttgtt.xyz
31
0 eng= -779.393279522 zpe= -779.127932
N -0.683095 0.516891 -3.174972
C -2.093672 0.699841 -2.660165
C -1.856753 0.568176 -1.144886
N -2.355665 -0.515345 -0.493905
C -1.429972 -1.106170 0.472604

C	-0.115518	-1.343209	-0.298691
N	1.031644	-1.137061	0.363623
C	2.309505	-1.319986	-0.282097
C	2.594985	-0.291403	-1.366085
H	-2.373651	1.732775	-2.870150
O	-1.075114	1.342749	-0.639593
H	-0.039309	1.164104	-2.694582
H	-0.616364	0.641883	-4.186095
H	-2.861106	-1.190711	-1.051333
H	-1.267545	-0.390997	1.280164
O	-0.143425	-1.708325	-1.471477
H	1.000121	-0.698785	1.272021
H	3.107141	-1.281791	0.458779
H	2.349902	-2.302308	-0.761218
O	3.865164	-0.375397	-1.742542
H	4.041683	0.269526	-2.442592
O	1.798904	0.473188	-1.846764
H	-0.340010	-0.425987	-2.905845
C	-1.980857	-2.421472	1.006936
H	-1.289871	-2.856670	1.729525
H	-2.119051	-3.137322	0.193007
H	-2.937176	-2.251045	1.502117
C	-3.023980	-0.291803	-3.328603
H	-2.665560	-1.317799	-3.208152
H	-3.114983	-0.082658	-4.396020
H	-4.022591	-0.208016	-2.896278

AAG_13.32_OtA.xyz
31
0 eng= -779.389975149 zpe= -779.127914

N	-0.986298	-0.015308	-4.635707
C	-1.831475	0.870020	-3.845068
C	-1.378796	0.837929	-2.397372
N	-0.455714	-0.033556	-2.057282
C	-0.092528	-0.462060	-0.694251
C	0.425925	0.728939	0.116908
N	1.557499	0.610422	0.783802
C	2.070462	1.676822	1.625596
C	3.371536	1.187707	2.226683
O	-1.926010	1.641324	-1.578562
H	-0.361263	0.493330	-5.249219
H	-1.534876	-0.646931	-5.207010
H	-0.125550	-0.574284	-2.862862
H	-1.020169	-0.756817	-0.187892
O	-0.255695	1.767821	0.171849
H	2.128191	-0.228501	0.742330
H	2.246757	2.585248	1.044614
H	1.362042	1.925483	2.419447
O	3.910913	2.102741	3.016935
H	4.738835	1.760992	3.386501

O	3.831840	0.103425	1.997367
H	-1.315632	1.745386	-0.702124
C	0.841135	-1.658950	-0.785999
H	1.037438	-2.069894	0.204848
H	1.787842	-1.392082	-1.261370
H	0.369633	-2.449031	-1.371629
H	-1.741686	1.915483	-4.156726
C	-3.308801	0.463920	-3.889013
H	-3.423435	-0.579896	-3.585348
H	-3.679631	0.576379	-4.908037
H	-3.905701	1.092002	-3.228874

AAG_14.88_OtB.xyz
31
0 eng= -779.389991327 zpe= -779.12732

N	0.436874	1.902126	-2.608852
C	0.393916	2.405141	-1.242181
C	-0.378341	1.431050	-0.369505
N	-0.991142	0.432583	-0.960086
C	-1.585320	-0.774385	-0.360316
C	-2.594618	-0.455138	0.732542
N	-3.713199	-1.178236	0.760735
C	-4.495852	-1.229515	1.980172
C	-3.719979	-1.985588	3.053799
O	-4.398937	-2.004225	4.194321
H	-3.890762	-2.489100	4.861166
O	-0.402945	1.659391	0.882872
H	1.359760	1.582618	-2.876958
H	0.147033	2.606507	-3.276915
H	-0.820090	0.449977	-1.971347
O	-2.345167	0.382268	1.608089
H	-3.828135	-1.921794	0.087190
H	-4.704970	-0.220246	2.335907
H	-5.445551	-1.728638	1.790559
O	-2.648408	-2.489610	2.873580
H	-1.177156	1.108179	1.347740
H	1.390191	2.459085	-0.789577
C	-0.269045	3.782409	-1.144451
H	-1.261490	3.758270	-1.602126
H	0.343499	4.514181	-1.671517
H	-0.367807	4.093976	-0.105541
H	-2.090093	-1.286280	-1.180900
C	-0.506127	-1.683068	0.245039
H	0.050241	-1.149180	1.017696
H	0.181241	-2.009996	-0.535168
H	-0.970906	-2.553424	0.711160

AAG_15.56_t-t_N-gtgttgtt.xyz
31
0 eng= -779.391990127 zpe= -779.127062

N	0.790266	2.119683	-0.292978
C	-0.602360	1.893095	-0.844497
C	-0.422462	0.518769	-1.509116
N	-0.878276	-0.566679	-0.827637
C	-0.020813	-1.759521	-0.862225
C	1.380071	-1.261661	-0.440112
N	2.446076	-1.697856	-1.123122
C	3.774442	-1.242018	-0.791978
C	4.013249	0.228500	-1.100276
O	0.273011	0.452424	-2.496289
H	0.874339	3.018491	0.184026
H	1.484314	2.066878	-1.058736
H	-1.329433	-0.388291	0.057927
O	1.483235	-0.480372	0.503707
H	2.303989	-2.226284	-1.970572
H	3.957997	-1.370621	0.278638
H	4.513585	-1.837357	-1.326585
O	5.306708	0.504483	-0.993662
H	5.456030	1.444280	-1.171831
O	3.172466	1.046591	-1.373966
H	1.066891	1.341133	0.338895
H	-1.277720	1.860473	0.011767
C	-0.961532	2.989307	-1.824847
H	-0.276958	2.970887	-2.674443
H	-0.935443	3.970866	-1.347657
H	-1.972156	2.822090	-2.198477
H	-0.356270	-2.397693	-0.040702
C	-0.108643	-2.538627	-2.167727
H	0.443932	-3.477589	-2.086159
H	0.263427	-1.953840	-3.008790
H	-1.150926	-2.789626	-2.363367

AAG_16.07_c-t_N-gcggtgtt.xyz
31
0 eng= -779.392269563 zpe= -779.126867

N	-2.981871	0.540629	-0.231794
C	-3.090460	-0.909765	-0.620009
C	-1.690435	-1.236737	-1.168053
N	-0.985050	-2.241114	-0.591539
C	-1.177093	-2.778063	0.760507
C	-1.087882	-1.614962	1.764163
N	0.146648	-1.203723	2.124182
C	0.317461	-0.028369	2.941874
C	-0.067096	1.250922	2.220764
O	0.332820	2.309783	2.903351
H	0.061214	3.119448	2.446277
H	-3.346180	-1.445159	0.289186
O	-1.291505	-0.582919	-2.106802
H	-3.887382	0.925170	0.042923
H	-2.617855	1.064275	-1.038438

H	-0.079259	-2.390577	-1.023522
H	-2.190212	-3.171843	0.857169
O	-2.096751	-1.045775	2.151385
H	0.962928	-1.668881	1.760110
H	-0.315038	-0.090043	3.832797
H	1.351772	0.048786	3.274986
O	-0.674310	1.313842	1.177883
H	-2.303537	0.667705	0.543579
C	-0.185110	-3.907653	0.998239
H	-0.273535	-4.282415	2.017964
H	0.848052	-3.587033	0.835269
H	-0.389317	-4.726968	0.308251
C	-4.138628	-1.080785	-1.707056
H	-4.195636	-2.132907	-1.988806
H	-3.871388	-0.506352	-2.595414
H	-5.123901	-0.777102	-1.347575

AAG_16.83_NtB.xyz
31
0 eng= -779.392368572 zpe= -779.126577

N	-2.568724	-0.453502	-3.566966
C	-3.057448	-0.324419	-2.149786
C	-2.286021	-1.438030	-1.414572
N	-2.889800	-2.015066	-0.367657
C	-2.394603	-3.291886	0.173519
C	-2.883553	-4.325991	-0.849916
N	-1.990815	-4.650774	-1.831601
C	-2.521226	-5.254723	-3.021253
C	-3.360767	-4.263307	-3.826950
O	-1.174343	-1.731488	-1.826281
H	-1.552247	-0.618755	-3.527316
H	-2.783546	0.366455	-4.134423
H	-3.867616	-1.821391	-0.196864
H	-1.305510	-3.236429	0.190418
O	-4.022347	-4.737377	-0.844052
H	-1.157667	-4.082358	-1.910956
H	-1.707008	-5.606425	-3.659959
H	-3.140486	-6.113260	-2.750491
O	-4.101488	-4.755615	-4.805055
H	-4.090744	-5.720857	-4.826340
O	-3.334006	-3.070441	-3.631308
H	-2.988368	-1.306191	-3.980590
C	-2.965785	-3.545249	1.554520
H	-2.578572	-4.485986	1.946519
H	-4.053030	-3.627967	1.505269
H	-2.684424	-2.740626	2.233988
H	-2.689447	0.635802	-1.779514
C	-4.573880	-0.393738	-2.107726
H	-4.928875	-1.380661	-2.413052
H	-5.014099	0.361510	-2.761761

H	-4.931438	-0.175384	-1.100337

GGG_0.00_NcA.xyz
25
0 eng= -700.778106755 zpe= -700.568451

C	-5.434338	-7.359985	2.867610
H	-6.152406	-6.750526	2.319438
H	-5.235595	-6.918635	3.840319
C	-4.233829	-7.623284	1.947058
O	-4.453199	-8.227908	0.923457
N	-6.034320	-8.707262	3.100316
H	-5.342179	-9.260030	3.635485
H	-6.199099	-9.158567	2.192944
N	-3.018036	-7.159100	2.325574
H	-2.275854	-7.422469	1.689325
C	-2.662948	-6.862215	3.695205
H	-3.062481	-5.893636	4.011462
H	-1.575576	-6.792704	3.759043
C	-3.146398	-7.958545	4.654688
O	-3.537862	-9.041634	4.248251
N	-3.162955	-7.629453	5.959705
H	-2.874706	-6.707789	6.252403
C	-3.718736	-8.536593	6.935690
H	-3.265902	-9.527392	6.840307
H	-3.512092	-8.173868	7.942040
C	-5.221061	-8.711619	6.769776
O	-5.714264	-9.413224	7.776837
H	-6.669090	-9.531263	7.666406
O	-5.875759	-8.294985	5.845118
H	-6.892020	-8.655290	3.653213

GGG_7.57_NcB.xyz
25
0 eng= -700.774526634 zpe= -700.565567

C	-2.300653	-0.074132	0.947595
H	-1.419699	-0.545558	1.378645
H	-3.204115	-0.548005	1.336454
C	-2.379226	-0.082234	-0.588417
O	-2.868506	0.885323	-1.135527
N	-2.303584	1.367470	1.326490
H	-2.834360	1.867739	0.592372
H	-1.325434	1.700934	1.310313
N	-1.948469	-1.187015	-1.227080
H	-2.128643	-1.201235	-2.223116
C	-1.144362	-2.237766	-0.615840
H	-0.926626	-2.984419	-1.380158
H	-1.674943	-2.740462	0.194217
C	0.164168	-1.695233	-0.031543
O	0.522393	-1.988693	1.087362
N	0.862856	-0.838924	-0.823741

H	0.470881	-0.527335	-1.698908
C	1.987544	-0.138496	-0.265754
H	2.657738	-0.834553	0.242879
H	2.556916	0.357245	-1.053442
C	1.557687	0.908589	0.747128
O	2.598925	1.452103	1.343128
H	2.312965	2.118668	1.984678
O	0.408279	1.218327	0.976404
H	-2.711667	1.539956	2.245110

GGG_7.69_OtA.xyz
25
0 eng= -700.771273417 zpe= -700.565523

C	-1.864384	0.840531	-3.883454
H	-1.656256	1.837387	-4.281190
H	-2.953812	0.753685	-3.830861
C	-1.374659	0.843195	-2.453987
O	-1.865683	1.698679	-1.656827
N	-1.232547	-0.217255	-4.649718
H	-0.726963	0.137222	-5.451449
H	-1.896408	-0.905825	-4.980613
N	-0.459510	-0.040761	-2.124871
H	-0.200121	-0.631279	-2.920928
C	-0.024303	-0.409345	-0.776743
H	0.745137	-1.172349	-0.878433
H	-0.867808	-0.847634	-0.231336
C	0.469043	0.771198	0.047221
O	-0.182757	1.828159	0.079228
N	1.561137	0.601914	0.767470
H	2.106614	-0.255131	0.735496
C	2.058809	1.628230	1.667643
H	2.278221	2.551305	1.126079
H	1.322516	1.862315	2.440275
C	3.323874	1.090568	2.304923
O	3.844559	1.966646	3.149261
H	4.650691	1.597837	3.540784
O	3.771689	0.005944	2.055503
H	-1.236499	1.815641	-0.792981

GGG_10.37_NtA.xyz
25
0 eng= -700.774176205 zpe= -700.564501

C	-0.876831	1.959166	-0.877339
H	-1.904504	2.127404	-0.559975
H	-0.666707	2.506820	-1.796487
C	-0.528926	0.475671	-1.034200
O	0.606464	0.124879	-0.761794
N	0.042036	2.438394	0.196609
H	0.098427	3.455041	0.258984
H	0.969207	2.029532	0.004818

N	-1.511093	-0.337137	-1.444308
H	-2.455401	0.023422	-1.479987
C	-1.380316	-1.780680	-1.224162
H	-0.386226	-2.099458	-1.533136
H	-2.140281	-2.300289	-1.802267
C	-1.654883	-1.959252	0.267990
O	-2.785045	-1.942603	0.701624
N	-0.555685	-1.976178	1.074427
H	0.344607	-1.771816	0.663775
C	-0.761882	-1.720352	2.473734
H	-1.526689	-2.385467	2.875944
H	0.159860	-1.892078	3.033284
C	-1.205609	-0.288223	2.729720
O	-1.715685	-0.149013	3.935424
H	-1.985896	0.768589	4.085411
O	-1.082971	0.631749	1.943096
H	-0.275083	2.028613	1.094355

GGG_12.69_OtB.xyz
25
0 eng= -700.769582963 zpe= -700.563617

C	-3.941594	0.682969	0.300476
H	-4.724564	1.128887	-0.318742
H	-3.393233	1.517220	0.748307
C	-2.978511	0.012142	-0.651556
O	-2.435618	0.728535	-1.546994
N	-4.455945	-0.261060	1.274865
H	-5.463601	-0.345349	1.234495
H	-4.202076	-0.015828	2.223349
N	-2.770901	-1.275471	-0.495299
H	-3.317839	-1.649276	0.286862
C	-1.719135	-2.099695	-1.092535
H	-1.882534	-3.123347	-0.760275
H	-0.739826	-1.773408	-0.721972
C	-1.640211	-2.014966	-2.607080
O	-1.761342	-0.928721	-3.186276
N	-1.368616	-3.133926	-3.271302
H	-1.186127	-3.980776	-2.752749
C	-0.916600	-3.055322	-4.647546
H	-0.887401	-4.053442	-5.083080
H	-1.604779	-2.445471	-5.233846
C	0.472602	-2.426016	-4.695548
O	0.891739	-2.325130	-5.951278
H	1.773818	-1.925239	-5.963201
O	1.082674	-2.079716	-3.725931
H	-2.035302	0.114846	-2.322265

GGG_16.39_t-t_N-gtgttgtt.xyz
25
0 eng= -700.771938061 zpe= -700.56221

C	-1.884384	-9.093507	-0.796565
H	-1.637558	-8.989291	-1.851137
H	-2.002626	-8.120053	-0.323695
C	-0.907657	-10.041556	-0.099809
O	-0.735258	-11.131986	-0.591477
N	-3.180700	-9.846580	-0.677350
H	-3.074675	-10.798760	-1.064330
H	-3.959805	-9.372582	-1.135787
N	-0.473720	-9.702754	1.144869
H	-0.819289	-8.836238	1.532043
C	-0.425953	-10.826064	2.070014
H	-0.180967	-10.461699	3.067709
H	0.333866	-11.539980	1.754884
C	-1.832120	-11.448868	2.078103
O	-2.821956	-10.728610	1.979295
N	-1.918909	-12.782394	2.164872
H	-1.076797	-13.335118	2.104993
C	-3.199261	-13.447170	2.107182
H	-3.079812	-14.501509	2.353057
H	-3.882439	-13.011974	2.842093
C	-3.879033	-13.329758	0.750647
O	-4.919630	-14.149910	0.696444
H	-5.361212	-14.064123	-0.160895
O	-3.556046	-12.589742	-0.143439
H	-3.397814	-10.015885	0.326017

GGG_17.31_c-t_N-gcggtgtt.xyz
25
0 eng= -700.77124273 zpe= -700.561859

C	-3.006195	-1.028862	-0.595934
H	-2.658064	-1.867115	-0.002205
H	-4.066852	-1.121818	-0.827700
C	-2.269716	-0.766528	-1.915586
O	-2.605513	0.205887	-2.552185
N	-2.808570	0.218496	0.204720
H	-3.366071	0.221412	1.060098
H	-3.066181	1.026099	-0.376958
N	-1.293446	-1.627917	-2.292326
H	-0.872220	-1.393975	-3.183219
C	-0.566401	-2.523740	-1.396095
H	0.195317	-3.034921	-1.984450
H	-1.222465	-3.283694	-0.971337
C	0.047101	-1.720617	-0.244869
O	-0.534235	-1.599354	0.821182
N	1.206886	-1.083152	-0.510495
H	1.641406	-1.179597	-1.415230
C	1.776856	-0.164038	0.444226
H	1.853989	-0.637192	1.428093
H	2.781307	0.120989	0.134192
C	0.939378	1.089872	0.625290

O	1.615591	2.010747	1.287444
H	1.063382	2.795438	1.422204
O	-0.198802	1.235184	0.243810
H	-1.805328	0.333639	0.449894

GGG_17.86_OtB.xyz
25
0 eng= -700.768892396 zpe= -700.561647

C	-1.609265	6.621538	14.152199
H	-0.927390	6.329847	13.348393
H	-1.366053	7.657098	14.406090
C	-1.222177	5.780570	15.346342
O	-0.007147	5.917570	15.739462
N	-3.004953	6.422445	13.816528
H	-3.128031	6.040150	12.887716
H	-3.546272	7.274839	13.880365
N	-2.139134	5.030964	15.880666
H	-3.025200	5.101091	15.368274
C	-1.971114	4.129148	17.004593
H	-2.903656	3.583316	17.149611
H	-1.766863	4.706452	17.911014
C	-0.817152	3.158647	16.718574
O	-0.379745	3.023945	15.600324
N	-0.288078	2.548063	17.809751
H	-0.611575	2.830355	18.723751
C	1.080223	2.098912	17.702817
H	1.390466	1.590797	18.615432
H	1.192224	1.398904	16.872698
C	1.998136	3.286983	17.444464
O	3.265930	2.934264	17.444999
H	3.824209	3.705330	17.261244
O	1.614616	4.421807	17.263789
H	0.389754	5.277058	16.382638

GGG_18.10_NcC.xyz
25
0 eng= -700.770870195 zpe= -700.561556

C	-2.703159	-2.023850	-2.417775
H	-3.607743	-2.479158	-2.820601
H	-2.056774	-2.773626	-1.973339
C	-3.114785	-0.854460	-1.515320
O	-3.684429	0.066948	-2.057095
N	-1.959447	-1.363295	-3.532873
H	-1.066850	-1.032992	-3.123513
H	-2.507838	-0.546409	-3.836407
N	-2.850076	-0.918385	-0.190628
H	-3.161565	-0.092108	0.307348
C	-1.757056	-1.665455	0.400754
H	-1.888837	-2.740851	0.250128
H	-1.778721	-1.499637	1.477179

C	-0.403780	-1.263777	-0.202795
O	-0.308524	-0.993714	-1.400926
N	0.638422	-1.270449	0.622100
H	0.538160	-1.462373	1.613973
C	1.983312	-0.976348	0.169083
H	2.316378	-1.698117	-0.580819
H	2.038680	0.017876	-0.281301
C	2.899427	-1.039399	1.372984
O	4.156386	-0.787005	1.032829
H	4.715125	-0.830966	1.823077
O	2.515572	-1.287989	2.481465
H	-1.784328	-1.978514	-4.328198

GGG_18.69_t-c_O1-ctggcgtt.xyz
25
0 eng= -700.768169602 zpe= -700.561332

C	-3.928149	1.095160	-0.140537
H	-3.599173	2.131800	-0.028107
H	-4.695327	0.933414	0.622919
C	-2.755448	0.232175	0.255173
O	-2.241917	0.417078	1.409894
N	-4.377565	0.776691	-1.481753
H	-4.288220	1.561453	-2.114130
H	-5.338557	0.461777	-1.505566
N	-2.327140	-0.658256	-0.599847
H	-2.799496	-0.611870	-1.506042
C	-1.342638	-1.703582	-0.331149
H	-1.324100	-2.365204	-1.190724
H	-1.663127	-2.277243	0.544696
C	0.011610	-1.100446	0.019039
O	0.066507	-0.339209	0.989725
N	1.106615	-1.434054	-0.665995
H	1.950680	-0.982727	-0.331891
C	1.178179	-2.156287	-1.920046
H	2.224956	-2.264434	-2.199492
H	0.776973	-3.170474	-1.829968
C	0.425664	-1.445362	-3.038567
O	0.833299	-1.872484	-4.223638
H	0.322778	-1.436115	-4.922410
O	-0.447297	-0.641563	-2.852838
H	-1.260100	0.062118	1.435430

AGG_0.00_NcA.xyz
28
0 eng= -740.089790196 zpe= -739.852264

N	0.001218	-1.517503	-2.147059
C	-0.967488	-0.406403	-2.441235
C	-2.316814	-1.135674	-2.578218
N	-3.356886	-0.724094	-1.810223
C	-3.204272	-0.012452	-0.561371

C	-2.114415	-0.640234	0.318400
N	-1.669477	0.123988	1.334037
C	-0.551441	-0.309239	2.137483
C	0.745688	-0.347276	1.343194
O	1.767055	-0.604073	2.144543
H	2.589902	-0.635911	1.634964
H	-0.893494	0.276659	-1.597351
O	-2.381311	-2.028094	-3.392721
H	0.959717	-1.168675	-2.081491
H	-0.086304	-2.230914	-2.880592
H	-4.186508	-1.291673	-1.931432
H	-4.155081	-0.052146	-0.026868
H	-2.977758	1.045197	-0.728220
O	-1.648882	-1.742838	0.077177
H	-2.045469	1.049990	1.472307
H	-0.425344	0.355245	2.991699
H	-0.723545	-1.317623	2.523807
O	0.839967	-0.189119	0.150265
H	-0.256159	-1.929969	-1.234682
C	-0.596965	0.282029	-3.744819
H	0.393871	0.735421	-3.678520
H	-1.317564	1.074027	-3.952725
H	-0.628178	-0.428287	-4.572650

AGG_8.54_NcB.xyz
28
0 eng= -740.085929959 zpe= -739.849012

N	-2.275252	1.400605	1.243618
C	-2.321844	-0.063540	0.922396
C	-2.359762	-0.105444	-0.616318
N	-1.901629	-1.222513	-1.218727
C	-1.088064	-2.241860	-0.570333
C	0.214033	-1.673497	0.002707
N	0.909412	-0.834790	-0.811206
C	2.033698	-0.120639	-0.269452
C	1.603014	0.938265	0.730073
H	-1.425072	-0.508617	1.351984
O	-2.859493	0.833540	-1.203487
H	-2.821597	1.886319	0.512985
H	-1.292938	1.717155	1.192299
H	-2.065774	-1.266153	-2.216592
H	-0.859842	-3.012495	-1.307506
H	-1.614583	-2.722578	0.255807
O	0.574674	-1.939660	1.127559
H	0.508884	-0.536413	-1.687170
H	2.710394	-0.805776	0.245053
H	2.595522	0.367421	-1.067263
O	2.643542	1.479956	1.330058
H	2.356209	2.156062	1.960856
O	0.454921	1.260285	0.944894

H	-2.655833	1.605669	2.168276
C	-3.593667	-0.691309	1.482841
H	-3.643116	-1.742119	1.195371
H	-4.479295	-0.191023	1.084355
H	-3.603422	-0.644465	2.573255

AGG_10.32_NtA.xyz
28
0 eng= -740.08596573 zpe= -739.848333

N	0.022357	2.401642	0.238968
C	-0.863676	1.973899	-0.896951
C	-0.565651	0.474543	-1.019971
N	-1.581065	-0.314903	-1.399133
C	-1.479532	-1.761194	-1.186059
C	-1.710624	-1.946569	0.311930
N	-0.586808	-1.999166	1.081685
C	-0.745018	-1.774341	2.492337
C	-1.174321	-0.346879	2.791346
H	-1.892717	2.153768	-0.582407
O	0.565604	0.090402	-0.773293
H	0.076328	3.416514	0.333573
H	0.956016	2.005933	0.056580
H	-2.516544	0.069886	-1.403228
H	-0.503088	-2.102386	-1.526238
H	-2.268168	-2.260385	-1.743435
O	-2.825900	-1.913960	0.782299
H	0.301795	-1.793267	0.646440
H	-1.498783	-2.445528	2.905548
H	0.194292	-1.961749	3.016462
O	-1.641832	-0.227273	4.016885
H	-1.902667	0.688750	4.190707
O	-1.076173	0.585640	2.017549
H	-0.316302	1.962746	1.115152
C	-0.505958	2.731591	-2.166671
H	0.533749	2.544527	-2.444337
H	-0.665247	3.804688	-2.044752
H	-1.141519	2.388836	-2.983724

AGG_11.04_NtA.xyz
28
0 eng= -740.085849594 zpe= -739.84806

N	-0.946778	-0.139860	-1.834979
C	-2.040092	0.724320	-2.403759
C	-2.036561	1.935383	-1.452199
N	-3.204515	2.556488	-1.235607
C	-3.338388	3.420047	-0.059749
C	-3.445880	2.451947	1.116661
N	-2.287381	2.206351	1.788858
C	-2.247884	1.049111	2.639793
C	-2.365575	-0.242272	1.846459

O	-2.658826	-1.262882	2.626154
H	-2.720043	-2.077993	2.107526
H	-1.695410	1.061327	-3.384500
O	-0.976735	2.254098	-0.937365
H	-0.151870	0.475268	-1.609556
H	-0.662291	-0.885799	-2.470189
H	-4.055169	2.118247	-1.559475
H	-4.255715	3.997360	-0.144245
H	-2.478334	4.085206	-0.005415
O	-4.473970	1.851384	1.340853
H	-1.425571	2.567416	1.404670
H	-1.308445	1.017904	3.195464
H	-3.063727	1.073716	3.363246
O	-2.184613	-0.344107	0.648689
H	-1.278032	-0.530920	-0.933922
C	-3.335657	-0.061219	-2.504254
H	-3.707688	-0.328792	-1.512487
H	-3.193665	-0.969718	-3.092644
H	-4.090454	0.529150	-3.026098

AGG_13.41_t-t_N-gtgttgtt.xyz
28
0 eng= -740.084694759 zpe= -739.847158

N	0.157462	1.916146	0.053048
C	-1.008446	2.090886	-0.900801
C	-1.644201	0.694850	-0.836177
N	-2.712514	0.535227	-0.005568
C	-2.715701	-0.719474	0.731621
C	-1.400465	-0.768986	1.523336
N	-0.764362	-1.945913	1.599959
C	0.516470	-2.049351	2.257017
C	1.628022	-1.308185	1.528479
H	-1.649891	2.863007	-0.472724
O	-1.068199	-0.228734	-1.360466
H	0.719980	2.765092	0.129702
H	0.757161	1.133688	-0.259451
H	-3.024234	1.345899	0.510035
H	-2.810731	-1.557369	0.042458
H	-3.556728	-0.729056	1.424874
O	-0.953590	0.254269	2.034134
H	-1.096405	-2.721685	1.046693
H	0.458568	-1.623974	3.262823
H	0.797115	-3.097024	2.358241
O	2.801801	-1.620226	2.060980
H	3.506620	-1.137254	1.605613
O	1.486096	-0.530353	0.619267
H	-0.171490	1.611075	0.991451
C	-0.504636	2.453958	-2.281043
H	0.110096	1.645188	-2.679222
H	0.067965	3.383220	-2.262054

H   -1.354792   2.594447   -2.949126

AGG_15.77_OtA.xyz
28
0 eng= -740.08028054 zpe= -739.846259

N	-1.115740	-0.119093	-4.643686
C	-1.854724	0.878601	-3.881310
C	-1.351707	0.890552	-2.449514
N	-0.463145	-0.014275	-2.104248
C	-0.046204	-0.378811	-0.749571
C	0.491758	0.794939	0.055898
N	1.583059	0.600091	0.772035
C	2.117016	1.622952	1.654763
C	3.345504	1.042500	2.324481
H	-1.678395	1.891832	-4.256350
O	-1.819721	1.767284	-1.657708
H	-0.506527	0.296274	-5.337841
H	-1.734969	-0.764093	-5.120202
H	-0.207549	-0.610821	-2.897716
H	0.691473	-1.174241	-0.836584
H	-0.908077	-0.772266	-0.198821
O	-0.125405	1.872051	0.076566
H	2.097537	-0.276025	0.752501
H	2.388467	2.521047	1.094776
H	1.381053	1.911855	2.409009
O	3.893992	1.913081	3.157248
H	4.673937	1.514724	3.572048
O	3.746174	-0.067307	2.107805
H	-1.190211	1.876668	-0.800034
C	-3.363821	0.612520	-3.859616
H	-3.566056	-0.398447	-3.496362
H	-3.755970	0.706797	-4.872341
H	-3.877217	1.327212	-3.217718

AGG_16.63_t-t_N-gtgttgtt.xyz
28
0 eng= -740.083580474 zpe= -739.845929

N	-0.989299	-0.221829	-3.276897
C	-2.052067	0.669779	-2.672413
C	-1.773608	0.455493	-1.174838
N	-2.694914	-0.202681	-0.420250
C	-2.098753	-1.155834	0.505112
C	-1.186227	-2.071330	-0.329495
N	-0.051823	-2.497971	0.241641
C	0.889556	-3.310443	-0.492190
C	1.570349	-2.565743	-1.631730
H	-1.781433	1.696247	-2.922873
O	-0.660593	0.715333	-0.777024
H	-0.064278	0.001822	-2.877870
H	-0.958367	-0.158288	-4.295423

H	-3.526268	-0.526269	-0.892707
H	-2.886877	-1.750788	0.967159
H	-1.544763	-0.632482	1.283283
O	-1.499347	-2.368763	-1.478724
H	0.224956	-2.112955	1.132238
H	1.651409	-3.695750	0.184071
H	0.377318	-4.168220	-0.937134
O	2.592884	-3.278465	-2.087924
H	3.013893	-2.810590	-2.823567
O	1.231607	-1.504253	-2.087716
H	-1.159526	-1.203241	-2.982487
C	-3.417522	0.290662	-3.208574
H	-3.624621	-0.772223	-3.056506
H	-3.490883	0.505740	-4.275962
H	-4.186309	0.880996	-2.706832

AGG_16.83_OtA.xyz
28
0 eng= -740.080148279 zpe= -739.845853

N	-0.514754	1.743057	-5.073246
C	-1.058528	0.803850	-4.101011
C	-0.102899	-0.364956	-3.942193
N	0.930464	-0.426079	-4.751670
C	2.103588	-1.294107	-4.637321
C	1.762906	-2.775865	-4.576119
N	2.510384	-3.605450	-5.279687
C	2.333960	-5.045884	-5.211766
C	3.363056	-5.669158	-6.132198
H	-1.133392	1.242210	-3.099639
O	-0.362056	-1.230465	-3.049031
H	-0.200683	2.606499	-4.647049
H	-1.194395	1.979350	-5.786482
H	0.951661	0.375257	-5.390698
H	2.643209	-1.053319	-3.714366
H	2.757123	-1.071945	-5.478858
O	0.851594	-3.178888	-3.835536
H	3.251062	-3.282031	-5.895718
H	2.475906	-5.410836	-4.191594
H	1.328873	-5.332023	-5.530341
O	3.271284	-6.989675	-6.129492
H	3.941103	-7.360718	-6.723366
O	4.147152	-5.023051	-6.769926
H	0.164926	-2.140635	-3.241073
C	-2.432262	0.262817	-4.508503
H	-2.386281	-0.174074	-5.509441
H	-3.152774	1.080898	-4.514954
H	-2.774013	-0.497288	-3.807343

AGG_16.95_NcC.xyz
28

0 eng= -740.082656716 zpe= -739.845807

N	-2.785958	0.634286	2.205009
C	-3.000476	-0.672849	1.491753
C	-3.675157	-0.233537	0.181512
N	-3.166578	-0.661526	-0.999090
C	-1.773047	-0.986026	-1.235049
C	-0.852808	0.193214	-0.891658
N	0.207246	0.367169	-1.675544
C	1.181044	1.412870	-1.434790
C	2.179370	1.385084	-2.572660
H	-2.014902	-1.109831	1.353122
O	-4.668913	0.452905	0.281911
H	-3.668694	1.162218	2.166307
H	-2.061955	1.148204	1.671628
H	-3.691531	-0.311695	-1.792730
H	-1.667623	-1.284334	-2.277546
H	-1.461661	-1.844772	-0.633641
O	-1.087578	0.905009	0.085384
H	0.362405	-0.200040	-2.502867
H	0.700715	2.393401	-1.391290
H	1.705261	1.259404	-0.488075
O	3.105944	2.322388	-2.421150
H	3.726328	2.279061	-3.164050
O	2.121833	0.606081	-3.482473
H	-2.500885	0.511759	3.177945
C	-3.920469	-1.576284	2.296025
H	-4.088117	-2.502233	1.744344
H	-4.887850	-1.096355	2.453238
H	-3.472950	-1.834462	3.257891

AGG_17.22_c-t_N-gcggtgtt.xyz
28
0 eng= -740.082905867 zpe= -739.845705

N	-2.770465	0.260028	0.162040
C	-3.003818	-1.010930	-0.611193
C	-2.244847	-0.773726	-1.928683
N	-1.252295	-1.633341	-2.269259
C	-0.538567	-2.519768	-1.353807
C	0.081894	-1.705799	-0.213955
N	1.250603	-1.087197	-0.487591
C	1.825007	-0.158468	0.454348
C	0.990683	1.099702	0.618421
H	-2.596240	-1.811803	-0.001398
O	-2.579983	0.171668	-2.606842
H	-3.322795	0.281400	1.021219
H	-3.036117	1.054694	-0.433803
H	-0.818206	-1.411812	-3.157000
H	0.219624	-3.049661	-1.930276
H	-1.204278	-3.265080	-0.918698
O	-0.496495	-1.563928	0.850816

H	1.678169	-1.191608	-1.394707
H	1.901636	-0.618855	1.444105
H	2.830139	0.120209	0.140322
O	1.655587	2.014928	1.300894
H	1.105464	2.803534	1.419490
O	-0.135667	1.253193	0.207448
H	-1.765754	0.377131	0.396444
C	-4.488633	-1.190633	-0.880772
H	-4.642083	-2.107974	-1.450554
H	-4.877744	-0.357880	-1.468574
H	-5.044732	-1.281287	0.054485

GGA_0.00_NcA.xyz
28
0 eng= -740.086554202 zpe= -739.848842

N	-0.658347	1.472592	-0.804045
C	-0.759856	0.657753	-2.051139
C	-1.937533	-0.293555	-1.794643
N	-1.738203	-1.624305	-1.952040
C	-0.438880	-2.254826	-1.881396
C	0.387580	-1.720792	-0.701779
N	1.702925	-1.992825	-0.741887
C	2.621677	-1.452137	0.242832
C	2.682283	0.070867	0.129710
H	-1.031602	1.316847	-2.875307
H	0.217730	0.218325	-2.229897
O	-2.986890	0.213706	-1.471933
H	-1.579583	1.873721	-0.592506
H	0.057313	2.197923	-0.877646
H	-2.548534	-2.179548	-1.706921
H	0.120799	-2.122075	-2.812389
H	-0.586883	-3.328121	-1.750722
O	-0.120252	-1.050795	0.185736
H	2.087258	-2.511445	-1.519198
O	3.562046	0.569507	0.986219
H	3.589522	1.533906	0.905857
O	2.013736	0.748439	-0.614235
H	-0.383514	0.826084	-0.043306
H	2.224370	-1.657381	1.242338
C	4.001714	-2.087868	0.090991
H	4.676760	-1.702119	0.852201
H	4.427280	-1.867800	-0.891165
H	3.922174	-3.168535	0.211709

GGA_4.66_NcA.xyz
28
0 eng= -740.084894141 zpe= -739.847067

N	-1.738388	-1.684010	0.106267
C	-1.468422	-1.679970	-1.361839
C	-2.734686	-1.073842	-1.983813

N	-2.599958	-0.028184	-2.834629
C	-1.460445	0.861527	-2.835145
C	-1.050530	1.256595	-1.406483
N	0.170492	1.807706	-1.291388
C	0.768079	2.136234	-0.005041
C	0.930420	0.874940	0.841963
O	1.533065	1.161896	1.987009
H	1.663160	0.351741	2.501021
H	-0.528470	-1.157198	-1.517445
H	-1.385352	-2.711820	-1.702425
O	-3.788432	-1.584397	-1.680519
H	-0.995641	-2.146170	0.633703
H	-2.649595	-2.127165	0.273830
H	-3.487883	0.339347	-3.153536
H	-1.731650	1.763265	-3.386780
H	-0.605950	0.415700	-3.353653
O	-1.775513	1.024725	-0.450298
H	0.726786	1.939731	-2.123152
H	1.785233	2.481160	-0.210106
O	0.583735	-0.239676	0.530662
H	-1.776994	-0.694864	0.407419
C	0.022666	3.243174	0.750335
H	-0.989504	2.928849	1.000467
H	-0.025357	4.131824	0.121362
H	0.561320	3.489359	1.664298

GGA_5.24_OtA.xyz
28
0 eng= -740.080588501 zpe= -739.846846

N	-1.218658	-0.270125	-4.795513
C	-1.878682	0.724011	-3.969624
C	-0.897710	1.394930	-3.036300
N	0.361080	1.021894	-3.108500
C	1.427063	1.318887	-2.150230
C	1.629590	2.808112	-1.907567
N	2.866865	3.258027	-1.863412
C	3.190050	4.651710	-1.558288
C	4.641018	4.834554	-1.973265
H	-2.346964	1.522741	-4.551033
H	-2.664919	0.308046	-3.332398
O	-1.336516	2.279333	-2.241791
H	-1.304897	-0.068019	-5.783252
H	-1.575997	-1.203619	-4.637459
H	0.503638	0.299637	-3.820611
H	2.337628	0.849022	-2.517077
H	1.178613	0.869001	-1.182342
O	0.646797	3.543834	-1.699947
H	3.660263	2.664728	-2.091247
H	2.561877	5.299198	-2.175895
O	5.023684	6.097923	-1.856796

H	5.957016	6.172693	-2.105837
O	5.341881	3.930041	-2.338048
H	-0.524184	2.894521	-1.873107
C	2.988743	4.986170	-0.078567
H	3.629856	4.360627	0.545188
H	1.946639	4.825251	0.194957
H	3.239040	6.032386	0.094410

GGA_6.84_OtA.xyz
28
0 eng= -740.080456233 zpe= -739.846238

N	-0.899033	2.170279	-3.322171
C	-0.798686	2.160380	-1.874541
C	0.112207	1.052521	-1.399020
N	0.635101	0.262796	-2.311187
C	1.733483	-0.687870	-2.131736
C	1.477177	-1.717465	-1.040074
N	1.805162	-2.968603	-1.290604
C	1.726466	-4.022837	-0.279564
C	2.561916	-5.171981	-0.821710
H	-0.391794	3.086136	-1.456892
H	-1.761158	2.000272	-1.381404
O	0.317270	0.939603	-0.153603
H	-0.535153	3.022372	-3.729556
H	-1.851935	2.055619	-3.642572
H	0.338547	0.523910	-3.255981
H	2.640577	-0.142757	-1.846813
H	1.913854	-1.165772	-3.092680
O	1.036249	-1.354114	0.066096
H	2.200937	-3.249041	-2.183956
O	2.674270	-6.149825	0.065596
H	3.195011	-6.871467	-0.317688
O	3.033030	-5.181348	-1.926168
H	0.704713	-0.045019	0.080061
H	2.184606	-3.656750	0.643140
C	0.286676	-4.463929	-0.007129
H	0.286123	-5.251168	0.746157
H	-0.175651	-4.845340	-0.919364
H	-0.289927	-3.619046	0.367499

GGA_7.67_NcB.xyz
28
0 eng= -740.08320422 zpe= -739.845919

N	-0.832352	0.864404	1.417405
C	-1.581171	-0.376818	1.071857
C	-1.224789	-0.662471	-0.396497
N	-1.375015	-1.926074	-0.836611
C	-1.542175	-3.088908	0.026039
C	-0.384606	-3.243113	1.019159
N	0.864804	-3.075618	0.515193

C	1.994984	-3.027393	1.413930
C	1.891108	-1.806970	2.320453
H	-1.308584	-1.153901	1.782791
H	-2.652934	-0.177693	1.130215
O	-0.873300	0.272905	-1.086951
H	-0.729973	1.406109	0.541712
H	0.106470	0.583036	1.747233
H	-1.243704	-2.049060	-1.832815
H	-1.605678	-3.973772	-0.607958
H	-2.461974	-3.036102	0.610614
O	-0.591543	-3.460394	2.193423
H	0.992623	-2.780435	-0.441756
O	2.700986	-1.905993	3.355822
H	2.637513	-1.114662	3.909992
O	1.184841	-0.842554	2.111751
H	-1.293996	1.424807	2.133607
H	1.970015	-3.902427	2.067766
C	3.309954	-2.986151	0.635605
H	3.392929	-3.878876	0.015529
H	4.153562	-2.963796	1.324121
H	3.357191	-2.101624	-0.004615

GGA_9.65_NtA.xyz
28
0 eng= -740.082823024 zpe= -739.845166

N	0.044248	2.505023	0.147547
C	-0.846689	2.035683	-0.953548
C	-0.508152	0.548814	-1.099986
N	-1.489248	-0.255596	-1.528878
C	-1.380194	-1.700853	-1.303194
C	-1.682925	-1.870624	0.184904
N	-0.598642	-1.890571	1.007619
C	-0.803905	-1.611371	2.408906
C	-1.374886	-0.207298	2.586495
H	-1.881571	2.212985	-0.665873
H	-0.604045	2.581096	-1.866027
O	0.618680	0.189945	-0.803916
H	0.105211	3.520871	0.216331
H	0.973612	2.090767	-0.019220
H	-2.429153	0.114775	-1.578531
H	-0.384104	-2.031592	-1.592627
H	-2.134511	-2.211488	-1.896348
O	-2.820983	-1.834600	0.599176
H	0.310212	-1.697248	0.607551
O	-2.053577	-0.093496	3.708852
H	-2.386423	0.809804	3.811784
O	-1.190294	0.723844	1.822426
H	-0.304683	2.091373	1.032221
H	-1.542944	-2.306253	2.811028
C	0.507703	-1.722346	3.185898

```
H       0.342930        -1.511274       4.242113
H       1.250264        -1.021351       2.795770
H       0.898068        -2.735444       3.090657

GGA_10.54_OtB.xyz
28
0 eng= -740.07859645 zpe= -739.844826
N       -1.092613       3.252444        -1.937524
C       0.099536        2.490073        -1.616151
C       -0.167267       1.503242        -0.502620
N       -1.373549       1.492422        0.019167
C       -1.969519       0.483536        0.896115
C       -1.152510       0.158052        2.134446
N       -1.815134       -0.082059       3.258877
C       -1.162426       -0.763561       4.371358
C       -0.820625       -2.185656       3.918379
H       0.480638        1.900778        -2.455584
H       0.930025        3.116662        -1.280485
O       0.785632        0.752822        -0.134933
H       -1.410177       3.094129        -2.885284
H       -0.956837       4.248035        -1.817275
H       -1.979701       2.185597        -0.430374
H       -2.075511       -0.462542       0.351191
H       -2.965631       0.833442        1.161670
O       0.080347        0.053431        2.070314
H       -2.826063       -0.066095       3.248490
O       -0.113891       -2.815726       4.852190
H       0.085367        -3.713542       4.548683
O       -1.157957       -2.659725       2.871575
H       0.574594        0.324703        0.828346
H       -0.223475       -0.250072       4.588337
C       -2.065396       -0.768255       5.600067
H       -1.561776       -1.277080       6.420241
H       -3.001061       -1.294801       5.394434
H       -2.288586       0.253871        5.906819

GGA_10.85_t-t_O1-ctggtgtt.xyz
28
0 eng= -740.078852415 zpe= -739.844708
N       -0.446404       2.760565        -3.007796
C       -0.443046       2.426800        -1.595657
C       0.100020        1.035853        -1.369402
N       0.472765        0.349368        -2.423505
C       0.810346        -1.074400       -2.467548
C       1.961897        -1.414305       -1.532160
N       2.998918        -2.082343       -2.018650
C       4.199699        -2.270945       -1.213164
C       4.835426        -0.898355       -0.975879
H       0.172946        3.099360        -0.992793
H       -1.440884       2.439131        -1.146974
```

O	0.164675	0.607874	-0.174880
H	0.151559	3.549771	-3.217462
H	-1.373523	2.970832	-3.354356
H	0.355627	0.882241	-3.290171
H	1.034088	-1.326557	-3.502067
H	-0.057320	-1.661624	-2.149180
O	1.885909	-1.100396	-0.336426
H	3.071051	-2.228484	-3.015974
O	5.863607	-1.002517	-0.138321
H	6.257100	-0.127251	-0.008643
O	4.463400	0.120461	-1.484450
H	0.841978	-0.211494	-0.101525
H	3.902600	-2.667814	-0.239835
C	5.166903	-3.224058	-1.907239
H	6.053649	-3.357206	-1.289803
H	5.480971	-2.824676	-2.875130
H	4.695545	-4.195913	-2.055592

GGA_14.24_NcB.xyz
28
0 eng= -740.080814759 zpe= -739.843418

N	-1.083845	0.403836	-3.317787
C	-1.800876	-0.813326	-2.844302
C	-2.645829	-0.343009	-1.648286
N	-2.993481	-1.267467	-0.731753
C	-2.354334	-2.570257	-0.592861
C	-0.836101	-2.454225	-0.411337
N	-0.407338	-1.490666	0.440777
C	1.013548	-1.225349	0.588954
C	1.592883	-0.872620	-0.781292
H	-2.483282	-1.155071	-3.624841
H	-1.063902	-1.585208	-2.631962
O	-2.987818	0.821773	-1.620670
H	-0.201189	0.472788	-2.780728
H	-1.684736	1.215372	-3.095872
H	-3.643499	-0.944672	-0.026138
H	-2.525744	-3.205247	-1.463388
H	-2.792541	-3.071820	0.270581
O	-0.075505	-3.152509	-1.048378
H	-1.073401	-0.894796	0.907096
O	2.880675	-1.128669	-0.868311
H	3.212968	-0.851238	-1.734832
O	0.959032	-0.352708	-1.677405
H	-0.871896	0.380034	-4.315215
H	1.102549	-0.299400	1.167530
C	1.767683	-2.340205	1.311886
H	1.302574	-2.506098	2.283441
H	2.807277	-2.054524	1.465147
H	1.734884	-3.262793	0.734318

```
GGA_15.45_NtA.xyz
28
0 eng= -740.080638193 zpe= -739.842957
N       -0.849159       1.499982        -2.362813
C       -2.094349       0.759367        -2.722056
C       -2.160513       -0.373100       -1.692722
N       -2.710652       -1.529027       -2.092423
C       -2.389459       -2.752465       -1.350646
C       -0.948807       -3.061037       -1.757160
N       0.015510        -2.641221       -0.899204
C       1.381649        -2.544174       -1.385441
C       1.380085        -1.706505       -2.668319
H       -2.949633       1.428091        -2.621539
H       -2.004582       0.404193        -3.747252
O       -1.686878       -0.166131       -0.589419
H       -0.852065       1.625716        -1.340428
H       -0.761529       2.398751        -2.837222
H       -2.902346       -1.652116       -3.077882
H       -3.047374       -3.552134       -1.681482
H       -2.522164       -2.570744       -0.285717
O       -0.699624       -3.508577       -2.857804
H       -0.261988       -2.091874       -0.098728
H       1.932343        -1.935501       -0.660049
O       2.289781        -2.083691       -3.538484
H       2.257605        -1.509249       -4.317522
O       0.655648        -0.742118       -2.848420
H       -0.041694       0.882975        -2.580450
C       2.068734        -3.898998       -1.528099
H       2.035446        -4.409712       -0.566075
H       3.109565        -3.769656       -1.822033
H       1.562436        -4.507207       -2.275961

SGA_0.00_NcA.xyz
31
0 eng= -779.389728157 zpe= -779.123537
C       -0.723707       0.596131        -2.011359
H       -1.036219       1.204948        -2.861224
H       0.266887        0.184781        -2.193151
C       -1.859709       -0.391425       -1.709040
O       -2.909650       0.081392        -1.336896
N       -0.632998       1.479758        -0.815442
H       -1.598823       1.616809        -0.485996
N       -1.638572       -1.712917       -1.910163
H       -2.426641       -2.288535       -1.640290
C       -0.322214       -2.307132       -1.849283
H       0.244621        -2.116754       -2.765990
H       -0.437014       -3.389123       -1.768252
C       0.472158        -1.791986       -0.639075
O       -0.046856       -1.095081       0.218018
N       1.777537        -2.117416       -0.620166
```

H	2.176795	-2.652553	-1.378369
C	2.667314	-1.604291	0.403038
H	2.239730	-1.825304	1.386527
C	2.746201	-0.081396	0.321583
O	3.562896	0.393420	1.253065
H	3.602970	1.358504	1.191529
O	2.145054	0.616828	-0.458964
H	-0.135949	0.934664	-0.093962
C	0.044549	2.782782	-1.062263
H	1.044334	2.577549	-1.436359
H	0.099521	3.329529	-0.123218
H	-0.540517	3.342878	-1.789218
C	4.047427	-2.246337	0.281199
H	4.700416	-1.881628	1.071499
H	4.504055	-2.008916	-0.682713
H	3.957580	-3.328829	0.376485

SGA_5.57_NcA.xyz
31
0 eng= -779.388168797 zpe= -779.121416

N	-2.274133	-2.396162	0.726696
C	-2.196979	-1.273727	-0.249238
C	-3.375189	-0.348993	0.089093
N	-3.140744	0.981462	0.197586
C	-1.853260	1.522094	0.571441
C	-1.239801	0.745476	1.749097
N	0.049938	1.027649	2.012824
C	0.810670	0.316903	3.028847
C	0.862680	-1.174282	2.704350
O	1.580573	-1.821144	3.613364
H	1.634627	-2.758465	3.378148
H	-1.195528	-0.852355	-0.188984
H	-2.372814	-1.678118	-1.247344
O	-4.462288	-0.867936	0.203095
H	-3.276555	-2.576979	0.877271
H	-3.965398	1.504683	0.464929
H	-1.990234	2.564956	0.862091
H	-1.157839	1.517035	-0.273489
O	-1.878566	-0.101621	2.351603
H	0.525605	1.719851	1.453518
O	0.350774	-1.714179	1.753193
C	-1.557972	-3.630844	0.302691
H	-0.513440	-3.376422	0.142336
H	-2.017856	-3.999910	-0.611996
H	-1.645366	-4.372952	1.093420
H	-1.886725	-2.033889	1.611551
H	1.844138	0.665561	2.951925
C	0.311126	0.579832	4.454545
H	0.342687	1.651668	4.648969
H	0.955516	0.073444	5.171577

```
H       -0.711862       0.226164        4.575500

SGA_5.71_NcA.xyz
31
0 eng= -779.387543797 zpe= -779.121364
N       -0.617248       1.370390        -0.592379
C       -0.490313       0.546249        -1.839701
C       -1.892973       0.351272        -2.428296
N       -2.358896       -0.919020       -2.547175
C       -1.835312       -2.071034       -1.853452
C       -1.638427       -1.803495       -0.355706
N       -0.884054       -2.706409       0.297499
C       -0.511759       -2.519117       1.685582
C       0.386678        -1.292706       1.830810
O       0.723406        -1.104482       3.098211
H       1.302488        -0.332425       3.174063
H       0.106575        1.108866        -2.557662
H       0.033986        -0.367328       -1.565893
O       -2.519370       1.325952        -2.774747
H       -1.418613       0.982810        -0.070055
H       -3.308768       -0.961648       -2.893803
H       -0.891077       -2.409454       -2.292128
H       -2.547850       -2.889785       -1.969166
O       -2.104874       -0.816360       0.191419
H       -0.472042       -3.478239       -0.207771
H       -1.413286       -2.297514       2.265490
O       0.744153        -0.571189       0.929168
H       0.193823        1.155795        -0.001305
C       -0.760184       2.842454        -0.809049
H       0.140332        3.203077        -1.302759
H       -0.876058       3.320204        0.161666
H       -1.631312       3.012888        -1.436025
C       0.163594        -3.775104       2.233149
H       0.412831        -3.638000       3.283451
H       1.083002        -3.994453       1.684810
H       -0.515768       -4.623085       2.144195

SGA_7.07_NcB.xyz
31
0 eng= -779.386603702 zpe= -779.120846
N       -1.653570       -0.579442       0.738993
C       -2.013939       -1.598652       -0.280019
C       -1.640410       -0.992632       -1.640170
N       -1.381362       -1.847422       -2.651089
C       -1.148601       -3.275169       -2.485238
C       0.011946        -3.573520       -1.530157
N       1.102379        -2.771444       -1.637550
C       2.189726        -2.916881       -0.697482
C       1.723081        -2.553383       0.706602
O       2.536791        -3.042337       1.622178
```

H	2.241600	-2.778752	2.505787
H	-1.510240	-2.528011	-0.018472
H	-3.096348	-1.749615	-0.258036
O	-1.636113	0.217117	-1.744095
H	-1.842135	0.340266	0.315178
H	-1.252990	-1.413554	-3.556471
H	-0.930029	-3.700215	-3.465537
H	-2.025964	-3.791932	-2.092779
O	-0.068177	-4.454815	-0.702244
H	1.110956	-1.997851	-2.285726
H	2.497273	-3.964705	-0.660650
O	0.756404	-1.869531	0.970536
H	-0.632488	-0.661261	0.867961
C	-2.332605	-0.757428	2.051941
H	-2.073936	-1.741862	2.437368
H	-3.408013	-0.676379	1.906118
H	-1.985750	0.015864	2.733786
C	3.371969	-2.031308	-1.090858
H	3.730287	-2.319367	-2.079471
H	4.187605	-2.155497	-0.379721
H	3.081063	-0.978000	-1.110045

SGA_8.70_NtA.xyz
31
0 eng= -779.386267851 zpe= -779.120225

N	-3.671281	1.716867	0.090253
C	-2.541338	1.396632	-0.824775
C	-1.870936	0.164818	-0.213249
N	-1.288772	-0.694425	-1.062131
C	-0.998084	-2.058128	-0.610590
C	-2.358012	-2.753982	-0.594215
N	-3.006880	-2.751914	0.602279
C	-4.427094	-3.010036	0.601785
C	-5.150973	-1.943269	-0.213189
O	-6.311446	-2.375140	-0.665333
H	-6.757903	-1.674675	-1.162321
H	-2.942566	1.217566	-1.821993
H	-1.853770	2.244309	-0.842843
O	-1.921131	0.027336	0.997455
H	-4.344209	0.934250	0.004348
H	-1.452033	-0.579181	-2.053309
H	-0.514845	-2.014441	0.364042
H	-0.346096	-2.541938	-1.333518
O	-2.869937	-3.152209	-1.617968
H	-2.620053	-2.196715	1.354634
H	-4.617738	-3.966544	0.112165
O	-4.748183	-0.808499	-0.390455
H	-3.292286	1.633208	1.043952
C	-4.317484	3.035799	-0.146902
H	-4.697693	3.060285	-1.166169

H   -3.577311   3.820662   -0.003216
H   -5.135442   3.157845   0.559697
C   -4.980748   -3.020827   2.026360
H   -6.051509   -3.222511   2.017233
H   -4.805941   -2.059983   2.517526
H   -4.483760   -3.803216   2.599777

SGA_10.62_NcA.xyz
31
0 eng= -779.385650183 zpe= -779.119491
N   -1.861257   1.770941   -1.123902
C   -1.970637   0.276731   -1.192680
C   -2.283718   -0.115218   -2.642124
N   -1.396365   -0.914663   -3.289504
C   -0.025608   -1.133159   -2.895720
C   0.703167   0.183039   -2.586670
N   1.881636   0.044722   -1.950596
C   2.645047   1.179704   -1.456207
C   1.835148   1.945608   -0.411500
O   2.530465   2.970272   0.059884
H   2.024479   3.417552   0.753748
H   -1.045858   -0.128471   -0.787242
H   -2.808207   -0.031811   -0.566835
O   -3.308480   0.285533   -3.144230
H   -1.271114   1.998625   -0.316187
H   -1.648100   -1.084215   -4.254912
H   0.489327   -1.634527   -3.717273
H   0.040606   -1.798794   -2.029002
O   0.212751   1.266539   -2.862951
H   2.200094   -0.883146   -1.714188
O   0.723331   1.662969   -0.030901
C   -3.159321   2.510034   -1.073621
H   -3.738280   2.247681   -1.954690
H   -2.943454   3.576208   -1.049097
H   -3.686644   2.211297   -0.169476
H   -1.290832   2.054916   -1.935735
H   3.501417   0.770651   -0.912736
C   3.169156   2.095166   -2.568953
H   3.766878   1.502746   -3.261339
H   3.797871   2.874668   -2.141273
H   2.345035   2.553168   -3.113643

SGA_13.66_c-t_N-tcgttgtt.xyz
31
0 eng= -779.384358459 zpe= -779.118333
N   -1.032000   1.429650   -0.556727
C   -0.985217   0.517775   -1.736047
C   -1.361417   -0.864736   -1.176234
N   -0.998505   -1.952850   -1.881492
C   0.074402   -1.961236   -2.848558

C	1.388529	-1.447973	-2.238291
N	2.408935	-1.312798	-3.112709
C	3.605329	-0.589863	-2.734769
C	3.254812	0.866371	-2.432350
O	4.330385	1.534978	-2.027488
H	4.093782	2.459002	-1.865114
H	-1.757913	0.824714	-2.444481
H	0.000207	0.601699	-2.192169
O	-2.032936	-0.893436	-0.164033
H	-1.814977	1.076890	0.020071
H	-1.248800	-2.825856	-1.434171
H	-0.180095	-1.348840	-3.719549
H	0.206509	-2.981978	-3.209240
O	1.469796	-1.142429	-1.065601
H	2.248251	-1.479964	-4.096148
H	3.990113	-1.010819	-1.801561
O	2.159934	1.361458	-2.535275
H	-1.233559	2.386407	-0.848328
C	0.223013	1.388693	0.264224
H	0.096229	2.050650	1.118138
H	1.047706	1.703633	-0.371505
H	0.375526	0.359908	0.583631
C	4.665470	-0.690967	-3.829053
H	5.570136	-0.169024	-3.522977
H	4.308123	-0.245966	-4.761250
H	4.908552	-1.738867	-4.005835

SGA_14.67_c-t_N-gcggtgtt.xyz
31
0 eng= -779.384021326 zpe= -779.117951

C	-3.072009	-1.018721	-0.693477
H	-2.744395	-1.848725	-0.075422
H	-4.138840	-1.088467	-0.906841
C	-2.337653	-0.831710	-2.023019
O	-2.704865	0.063870	-2.748627
N	-2.835649	0.236694	0.078473
H	-3.173629	1.019592	-0.492496
N	-1.298699	-1.658459	-2.304768
H	-0.858398	-1.464913	-3.195602
C	-0.599894	-2.516942	-1.353989
H	0.181598	-3.045606	-1.899594
H	-1.267435	-3.264972	-0.925816
C	-0.019168	-1.678634	-0.209780
O	-0.642202	-1.505150	0.827452
N	1.164986	-1.083261	-0.450609
H	1.628242	-1.223564	-1.336570
C	1.756748	-0.151145	0.488472
H	1.759968	-0.613278	1.482014
C	0.891065	1.097794	0.626084
O	1.472457	1.995925	1.405862

H	0.900825	2.773003	1.490124
O	-0.193507	1.265180	0.118369
H	-1.814136	0.383381	0.179075
C	-3.460192	0.231274	1.430085
H	-2.990477	-0.565013	2.004781
H	-4.528985	0.053911	1.325524
H	-3.280272	1.194479	1.902765
C	3.185472	0.189515	0.069993
H	3.776229	-0.725258	0.014832
H	3.642201	0.854989	0.799361
H	3.198437	0.683608	-0.904576

SGA_14.69_NcB.xyz
31
0 eng= -779.384385795 zpe= -779.11794

C	-1.039222	-1.258860	-2.666595
H	-1.885912	-1.802902	-3.092854
H	-0.260140	-1.964218	-2.380374
C	-1.574559	-0.348045	-1.552109
O	-1.978574	0.753245	-1.864135
N	-0.480745	-0.356121	-3.705982
H	0.454689	-0.080319	-3.363667
N	-1.599204	-0.844066	-0.297213
H	-2.063643	-0.258119	0.384960
C	-0.870366	-2.028029	0.135049
H	-1.239508	-2.937045	-0.342742
H	-1.015327	-2.142916	1.209900
C	0.628922	-1.937170	-0.169716
O	1.238271	-2.891400	-0.604682
N	1.206651	-0.722560	0.011197
H	0.664697	0.051420	0.362730
C	2.612385	-0.531542	-0.306561
H	2.800717	0.546742	-0.265517
C	2.831040	-0.899569	-1.774556
O	4.045893	-1.346921	-2.011931
H	4.148190	-1.526748	-2.958173
O	2.003845	-0.739549	-2.649314
H	-1.078942	0.481914	-3.721615
C	-0.338271	-0.980730	-5.050038
H	0.312222	-1.847724	-4.953125
H	0.105798	-0.254614	-5.727364
H	-1.322214	-1.278200	-5.407461
C	3.555588	-1.244362	0.660052
H	3.337490	-0.911039	1.674744
H	4.591287	-1.001354	0.427308
H	3.423766	-2.323622	0.598611

SGA_15.46_NtA.xyz
31
0 eng= -779.384073373 zpe= -779.11765

N	-0.883602	0.996826	-3.034061
C	-2.303075	0.611467	-3.266306
C	-2.465064	-0.738969	-2.565760
N	-3.315742	-1.616605	-3.120653
C	-3.185138	-3.036215	-2.780145
C	-1.930452	-3.485206	-3.527802
N	-0.781088	-3.500756	-2.805427
C	0.484193	-3.529132	-3.519767
C	0.484754	-2.392239	-4.546799
O	1.143899	-2.684729	-5.646977
H	1.121826	-1.928896	-6.251735
H	-2.949411	1.364356	-2.811268
H	-2.477793	0.565278	-4.340938
O	-1.799291	-0.947930	-1.566535
H	-0.703182	0.826635	-2.035596
H	-3.672691	-1.417311	-4.045578
H	-4.051500	-3.574198	-3.156852
H	-3.117988	-3.140279	-1.698628
O	-1.954424	-3.658835	-4.729281
H	-0.804808	-3.135027	-1.864263
H	1.260729	-3.251873	-2.798792
O	-0.016551	-1.299031	-4.350181
H	-0.308113	0.293620	-3.533082
C	-0.536193	2.385392	-3.439465
H	0.517885	2.557417	-3.233016
H	-1.148597	3.082366	-2.870587
H	-0.730998	2.497051	-4.504257
C	0.813090	-4.897946	-4.108314
H	0.808569	-5.633706	-3.304563
H	1.799340	-4.886955	-4.570493
H	0.074095	-5.178027	-4.857278

GAS_0.00_OtB.xyz
31
0 eng= -779.378207905 zpe= -779.115821

C	0.779656	1.345025	-2.989899
H	0.362150	2.194747	-3.538106
H	1.546997	0.911497	-3.636647
C	-0.330463	0.321875	-2.900611
O	-0.869373	-0.057534	-3.983614
N	1.280759	1.696921	-1.674043
H	2.267123	1.496087	-1.572037
H	1.138475	2.674338	-1.454245
N	-0.655647	-0.118464	-1.705882
H	-0.081966	0.325110	-0.982569
C	-1.849790	-0.875253	-1.289108
C	-2.049007	-2.155033	-2.089935
O	-1.907706	-2.128827	-3.328276
N	-2.477107	-3.256549	-1.476191
C	-3.036133	-4.288645	-2.338610

H	-2.413191	-4.433653	-3.220163
H	-3.094276	-5.233081	-1.798536
C	-4.430674	-3.857796	-2.779915
O	-4.988125	-4.777886	-3.556556
H	-5.870197	-4.479501	-3.823838
O	-4.939228	-2.821811	-2.454607
C	-2.847857	-3.303365	-0.057085
H	-3.030777	-4.342480	0.206820
H	-3.758921	-2.729461	0.128736
H	-2.033750	-2.948152	0.571591
H	-1.423927	-0.970642	-3.810257
H	-1.685745	-1.102164	-0.239081
C	-3.126441	-0.035453	-1.447017
H	-4.000922	-0.626143	-1.167684
H	-3.254302	0.268112	-2.487848
H	-3.067706	0.850731	-0.815281

GAS_0.10_OtB.xyz
31
0 eng= -779.378105335 zpe= -779.115782

C	-2.760300	0.837491	-0.914711
H	-3.094235	1.515794	-1.705425
H	-2.004696	1.383261	-0.343559
C	-2.033632	-0.289648	-1.613979
O	-1.070300	0.016931	-2.378812
N	-3.844527	0.336934	-0.089993
H	-4.748250	0.681387	-0.387402
H	-3.726879	0.575391	0.886254
N	-2.438843	-1.516045	-1.370867
H	-3.226923	-1.515831	-0.716555
C	-2.144744	-2.764516	-2.098448
H	-2.609867	-3.542379	-1.499398
C	-0.652219	-3.025257	-2.252613
O	0.111001	-2.074588	-2.514156
N	-0.181507	-4.270664	-2.209077
C	1.165149	-4.452681	-2.735298
H	1.842006	-3.695862	-2.341669
H	1.540673	-5.435307	-2.450068
C	1.122666	-4.341984	-4.255857
O	2.336610	-4.479533	-4.773802
H	2.282613	-4.407337	-5.738389
O	0.116596	-4.160458	-4.882019
H	-0.474320	-0.866221	-2.563111
C	-2.760124	-2.748226	-3.505262
H	-2.543728	-3.685889	-4.019759
H	-2.329919	-1.939351	-4.098898
H	-3.839640	-2.614439	-3.436041
C	-1.005392	-5.480331	-2.150574
H	-0.413259	-6.272881	-1.696067
H	-1.300741	-5.782065	-3.158962

```
H       -1.886538       -5.335959       -1.532545

GAS_1.32_t-c_O1-ctggcgtt.xyz
31
0 eng= -779.377908208 zpe= -779.115318
N       -3.770470       -0.266829       1.050675
C       -3.207054       1.026970        0.713918
C       -1.974428       0.880465        -0.147786
N       -1.593030       -0.330128       -0.468472
C       -0.573784       -0.736753       -1.450040
C       0.786808        -0.139813       -1.086340
N       1.872401        -0.910962       -1.020417
C       1.826070        -2.362516       -0.985683
C       1.139581        -2.882690       0.272833
O       1.466948        -4.148421       0.498295
H       0.998333        -4.470776       1.282889
H       -3.896541       1.663462        0.151446
H       -2.901519       1.605756        1.589620
O       -1.374055       1.946056        -0.502177
H       -3.762060       -0.442324       2.047007
H       -4.721109       -0.373403       0.721894
H       -2.177676       -1.045488       -0.028319
H       -0.559468       -1.820333       -1.397515
O       0.855111        1.093119        -0.940575
H       1.315396        -2.782250       -1.856936
H       2.845927        -2.743404       -1.018843
O       0.376950        -2.235239       0.935354
H       -0.380778       1.711782        -0.805356
C       -0.952045       -0.297358       -2.868001
H       -0.191163       -0.636957       -3.573342
H       -1.022200       0.788438        -2.941629
H       -1.908836       -0.737937       -3.148205
C       3.162874        -0.296991       -0.676810
H       3.392493        -0.484047       0.374861
H       3.106570        0.772798        -0.850207
H       3.938126        -0.731941       -1.306522

GAS_1.76_OtB.xyz
31
0 eng= -779.37705655 zpe= -779.115152
C       -1.225283       0.071191        -4.452735
H       -2.294242       -0.089113       -4.620567
H       -0.711331       -0.370716       -5.310501
C       -0.855489       -0.777929       -3.257340
O       -1.120124       -2.017392       -3.310417
N       -0.879043       1.464152        -4.238493
H       -1.686655       2.072936        -4.266200
H       -0.210156       1.803033        -4.917943
N       -0.268151       -0.177788       -2.247553
H       -0.146631       0.821932        -2.436233
```

C	-0.025787	-0.662271	-0.877074
H	0.585464	0.113021	-0.421172
C	0.725506	-1.990913	-0.862746
O	0.401201	-2.889170	-1.663126
N	1.660887	-2.206672	0.058081
C	2.443780	-3.423731	-0.096673
H	2.984883	-3.636336	0.824713
H	1.795776	-4.271396	-0.318795
C	3.432439	-3.246206	-1.243767
O	4.176712	-4.336736	-1.391286
H	4.794894	-4.205475	-2.125084
O	3.515273	-2.253258	-1.908242
H	-0.533775	-2.534146	-2.565975
C	-1.340954	-0.818902	-0.104010
H	-1.138735	-1.131906	0.921979
H	-1.974674	-1.573939	-0.573002
H	-1.874023	0.131576	-0.081395
C	2.192493	-1.164562	0.941698
H	2.866529	-0.499610	0.396232
H	2.746958	-1.651136	1.741093
H	1.386256	-0.598616	1.405384

GAS_5.67_c-t_N-tcggtgtt.xyz
31
0 eng= -779.379241546 zpe= -779.113661

N	-0.675525	-0.640945	-3.024309
C	-1.963737	-1.383038	-2.895728
C	-2.944283	-0.361111	-2.302765
N	-4.030734	-0.804461	-1.646426
C	-4.272852	-2.146595	-1.094202
C	-3.069105	-2.584473	-0.234962
N	-2.770125	-1.849092	0.871537
C	-1.525703	-2.187004	1.520302
C	-0.318922	-1.760669	0.705767
O	0.805399	-2.088359	1.313508
H	1.569167	-1.811161	0.787399
H	-2.315000	-1.651795	-3.893417
H	-1.803065	-2.278562	-2.299118
O	-2.712212	0.816962	-2.507411
H	-0.191631	-0.683977	-2.112375
H	-0.921892	0.346689	-3.210510
H	-4.658310	-0.056801	-1.376730
H	-5.138227	-2.033146	-0.438388
O	-2.386945	-3.536494	-0.569527
H	-1.466869	-1.707995	2.498728
H	-1.446762	-3.266249	1.668263
O	-0.368759	-1.190833	-0.362755
H	-0.072212	-1.009418	-3.758735
C	-4.608138	-3.185766	-2.153550
H	-4.859819	-4.132313	-1.675233

H	-3.764780	-3.379013	-2.816680
H	-5.460048	-2.844818	-2.742077
C	-3.427122	-0.606916	1.265655
H	-3.345997	-0.497538	2.346674
H	-4.488250	-0.639757	1.027351
H	-2.971080	0.261904	0.780945

GAS_6.16_t-c_O1-ctggcgtt.xyz
31
0 eng= -779.375597536 zpe= -779.113473

N	-4.703786	2.130512	-1.565717
C	-4.289232	0.967694	-2.329761
C	-2.839842	0.627292	-2.067740
N	-2.166977	1.408800	-1.252714
C	-0.852828	1.141878	-0.631715
C	0.212309	0.929266	-1.710759
N	1.317875	1.667758	-1.745871
C	1.608897	2.768474	-0.844244
C	2.053733	2.288413	0.530809
O	2.572132	3.292340	1.229493
H	2.833003	2.973472	2.106788
H	-4.859501	0.064778	-2.092755
H	-4.381561	1.113241	-3.409183
O	-2.356046	-0.385500	-2.658342
H	-5.432515	1.913703	-0.897829
H	-5.039907	2.878712	-2.158101
H	-2.753376	2.165465	-0.890074
H	-0.637112	2.016938	-0.024064
O	0.019005	0.011134	-2.532265
H	2.404397	3.370672	-1.283981
H	0.754948	3.438428	-0.723654
O	1.918401	1.165426	0.922959
C	-0.920218	-0.097587	0.268551
H	0.054146	-0.273528	0.721780
H	-1.206224	-0.980126	-0.304984
H	-1.655610	0.070424	1.055925
C	2.404087	1.303478	-2.667274
H	3.299316	1.081286	-2.083319
H	2.601331	2.137363	-3.341893
H	2.113038	0.428371	-3.238477
H	-1.275055	-0.371180	-2.606619

GAS_8.99_t-t_N-gtgttgtt.xyz
31
0 eng= -779.378022701 zpe= -779.112396

N	-0.723121	0.344164	-3.191322
C	-2.067955	0.734539	-2.643989
C	-1.857526	0.550090	-1.141479
N	-2.357111	-0.579741	-0.576036
C	-1.474770	-1.193386	0.416894

C	-0.122535	-1.395852	-0.301700
N	1.016540	-1.150603	0.361980
C	2.257900	-1.331104	-0.363358
C	2.537635	-0.226029	-1.370564
O	3.817559	-0.233158	-1.717857
H	3.980483	0.453306	-2.381280
H	-2.254249	1.779113	-2.883854
H	-2.825708	0.089865	-3.086105
O	-1.084743	1.294280	-0.581783
H	0.020144	0.919209	-2.758991
H	-0.670215	0.418230	-4.207619
H	-2.833201	-1.230334	-1.186885
H	-1.382048	-0.500521	1.249429
O	-0.128678	-1.780816	-1.475800
H	3.091776	-1.396324	0.333811
H	2.222911	-2.264203	-0.932180
O	1.722767	0.535315	-1.829289
H	-0.483373	-0.621923	-2.879119
C	-2.029857	-2.532502	0.882022
H	-1.366886	-2.979329	1.624378
H	-2.111463	-3.224663	0.040817
H	-3.012116	-2.393559	1.334207
C	1.043755	-0.461174	1.658265
H	0.463603	-1.008432	2.401007
H	0.663821	0.558069	1.558345
H	2.073736	-0.424047	2.005675

GAS_9.46_t-c_O1-ctgtcgtt.xyz
31
0 eng= -779.375683559 zpe= -779.112218

N	-3.916671	1.907849	-1.690753
C	-3.154979	2.133260	-0.478705
C	-2.085164	1.081865	-0.319310
N	-1.997702	0.142223	-1.209509
C	-0.916919	-0.834324	-1.314829
C	0.407311	-0.043329	-1.466343
N	1.565668	-0.694412	-1.165332
C	1.604411	-1.836571	-0.283059
C	1.250200	-1.470663	1.152283
O	1.780775	-2.295731	2.028598
H	1.501229	-2.053617	2.925779
H	-2.631782	3.094080	-0.463515
H	-3.769571	2.099702	0.424929
O	-1.333613	1.204202	0.716514
H	-4.907274	1.806475	-1.513752
H	-3.790572	2.648792	-2.368396
H	-2.674438	0.266971	-1.969221
H	-0.935353	-1.443838	-0.410048
O	0.371076	1.085605	-1.901720
H	0.918649	-2.630262	-0.596434

H	2.602077	-2.274674	-0.300033
O	0.529754	-0.544742	1.455301
H	-0.613479	0.537106	0.853026
C	-1.143558	-1.705218	-2.548219
H	-0.348157	-2.445203	-2.639532
H	-1.131418	-1.085640	-3.447356
H	-2.097801	-2.229065	-2.479257
C	2.808469	0.074989	-1.286569
H	2.720021	0.754641	-2.129312
H	3.634204	-0.614156	-1.457812
H	2.995088	0.660764	-0.381069

GAS_10.37_c-t_N-tcggtgtt.xyz
31
0 eng= -779.377731889 zpe= -779.11187

N	-3.514398	-0.182641	0.786727
C	-2.290394	-0.704287	0.113834
C	-2.632086	-0.700716	-1.385043
N	-1.607991	-0.725868	-2.254387
C	-0.225354	-0.362255	-1.936031
C	-0.127390	1.046006	-1.319985
N	-0.703340	2.116925	-1.937092
C	-0.739640	3.324526	-1.145824
C	-1.743271	3.228549	-0.011339
O	-1.742052	4.323972	0.722759
H	-2.386745	4.251362	1.441141
H	-1.441207	-0.085097	0.397281
H	-2.122263	-1.736267	0.427346
O	-3.807662	-0.727961	-1.698831
H	-4.316021	-0.445781	0.186318
H	-3.451121	0.848420	0.805013
H	-1.886750	-0.789917	-3.226627
H	0.131594	-1.011049	-1.133041
O	0.435594	1.163300	-0.245224
H	0.235506	3.526766	-0.699122
H	-1.008682	4.177875	-1.770337
O	-2.463571	2.275601	0.198478
H	-3.637402	-0.541056	1.733165
C	0.688912	-0.573735	-3.137614
H	1.710962	-0.318292	-2.857213
H	0.409453	0.040847	-3.994333
H	0.666534	-1.622536	-3.437259
C	-1.549667	2.078514	-3.126899
H	-1.195034	1.333220	-3.831886
H	-1.486596	3.045117	-3.626049
H	-2.594789	1.873127	-2.874267

GAS_10.66_OtB.xyz
31
0 eng= -779.375631421 zpe= -779.111762

N	-4.521561	-0.072078	-3.169813
C	-3.315920	0.638982	-2.794539
C	-2.503352	-0.160947	-1.801440
N	-2.955590	-1.323492	-1.445410
C	-2.359297	-2.287827	-0.521167
C	-0.902392	-2.527521	-0.949572
N	-0.034982	-2.968831	0.001615
C	1.358412	-2.752488	-0.324647
C	1.657401	-1.259896	-0.325656
O	2.945307	-1.011078	-0.438145
H	3.094765	-0.053365	-0.461356
H	-2.650213	0.827458	-3.641465
H	-3.509288	1.609877	-2.330156
O	-1.437400	0.423281	-1.384494
H	-5.363428	0.427438	-2.914369
H	-4.560781	-0.269538	-4.161364
H	-3.826516	-1.550419	-1.939033
O	-0.560771	-2.283459	-2.089093
H	2.004123	-3.251277	0.398248
H	1.592615	-3.140185	-1.317570
O	0.812714	-0.395941	-0.230915
C	-0.358401	-3.042622	1.425498
H	-1.358834	-3.440506	1.577564
H	-0.275088	-2.062748	1.908132
H	0.337689	-3.732538	1.900961
H	-0.743550	-0.085749	-0.893267
H	-2.389833	-1.847239	0.479131
C	-3.171615	-3.580642	-0.576530
H	-2.753723	-4.328603	0.096180
H	-3.145671	-3.986337	-1.589883
H	-4.206662	-3.396219	-0.284589

ASG_0.00_NcA.xyz
31
0 eng= -779.389632292 zpe= -779.12433

N	-0.198864	-1.627974	-3.594239
C	-0.608416	-0.544297	-2.635687
C	-1.612864	-1.259398	-1.710659
N	-1.421940	-1.220472	-0.370804
C	-0.115886	-0.935535	0.186033
C	0.975550	-1.804844	-0.453221
N	2.245189	-1.434156	-0.195755
C	3.345108	-2.077943	-0.872722
C	3.358333	-1.772646	-2.363222
O	4.461360	-2.246828	-2.920302
H	4.461114	-2.059747	-3.870441
O	-2.544131	-1.812774	-2.261266
H	0.439608	-1.274843	-4.309769
H	-1.048167	-2.027557	-4.011442
H	-0.149692	-1.144169	1.257127

H	0.149053	0.121049	0.078410
O	0.703887	-2.738872	-1.191231
H	2.426905	-0.622754	0.375769
H	4.291234	-1.760970	-0.435066
H	3.277705	-3.163993	-0.765950
O	2.483653	-1.200208	-2.966192
H	0.292322	-2.358508	-3.053520
C	-2.360565	-1.982960	0.458293
H	-2.027019	-3.019795	0.556077
H	-3.337721	-1.968838	-0.016573
H	-2.425447	-1.516377	1.440220
H	0.314216	-0.211422	-2.166020
C	-1.284884	0.592386	-3.384646
H	-0.601912	1.049539	-4.103302
H	-1.588470	1.360988	-2.672508
H	-2.179883	0.233925	-3.895817

ASG_10.00_NcB.xyz
31
0 eng= -779.385666737 zpe= -779.12052

N	-2.803091	1.018544	-0.292505
C	-2.569799	-0.437139	-0.569186
C	-1.938792	-0.449699	-1.977639
N	-1.155601	-1.495002	-2.307838
C	-0.643824	-2.418277	-1.300447
C	0.236928	-1.736037	-0.250732
N	1.081587	-0.771696	-0.709139
C	1.813733	0.015564	0.245593
C	0.909037	0.969053	1.006103
O	1.552990	1.552163	1.996967
H	0.966296	2.162660	2.467007
O	-2.245652	0.459840	-2.731277
H	-3.570817	1.169572	0.362699
H	-3.010246	1.461210	-1.203826
H	-0.057565	-3.182515	-1.813487
H	-1.441140	-2.932137	-0.760666
O	0.161529	-2.031831	0.920604
H	1.028680	-0.465045	-1.668000
H	2.306250	-0.629535	0.975583
H	2.585157	0.601657	-0.256536
O	-0.251492	1.186029	0.735132
H	-1.933792	1.424402	0.091356
C	-0.688858	-1.638936	-3.689499
H	-0.956572	-2.629374	-4.059996
H	0.396846	-1.522995	-3.744944
H	-1.163615	-0.877552	-4.302341
H	-1.913933	-0.800768	0.219873
C	-3.897663	-1.186922	-0.595813
H	-3.726955	-2.234346	-0.846138
H	-4.562837	-0.763653	-1.351867

```
H       -4.382661       -1.153539       0.381604

ASG_15.13_NtA.xyz
31
0 eng= -779.384727873 zpe= -779.118567
C       -0.826974       1.967456        -0.854435
H       -1.824560       2.177346        -0.471097
C       -0.572090       0.458840        -1.002432
O       0.559049        0.059812        -0.743446
N       0.136584        2.363813        0.228165
H       0.251703        3.375105        0.300201
H       1.033083        1.902820        0.010464
N       -1.585111       -0.327465       -1.389153
C       -1.403106       -1.770301       -1.169493
H       -0.402725       -2.063184       -1.482181
H       -2.152492       -2.312926       -1.741108
C       -1.658053       -1.959787       0.323553
O       -2.783841       -1.964410       0.773687
N       -0.546933       -1.974250       1.113792
H       0.335471        -1.724303       0.688154
C       -0.734003       -1.738080       2.518751
H       -1.492145       -2.409877       2.922384
H       0.195655        -1.916023       3.063323
C       -1.175437       -0.310257       2.797366
O       -1.653416       -0.179408       4.018719
H       -1.919771       0.737570        4.178846
O       -1.078861       0.613860        2.013608
H       -0.190532       1.960650        1.123667
C       -0.518915       2.726454        -2.137260
H       0.500405        2.514092        -2.468216
H       -0.641895       3.802791        -2.002407
H       -1.198317       2.407259        -2.928290
C       -2.962603       0.144979        -1.550642
H       -3.476212       -0.535604       -2.227590
H       -2.978032       1.134233        -2.004671
H       -3.490235       0.147747        -0.593421

ASG_17.83_c-t_N-gcggtgtt.xyz
31
0 eng= -779.383049943 zpe= -779.11754
C       -2.995297       -0.997305       -0.569773
H       -2.569047       -1.762922       0.069948
C       -2.234480       -0.759483       -1.886645
O       -2.562042       0.215217        -2.535494
N       -2.815069       0.303946        0.165954
H       -3.378377       0.338895        1.017025
H       -3.095640       1.065035        -0.466699
N       -1.269194       -1.631758       -2.264234
C       -0.609666       -2.522459       -1.306786
H       0.126388        -3.111064       -1.854404
```

H	-1.312845	-3.218218	-0.850417
C	0.050444	-1.705209	-0.193333
O	-0.472957	-1.572215	0.899525
N	1.194780	-1.065011	-0.525877
H	1.595805	-1.186010	-1.442740
C	1.806431	-0.141692	0.397027
H	1.950682	-0.614810	1.373162
H	2.786054	0.161972	0.029307
C	0.963970	1.099424	0.630611
O	1.636153	1.989078	1.340114
H	1.084433	2.768791	1.501515
O	-0.169471	1.265219	0.245630
H	-1.816460	0.460899	0.402789
C	-4.470696	-1.237046	-0.847135
H	-4.587130	-2.176573	-1.388900
H	-4.883542	-0.436369	-1.462802
H	-5.033710	-1.317838	0.085015
C	-0.593848	-1.350243	-3.533127
H	-0.148277	-2.271008	-3.906294
H	0.174047	-0.579597	-3.411411
H	-1.325316	-0.984390	-4.249785

ASG_18.10_NcC.xyz
31
0 eng= -779.382753353 zpe= -779.117437

N	-2.816197	1.021152	-1.840950
C	-3.243471	0.376602	-0.550135
C	-2.017325	-0.484027	-0.193019
N	-1.470795	-0.400085	1.041510
C	-1.587617	0.801849	1.848685
C	-0.935006	2.004307	1.150507
N	-0.255177	2.848208	1.922554
C	0.360798	4.047731	1.392736
C	1.100658	4.728060	2.524281
O	1.693896	5.840229	2.109667
H	2.157668	6.249470	2.855170
H	-3.440273	1.190219	0.141408
O	-1.626282	-1.231698	-1.069806
H	-3.582771	1.494188	-2.321436
H	-2.428330	0.279977	-2.441379
H	-1.124777	0.606415	2.815725
H	-2.632207	1.051817	2.049750
O	-1.077226	2.173958	-0.060840
H	-0.112891	2.675275	2.912348
H	1.061218	3.807321	0.589043
H	-0.387241	4.731644	0.983751
O	1.133428	4.300468	3.644057
C	-4.469337	-0.494165	-0.770300
H	-4.746959	-0.970602	0.170862
H	-4.255765	-1.279096	-1.497607

H	-5.319184	0.102489	-1.108062
C	-0.288112	-1.230777	1.294827
H	-0.237397	-1.453343	2.359708
H	0.622959	-0.713454	0.978847
H	-0.379351	-2.155102	0.731454
H	-2.061874	1.688445	-1.602507

ASG_20.22_NcA.xyz
31
0 eng= -779.382396657 zpe= -779.11663

N	-1.665212	-0.365283	1.707471
C	-2.679542	-0.548192	0.610305
C	-1.804236	-0.465823	-0.655561
N	-1.879380	-1.434461	-1.596619
C	-2.297858	-2.785887	-1.263083
C	-1.272397	-3.419749	-0.315141
N	-0.828400	-4.647469	-0.618826
C	0.322357	-5.179499	0.075998
C	1.573707	-4.399653	-0.312229
O	2.629741	-4.844677	0.364017
H	3.414605	-4.350599	0.085791
H	-3.134466	-1.518266	0.785587
O	-1.089216	0.515823	-0.732565
H	-1.079727	0.442800	1.453460
H	-1.077710	-1.216949	1.714237
H	-2.391376	-3.347888	-2.192127
H	-3.279284	-2.801076	-0.786838
O	-0.892519	-2.807609	0.679225
H	-1.061413	-5.034480	-1.521198
H	0.181659	-5.105645	1.154776
H	0.458178	-6.231055	-0.176334
O	1.595522	-3.514689	-1.118727
H	-2.093477	-0.213282	2.621729
C	-3.701112	0.576268	0.636785
H	-4.412545	0.434124	-0.177759
H	-3.211683	1.541062	0.494223
H	-4.259878	0.579005	1.574884
C	-0.933740	-1.336117	-2.718333
H	-1.373528	-1.825675	-3.586584
H	0.015426	-1.815067	-2.455715
H	-0.758086	-0.287709	-2.940516

ASG_20.94_NcD.xyz
31
0 eng= -779.38194386 zpe= -779.116356

N	0.172895	-1.147876	-3.537052
C	-0.772329	-0.130012	-2.958839
C	-1.912916	-0.992407	-2.387482
N	-2.277532	-0.831529	-1.091787
C	-1.371198	-0.236653	-0.132288

C	0.009750	-0.903921	-0.167670
N	0.988257	-0.277157	0.521230
C	2.354613	-0.708201	0.362122
C	2.836037	-0.541126	-1.082693
O	4.064298	-0.976736	-1.337712
H	4.506945	-1.338010	-0.560002
H	-0.187447	0.421934	-2.227864
O	-2.437018	-1.769349	-3.160290
H	0.987257	-0.701778	-3.966521
H	-0.341547	-1.725065	-4.212565
H	-1.798593	-0.361800	0.864537
H	-1.258458	0.840031	-0.293733
O	0.222039	-1.912476	-0.820614
H	0.800514	0.607874	0.968170
H	2.996843	-0.128961	1.028174
H	2.447816	-1.764638	0.631659
O	2.160084	-0.069905	-1.956315
H	0.507640	-1.746794	-2.766011
C	-1.288852	0.787264	-4.055341
H	-0.469978	1.337751	-4.522447
H	-1.978578	1.512493	-3.621330
H	-1.831607	0.213839	-4.808619
C	-3.312952	-1.732948	-0.576842
H	-2.866642	-2.668804	-0.229043
H	-4.014514	-1.952161	-1.377051
H	-3.836247	-1.240594	0.241563

ASG_21.69_c-c_N-gcgtcgtt.xyz

31

0 eng= -779.381648059 zpe= -779.116068

N	-0.233271	-2.400045	-1.905317
C	-1.133963	-3.603890	-1.790919
C	-2.473341	-2.958737	-1.381174
N	-3.592651	-3.257667	-2.074809
C	-3.516546	-3.794543	-3.417312
C	-2.687390	-2.895170	-4.350779
N	-2.675631	-3.220468	-5.661811
C	-2.977558	-4.532510	-6.183463
C	-1.956851	-5.570934	-5.727284
O	-2.065745	-6.687492	-6.431440
H	-1.428111	-7.341892	-6.108985
O	-2.429898	-2.209102	-0.423701
H	-0.557670	-1.841693	-2.712413
H	0.750170	-2.651393	-2.015538
H	-4.533783	-3.872859	-3.805593
H	-3.095532	-4.802256	-3.418975
O	-2.080859	-1.926014	-3.918289
H	-2.077337	-2.625703	-6.222146
H	-3.969549	-4.873828	-5.873760
H	-2.994315	-4.497105	-7.272404

O	-1.180448	-5.399154	-4.824211
H	-0.363161	-1.839451	-1.050973
C	-4.817627	-2.541469	-1.705809
H	-5.677736	-3.167561	-1.939280
H	-4.883858	-1.594155	-2.248480
H	-4.795317	-2.336567	-0.639077
H	-1.126795	-4.089293	-2.765312
C	-0.626812	-4.535147	-0.702518
H	0.367020	-4.916760	-0.944724
H	-1.300997	-5.388591	-0.619482
H	-0.605375	-4.024204	0.261492

ASG_23.54_t-t_N-gtgttgtt.xyz
31
0 eng= -779.380913433 zpe= -779.115365

N	-0.891356	1.623541	0.227987
C	-0.616824	1.741092	-1.257641
C	-0.448320	0.253921	-1.614627
N	-1.414984	-0.388495	-2.316027
C	-1.633409	-1.743965	-1.825630
C	-1.950498	-1.624581	-0.325552
N	-1.502254	-2.599079	0.478512
C	-1.707328	-2.533412	1.905757
C	-0.929745	-1.411475	2.578741
O	-0.981702	-1.557111	3.897599
H	-0.501653	-0.832787	4.324276
H	-1.502092	2.198847	-1.694110
O	0.452393	-0.334313	-1.048396
H	-1.010689	2.534393	0.673102
H	-0.120958	1.108383	0.683930
H	-0.758994	-2.369992	-2.002364
H	-2.493127	-2.176022	-2.339324
O	-2.579088	-0.658765	0.097489
H	-0.865070	-3.287163	0.106499
H	-2.764692	-2.360292	2.124978
H	-1.427045	-3.481029	2.363980
O	-0.368112	-0.503285	2.023140
H	-1.722965	1.023727	0.393620
C	0.637738	2.557052	-1.495526
H	1.498584	2.059436	-1.046022
H	0.537516	3.565635	-1.089858
H	0.815852	2.642237	-2.568188
C	-2.528830	0.288577	-2.975486
H	-2.926892	-0.376223	-3.741047
H	-2.174494	1.183216	-3.484758
H	-3.326835	0.537475	-2.269299

ASG_25.45_c-t_N-tcggtgtc.xyz
31
0 eng= -779.379866035 zpe= -779.114635

N	-1.427939	0.255768	-0.179620
C	-2.208478	-0.848456	-0.829606
C	-1.793622	-0.770888	-2.313564
N	-1.889448	-1.890422	-3.060870
C	-2.093302	-3.208331	-2.467886
C	-0.929977	-3.643142	-1.574511
N	0.323719	-3.481820	-2.091802
C	1.429348	-3.562952	-1.175669
C	1.382825	-2.420696	-0.160055
O	2.208005	-2.491436	0.869279
H	2.715706	-3.312468	0.884749
O	-1.473250	0.324672	-2.742600
H	-1.871371	0.592748	0.675326
H	-1.356932	1.015516	-0.876745
H	-2.213532	-3.925223	-3.281949
H	-2.996112	-3.255137	-1.857080
O	-1.117409	-4.082547	-0.462187
H	0.436211	-2.914197	-2.918972
H	1.394605	-4.516214	-0.642469
H	2.372411	-3.508386	-1.723929
O	0.645296	-1.474987	-0.288858
H	-0.474441	-0.087451	0.027136
C	-1.689479	-1.805378	-4.510043
H	-2.566532	-2.204752	-5.021578
H	-0.812086	-2.382206	-4.814729
H	-1.546926	-0.764013	-4.785223
H	-1.920027	-1.773673	-0.333375
C	-3.703353	-0.573631	-0.701953
H	-4.270265	-1.352497	-1.212832
H	-3.958339	0.384281	-1.160910
H	-4.012070	-0.572085	0.345172

SAG_0.00_c-t_N-tccttgtt.xyz
31
0 eng= -779.390050804 zpe= -779.123791

N	0.873803	-1.269479	-2.810542
C	-0.269604	-0.337574	-2.603598
C	-1.522089	-1.224408	-2.577058
N	-2.422379	-1.049349	-1.581838
C	-2.092840	-0.533613	-0.264468
C	-0.772557	-1.152276	0.230250
N	-0.213367	-0.550812	1.300989
C	1.066731	-0.991268	1.797478
C	2.190624	-0.729212	0.809022
O	3.361374	-1.017028	1.357168
H	4.073620	-0.858431	0.720934
H	-0.056432	0.261027	-1.720591
H	-0.344016	0.307732	-3.480431
O	-1.647358	-2.014455	-3.486483
H	0.525736	-2.012722	-3.432448

H	-3.192936	-1.706752	-1.633747
O	-0.250011	-2.104240	-0.326373
H	-0.640708	0.271382	1.699520
H	1.296518	-0.490771	2.737655
H	1.054094	-2.067709	1.990730
O	2.050410	-0.335833	-0.323159
C	2.099222	-0.631621	-3.366604
H	2.410179	0.156732	-2.686049
H	1.862052	-0.231988	-4.350768
H	2.878301	-1.386977	-3.446702
H	1.065377	-1.699091	-1.892778
H	-1.963066	0.553579	-0.314120
C	-3.235657	-0.855263	0.699569
H	-3.045920	-0.434181	1.686636
H	-3.349743	-1.936639	0.803088
H	-4.167061	-0.431760	0.321901

SAG_3.96_NtA.xyz
31
0 eng= -779.388237099 zpe= -779.122282

N	-1.711563	-0.244049	-4.042819
C	-2.358534	-1.298605	-3.214911
C	-1.786613	-1.110133	-1.807386
N	-1.690302	-2.204842	-1.043416
C	-0.834509	-2.210782	0.156483
C	0.582393	-2.309543	-0.427178
N	1.208933	-1.119749	-0.652300
C	2.332236	-1.123639	-1.548639
C	1.913658	-1.430211	-2.977771
O	2.945542	-1.775073	-3.722213
H	2.659044	-1.959278	-4.628218
H	-3.437445	-1.132560	-3.219793
H	-2.126330	-2.271661	-3.647059
O	-1.444446	0.013831	-1.476776
H	-0.710049	-0.495742	-4.103050
H	-1.858137	-3.109856	-1.465078
H	-0.988433	-1.260445	0.668978
O	1.052219	-3.370790	-0.773459
H	0.672827	-0.270235	-0.540137
H	2.826846	-0.150143	-1.544376
H	3.063419	-1.872921	-1.243754
O	0.779282	-1.342560	-3.405590
H	-1.732584	0.611816	-3.469934
C	-2.314854	-0.041384	-5.387458
H	-2.254576	-0.975822	-5.941957
H	-1.761254	0.737535	-5.907247
H	-3.354748	0.255497	-5.265114
C	-1.171026	-3.390336	1.046166
H	-0.540560	-3.373456	1.935340
H	-0.978415	-4.327173	0.520311

H	-2.215210	-3.345394	1.356100

SAG_4.62_NcB.xyz
31
0 eng= -779.38768191 zpe= -779.122031

N	-2.311363	-0.065718	0.172969
C	-2.263099	-1.008333	-0.975199
C	-3.624641	-0.893142	-1.676554
N	-3.686824	-1.253556	-2.973425
C	-2.535077	-1.488960	-3.844490
C	-1.632480	-0.249677	-3.912693
N	-2.260958	0.948513	-4.057019
C	-1.481591	2.154184	-3.989106
C	-0.978093	2.429539	-2.582952
O	-0.128738	3.438030	-2.571334
H	0.176369	3.610050	-1.668708
H	-1.395757	-0.759923	-1.584936
H	-2.160642	-2.025962	-0.589430
O	-4.575756	-0.523757	-1.016485
H	-3.291711	-0.052964	0.492639
H	-4.619580	-1.275270	-3.369960
H	-1.908352	-2.284986	-3.434790
O	-0.427209	-0.344321	-3.827178
H	-3.266435	1.000894	-4.002701
H	-0.610334	2.085144	-4.643633
H	-2.075614	3.010202	-4.313303
O	-1.314833	1.821207	-1.590780
H	-2.107122	0.866695	-0.218309
C	-1.357789	-0.377815	1.272579
H	-1.610380	-1.350732	1.689627
H	-1.441030	0.391387	2.037250
H	-0.349885	-0.391685	0.862124
C	-3.014969	-1.892770	-5.234385
H	-2.156288	-2.059174	-5.884489
H	-3.634981	-1.109514	-5.677056
H	-3.592817	-2.816496	-5.180070

SAG_5.72_NcA.xyz
31
0 eng= -779.387926328 zpe= -779.121614

N	-3.023673	0.394142	-1.544491
C	-3.455647	-1.011265	-1.247228
C	-2.677065	-1.956623	-2.170138
N	-1.885411	-2.897264	-1.595728
C	-1.387074	-2.893475	-0.234101
C	-0.847879	-1.504175	0.141774
N	-0.689554	-1.285069	1.464143
C	-0.291530	0.012044	1.950508
C	-1.378355	1.057500	1.760215
O	-1.034904	2.195336	2.339967

H	-1.731895	2.856360	2.216889
H	-3.311175	-1.171137	-0.180627
H	-4.515786	-1.096872	-1.486638
O	-2.811302	-1.838118	-3.367093
H	-3.202354	0.952383	-0.702245
H	-1.372486	-3.447608	-2.274896
O	-0.598199	-0.648544	-0.692779
H	-0.970598	-1.992274	2.126592
H	-0.035968	-0.044924	3.008416
H	0.593922	0.365476	1.415267
O	-2.408669	0.889351	1.152542
C	-3.650548	1.016584	-2.750386
H	-4.725600	1.064716	-2.587080
H	-3.425103	0.394981	-3.612342
H	-3.241481	2.017888	-2.868886
H	-1.994993	0.372252	-1.618888
H	-2.198225	-3.143693	0.459726
C	-0.281214	-3.943241	-0.103701
H	0.085691	-3.998480	0.920998
H	0.556526	-3.690782	-0.757155
H	-0.668928	-4.924343	-0.380530

SAG_7.01_c-t_N-tccttgtt.xyz
31
0 eng= -779.387290316 zpe= -779.121121

C	-0.651190	0.602242	-1.948095
H	-0.925031	1.206653	-2.814550
H	0.337037	0.172918	-2.092843
C	-1.815543	-0.358380	-1.672219
O	-2.859283	0.146689	-1.320136
N	-0.583730	1.497108	-0.759029
H	-1.555878	1.627580	-0.445248
N	-1.641204	-1.685481	-1.870658
H	-2.471051	-2.216871	-1.636865
C	-0.364603	-2.384428	-1.786267
H	-0.607607	-3.403497	-1.470111
C	0.472301	-1.797680	-0.629620
O	-0.032881	-1.113192	0.245959
N	1.790944	-2.081975	-0.634891
H	2.199384	-2.602165	-1.394380
C	2.646591	-1.568083	0.406284
H	2.240980	-1.811276	1.392519
H	3.634806	-2.021407	0.333948
C	2.791678	-0.057219	0.345019
O	3.690275	0.353679	1.227076
H	3.774022	1.317388	1.190221
O	2.170198	0.678366	-0.382252
H	-0.089798	0.963465	-0.027815
C	0.081092	2.806573	-1.006473
H	1.088291	2.613248	-1.366031

H	0.116513	3.360586	-0.070666
H	-0.502422	3.353552	-1.744562
C	0.358054	-2.470604	-3.134301
H	1.177980	-3.191172	-3.107487
H	0.754972	-1.505801	-3.455878
H	-0.347431	-2.816771	-3.888838

SAG_8.65_NcB.xyz
31
0 eng= -779.386587314 zpe= -779.120497

C	-1.764955	-0.986931	-2.189474
H	-2.588201	-1.585725	-2.586301
H	-0.920489	-1.632831	-1.953950
C	-2.307754	-0.136038	-1.032571
O	-2.769348	0.954747	-1.308824
N	-1.336584	-0.024445	-3.239110
H	-0.415740	0.330096	-2.940633
N	-2.281222	-0.645347	0.215177
H	-2.770744	-0.078909	0.897003
C	-1.591304	-1.860085	0.666177
H	-1.619104	-1.815381	1.758291
C	-0.104537	-1.808945	0.264454
O	0.443490	-2.732184	-0.297106
N	0.550592	-0.654350	0.561542
H	0.053558	0.127773	0.957736
C	1.924923	-0.508777	0.165363
H	2.362208	0.372307	0.638016
H	2.510264	-1.377750	0.473434
C	2.078504	-0.363402	-1.337680
O	3.345459	-0.447112	-1.694111
H	3.431899	-0.340565	-2.652530
O	1.171322	-0.178328	-2.118550
H	-2.000901	0.762360	-3.199569
C	-1.221898	-0.612203	-4.601590
H	-0.886905	0.160715	-5.289858
H	-2.196165	-0.990269	-4.905132
H	-0.495222	-1.421559	-4.563835
C	-2.250658	-3.154355	0.211801
H	-1.739401	-4.005033	0.661163
H	-2.191578	-3.280644	-0.869925
H	-3.296783	-3.160817	0.517952

SAG_11.96_t-t_N-gtgttgtt.xyz
31
0 eng= -779.385087409 zpe= -779.119234

C	-2.068577	0.755513	-2.612157
H	-2.297647	1.790423	-2.862679
H	-2.813690	0.080843	-3.032987
C	-1.824641	0.598689	-1.111808
O	-1.023971	1.340388	-0.590081

N	-0.730183	0.413041	-3.194297
H	-0.017729	0.993680	-2.724055
N	-2.339096	-0.502103	-0.499832
H	-2.856181	-1.148783	-1.080801
C	-1.444205	-1.135667	0.468453
H	-1.279617	-0.440645	1.293011
C	-0.125643	-1.389744	-0.287390
O	-0.145836	-1.785738	-1.449392
N	1.017779	-1.156325	0.374300
H	0.977733	-0.685819	1.266132
C	2.298081	-1.330684	-0.267875
H	3.095117	-1.273719	0.472372
H	2.351226	-2.318829	-0.734054
C	2.572573	-0.315164	-1.367059
O	3.849694	-0.373528	-1.724602
H	4.019205	0.260658	-2.436042
O	1.765567	0.420137	-1.875746
H	-0.475266	-0.543822	-2.882658
C	-0.639637	0.547096	-4.672153
H	0.371598	0.290002	-4.980298
H	-0.865532	1.576184	-4.944878
H	-1.355995	-0.130579	-5.132421
C	-2.036928	-2.446257	0.967662
H	-1.364835	-2.916620	1.685942
H	-2.185629	-3.138760	0.135885
H	-2.992849	-2.259773	1.457651

SAG_12.02_NcA.xyz
31
0 eng= -779.385144507 zpe= -779.119214

N	-1.480495	0.089277	-3.061719
C	-1.084834	-0.768953	-1.897075
C	-2.345449	-1.435285	-1.336692
N	-2.719239	-1.148379	-0.064617
C	-2.258252	-0.061683	0.785977
C	-2.173587	1.248165	-0.020688
N	-1.366592	2.208990	0.473940
C	-1.216099	3.464893	-0.218311
C	-0.429484	3.326521	-1.510724
O	-0.183390	4.516167	-2.033062
H	0.312066	4.420913	-2.859688
H	-0.420361	-1.551295	-2.264435
H	-0.544615	-0.128677	-1.203400
O	-2.956567	-2.201590	-2.047686
H	-0.741575	0.790936	-3.183216
H	-3.578657	-1.613622	0.199333
H	-3.069412	0.113207	1.500011
O	-2.813442	1.414726	-1.048105
H	-0.800397	2.028066	1.287103
H	-2.195408	3.874337	-0.481403

```
H       -0.716259       4.188986        0.424638
O       -0.087022       2.281964        -2.011097
C       -1.751059       -0.636576       -4.340219
H       -0.839461       -1.151147       -4.638623
H       -2.033998       0.096435        -5.092900
H       -2.549401       -1.352887       -4.168849
C       -1.000419       -0.423901       1.583031
H       -0.812496       0.289477        2.388401
H       -0.111059       -0.483749       0.951743
H       -1.149549       -1.397051       2.049492
H       -2.298636       0.637149        -2.751797

SAG_12.84_c-t_N-gcggtgtt.xyz
31
0 eng= -779.384995671 zpe= -779.118902
N       -1.693081       -0.801468       -0.876112
C       -1.489947       -1.603013       -2.118748
C       -0.761657       -0.676148       -3.094806
N       0.480854        -1.026669       -3.513004
C       1.370962        -2.014280       -2.895803
C       1.601500        -1.612698       -1.428333
N       2.552976        -0.688576       -1.183954
C       2.729799        -0.148829       0.141567
C       1.560636        0.711572        0.586939
O       1.867411        1.381933        1.684574
H       1.108017        1.910513        1.971481
H       -0.967520       -2.512240       -1.839753
H       -2.470249       -1.828514       -2.539166
O       -1.328007       0.339018        -3.432453
H       -0.771970       -0.469502       -0.535247
H       0.897371        -0.352484       -4.146025
H       0.871702        -2.984124       -2.859938
O       0.893875        -2.062344       -0.538136
H       3.098459        -0.310797       -1.942387
H       2.824140        -0.958079       0.872059
H       3.642949        0.443617        0.184884
O       0.484938        0.772532        0.040737
C       -2.372976       -1.552714       0.214530
H       -2.527357       -0.881915       1.056989
H       -1.721136       -2.375631       0.502481
H       -3.327217       -1.925791       -0.152813
C       2.635339        -2.144122       -3.733249
H       3.339232        -2.827241       -3.257722
H       3.131508        -1.180115       -3.879225
H       2.383092        -2.536663       -4.718740
H       -2.212107       0.044695        -1.136435

SAG_13.48_c-t_N-tcgttgtt.xyz
31
0 eng= -779.384692996 zpe= -779.118658
```

N	-0.218972	-1.045655	-4.321420
C	-1.061697	-0.718837	-3.134946
C	-1.336645	-2.075077	-2.465259
N	-1.684103	-2.083280	-1.166085
C	-1.428908	-1.010180	-0.222228
C	0.061371	-0.621191	-0.250435
N	0.367227	0.514849	0.422341
C	1.674987	1.100005	0.272031
C	1.898270	1.629310	-1.136100
O	3.080365	2.225785	-1.227189
H	3.203994	2.569919	-2.123285
H	-0.531899	0.019231	-2.534831
H	-2.010760	-0.308333	-3.486977
O	-1.294209	-3.063716	-3.172240
H	-0.318995	-0.329954	-5.041739
H	-1.819431	-3.015028	-0.790249
H	-2.013004	-0.128239	-0.510736
O	0.890073	-1.267542	-0.858790
H	-0.375481	1.081035	0.803914
H	1.809570	1.914101	0.984263
H	2.449479	0.355511	0.470302
O	1.123178	1.519717	-2.052589
H	-0.593998	-1.941723	-4.675626
C	1.228827	-1.239835	-3.978985
H	1.599479	-0.309844	-3.554020
H	1.762295	-1.508617	-4.888089
H	1.290213	-2.035941	-3.239956
C	-1.858592	-1.460136	1.174024
H	-1.715565	-0.663681	1.903594
H	-1.264608	-2.320475	1.490771
H	-2.913698	-1.735555	1.168975

GSA_0.00_NcA.xyz
31
0 eng= -779.386675515 zpe= -779.1208

N	1.019874	0.021414	-3.232454
C	0.013798	-1.053181	-2.976840
C	-0.814720	-0.520146	-1.797778
N	-0.968829	-1.292647	-0.699119
C	-0.049055	-2.376612	-0.420664
C	1.414498	-1.925302	-0.534204
N	2.329359	-2.910289	-0.547800
C	3.735454	-2.638400	-0.780809
C	3.940636	-2.079176	-2.188321
O	5.226934	-1.856976	-2.419361
H	5.344605	-1.503632	-3.312962
H	-0.641800	-1.124564	-3.844275
H	0.560297	-1.981923	-2.843464
O	-1.285287	0.589820	-1.944075
H	1.596375	-0.175630	-4.052133

H	1.639458	0.058778	-2.404544
H	-0.221490	-3.233267	-1.079779
H	-0.231107	-2.721926	0.598777
O	1.713765	-0.746018	-0.654148
H	2.031607	-3.874425	-0.495162
H	4.057984	-1.845693	-0.098036
O	3.066128	-1.846062	-2.988171
H	0.524305	0.916353	-3.323407
C	4.567398	-3.897117	-0.545840
H	4.421954	-4.246087	0.476809
H	5.623579	-3.680840	-0.692565
H	4.278166	-4.691008	-1.238983
C	-1.695423	-0.705637	0.430988
H	-2.160101	-1.505294	1.006085
H	-2.463820	-0.041239	0.045236
H	-1.013349	-0.132856	1.065246

GSA_5.39_NcA.xyz
31
0 eng= -779.384911572 zpe= -779.118746

N	-0.843386	-1.786864	-1.781913
C	-1.135120	-0.323972	-1.860271
C	-2.668847	-0.251630	-1.927776
N	-3.329181	0.547495	-1.060298
C	-2.704360	0.971494	0.175888
C	-2.077762	-0.212529	0.931186
N	-1.266467	0.121319	1.951221
C	-0.484166	-0.860571	2.688071
C	0.482606	-1.579822	1.748741
O	1.226866	-2.444433	2.423939
H	1.858977	-2.869471	1.826405
H	-0.633068	0.148508	-1.020911
H	-0.737365	0.057183	-2.800469
O	-3.190156	-0.936537	-2.784718
H	0.156855	-1.986906	-1.828051
H	-1.359225	-2.260835	-2.533536
H	-3.472074	1.424905	0.805858
H	-1.944365	1.739655	0.002033
O	-2.268394	-1.365944	0.575148
H	-1.129902	1.098169	2.164990
H	0.147763	-0.301473	3.383599
O	0.586899	-1.393823	0.560076
H	-1.194499	-2.115863	-0.866732
C	-1.342503	-1.843452	3.493026
H	-1.984791	-1.280380	4.169961
H	-0.699961	-2.497339	4.080931
H	-1.963512	-2.446131	2.831897
C	-4.794152	0.516448	-1.104867
H	-5.176734	-0.276755	-0.456565
H	-5.109318	0.322431	-2.126429

H	-5.179017	1.482731	-0.781679

GSA_8.45_NcB.xyz
31
0 eng= -779.38317517 zpe= -779.117581

C	-2.287125	-0.057944	0.936414
H	-1.410370	-0.517787	1.385265
H	-3.193178	-0.508737	1.345814
C	-2.374139	-0.117259	-0.600962
O	-2.861870	0.849213	-1.163390
N	-2.272142	1.395204	1.267018
H	-2.783010	1.874121	0.504232
H	-1.286647	1.708719	1.262579
N	-1.958485	-1.242649	-1.207609
C	-1.159323	-2.237150	-0.498207
H	-0.944728	-3.052566	-1.190786
H	-1.691076	-2.667626	0.352345
C	0.160821	-1.670755	0.034392
O	0.587832	-1.994795	1.120912
N	0.793202	-0.765800	-0.758498
H	0.360012	-0.436432	-1.608442
C	1.963072	-0.079456	-0.260986
H	2.669330	-0.815187	0.130423
C	1.583650	0.823927	0.905897
O	2.642214	1.171632	1.610094
H	2.385862	1.764677	2.331499
O	0.461018	1.216034	1.146688
H	-2.693471	1.605777	2.171365
C	2.619180	0.751996	-1.363121
H	2.918365	0.098576	-2.182942
H	3.506846	1.251991	-0.978015
H	1.929235	1.509227	-1.744259
C	-2.170917	-1.408221	-2.647823
H	-2.670513	-2.360301	-2.830601
H	-1.217252	-1.400596	-3.182585
H	-2.792230	-0.592772	-3.008144

GSA_12.60_NtA.xyz
31
0 eng= -779.381918621 zpe= -779.116001

N	-1.139267	0.713246	0.255847
C	-1.059378	1.656132	-0.897847
C	-0.065876	0.985188	-1.856747
N	-0.149618	1.283680	-3.158655
C	0.458333	0.314252	-4.082857
C	-0.532628	-0.848049	-4.101234
N	-0.242678	-1.887732	-3.272235
C	-1.307445	-2.802286	-2.934769
C	-2.423054	-2.063743	-2.202193
O	-3.583098	-2.674818	-2.333428

H	-4.268053	-2.202940	-1.837794
H	-2.055120	1.752541	-1.324855
H	-0.696907	2.624491	-0.550345
O	0.714524	0.173954	-1.372242
H	-1.555250	1.125642	1.090432
H	-0.182134	0.374653	0.438491
H	1.449956	0.040912	-3.727009
H	0.520869	0.760592	-5.072591
O	-1.578302	-0.770470	-4.712478
H	0.523816	-1.778971	-2.620474
H	-1.742691	-3.200256	-3.852936
O	-2.273197	-1.061415	-1.526786
H	-1.681684	-0.110203	-0.061687
C	-0.794266	-3.939655	-2.052280
H	-1.606168	-4.621307	-1.799866
H	-0.361123	-3.548917	-1.127782
H	-0.027683	-4.496775	-2.590643
C	-1.169425	2.181034	-3.705909
H	-0.810339	2.559485	-4.661173
H	-1.312921	3.037168	-3.047840
H	-2.111424	1.651511	-3.872477

GSA_14.54_NcB.xyz
31
0 eng= -779.380828016 zpe= -779.115263

N	0.478369	-0.203070	-3.461888
C	-0.785024	-0.256389	-2.673357
C	-0.343250	-0.559733	-1.228307
N	-1.244517	-1.119227	-0.401882
C	-2.465552	-1.734634	-0.914886
C	-2.184948	-2.864945	-1.911205
N	-1.133013	-3.672346	-1.621576
C	-0.705503	-4.681100	-2.576365
C	-0.368583	-3.993577	-3.900158
O	-0.483081	-4.799287	-4.934786
H	-0.223068	-4.334445	-5.743819
H	-1.254152	0.729283	-2.681876
H	-1.444951	-0.984614	-3.138886
O	0.787337	-0.224494	-0.916703
H	0.672706	-1.158611	-3.806687
H	1.223938	0.082073	-2.801896
H	-3.113417	-1.015284	-1.418878
H	-3.022806	-2.132981	-0.065513
O	-2.844029	-2.984001	-2.922297
H	-0.555984	-3.478746	-0.818108
H	0.254989	-5.067416	-2.218539
O	0.024412	-2.848524	-3.992254
H	0.432273	0.440716	-4.251221
C	-1.688869	-5.842273	-2.713913
H	-1.282632	-6.603604	-3.378127

H	-2.640160	-5.494338	-3.113329
H	-1.850374	-6.284768	-1.731077
C	-0.924829	-1.268684	1.020196
H	0.000581	-0.738844	1.229626
H	-0.804601	-2.323464	1.282346
H	-1.734255	-0.846225	1.616571

GSA_16.10_c-t_O1-tcgttgtt.xyz
31
0 eng= -779.378381439 zpe= -779.114666

C	-1.769518	-1.200562	-1.962934
H	-1.749313	-2.291053	-1.885532
H	-0.755309	-0.833848	-1.783498
C	-2.709004	-0.661431	-0.889577
O	-3.734206	-0.022440	-1.323724
N	-2.312104	-0.757882	-3.240100
H	-1.690361	-0.119628	-3.722353
H	-2.534554	-1.523062	-3.864513
N	-2.536764	-0.846320	0.388237
C	-1.267793	-1.373546	0.868918
H	-0.993668	-2.277713	0.320511
H	-1.382153	-1.643053	1.919310
C	-0.171238	-0.306893	0.686207
O	-0.431770	0.766008	0.188799
N	1.065603	-0.680724	1.079690
H	1.223515	-1.643256	1.345575
C	2.212807	0.070353	0.602023
H	2.068055	1.123845	0.853114
C	2.264553	-0.015902	-0.924305
O	3.249026	0.733800	-1.408458
H	3.263375	0.657862	-2.373358
O	1.521879	-0.676132	-1.604074
H	-3.613276	-0.040805	-2.326822
C	3.501933	-0.448478	1.232113
H	3.445465	-0.355966	2.316827
H	4.349947	0.132627	0.873819
H	3.669143	-1.497025	0.971825
C	-3.411934	-0.197243	1.376028
H	-3.592060	-0.898005	2.189685
H	-4.353017	0.072952	0.906776
H	-2.911208	0.698880	1.748086

GSA_17.08_NcC.xyz
31
0 eng= -779.379751299 zpe= -779.114295

C	-3.217301	-1.217078	-1.597111
H	-4.306524	-1.219708	-1.636293
H	-2.859416	-1.793461	-0.751550
C	-2.734776	0.235747	-1.710148
O	-2.981794	0.782650	-2.768536

N	-2.693518	-1.850874	-2.845645
H	-2.895345	-1.207032	-3.624097
H	-3.092181	-2.770316	-3.037427
N	-2.100282	0.817161	-0.670750
C	-1.380172	0.031841	0.316091
H	-2.043408	-0.662840	0.837838
H	-0.992627	0.713724	1.072487
C	-0.248507	-0.782532	-0.330525
O	-0.417455	-1.318538	-1.428360
N	0.871693	-0.899835	0.375312
H	0.968188	-0.465092	1.287515
C	1.997365	-1.716554	-0.060736
H	1.621601	-2.685102	-0.401538
C	2.862239	-1.937468	1.169033
O	3.849092	-2.791804	0.922199
H	4.392552	-2.884659	1.718737
O	2.678999	-1.384957	2.218686
H	-1.667029	-1.914287	-2.723806
C	2.799981	-1.049572	-1.181122
H	3.622607	-1.697116	-1.483302
H	3.206254	-0.095215	-0.840761
H	2.149756	-0.877421	-2.038198
C	-1.638165	2.195586	-0.869923
H	-2.346050	2.715195	-1.509653
H	-0.654388	2.208308	-1.348658
H	-1.586436	2.690822	0.098480

GSA_17.37_c-t_N-gcggtgtt.xyz
31
0 eng= -779.379910768 zpe= -779.114186

C	-3.058674	-1.050368	-0.572665
H	-2.744869	-1.894024	0.030961
H	-4.111872	-1.124904	-0.841813
C	-2.276752	-0.778631	-1.865323
O	-2.590004	0.221766	-2.478422
N	-2.870738	0.189090	0.242595
H	-3.502233	0.230120	1.043397
H	-3.024744	1.003408	-0.367431
N	-1.306390	-1.640956	-2.250365
C	-0.646866	-2.534151	-1.294134
H	0.100114	-3.110823	-1.839119
H	-1.349160	-3.238944	-0.850644
C	-0.015840	-1.704144	-0.173723
O	-0.602175	-1.517685	0.883059
N	1.159551	-1.112485	-0.462681
H	1.601862	-1.279789	-1.354698
C	1.763378	-0.143582	0.431480
H	1.814464	-0.579496	1.435403
C	0.866544	1.084232	0.569162
O	1.450196	2.018849	1.304425

H	0.859656	2.781412	1.390547
O	-0.240991	1.201684	0.099935
H	-1.889941	0.236918	0.571351
C	3.165813	0.222987	-0.047528
H	3.779829	-0.676239	-0.104522
H	3.630896	0.917004	0.649407
H	3.131042	0.695292	-1.032346
C	-0.600083	-1.311834	-3.490667
H	-0.139649	-2.217223	-3.882923
H	0.158912	-0.541662	-3.320284
H	-1.315653	-0.926385	-4.213085

GSA_18.32_c-t_N-gcggtgtt.xyz
31
0 eng= -779.379932275 zpe= -779.113824

N	-1.553206	1.605168	0.693020
C	-2.087147	0.207565	0.706178
C	-2.531252	-0.062069	-0.738409
N	-1.981192	-1.093146	-1.426535
C	-0.664654	-1.634431	-1.078164
C	0.367036	-0.509145	-1.156836
N	0.811829	-0.195325	-2.386436
C	1.569837	1.016038	-2.637423
C	0.716467	2.242150	-2.315003
O	1.349108	3.360453	-2.643431
H	0.796888	4.124242	-2.421406
H	-1.316888	-0.435566	1.117114
H	-2.984862	0.189797	1.322769
O	-3.378533	0.682974	-1.184301
H	-2.156320	2.184545	0.092946
H	-0.610202	1.588185	0.267505
H	-0.441209	-2.442703	-1.773989
H	-0.652397	-2.051008	-0.071990
O	0.677303	0.132291	-0.159758
H	0.507669	-0.739116	-3.181310
H	2.412210	1.051673	-1.938310
O	-0.377432	2.217563	-1.804996
H	-1.501590	2.011733	1.627736
C	2.085105	1.035641	-4.073993
H	2.709838	0.159891	-4.251952
H	2.682226	1.928975	-4.245197
H	1.256122	1.034568	-4.786118
C	-2.453476	-1.265679	-2.802461
H	-2.203124	-2.271698	-3.135617
H	-2.007874	-0.519922	-3.468784
H	-3.532891	-1.135112	-2.825435

GSA_18.71_NcC.xyz
31
0 eng= -779.379616253 zpe= -779.113673

N	-3.160658	0.694375	1.174906
C	-3.243737	-0.356675	0.115265
C	-2.305565	0.169948	-0.979641
N	-1.356279	-0.637345	-1.499658
C	-0.808450	-1.751607	-0.744653
C	-0.128833	-1.267482	0.545563
N	1.017789	-1.855881	0.869927
C	1.758487	-1.523814	2.080345
C	3.168873	-2.049851	1.871916
O	3.972677	-1.691115	2.866649
H	4.853336	-2.063604	2.711288
H	-2.992237	-1.310140	0.565976
H	-4.258332	-0.371181	-0.282432
O	-2.502985	1.320994	-1.320112
H	-3.877900	0.607590	1.895351
H	-2.213408	0.607035	1.585711
H	-0.107128	-2.282463	-1.387824
H	-1.584145	-2.470798	-0.470988
O	-0.662541	-0.396480	1.237173
H	1.472061	-2.508745	0.239293
H	1.802838	-0.435534	2.177821
O	3.495632	-2.723009	0.933458
C	1.129078	-2.130230	3.336913
H	1.098142	-3.218733	3.260434
H	0.115259	-1.748920	3.454669
H	1.714370	-1.853391	4.213333
C	-0.474423	-0.047703	-2.513808
H	-0.117037	-0.839331	-3.170588
H	0.374183	0.456025	-2.041354
H	-1.038322	0.680308	-3.090261
H	-3.236238	1.609198	0.707558

AGS_0.00_NcA.xyz
31
0 eng= -779.386490698 zpe= -779.120909

N	-1.698236	-0.890207	-3.041382
C	-1.967698	0.383504	-2.290204
C	-2.269094	-0.104140	-0.861664
N	-1.537471	0.407495	0.159619
C	-0.222052	0.982723	-0.008741
C	0.690428	0.071816	-0.848721
N	1.874476	0.574295	-1.248019
C	2.619784	-0.205392	-2.214375
C	1.950209	-0.224475	-3.580532
O	2.765771	-0.736406	-4.490002
H	2.327620	-0.756133	-5.353322
O	-3.166639	-0.904317	-0.726614
H	-1.522715	-0.710862	-4.031775
H	-2.498336	-1.521477	-2.915784
H	-1.757229	-0.014339	1.053421

H	0.213822	1.132455	0.980459
H	-0.283824	1.970272	-0.474726
O	0.314745	-1.048325	-1.179806
H	3.628372	0.193726	-2.317568
H	2.702045	-1.244599	-1.885474
O	0.824433	0.145669	-3.811615
H	-0.852753	-1.320043	-2.625583
C	2.316704	1.941732	-0.978167
H	1.942454	2.283903	-0.016004
H	1.989715	2.628671	-1.764458
H	3.404478	1.954285	-0.925810
H	-1.059657	0.974909	-2.387118
C	-3.169572	1.100837	-2.881150
H	-2.987331	1.374218	-3.922257
H	-3.356761	2.016464	-2.318778
H	-4.059854	0.473722	-2.810413

AGS_12.09_c-t_N-tcggtgtt.xyz
31
0 eng= -779.382064408 zpe= -779.116306

N	-2.961469	2.127067	0.593272
C	-2.160446	0.892757	0.299238
C	-2.587948	0.516561	-1.129073
N	-1.727041	-0.202882	-1.875664
C	-0.294475	-0.335964	-1.634040
C	0.410278	1.023086	-1.546536
N	0.345196	1.856708	-2.618599
C	0.785039	3.210091	-2.376979
C	-0.161706	3.960458	-1.457313
O	0.271275	5.185875	-1.228331
H	-0.341948	5.657748	-0.646811
O	-3.710325	0.817546	-1.488380
H	-3.883328	1.998986	0.145008
H	-2.497917	2.935309	0.146138
H	-2.103278	-0.487197	-2.771125
H	0.123394	-0.954739	-2.427960
H	-0.096195	-0.841208	-0.687862
O	0.951560	1.352661	-0.506308
H	1.769740	3.222243	-1.904430
H	0.856108	3.758653	-3.317377
O	-1.184410	3.506659	-0.991992
H	-3.068217	2.290795	1.594750
C	-0.367217	1.574254	-3.859734
H	-0.323113	0.510981	-4.088669
H	0.130463	2.096496	-4.676383
H	-1.411941	1.896995	-3.810251
H	-1.108292	1.162828	0.384097
C	-2.540188	-0.227086	1.261408
H	-1.983282	-1.130221	1.008860
H	-3.605966	-0.455532	1.189049

H   -2.290068   0.039843   2.289907

AGS_12.33_c-c_N-gcgtcgtt.xyz
31
0 eng= -779.381623497 zpe= -779.116214

N	-1.548364	-3.469893	0.938848
C	-2.160709	-2.744398	-0.230885
C	-2.053473	-1.272343	0.209605
N	-1.488461	-0.380468	-0.635469
C	-0.562797	-0.737848	-1.686678
C	0.587224	-1.623955	-1.165186
N	1.580032	-1.921479	-2.030037
C	1.362404	-1.862060	-3.458993
C	0.401920	-2.960572	-3.909092
O	0.458350	-3.147957	-5.218960
H	-0.180343	-3.828579	-5.479552
O	-2.511474	-0.990183	1.295746
H	-1.683092	-4.480760	0.888591
H	-1.982225	-3.099786	1.796085
H	-1.410516	0.546779	-0.235583
H	-0.156217	0.185126	-2.102317
H	-1.083177	-1.253646	-2.497151
O	0.580853	-2.022689	-0.005849
H	0.952044	-0.900600	-3.777985
H	2.312970	-1.977451	-3.979662
O	-0.331579	-3.551855	-3.159654
H	-0.540324	-3.236480	0.953412
C	2.628958	-2.836229	-1.574213
H	3.525108	-2.663505	-2.168215
H	2.308713	-3.877210	-1.683828
H	2.844348	-2.636237	-0.528451
H	-1.564118	-3.004520	-1.104817
C	-3.614710	-3.151473	-0.402279
H	-3.698413	-4.215760	-0.630789
H	-4.046640	-2.596025	-1.235707
H	-4.188892	-2.916198	0.495494

AGS_14.89_t-t_N-gtgttgtt.xyz
31
0 eng= -779.380802825 zpe= -779.115236

N	-0.256521	2.263025	0.263554
C	-1.013933	2.083852	-1.036654
C	-0.494311	0.709571	-1.478881
N	-1.282978	-0.366687	-1.212999
C	-0.543289	-1.546094	-0.787250
C	0.259767	-1.130422	0.459286
N	1.507022	-1.593498	0.623974
C	2.238602	-1.115798	1.779908
C	2.704759	0.326082	1.643353
O	3.648042	0.591196	2.537551

H	3.918482	1.517864	2.461801
O	0.651210	0.618190	-1.856857
H	-0.484299	3.146099	0.722742
H	0.761250	2.218130	0.087784
H	-2.149840	-0.193067	-0.724596
H	0.082213	-1.892762	-1.604099
H	-1.244334	-2.335589	-0.514274
O	-0.266859	-0.357307	1.264402
H	1.598994	-1.151082	2.665986
H	3.102032	-1.752659	1.965703
O	2.270632	1.137990	0.864986
H	-0.427582	1.458990	0.904460
C	2.234706	-2.309484	-0.431636
H	3.193428	-2.629422	-0.030122
H	2.407483	-1.656436	-1.289985
H	1.691515	-3.202282	-0.738983
H	-2.074777	2.065944	-0.781998
C	-0.670017	3.195083	-2.005117
H	0.391831	3.161485	-2.254177
H	-0.924171	4.173171	-1.592354
H	-1.239192	3.059046	-2.925235

AGS_16.50_t-c_O1-ctggcgtt.xyz
31
0 eng= -779.377136915 zpe= -779.114625

N	-2.450019	1.730620	-1.496081
C	-2.211659	0.448877	-2.144294
C	-2.882608	-0.649830	-1.345888
N	-3.387310	-0.351194	-0.175943
C	-4.215097	-1.224610	0.652941
C	-3.443202	-2.462123	1.099774
N	-3.358887	-2.797033	2.386490
C	-3.744044	-1.911613	3.472371
C	-2.892360	-0.648414	3.517410
O	-2.960040	-0.077690	4.711979
H	-2.440877	0.740597	4.712523
O	-2.904573	-1.825547	-1.841873
H	-3.151257	2.284075	-1.973708
H	-1.602990	2.282910	-1.439939
H	-3.219798	0.618920	0.099532
H	-5.056043	-1.586668	0.051986
H	-4.608784	-0.621613	1.463212
O	-2.955072	-3.165931	0.200429
H	-4.794992	-1.615179	3.411085
H	-3.634179	-2.448532	4.413561
O	-2.283013	-0.217051	2.577221
H	-3.008730	-2.539858	-1.069547
C	-2.599649	-3.999374	2.758820
H	-2.505676	-4.643160	1.889911
H	-3.136107	-4.522812	3.549098

H   -1.605225   -3.715456   3.110894
H   -2.654279   0.387485   -3.145051
C   -0.717235   0.125083   -2.243969
H   -0.251237   0.190333   -1.257404
H   -0.238136   0.843923   -2.909034
H   -0.562524   -0.876106   -2.643913

AGS_16.76_t-c_O1-ctggcgtt.xyz
31
0 eng= -779.377375912 zpe= -779.114527
N   -3.508179   1.041517   -1.658468
C   -3.637019   1.158509   -0.213048
C   -2.507186   0.400954   0.453578
N   -1.734300   -0.349340   -0.289880
C   -0.753803   -1.315955   0.199249
C   0.367250   -0.620887   0.965303
N   1.644368   -0.792804   0.628452
C   2.072586   -1.448272   -0.595664
C   1.636954   -0.689767   -1.843797
O   2.376736   -1.038234   -2.887170
H   2.063846   -0.574653   -3.678598
O   -2.368760   0.514373   1.717548
H   -3.183921   1.899138   -2.088699
H   -4.384944   0.783850   -2.095027
H   -1.916428   -0.253438   -1.291271
H   -0.425484   -1.902625   -0.651024
H   -1.244846   -1.985030   0.913600
O   0.024940   0.064950   1.942436
H   3.159126   -1.522466   -0.585740
H   1.697827   -2.473317   -0.669148
O   0.718106   0.082748   -1.873864
H   -1.378794   0.268928   1.994506
C   2.688870   -0.095806   1.392238
H   3.487715   -0.800159   1.622836
H   2.260587   0.289071   2.312180
H   3.085468   0.731489   0.799594
H   -3.536785   2.192390   0.133085
C   -4.961994   0.588264   0.304271
H   -5.093140   -0.439035   -0.045050
H   -5.784337   1.196102   -0.073618
H   -4.993036   0.599210   1.393138

AGS_18.15_t-t_N-gtgttgtt.xyz
31
0 eng= -779.379724682 zpe= -779.113996
C   -2.819291   -0.461147   -2.703597
H   -3.342560   0.425310   -3.064449
C   -2.750403   -0.385007   -1.168563
O   -2.251311   0.609017   -0.689541
N   -1.357190   -0.278022   -3.045905

```
H       -0.988459       0.564268        -2.580745
H       -1.192209       -0.227385       -4.052015
N       -3.055777       -1.492054       -0.438184
H       -3.277728       -2.325033       -0.964323
C       -2.132522       -1.700800       0.667247
H       -2.381458       -2.627455       1.186486
H       -2.216475       -0.870805       1.363630
C       -0.719973       -1.801124       0.051640
O       -0.602783       -2.195418       -1.111814
N       0.337791        -1.434544       0.788494
C       1.633021        -1.453214       0.138145
H       2.423436        -1.357499       0.880871
H       1.776041        -2.404016       -0.382944
C       1.799471        -0.360251       -0.907550
O       3.083077        -0.217728       -1.217769
H       3.178780        0.460718        -1.901736
O       0.909095        0.268596        -1.419940
H       -0.816670       -1.061441       -2.626557
C       -3.344529       -1.733906       -3.335645
H       -2.733394       -2.596337       -3.056241
H       -3.352928       -1.652821       -4.424038
H       -4.374511       -1.904424       -3.017395
C       0.213426        -0.803782       2.105835
H       1.179281        -0.850367       2.604481
H       -0.499520       -1.346622       2.724080
H       -0.092759       0.241293        2.013648

AGS_18.40_t-c_O2-ctgtcgtt.xyz
31
0 eng= -779.375050274 zpe= -779.113902
N       -0.569296       -5.249526       -3.892600
C       -1.615547       -4.438455       -3.276222
C       -1.058440       -3.077833       -2.891420
N       0.256586        -2.900089       -3.018841
C       0.909564        -1.596827       -2.981127
C       0.791140        -0.922945       -1.623232
N       1.826618        -0.412680       -0.987101
C       3.149681        -0.303048       -1.585465
C       3.145723        0.666162        -2.762981
O       4.383904        1.003726        -3.094315
H       4.365254        1.601479        -3.857320
H       -2.432872       -4.220669       -3.972040
O       -1.814692       -2.190364       -2.446405
H       -0.711920       -5.370177       -4.888050
H       -0.522087       -6.171945       -3.476622
H       0.736258        -3.711319       -3.409120
H       0.436793        -0.910427       -3.692541
H       1.939907        -1.731843       -3.293923
O       -0.363707       -0.839681       -1.110499
H       3.844214        0.053999        -0.826234
```

H	3.527427	-1.271288	-1.921836
O	2.147564	1.030815	-3.315086
H	-1.134724	-1.375479	-1.733997
C	-2.198638	-5.095890	-2.022773
H	-2.706424	-6.020195	-2.299351
H	-2.915998	-4.434095	-1.539282
H	-1.400887	-5.331690	-1.313226
C	1.616552	0.339356	0.262039
H	1.620670	1.409535	0.043930
H	2.424542	0.096402	0.950205
H	0.663655	0.060091	0.698806

AGS_18.59_t-t_N-gtgttgtt.xyz
31
0 eng= -779.379748924 zpe= -779.113829

C	-2.119408	0.666223	-2.660448
H	-2.373575	1.707520	-2.860348
C	-1.908050	0.514834	-1.145481
O	-1.165003	1.305441	-0.607708
N	-0.703616	0.458997	-3.152651
H	-0.053249	1.094343	-2.663857
H	-0.620042	0.579588	-4.162974
N	-2.377515	-0.602944	-0.529485
H	-2.827461	-1.291817	-1.114410
C	-1.452692	-1.135553	0.460377
H	-1.838865	-2.078016	0.850761
H	-1.353226	-0.427243	1.277635
C	-0.119145	-1.388268	-0.272670
O	-0.149743	-1.726198	-1.459067
N	1.032342	-1.211599	0.390916
C	2.259761	-1.393449	-0.357285
H	3.100943	-1.502073	0.325426
H	2.196308	-2.303993	-0.959246
C	2.545737	-0.255460	-1.325882
O	3.818285	-0.278992	-1.702155
H	3.982431	0.431707	-2.338943
O	1.738719	0.541406	-1.733135
H	-0.382519	-0.490195	-2.872015
C	-3.059325	-0.297156	-3.356248
H	-2.711799	-1.330287	-3.268716
H	-3.148465	-0.056151	-4.416995
H	-4.056690	-0.219276	-2.920153
C	1.094342	-0.590299	1.719075
H	0.457693	-1.122758	2.424407
H	0.802956	0.461533	1.667806
H	2.116958	-0.657905	2.083032

AGS_18.68_NcD.xyz
31
0 eng= -779.379276203 zpe= -779.113796

N	-1.892504	-2.565295	-2.121608
C	-1.558329	-1.099828	-2.080775
C	-2.736183	-0.440881	-2.818835
N	-3.428904	0.536404	-2.179095
C	-3.471811	0.679747	-0.741534
C	-3.799912	-0.654180	-0.050333
N	-3.682935	-0.710941	1.293435
C	-3.745701	-2.032535	1.877174
C	-2.578568	-2.911468	1.418727
O	-2.570793	-4.155241	1.886817
H	-3.301540	-4.334102	2.491260
O	-2.966856	-0.813513	-3.946325
H	-2.791003	-2.693695	-1.626835
H	-1.180845	-3.123021	-1.642971
H	-4.224215	0.868982	-2.710192
H	-4.231958	1.422771	-0.494135
H	-2.522946	1.065788	-0.358436
O	-4.100456	-1.641759	-0.711608
H	-3.730956	-1.946745	2.965674
H	-4.674107	-2.530753	1.582321
O	-1.725516	-2.540770	0.658067
H	-1.999692	-2.856305	-3.099860
C	-3.189203	0.390359	2.118926
H	-2.099462	0.359799	2.210929
H	-3.633967	0.314281	3.110348
H	-3.494661	1.346980	1.701258
H	-1.487238	-0.849426	-1.025074
C	-0.248769	-0.838301	-2.805621
H	-0.022406	0.228016	-2.768203
H	-0.328240	-1.130054	-3.854231
H	0.573570	-1.375791	-2.329722

ASA_0.00_NcA.xyz
34
0 eng= -818.696193069 zpe= -818.40245

N	-1.751550	-0.913710	-2.743266
C	-2.344053	0.002779	-1.709248
C	-3.832573	-0.397512	-1.703497
N	-4.422418	-0.725955	-0.530049
C	-3.628259	-1.151631	0.604047
C	-2.625317	-2.250675	0.215345
N	-1.683884	-2.522199	1.138144
C	-0.578200	-3.434984	0.888219
C	0.271315	-2.931372	-0.277914
O	1.295541	-3.746757	-0.490890
H	1.844350	-3.400529	-1.209234
O	-4.395667	-0.374334	-2.780035
H	-0.762188	-0.714624	-2.902073
H	-2.298113	-0.818763	-3.607760
H	-4.305583	-1.545171	1.364323

H	-3.093623	-0.313239	1.061412
O	-2.662561	-2.785137	-0.882605
H	-1.695213	-2.013390	2.009574
O	0.073669	-1.927190	-0.918426
H	-1.833073	-1.881359	-2.389341
C	-5.815501	-1.179030	-0.588998
H	-6.300656	-0.953376	0.359606
H	-5.857838	-2.253696	-0.787304
H	-6.322405	-0.650720	-1.391885
H	-1.798016	-0.205875	-0.792526
C	-2.177629	1.452928	-2.131978
H	-2.693233	1.638537	-3.075747
H	-1.121598	1.713164	-2.227612
H	-2.617682	2.101605	-1.373370
H	0.074764	-3.385727	1.763974
C	-1.022128	-4.889679	0.694786
H	-0.149096	-5.531641	0.588127
H	-1.652101	-4.986971	-0.187960
H	-1.586646	-5.206754	1.571501

ASA_4.70_NcB.xyz
34
0 eng= -818.694350293 zpe= -818.400659

N	-1.485919	1.832812	-0.449485
C	-1.622190	0.386019	-0.080271
C	-3.102507	0.066471	-0.377246
N	-3.427193	-1.209891	-0.659949
C	-2.413448	-2.189712	-1.035432
C	-1.629744	-1.794280	-2.290648
N	-2.341229	-1.193532	-3.280888
C	-1.650125	-0.674309	-4.438676
C	-0.723755	0.463762	-4.030354
O	0.191191	0.694497	-4.951188
H	0.751312	1.438588	-4.685799
H	-0.910568	-0.159114	-0.698138
O	-3.900577	0.983344	-0.268933
H	-2.395208	2.278125	-0.240377
H	-1.300147	1.892922	-1.465287
H	-2.916669	-3.140813	-1.217358
H	-1.682447	-2.358080	-0.242479
O	-0.436918	-1.989151	-2.372826
H	-3.309306	-0.945698	-3.139459
H	-1.016654	-1.457928	-4.860275
O	-0.825914	1.116034	-3.012848
H	-0.733814	2.293738	0.063590
C	-2.644119	-0.174858	-5.487013
H	-3.278506	-1.000374	-5.810522
H	-2.112006	0.212801	-6.354737
H	-3.273387	0.620184	-5.078662
C	-1.346391	0.190851	1.406645

```
H       -2.034598       0.790573        2.006579
H       -0.316557       0.459147        1.649784
H       -1.489731       -0.856403       1.674635
C       -4.839147       -1.584220       -0.772651
H       -5.105661       -1.794471       -1.812382
H       -5.451132       -0.764543       -0.405976
H       -5.022194       -2.477494       -0.174563

ASA_7.68_NtA.xyz
34
0 eng= -818.693213288 zpe= -818.399525
N       -0.810753       2.736113        0.382781
C       -1.200711       1.977309        -0.853671
C       -1.043088       0.512174        -0.416815
N       -1.838414       -0.411947       -0.972028
C       -1.911332       -1.706204       -0.276917
C       -2.832253       -1.440157       0.911908
N       -2.208027       -1.137649       2.083714
C       -2.977873       -0.472574       3.108091
C       -3.461902       0.882627        2.602286
O       -4.545109       1.285796        3.235379
H       -4.816792       2.156754        2.911174
O       -0.193027       0.276662        0.436145
H       -0.672047       3.731195        0.205223
H       0.050650        2.300260        0.743217
H       -0.910350       -2.024062       0.008389
H       -2.367678       -2.438320       -0.939182
O       -4.035224       -1.368067       0.771347
H       -1.208352       -0.979601       2.057430
O       -2.900049       1.553213        1.755130
H       -1.542675       2.581918        1.099491
C       -2.940272       -0.094982       -1.884177
H       -3.139131       -0.975769       -2.492410
H       -2.661126       0.713097        -2.557731
H       -3.847713       0.156720        -1.329292
H       -2.240420       2.232442        -1.053821
C       -0.273992       2.334016        -2.007032
H       0.759541        2.087276        -1.753512
H       -0.343801       3.393529        -2.260912
H       -0.546858       1.758453        -2.892127
H       -3.867130       -1.064954       3.329127
C       -2.144200       -0.270288       4.372945
H       -2.734424       0.226027        5.142805
H       -1.258545       0.334677        4.161121
H       -1.823145       -1.240693       4.751136

ASA_12.15_c-t_N-gcggtgtt.xyz
34
0 eng= -818.691235533 zpe= -818.397822
N       -0.365496       1.909413        2.084976
```

C	-1.288766	1.215552	1.117742
C	-1.112806	2.020463	-0.184107
N	-0.654808	1.401061	-1.301942
C	0.125222	0.161970	-1.243388
C	1.395322	0.415274	-0.430349
N	2.404507	1.022666	-1.081183
C	3.542033	1.576335	-0.372051
C	3.080109	2.690318	0.566460
O	4.115626	3.275361	1.154053
H	3.799971	3.969417	1.750730
H	-0.940960	0.188640	1.065746
O	-1.413785	3.195887	-0.138947
H	0.612698	1.677363	1.843628
H	-0.556848	1.636741	3.050535
H	0.350005	-0.138712	-2.266535
H	-0.435745	-0.646732	-0.777709
O	1.436103	0.176418	0.770229
H	2.313870	1.224797	-2.066503
H	3.961325	0.799638	0.276586
O	1.931429	2.994480	0.778881
H	-0.483858	2.926126	1.981431
C	4.602852	2.059654	-1.357184
H	4.925256	1.229290	-1.985927
H	5.465933	2.444675	-0.818134
H	4.210687	2.857854	-1.992492
C	-2.724201	1.310784	1.607264
H	-3.032274	2.353598	1.693172
H	-2.841297	0.804899	2.567996
H	-3.382410	0.820345	0.889023
C	-0.493986	2.255280	-2.481671
H	-0.447628	1.625269	-3.368657
H	0.408035	2.870456	-2.400549
H	-1.350017	2.921971	-2.554592

ASA_12.35_c-t_N-gcggtgtt.xyz
34
0 eng= -818.691209986 zpe= -818.397748

C	-3.066540	-1.034555	-0.585795
H	-2.693254	-1.847586	0.027933
C	-2.257910	-0.773015	-1.869558
O	-2.566114	0.210233	-2.513857
N	-2.845436	0.220355	0.217537
H	-3.444088	0.249664	1.044399
H	-3.047455	1.027740	-0.386697
N	-1.268631	-1.629768	-2.223924
C	-0.629902	-2.526784	-1.257596
H	0.110730	-3.119526	-1.794250
H	-1.345715	-3.217551	-0.814316
C	0.009339	-1.699039	-0.140362
O	-0.579135	-1.488045	0.909966

N	1.198245	-1.133049	-0.430293
H	1.639140	-1.317169	-1.319752
C	1.801041	-0.144603	0.441711
H	1.840818	-0.551634	1.458152
C	0.911128	1.092290	0.534205
O	1.488824	2.040447	1.257374
H	0.901101	2.807802	1.316180
O	-0.186853	1.206516	0.042381
H	-1.852990	0.291529	0.503667
C	3.209976	0.200145	-0.034790
H	3.818988	-0.703938	-0.060693
H	3.672721	0.911518	0.646034
H	3.186426	0.643961	-1.033146
C	-4.543366	-1.188611	-0.910021
H	-4.688953	-2.092255	-1.503228
H	-4.900090	-0.338709	-1.493615
H	-5.133587	-1.289439	0.003142
C	-0.532607	-1.293373	-3.445031
H	-0.034777	-2.188689	-3.814312
H	0.198579	-0.500224	-3.258488
H	-1.235093	-0.933711	-4.193250

ASA_12.95_NcC.xyz
34
0 eng= -818.691089681 zpe= -818.397519

N	-3.224767	1.548724	2.061469
C	-2.938809	0.347764	1.201103
C	-3.248635	0.877294	-0.211189
N	-2.342567	0.725578	-1.203631
C	-0.920452	0.612227	-0.930031
C	-0.386544	1.877369	-0.240291
N	0.801613	2.316890	-0.645040
C	1.444510	3.490513	-0.068367
C	2.514968	3.917431	-1.058796
O	3.096074	5.052784	-0.686999
H	3.783919	5.280217	-1.329845
O	-4.342069	1.392965	-0.352377
H	-3.240364	1.328536	3.058213
H	-4.137329	1.926340	1.770174
H	-0.408469	0.430110	-1.874649
H	-0.700658	-0.244406	-0.288834
O	-1.038740	2.416622	0.656367
H	1.272404	1.901693	-1.442487
O	2.803294	3.288268	-2.039396
H	-2.489165	2.245245	1.847313
C	-2.702997	1.289859	-2.509439
H	-2.161768	0.750299	-3.285220
H	-2.453189	2.354305	-2.553116
H	-3.772472	1.174354	-2.661468
H	-1.895281	0.099073	1.370003

C	-3.865429	-0.800611	1.564236
H	-3.662955	-1.647440	0.907218
H	-4.908246	-0.508945	1.429199
H	-3.701853	-1.127258	2.593286
H	0.705425	4.292882	0.009841
C	2.048868	3.209893	1.309362
H	1.260724	2.889754	1.990148
H	2.509536	4.115606	1.702653
H	2.806757	2.427231	1.241378

ASA_13.56_NcA.xyz
34
0 eng= -818.690913098 zpe= -818.397287

N	-3.686609	0.908963	1.538800
C	-3.799640	0.239908	0.194972
C	-2.407124	0.498014	-0.412699
N	-1.679523	-0.530678	-0.904588
C	-1.859928	-1.889639	-0.421210
C	-1.451713	-1.966116	1.055594
N	-0.627249	-2.956227	1.416858
C	0.005233	-2.946246	2.724970
C	0.953940	-1.747853	2.796046
O	1.425802	-1.594167	4.033285
H	2.046004	-0.850597	4.041757
O	-2.051655	1.661737	-0.424868
H	-4.584218	0.987007	2.018908
H	-3.287070	1.845168	1.384457
H	-1.256178	-2.550725	-1.042752
H	-2.893984	-2.222501	-0.521195
O	-1.883638	-1.138515	1.855350
H	-0.193866	-3.513898	0.694231
O	1.247568	-1.052749	1.865087
H	-3.018642	0.344618	2.093934
C	-0.319860	-0.212990	-1.365121
H	0.384908	-0.269869	-0.528853
H	-0.313183	0.793298	-1.773528
H	-0.040047	-0.925713	-2.140158
H	-4.020833	-0.803132	0.400133
C	-4.885034	0.900957	-0.638059
H	-4.656839	1.956741	-0.792677
H	-5.863063	0.798347	-0.163540
H	-4.932765	0.418267	-1.614993
H	-0.765904	-2.799996	3.483973
C	0.754004	-4.252524	2.973196
H	1.217700	-4.230732	3.958060
H	1.540298	-4.396977	2.227753
H	0.062200	-5.094037	2.929266

ASA_14.57_c-c_N-gcgtcgtt.xyz
34

0 eng= -818.690111895 zpe= -818.396901

N	-2.332051	-5.203981	-2.548780
C	-2.725542	-3.776005	-2.268722
C	-2.201448	-3.579243	-0.831920
N	-1.419330	-2.515165	-0.550340
C	-0.707979	-1.815378	-1.599549
C	0.178350	-2.765044	-2.425316
N	1.008747	-2.200444	-3.325986
C	0.847085	-0.882595	-3.912156
C	-0.449911	-0.835034	-4.729434
O	-0.532501	0.273111	-5.454376
H	-1.373881	0.282345	-5.934389
H	-2.242752	-3.167280	-3.031261
O	-2.549386	-4.417833	-0.021363
H	-2.748503	-5.567027	-3.407213
H	-2.634322	-5.766913	-1.740838
H	-0.080799	-1.054826	-1.131076
H	-1.397041	-1.293616	-2.266976
O	0.132191	-3.975333	-2.252451
H	1.533842	-2.874178	-3.872816
H	0.736350	-0.135775	-3.118087
O	-1.305315	-1.682094	-4.689269
H	-1.300409	-5.239389	-2.602023
C	2.073318	-0.525336	-4.748844
H	2.191343	-1.226098	-5.578983
H	2.964338	-0.565211	-4.122656
H	1.970541	0.476854	-5.159728
C	-4.237614	-3.631503	-2.316287
H	-4.709429	-4.278398	-1.574576
H	-4.622413	-3.862224	-3.311612
H	-4.505878	-2.599684	-2.085740
C	-0.862775	-2.438335	0.803714
H	0.076858	-2.995605	0.860874
H	-1.574214	-2.872373	1.500631
H	-0.693335	-1.393117	1.058670

ASA_17.39_t-t_N-ttggtggc.xyz
34
0 eng= -818.689831944 zpe= -818.395827

N	0.258206	2.166436	-1.316496
C	-1.216491	2.070911	-1.046944
C	-1.675186	0.869940	-1.892055
N	-2.777900	0.194611	-1.518034
C	-3.004844	-1.115071	-2.157471
C	-2.035489	-2.035850	-1.420763
N	-0.813109	-2.177808	-1.990553
C	0.363929	-2.452931	-1.156210
C	0.573791	-1.130466	-0.406086
O	-0.257960	-0.871188	0.598930
H	-0.901611	-1.596984	0.718868

H	-1.323910	1.872272	0.019473
O	-0.997332	0.581419	-2.871400
H	0.650748	3.042474	-0.969563
H	0.397068	2.091336	-2.333312
H	-2.814809	-1.040221	-3.226521
H	-4.030233	-1.425972	-1.971508
O	-2.293429	-2.432228	-0.293941
H	-0.636192	-1.655253	-2.841714
O	1.346392	-0.275002	-0.774707
H	0.775087	1.349388	-0.906767
C	-1.900268	3.363410	-1.477278
H	-1.724856	3.546763	-2.539983
H	-1.535223	4.213628	-0.898096
H	-2.977292	3.292843	-1.326358
C	-3.526954	0.460582	-0.287454
H	-4.572028	0.208339	-0.462685
H	-3.480803	1.515720	-0.028620
H	-3.153106	-0.147288	0.539755
H	0.100879	-3.246860	-0.454916
C	1.558688	-2.832533	-2.006379
H	1.330577	-3.736157	-2.571272
H	2.426271	-3.021585	-1.374394
H	1.812054	-2.025967	-2.696293

ASA_18.41_c-t_N-tcggtgtc.xyz
34
0 eng= -818.688541218 zpe= -818.395439

N	-3.922994	-0.581051	0.679318
C	-3.705765	-1.240702	-0.650315
C	-2.350562	-0.679042	-1.127356
N	-1.625841	-1.418857	-1.993039
C	-1.920151	-2.824992	-2.251088
C	-1.757764	-3.700088	-1.006406
N	-0.613921	-3.524203	-0.286591
C	-0.564416	-4.080427	1.046802
C	-1.692720	-3.471080	1.886784
O	-2.110073	-4.152717	2.939037
H	-1.713433	-5.031404	2.988766
H	-3.707339	-2.315230	-0.473682
O	-2.051764	0.441963	-0.752916
H	-3.515915	0.366176	0.609087
H	-3.400233	-1.109083	1.400842
H	-1.238901	-3.170745	-3.030316
H	-2.937228	-2.977103	-2.615911
O	-2.618466	-4.483898	-0.668079
H	-0.047524	-2.706914	-0.468030
H	-0.733805	-5.158668	0.973624
O	-2.161515	-2.385470	1.639738
H	-4.910901	-0.529515	0.929269
C	0.784268	-3.791280	1.704980

```
H       1.582338        -4.238327       1.112260
H       0.828665        -4.211330       2.710402
H       0.952218        -2.714313       1.779461
C       -4.800751       -0.823155       -1.626632
H       -5.778844       -1.171801       -1.289846
H       -4.611415       -1.261494       -2.607098
H       -4.818025       0.263075        -1.741303
C       -0.434541       -0.833566       -2.612995
H       -0.500441       -0.945118       -3.696121
H       0.473515        -1.331679       -2.262204
H       -0.385433       0.220593        -2.354264

SAS_0.00_NcA.xyz
34
0 eng= -818.685327333 zpe= -818.390453
N       -0.613630       -2.661193       -2.520533
C       -1.453990       -1.431821       -2.485258
C       -2.900151       -1.928543       -2.615167
N       -3.829186       -1.457434       -1.753483
C       -3.532784       -0.965956       -0.416414
C       -2.516025       -1.900412       0.273292
N       -1.921330       -1.477071       1.410494
C       -0.954617       -2.374829       2.005929
C       0.331512        -2.450312       1.201549
O       1.268405        -3.098337       1.878340
H       2.079412        -3.150653       1.352211
H       -1.193552       -0.875353       -1.587461
H       -1.226759       -0.838414       -3.372329
O       -3.127794       -2.703447       -3.518401
H       -1.080764       -3.303146       -3.176838
H       -4.736212       -1.890596       -1.887251
H       -3.107537       0.038304        -0.495118
O       -2.255645       -2.996845       -0.210307
H       -0.721093       -2.048053       3.019736
H       -1.355684       -3.389787       2.063768
O       0.486016        -2.006424       0.089335
H       -0.680801       -3.079368       -1.579863
C       0.805563        -2.430891       -2.906483
H       1.236691        -1.721396       -2.205073
H       0.826542        -2.041986       -3.922752
H       1.338439        -3.378196       -2.856676
C       -1.997226       -0.138596       1.991964
H       -1.009766       0.329603        1.968828
H       -2.336941       -0.202930       3.027510
H       -2.688859       0.493951        1.445480
C       -4.838339       -0.911960       0.379791
H       -4.677588       -0.525612       1.385372
H       -5.260803       -1.915550       0.466539
H       -5.556436       -0.266715       -0.127794
```

SAS_4.97_NcA.xyz
34
0 eng= -818.683166141 zpe= -818.38856

N	-1.636747	-1.623843	-0.343312
C	-1.903486	-2.058884	-1.753583
C	-3.374773	-1.765622	-2.069611
N	-3.648889	-0.894310	-3.070970
C	-2.734353	0.062838	-3.668338
C	-1.969845	0.834387	-2.576020
N	-0.867203	1.528934	-2.935095
C	-0.138120	2.186552	-1.872410
C	0.658481	1.202699	-1.032292
O	1.492792	1.837445	-0.225609
H	1.988723	1.199618	0.308726
H	-1.179551	-1.550746	-2.387632
H	-1.747377	-3.136187	-1.811415
O	-4.228277	-2.330153	-1.422448
H	-2.076186	-0.694386	-0.243373
H	-4.640457	-0.708728	-3.168319
H	-2.021430	-0.474375	-4.299624
O	-2.359999	0.816529	-1.412483
H	0.542800	2.927912	-2.291839
H	-0.824791	2.703190	-1.197660
O	0.539869	0.000539	-1.073231
H	-0.631446	-1.434941	-0.266168
C	-2.092489	-2.570462	0.719500
H	-1.869039	-2.127947	1.688149
H	-1.549572	-3.505976	0.597543
H	-3.159941	-2.733114	0.600100
C	-0.191437	1.478564	-4.230593
H	0.744407	0.919085	-4.144292
H	0.030159	2.494180	-4.562203
H	-0.810178	1.007706	-4.987151
C	-3.547475	1.037732	-4.524212
H	-2.906600	1.773564	-5.008386
H	-4.261423	1.572669	-3.894185
H	-4.091621	0.491279	-5.295576

SAS_6.18_c-t_N-tcggtgtt.xyz
34
0 eng= -818.682249471 zpe= -818.388098

N	-0.860391	-1.251463	-1.765758
C	-2.346968	-1.297172	-1.711631
C	-2.793025	0.128952	-1.369085
N	-4.004077	0.318682	-0.812328
C	-4.875472	-0.675077	-0.167790
C	-4.078930	-1.468972	0.887588
N	-3.562776	-0.785720	1.947120
C	-2.666086	-1.538384	2.791393
C	-1.335171	-1.811191	2.115997

```
O       -0.527422       -2.487954       2.912314
H       0.317987        -2.654139       2.471434
H       -2.720172       -1.541267       -2.709171
H       -2.651485       -2.065378       -1.002309
O       -2.042702       1.039970        -1.670348
H       -0.537167       -1.281169       -0.786963
H       -4.238357       1.298370        -0.708664
O       -3.889278       -2.663907       0.748206
H       -2.477043       -0.998540       3.720292
H       -3.099205       -2.507602       3.048201
O       -1.039505       -1.460488       0.995179
H       -0.620830       -0.313733       -2.123473
C       -0.234936       -2.341540       -2.561213
H       -0.538333       -3.297850       -2.139522
H       0.846769        -2.235320       -2.514536
H       -0.574308       -2.261079       -3.592041
C       -3.600465       0.662890        2.119756
H       -2.758368       1.151045        1.619474
H       -3.563144       0.887772        3.185258
H       -4.533895       1.071926        1.739258
H       -5.639863       -0.091620       0.349009
C       -5.569133       -1.603720       -1.153760
H       -6.262795       -2.254274       -0.621146
H       -4.859927       -2.249805       -1.670862
H       -6.121017       -1.012979       -1.885042
```

```
SAS_8.54_t-t_N-gtgttgtt.xyz
34
0 eng= -818.681255727 zpe= -818.387199
N       -2.442983       -0.287686       -3.315552
C       -2.539745       0.114554        -1.874959
C       -1.470378       -0.755993       -1.218421
N       -1.887860       -1.850008       -0.529035
C       -1.055677       -3.036814       -0.730051
C       -1.006526       -3.251363       -2.258728
N       0.148696        -3.622375       -2.833385
C       0.144597        -3.799211       -4.270905
C       0.128271        -2.490536       -5.046162
O       0.506788        -2.694399       -6.302162
H       0.467791        -1.860404       -6.792389
H       -2.285066       1.170281        -1.793629
H       -3.558004       -0.066648       -1.531350
O       -0.313488       -0.554150       -1.513984
H       -2.590175       -1.314784       -3.376553
H       -2.883604       -2.027009       -0.507440
H       -0.069833       -2.820813       -0.325791
O       -2.038337       -3.077943       -2.913092
H       1.010355        -4.383968       -4.578097
H       -0.753189       -4.345394       -4.573295
O       -0.210436       -1.416492       -4.615925
```

H	-1.458554	-0.192645	-3.613653
C	-1.670386	-4.252187	-0.049654
H	-1.037561	-5.128668	-0.196866
H	-2.653476	-4.469508	-0.473724
H	-1.769008	-4.072801	1.021210
C	1.433596	-3.584553	-2.124476
H	2.202987	-3.973566	-2.787588
H	1.407175	-4.219955	-1.239403
H	1.687914	-2.559762	-1.845113
C	-3.351260	0.451205	-4.231057
H	-3.128882	1.514420	-4.163245
H	-4.381294	0.262487	-3.934896
H	-3.182287	0.096438	-5.245498

SAS_10.75_NtA.xyz
34
0 eng= -818.680609563 zpe= -818.38636

N	-1.813485	-0.467358	-3.947879
C	-3.096914	-0.801833	-3.272171
C	-2.696384	-1.289369	-1.875487
N	-3.523905	-2.161401	-1.277461
C	-2.988419	-3.038224	-0.220851
C	-2.189155	-4.070287	-1.035225
N	-0.833129	-4.045921	-1.009878
C	-0.209102	-4.780157	-2.085624
C	-0.613315	-4.237521	-3.447761
O	-0.376196	-5.123338	-4.397188
H	-0.622519	-4.761366	-5.260033
H	-3.705831	0.101936	-3.209191
H	-3.611312	-1.563410	-3.857823
O	-1.656442	-0.867045	-1.402438
H	-1.240405	0.005173	-3.233181
H	-4.235427	-2.599798	-1.851066
O	-2.795287	-4.786475	-1.815715
H	0.876869	-4.698207	-2.009391
H	-0.476169	-5.838295	-2.064328
O	-1.057067	-3.128510	-3.665495
H	-1.332004	-1.367481	-4.116000
C	-1.945475	0.333859	-5.193458
H	-2.556795	-0.219932	-5.903278
H	-0.954275	0.505788	-5.607414
H	-2.419780	1.282952	-4.951439
C	-0.009292	-3.253805	-0.096814
H	0.949791	-3.756044	0.023859
H	-0.471570	-3.212396	0.887328
H	0.144986	-2.239653	-0.470320
H	-2.363115	-2.417020	0.415060
C	-4.115949	-3.686262	0.561465
H	-3.706352	-4.319047	1.349899
H	-4.719135	-4.314613	-0.096211

H	-4.744698	-2.922144	1.018768

SAS_10.76_c-t_N-tcggtgtt.xyz
34
0 eng= -818.680766029 zpe= -818.386353
C	-1.888348	0.143617	1.183853
H	-1.010745	-0.353776	1.595397
H	-2.787580	-0.211120	1.693983
C	-2.088091	-0.024715	-0.329002
O	-2.529141	0.920733	-0.955469
N	-1.756388	1.606589	1.414866
H	-2.356664	2.061250	0.709815
N	-1.815626	-1.229075	-0.863195
H	-2.020563	-1.302380	-1.852930
C	-1.013419	-2.274157	-0.226298
H	-1.469436	-2.529550	0.732837
C	0.386056	-1.770005	0.172931
O	0.738194	-1.910955	1.331329
N	1.183766	-1.140282	-0.736864
C	2.361792	-0.517541	-0.180032
H	2.883077	-1.201194	0.492321
H	3.052645	-0.235081	-0.975980
C	2.018583	0.726533	0.617983
O	3.099151	1.268926	1.147497
H	2.866532	2.064841	1.646764
O	0.901936	1.177658	0.753034
H	-0.783986	1.845702	1.169404
C	-0.988424	-3.539936	-1.075876
H	-0.421020	-4.310584	-0.553919
H	-0.527133	-3.382991	-2.051703
H	-2.006469	-3.903449	-1.224118
C	0.806602	-0.749205	-2.092663
H	0.186502	-1.508837	-2.558867
H	1.713414	-0.667472	-2.691317
H	0.280481	0.210643	-2.107059
C	-2.067031	2.048296	2.801121
H	-1.401674	1.528751	3.488186
H	-3.103376	1.802084	3.023717
H	-1.914620	3.123176	2.870213

SAS_11.00_c-c_N-gcgtcgtt.xyz
34
0 eng= -818.680515091 zpe= -818.386262
C	-2.265331	-3.112434	-2.094050
H	-1.369718	-2.509946	-1.958823
H	-2.917255	-2.653028	-2.838414
C	-3.096035	-3.412933	-0.845950
O	-4.021266	-4.185171	-0.975009
N	-1.850719	-4.451929	-2.605175
H	-2.700284	-5.028739	-2.650121

N	-2.765389	-2.805194	0.316356
H	-3.338800	-3.118127	1.091818
C	-1.459530	-2.260283	0.656793
H	-1.315828	-1.314848	0.124375
C	-0.333204	-3.242390	0.258980
O	-0.604826	-4.370066	-0.136376
N	0.949875	-2.829225	0.410062
C	1.314320	-1.430786	0.340228
H	0.703420	-0.802109	0.990338
H	2.345405	-1.303849	0.671580
C	1.177401	-0.897374	-1.081197
O	1.804005	0.259173	-1.228562
H	1.677988	0.588425	-2.131164
O	0.550188	-1.453544	-1.946878
H	-1.254774	-4.857591	-1.863654
C	-1.441271	-2.015251	2.169293
H	-1.599258	-2.958890	2.696394
H	-2.236233	-1.319355	2.440096
H	-0.490771	-1.605448	2.505358
C	2.001555	-3.775999	0.031055
H	2.926854	-3.484385	0.525601
H	2.150449	-3.780733	-1.053636
H	1.717218	-4.773338	0.354740
C	-1.130670	-4.411214	-3.906836
H	-0.842466	-5.423231	-4.182728
H	-0.250025	-3.782418	-3.785148
H	-1.792902	-3.988395	-4.659778

SAS_13.41_c-t_N-tcgttgtt.xyz
34
0 eng= -818.67968785 zpe= -818.385344

N	-1.881948	-2.024916	0.084227
C	-2.574771	-1.132377	-0.889418
C	-2.345936	0.286138	-0.341701
N	-2.469974	1.323062	-1.185313
C	-2.401048	1.253171	-2.636500
C	-1.117204	0.514565	-3.067895
N	-1.027654	0.118489	-4.365769
C	0.119606	-0.697667	-4.693177
C	-0.030374	-2.107507	-4.145844
O	0.957651	-2.887733	-4.565029
H	0.836072	-3.783454	-4.218606
H	-2.177261	-1.336184	-1.882505
H	-3.645048	-1.348193	-0.858396
O	-2.132220	0.392475	0.851619
H	-2.299232	-2.955781	0.084535
H	-2.264896	2.216454	-0.752676
O	-0.226599	0.302890	-2.263474
H	0.249251	-0.747543	-5.775080
H	1.028209	-0.272591	-4.262958

O	-0.920059	-2.475755	-3.418826
H	-2.036831	-1.577857	1.004248
C	-0.403904	-2.115114	-0.154497
H	-0.250463	-2.575274	-1.127545
H	0.037648	-2.706498	0.644599
H	-0.007207	-1.101686	-0.156590
C	-2.153214	0.028152	-5.294133
H	-2.621104	-0.960313	-5.241999
H	-1.786397	0.193766	-6.307341
H	-2.900851	0.788647	-5.090493
H	-3.276107	0.709726	-3.004705
C	-2.407432	2.683349	-3.180714
H	-2.346922	2.698556	-4.267656
H	-1.542122	3.225154	-2.791918
H	-3.318749	3.197766	-2.873472

SAS_14.00_t-t_N-gtgttggt.xyz
34
0 eng= -818.680060818 zpe= -818.385122

N	-3.392116	-1.368956	-3.257062
C	-3.104094	-0.440636	-2.117835
C	-2.196664	-1.278935	-1.218838
N	-2.702087	-1.717120	-0.040143
C	-2.294514	-3.072809	0.340267
C	-2.535984	-3.927369	-0.926998
N	-1.633935	-4.863783	-1.273386
C	-1.726664	-5.363322	-2.647174
C	-1.150692	-4.282906	-3.553675
O	0.177112	-4.277560	-3.514908
H	0.511409	-3.526611	-4.027890
H	-2.577555	0.430612	-2.505917
H	-4.049156	-0.145349	-1.662715
O	-1.147489	-1.676287	-1.685603
H	-3.748986	-2.261825	-2.863948
H	-3.664780	-1.482981	0.161948
H	-1.235763	-3.044080	0.590012
O	-3.519108	-3.678423	-1.626016
H	-1.149509	-6.281904	-2.735024
H	-2.767051	-5.534671	-2.908778
O	-1.803371	-3.480069	-4.175756
H	-2.495604	-1.660504	-3.675537
C	-4.303394	-0.828347	-4.299748
H	-5.275861	-0.632163	-3.852636
H	-4.394555	-1.572284	-5.088687
H	-3.878421	0.091661	-4.696833
C	-0.388457	-5.082952	-0.533506
H	0.045441	-6.024322	-0.864831
H	-0.594917	-5.168171	0.532388
H	0.328402	-4.279085	-0.718268
C	-3.123918	-3.574376	1.513179

H	-2.805821	-4.577796	1.799759
H	-4.180703	-3.621145	1.239892
H	-2.999931	-2.915530	2.373213

SAS_14.02_NcA.xyz
34
0 eng= -818.679851159 zpe= -818.385114

N	1.206754	-1.541974	-3.087086
C	-0.188190	-1.984467	-2.807526
C	-0.668356	-1.068569	-1.673770
N	-1.245778	-1.617923	-0.580183
C	-1.051056	-2.990767	-0.140837
C	0.462228	-3.311575	-0.089721
N	0.839457	-4.523699	0.362608
C	2.209353	-4.906441	0.091682
C	2.457997	-5.072521	-1.400516
O	3.668782	-5.574643	-1.613825
H	3.812554	-5.688610	-2.564197
H	-0.795570	-1.782610	-3.691508
H	-0.153603	-3.051248	-2.606026
O	-0.514888	0.122898	-1.838944
H	1.742765	-1.814379	-2.247053
H	-1.470918	-0.928540	0.126532
H	-1.434045	-3.021412	0.880606
O	1.290356	-2.505680	-0.505541
H	2.908006	-4.149733	0.456940
H	2.439588	-5.845895	0.594087
O	1.684020	-4.776765	-2.276002
H	1.189154	-0.513723	-3.111126
C	1.800433	-2.120583	-4.322784
H	1.205715	-1.802335	-5.176838
H	1.785710	-3.204552	-4.224653
H	2.821982	-1.759430	-4.422047
C	-0.049761	-5.543189	0.912241
H	-0.326881	-6.281737	0.155005
H	-0.950940	-5.088947	1.316775
H	0.461462	-6.045437	1.733350
C	-1.829639	-4.019676	-1.001472
H	-2.462408	-4.655111	-0.383411
H	-1.162344	-4.669994	-1.571751
H	-2.482277	-3.488722	-1.694080

SSA_0.00_NcA.xyz
34
0 eng= -818.689728897 zpe= -818.395215

C	-1.707288	1.227297	0.112727
H	-2.305882	2.122116	-0.064953
H	-0.716674	1.349291	-0.318586
C	-2.544762	0.015134	-0.325597
O	-3.549402	-0.195905	0.324893

N	-1.538184	1.071048	1.585095
H	-2.425842	0.676299	1.928246
N	-2.145787	-0.713288	-1.391962
C	-0.756842	-0.705251	-1.800847
H	-0.481692	0.237659	-2.283572
H	-0.615613	-1.491343	-2.544715
C	0.182517	-0.963979	-0.612796
O	-0.247130	-1.234320	0.497281
N	1.495876	-0.818614	-0.868813
H	1.802386	-0.539569	-1.790056
C	2.483637	-0.902660	0.188727
H	2.348399	-1.846560	0.726548
C	2.251066	0.199120	1.220127
O	3.141215	0.113514	2.200588
H	2.987895	0.820051	2.843972
O	1.379612	1.033664	1.179262
H	-0.811008	0.351724	1.715284
C	-1.184948	2.324486	2.305961
H	-1.060057	2.094452	3.362137
H	-1.994858	3.038929	2.171755
H	-0.254095	2.702921	1.891631
C	-2.940012	-1.903021	-1.713441
H	-2.821125	-2.131725	-2.771409
H	-3.985176	-1.694363	-1.501643
H	-2.616554	-2.752460	-1.105232
C	3.894117	-0.834228	-0.393168
H	4.039504	-1.652934	-1.098473
H	4.631692	-0.925615	0.401512
H	4.056758	0.115190	-0.909261

SSA_3.64_NcA.xyz
34
0 eng= -818.688108347 zpe= -818.393827

N	-2.340961	-2.084879	-0.053810
C	-1.883033	-1.828810	-1.448345
C	-2.649457	-0.576067	-1.902480
N	-1.974183	0.436337	-2.491661
C	-0.557095	0.605615	-2.247564
C	-0.236727	0.561696	-0.743881
N	1.074176	0.546246	-0.435321
C	1.549461	0.380321	0.929199
C	1.053875	-0.945279	1.503383
O	1.499824	-1.109539	2.742191
H	1.208449	-1.967680	3.081935
H	-0.796661	-1.790198	-1.433441
H	-2.216813	-2.659901	-2.071710
O	-3.850468	-0.597364	-1.717955
H	-3.339109	-1.832192	-0.034780
H	-0.255833	1.574925	-2.648928
H	0.037281	-0.150310	-2.770024

O	-1.122523	0.488064	0.092230
H	1.753486	0.575431	-1.180800
O	0.369914	-1.757784	0.928859
H	-1.837762	-1.409670	0.541733
C	-2.121690	-3.477409	0.423277
H	-2.454898	-3.547723	1.456624
H	-1.058436	-3.694271	0.358920
H	-2.700229	-4.152390	-0.204559
C	-2.747843	1.631542	-2.841498
H	-2.258986	2.138484	-3.672193
H	-2.822616	2.302708	-1.981248
H	-3.747155	1.326346	-3.139458
H	2.637630	0.284715	0.876538
C	1.206825	1.566217	1.838705
H	0.127756	1.674974	1.939117
H	1.617823	2.476602	1.402933
H	1.647782	1.417257	2.823176

SSA_6.33_NcB.xyz
34
0 eng= -818.686348787 zpe= -818.392803

N	-2.691056	0.762323	-0.415887
C	-3.160164	-0.419715	-1.184964
C	-2.576410	-0.262482	-2.599779
N	-2.410192	-1.376473	-3.337982
C	-2.503056	-2.705419	-2.743812
C	-1.476343	-2.939866	-1.631785
N	-0.256145	-2.365892	-1.805544
C	0.723995	-2.442461	-0.746800
C	0.246108	-1.661949	0.471383
O	0.897294	-2.025117	1.559564
H	0.600945	-1.498413	2.316070
H	-2.864737	-1.312500	-0.636479
H	-4.249978	-0.378606	-1.257748
O	-2.337179	0.872180	-2.977251
H	-1.726975	0.539925	-0.121622
H	-2.340351	-3.440051	-3.534178
H	-3.488601	-2.903602	-2.318382
O	-1.759626	-3.577985	-0.641845
H	-0.080099	-1.760328	-2.593776
H	0.828486	-3.482459	-0.428972
O	-0.593161	-0.786395	0.458582
H	-2.639771	1.540798	-1.090044
C	2.072741	-1.892968	-1.211249
H	2.434789	-2.480224	-2.055366
H	2.802565	-1.959619	-0.405457
H	1.983285	-0.847315	-1.516679
C	-3.513547	1.089297	0.780053
H	-4.524629	1.329518	0.456941
H	-3.068393	1.941777	1.288592

H	-3.521189	0.222852	1.438685
C	-1.998729	-1.261072	-4.739119
H	-2.704624	-1.803384	-5.369468
H	-1.000259	-1.682883	-4.882374
H	-1.989947	-0.210598	-5.017123

SSA_11.10_c-t_N-tcgttgtt.xyz
34
0 eng= -818.684915817 zpe= -818.390989

C	-0.767468	0.641736	-1.839278
H	-0.810912	0.947869	-2.886707
H	0.248705	0.358886	-1.570288
C	-1.861574	-0.414197	-1.583363
O	-2.940555	0.003412	-1.195938
N	-1.161041	1.801037	-0.987278
H	-2.196966	1.786388	-0.995356
N	-1.586662	-1.699825	-1.862102
C	-0.217701	-2.147283	-1.995718
H	0.292111	-1.628093	-2.813446
H	-0.224313	-3.207618	-2.253142
C	0.570425	-1.926182	-0.694729
O	0.055973	-1.414559	0.279726
N	1.872564	-2.283695	-0.744675
H	2.267353	-2.596118	-1.620710
C	2.786097	-1.856506	0.294502
H	2.378621	-2.153880	1.264629
C	2.870329	-0.330873	0.313816
O	3.646715	0.084503	1.310450
H	3.711831	1.049584	1.287371
O	2.318764	0.412913	-0.458363
H	-0.829645	2.677815	-1.389552
C	-0.692516	1.665753	0.430364
H	-1.068704	2.513547	0.998801
H	0.395030	1.636708	0.422861
H	-1.083241	0.727531	0.819739
C	-2.596404	-2.705856	-1.521705
H	-2.424391	-3.081460	-0.509508
H	-2.537437	-3.523028	-2.239559
H	-3.579708	-2.246588	-1.571714
C	4.163950	-2.483112	0.092840
H	4.834142	-2.179327	0.894869
H	4.599641	-2.169244	-0.859324
H	4.076451	-3.569755	0.102206

SSA_11.33_NtA.xyz
34
0 eng= -818.685140045 zpe= -818.390901

C	-0.877932	1.918529	-0.799587
H	-1.853291	2.089384	-0.346546
H	-0.783956	2.495933	-1.721758

C	-0.583728	0.430544	-1.023840
O	0.549062	0.038715	-0.768110
N	0.158598	2.374566	0.166300
H	1.041087	1.934846	-0.130373
N	-1.587533	-0.356164	-1.433993
C	-1.416507	-1.798694	-1.206443
H	-0.416739	-2.101382	-1.511655
H	-2.167657	-2.338349	-1.778568
C	-1.682278	-1.971181	0.287413
O	-2.811577	-1.953121	0.730958
N	-0.578192	-1.982931	1.084537
H	0.311572	-1.751486	0.661313
C	-0.754236	-1.715482	2.492032
H	-1.495854	-2.405913	2.897050
C	-1.302064	-0.306466	2.696016
O	-1.944960	-0.192803	3.842233
H	-2.259622	0.715145	3.958713
O	-1.132959	0.628113	1.934822
H	-0.078401	1.911956	1.059900
C	0.271114	3.849158	0.318986
H	0.551437	4.281134	-0.639651
H	1.032366	4.068277	1.064626
H	-0.691206	4.242224	0.640866
C	-2.955748	0.127394	-1.626757
H	-3.455638	-0.540551	-2.326275
H	-2.949103	1.121481	-2.072000
H	-3.510354	0.123247	-0.684457
C	0.569250	-1.853864	3.243968
H	0.426841	-1.652902	4.305505
H	1.313526	-1.157257	2.849065
H	0.944649	-2.870437	3.128473

SSA_12.58_NcA.xyz
34
0 eng= -818.685030541 zpe= -818.390424

N	-2.045091	-1.439473	0.729961
C	-1.515752	-1.853764	-0.612896
C	-1.672523	-0.657404	-1.561655
N	-0.568830	-0.166563	-2.179679
C	0.764189	-0.448828	-1.694533
C	0.909863	-0.151089	-0.194766
N	2.062749	-0.573211	0.359558
C	2.304398	-0.531845	1.792640
C	1.274848	-1.391481	2.524099
O	1.490224	-1.370348	3.831395
H	0.861824	-1.957212	4.276287
H	-0.503540	-2.217877	-0.455539
H	-2.139987	-2.666798	-0.984277
O	-2.791859	-0.214320	-1.718584
H	-1.551129	-1.992128	1.439555

H	1.466538	0.179691	-2.245382
H	1.054415	-1.486435	-1.888218
O	0.015540	0.382865	0.442489
H	2.734710	-1.049612	-0.223420
H	3.265313	-1.025146	1.964243
O	0.392276	-2.032936	2.004266
H	-1.711208	-0.476663	0.886170
C	2.382559	0.889700	2.361454
H	1.430089	1.404775	2.248319
H	3.152642	1.443159	1.824558
H	2.649594	0.850007	3.416438
C	-3.526946	-1.541102	0.893801
H	-3.810563	-2.586157	0.782557
H	-3.995426	-0.933920	0.123874
H	-3.786811	-1.184230	1.888404
C	-0.749253	1.028733	-3.008632
H	0.009080	1.035844	-3.790264
H	-0.667170	1.932437	-2.398241
H	-1.736793	0.996232	-3.460597

SSA_14.26_NcB.xyz
34
0 eng= -818.684173307 zpe= -818.389784

C	-1.003227	-1.250777	-2.713991
H	-1.869212	-1.788975	-3.107834
H	-0.244240	-1.964537	-2.399514
C	-1.507081	-0.259695	-1.650443
O	-1.864502	0.837056	-2.045056
N	-0.422516	-0.424243	-3.804193
H	0.530799	-0.176871	-3.491244
N	-1.565133	-0.679116	-0.371240
C	-0.866199	-1.883205	0.064982
H	-1.252384	-2.785847	-0.412219
H	-1.024805	-1.994343	1.139083
C	0.639195	-1.836178	-0.212380
O	1.241820	-2.827229	-0.566245
N	1.236374	-0.621495	-0.099845
H	0.701897	0.187494	0.175663
C	2.649947	-0.478108	-0.409008
H	2.857301	0.596780	-0.444251
C	2.878938	-0.957851	-1.842940
O	4.080790	-1.461134	-2.029073
H	4.185589	-1.716442	-2.957578
O	2.068942	-0.833019	-2.738916
H	-0.984076	0.438940	-3.841379
C	3.566073	-1.134537	0.621183
H	3.350117	-0.715113	1.603825
H	4.609665	-0.938837	0.378955
H	3.406030	-2.211471	0.644737
C	-0.339209	-1.110771	-5.121819

H	0.269513	-2.005151	-5.002957
H	0.124234	-0.436140	-5.838373
H	-1.344112	-1.373246	-5.446948
C	-2.178865	0.183105	0.641249
H	-2.686113	1.004557	0.142390
H	-1.421513	0.586717	1.319508
H	-2.897790	-0.397446	1.220221

SSA_15.25_NcC.xyz
34
0 eng= -818.683256051 zpe= -818.389405

C	-2.695704	-2.118735	-2.394025
H	-3.585845	-2.632693	-2.760116
H	-2.012997	-2.822954	-1.929618
C	-3.146107	-0.920394	-1.549970
O	-3.751228	-0.054764	-2.154702
N	-1.998285	-1.503776	-3.559853
H	-1.139355	-1.088739	-3.160817
N	-2.874326	-0.881991	-0.227152
C	-1.769200	-1.626308	0.348775
H	-1.874903	-2.699981	0.174684
H	-1.794337	-1.485606	1.429016
C	-0.422294	-1.178616	-0.239329
O	-0.324778	-0.916942	-1.439691
N	0.606113	-1.136565	0.603669
H	0.496569	-1.383922	1.581760
C	1.961587	-0.819103	0.174041
H	2.185415	-1.382255	-0.735969
C	2.884406	-1.299391	1.280865
O	4.161966	-1.183661	0.933094
H	4.716375	-1.469901	1.673872
O	2.497361	-1.707498	2.341077
H	-2.596026	-0.724087	-3.866981
C	-1.692704	-2.435443	-4.678170
H	-1.055153	-3.233101	-4.302055
H	-1.178583	-1.885008	-5.463135
H	-2.626866	-2.846006	-5.056360
C	-3.291632	0.326953	0.491666
H	-4.229164	0.680722	0.071809
H	-2.540055	1.115764	0.390644
H	-3.431007	0.079476	1.542834
C	2.154444	0.677784	-0.085134
H	1.461709	1.002860	-0.860681
H	3.175076	0.867315	-0.416606
H	1.966398	1.248073	0.826492

SSA_16.13_NcC.xyz
34
0 eng= -818.683034659 zpe= -818.389071

N	-2.733699	0.729126	1.414979

C	-3.049287	-0.651021	0.947120
C	-2.747010	-0.618764	-0.555838
N	-1.964840	-1.571534	-1.109640
C	-0.985471	-2.305897	-0.328049
C	0.081878	-1.363371	0.247856
N	1.341493	-1.788248	0.195220
C	2.453705	-1.010108	0.724215
C	3.710858	-1.574478	0.084791
O	4.781191	-0.851907	0.397350
H	5.563167	-1.255464	-0.007330
H	-2.474891	-1.345468	1.551614
H	-4.119051	-0.821129	1.077106
O	-3.262451	0.294761	-1.172771
H	-3.156604	1.366672	0.726707
H	-0.540970	-3.065269	-0.970888
H	-1.453998	-2.837707	0.503157
O	-0.248973	-0.287321	0.749973
H	1.590482	-2.638843	-0.298843
O	3.725647	-2.562756	-0.596080
H	-1.707615	0.808436	1.313936
C	-3.174090	1.041057	2.800816
H	-2.680351	0.353088	3.484328
H	-4.254050	0.921381	2.860828
H	-2.895688	2.065975	3.036348
C	-1.681768	-1.436356	-2.542784
H	-1.464013	-2.421937	-2.951295
H	-0.831283	-0.768061	-2.708774
H	-2.555446	-1.020503	-3.036935
H	2.336621	0.029539	0.406048
C	2.539817	-1.076113	2.251001
H	1.614496	-0.693924	2.681241
H	3.371101	-0.465129	2.601690
H	2.692152	-2.105555	2.580621

SSA_16.73_NcA.xyz
34
0 eng= -818.682871009 zpe= -818.388842

N	-2.319306	-0.156407	0.169576
C	-2.878193	-1.075463	-0.864100
C	-1.654216	-1.497819	-1.688195
N	-1.404268	-2.806997	-1.913100
C	-1.920330	-3.842701	-1.034327
C	-1.286590	-3.703590	0.354812
N	-0.785272	-4.811163	0.915206
C	0.048750	-4.720933	2.101085
C	1.336456	-3.981572	1.733272
O	2.053191	-3.712694	2.824815
H	2.875186	-3.275263	2.559422
H	-3.426327	-1.857021	-0.348216
H	-3.543756	-0.500346	-1.509306

O	-0.965651	-0.581222	-2.096165
H	-1.745338	-0.765204	0.777411
H	-1.695051	-4.808921	-1.485702
H	-3.005668	-3.786649	-0.937374
O	-1.260401	-2.608336	0.911060
H	-0.701271	-5.644510	0.349682
H	-0.472951	-4.121212	2.849525
O	1.666689	-3.699079	0.616404
H	-1.673499	0.470904	-0.328948
C	0.354772	-6.110716	2.652752
H	-0.572975	-6.622057	2.910466
H	0.968447	-6.026444	3.548192
H	0.900000	-6.708806	1.918113
C	-3.331187	0.602671	0.951510
H	-3.921282	1.207527	0.265809
H	-2.816618	1.240232	1.667233
H	-3.970053	-0.106253	1.474477
C	-0.140360	-3.134249	-2.588043
H	-0.275477	-4.055721	-3.153397
H	0.659382	-3.258696	-1.850275
H	0.116606	-2.324913	-3.265164

AAS_0.00_NcA.xyz
34
0 eng= -818.694136589 zpe= -818.400136

N	-3.585170	-2.025642	-1.599282
C	-2.161546	-1.677320	-1.933064
C	-1.871499	-0.481781	-1.009172
N	-0.816020	-0.553764	-0.165209
C	-0.188776	-1.782142	0.297379
C	-1.262617	-2.817218	0.690092
N	-0.892340	-4.111541	0.807976
C	-1.945688	-5.064344	1.090014
C	-2.833021	-5.314410	-0.118671
O	-3.625455	-6.354720	0.086740
H	-4.193193	-6.495973	-0.685067
O	-2.607847	0.475739	-1.102767
H	-3.922937	-2.802967	-2.170285
H	-4.165085	-1.187796	-1.724843
H	-0.750349	0.251283	0.447654
H	0.429287	-2.189690	-0.507028
O	-2.423299	-2.465124	0.881767
H	-1.513686	-6.010908	1.415578
H	-2.590678	-4.694385	1.890575
O	-2.839345	-4.645845	-1.124711
H	-3.608924	-2.315239	-0.605685
C	0.689058	-1.450807	1.506864
H	0.071489	-1.039090	2.308308
H	1.446367	-0.717556	1.226759
H	1.191271	-2.337932	1.890766

```
C       0.391034        -4.676459       0.392150
H       1.165782        -3.917592       0.355034
H       0.301320        -5.145230       -0.592390
H       0.699419        -5.429856       1.117527
H       -1.582619       -2.574197       -1.722062
C       -2.044820       -1.274357       -3.393462
H       -2.332326       -2.097856       -4.049882
H       -1.008764       -1.013199       -3.613189
H       -2.663924       -0.399591       -3.599621

AAS_10.52_OtB.xyz
34
0 eng= -818.686847375 zpe= -818.39613
C       -2.834941       2.307558        -0.297893
H       -2.846175       2.739772        -1.304451
C       -1.558371       1.486578        -0.237894
O       -0.471619       2.061871        -0.555980
N       -3.982891       1.443308        -0.054541
H       -4.525802       1.274113        -0.892704
H       -4.604633       1.835635        0.642297
N       -1.650752       0.240268        0.168447
H       -2.625810       -0.005877       0.365725
C       -0.654991       -0.842699       0.085134
H       -1.107713       -1.681661       0.607596
C       0.678530        -0.473608       0.721177
O       1.177106        0.644710        0.491679
N       1.344335        -1.388494       1.424051
C       2.757830        -1.117304       1.645061
H       2.910359        -0.077350       1.930697
H       3.138101        -1.752719       2.444329
C       3.520383        -1.396555       0.354539
O       4.819725        -1.178100       0.514008
H       5.279041        -1.359737       -0.319382
O       2.997691        -1.767223       -0.658824
H       0.361825        1.489231        -0.188898
C       -0.372521       -1.230442       -1.374293
H       0.388971        -2.011562       -1.413663
H       0.009674        -0.370701       -1.927834
H       -1.287419       -1.588468       -1.846155
C       -2.732867       3.441967        0.725903
H       -1.822693       4.020663        0.574569
H       -2.729047       3.036265        1.740916
H       -3.590471       4.105797        0.615081
C       0.892662        -2.777121       1.572404
H       1.587369        -3.286218       2.236215
H       0.889239        -3.295567       0.610839
H       -0.091717       -2.817716       2.035943

AAS_11.28_t-t_N-gtgttgtt.xyz
34
```

```
0 eng= -818.689463873 zpe= -818.395841
N       -3.530347       -1.203478       -2.782768
C       -3.282726       0.092490        -2.042656
C       -2.129790       -0.335104       -1.119227
N       -2.372812       -0.463658       0.210082
C       -1.735841       -1.634563       0.812878
C       -2.180909       -2.839424       -0.047593
N       -1.309220       -3.833940       -0.275088
C       -1.758046       -4.933719       -1.104298
C       -1.826157       -4.585727       -2.583642
O       -1.854449       -5.691097       -3.318027
H       -1.927872       -5.456465       -4.254620
H       -2.885196       0.800052        -2.770822
O       -1.112221       -0.725177       -1.649928
H       -2.651670       -1.559583       -3.191899
H       -4.244088       -1.110383       -3.506660
H       -3.319246       -0.291756       0.521323
H       -0.659530       -1.490363       0.760253
O       -3.330926       -2.857276       -0.496579
H       -1.105223       -5.794705       -0.968855
H       -2.768684       -5.229864       -0.810423
O       -1.884746       -3.473172       -3.043330
H       -3.800715       -1.939213       -2.098536
C       -2.192515       -1.819634       2.253704
H       -1.707460       -2.691192       2.695901
H       -3.273059       -1.976208       2.295529
H       -1.931436       -0.941511       2.844840
C       -4.568034       0.583928        -1.408848
H       -5.029576       -0.188624       -0.787646
H       -5.288533       0.883797        -2.171764
H       -4.363648       1.460990        -0.792439
C       0.121774        -3.713425       0.027852
H       0.598701        -4.669205       -0.177689
H       0.276400        -3.486865       1.082410
H       0.583403        -2.942543       -0.593498

AAS_12.45_OtB.xyz
34
0 eng= -818.685831068 zpe= -818.395394
N       -1.242595       2.340059        -2.989315
C       -1.935088       1.243592        -3.653947
C       -2.261981       0.158562        -2.642194
N       -1.768310       0.283683        -1.431706
C       -2.093178       -0.454778       -0.198425
C       -1.949891       -1.964255       -0.376840
N       -1.447003       -2.697317       0.613662
C       -1.184854       -4.095633       0.307355
C       0.070085        -4.197083       -0.552157
O       0.347925        -5.466771       -0.827675
H       1.145836        -5.507635       -1.374953
```

O	-2.969265	-0.821112	-3.035470
H	-0.383461	2.584206	-3.467367
H	-1.809924	3.176366	-2.924948
H	-1.228619	1.151303	-1.349601
O	-2.375876	-2.503635	-1.415297
H	-1.046815	-4.658493	1.230044
H	-2.021082	-4.531192	-0.239197
O	0.707167	-3.251485	-0.919097
H	-2.841930	-1.641496	-2.354470
C	-0.797994	-2.131386	1.800092
H	-0.712774	-2.918859	2.545741
H	-1.404657	-1.338418	2.233753
H	0.201441	-1.762941	1.556452
H	-1.366227	-0.098759	0.527656
C	-3.514877	-0.136834	0.280587
H	-4.251243	-0.463541	-0.456011
H	-3.621243	0.935961	0.441382
H	-3.717658	-0.653795	1.220457
H	-2.904895	1.553579	-4.058331
C	-1.104062	0.621552	-4.780371
H	-1.608377	-0.248149	-5.199279
H	-0.125683	0.313706	-4.402396
H	-0.961337	1.357860	-5.571541

AAS_12.46_OtB.xyz
34
0 eng= -818.685831647 zpe= -818.395389

C	-1.233175	0.075451	-4.433974
H	-2.301097	-0.103226	-4.600570
C	-0.880037	-0.768310	-3.221035
O	-1.175514	-2.003667	-3.255520
N	-0.948796	1.477111	-4.154762
H	-1.789650	2.033888	-4.063162
H	-0.377963	1.894568	-4.880119
N	-0.263196	-0.176334	-2.224165
H	-0.129550	0.820364	-2.423113
C	-0.010506	-0.653207	-0.852871
H	0.626635	0.111646	-0.415294
C	0.710003	-1.999071	-0.834763
O	0.359022	-2.895153	-1.625074
N	1.648805	-2.228455	0.080372
C	2.404934	-3.462487	-0.072233
H	2.937112	-3.688541	0.851311
H	1.738400	-4.294759	-0.297468
C	3.400164	-3.306184	-1.216373
O	4.121388	-4.412146	-1.364414
H	4.744512	-4.291585	-2.095868
O	3.505367	-2.314648	-1.880051
H	-0.591944	-2.524570	-2.519996
C	-1.316018	-0.769908	-0.056563

```
H       -1.102695       -1.075532       0.969502
H       -1.975524       -1.514673       -0.505938
H       -1.825636       0.193378        -0.035881
C       -0.448051       -0.454055       -5.637880
H       0.621601        -0.276869       -5.498978
H       -0.777735       0.063565        -6.538907
H       -0.613143       -1.522629       -5.768656
C       2.215349        -1.190676       0.947021
H       2.907523        -0.555302       0.389177
H       2.756881        -1.682394       1.752221
H       1.428176        -0.593148       1.403303

AAS_12.64_NtA.xyz
34
0 eng= -818.689368232 zpe= -818.395321
N       -3.835423       -0.295849       -3.396068
C       -3.375157       0.170309        -2.041180
C       -1.909930       0.574542        -2.306661
N       -1.049984       0.489611        -1.277587
C       0.374071        0.271930        -1.583990
C       0.399757        -1.204370       -2.021726
N       0.753417        -1.524621       -3.290989
C       0.440770        -2.878611       -3.678617
C       -1.056145       -3.137619       -3.723771
O       -1.302261       -4.419464       -3.929826
H       -2.256027       -4.577933       -3.961562
H       -3.950949       1.068355        -1.804936
O       -1.610925       0.922104        -3.436034
H       -3.451285       0.360638        -4.092030
H       -4.850573       -0.358757       -3.469007
H       -1.360116       -0.013666       -0.455486
H       0.635109        0.959064        -2.384460
O       -0.014154       -2.045093       -1.238475
H       0.847532        -3.092648       -4.667985
H       0.866253        -3.599598       -2.977343
O       -1.925035       -2.299434       -3.606800
H       -3.394733       -1.212283       -3.596256
C       1.229917        0.499389        -0.350175
H       2.282086        0.342177        -0.591954
H       0.954519        -0.206422       0.435764
H       1.104445        1.519661        0.012523
C       -3.581608       -0.923948       -1.007382
H       -2.899775       -1.759755       -1.185967
H       -4.612712       -1.282677       -1.020530
H       -3.402979       -0.529115       -0.006166
C       1.181035        -0.554988       -4.302169
H       1.621618        -1.100627       -5.134562
H       1.955787        0.096253        -3.899483
H       0.342410        0.045361        -4.660539
```

AAS_12.82_c-t_N-tcggtgtt.xyz
34
0 eng= -818.688857878 zpe= -818.395255

N	-1.873561	0.660272	2.678143
C	-2.172589	0.066730	1.333249
C	-3.340061	0.922306	0.807508
N	-3.527557	0.971099	-0.524542
C	-2.513986	0.656788	-1.532578
C	-1.226096	1.478226	-1.339738
N	-1.277040	2.838121	-1.246710
C	-0.035075	3.462156	-0.853236
C	0.287330	3.202061	0.606414
O	1.444639	3.735450	0.946567
H	1.631968	3.566518	1.881160
O	-4.076786	1.446838	1.622764
H	-1.429126	-0.008483	3.307559
H	-2.775368	0.982834	3.067140
H	-4.356683	1.480133	-0.808272
O	-0.170886	0.876742	-1.239302
H	0.798087	3.078574	-1.444645
H	-0.088840	4.541511	-1.004793
O	-0.425980	2.591412	1.373067
H	-1.263039	1.483368	2.545789
C	-2.484013	3.655408	-1.153119
H	-3.273413	3.258867	-1.783774
H	-2.252222	4.653931	-1.522987
H	-2.844043	3.730180	-0.121930
H	-2.176907	-0.371502	-1.382995
C	-3.091924	0.752636	-2.940416
H	-2.323680	0.473972	-3.661845
H	-3.436582	1.757774	-3.185872
H	-3.928834	0.060266	-3.044342
H	-1.264599	0.151703	0.736421
C	-2.623786	-1.382222	1.481523
H	-2.883042	-1.787686	0.503057
H	-3.507908	-1.448107	2.119883
H	-1.824312	-1.999015	1.896146

AAS_15.13_c-c_N-gcgtcgtt.xyz
34
0 eng= -818.687980005 zpe= -818.394372

N	-0.313285	-5.102188	-0.422581
C	-0.642923	-3.876071	-1.234839
C	-2.174663	-3.948288	-1.328743
N	-2.907157	-2.872276	-0.964210
C	-2.501918	-1.820200	-0.042744
C	-1.791987	-2.414122	1.195168
N	-1.238405	-1.549389	2.079328
C	-0.798470	-0.226578	1.689820
C	0.432254	-0.293722	0.792885

O	1.038080	0.881204	0.712646
H	1.795960	0.818161	0.111970
H	-0.260618	-3.019663	-0.682846
O	-2.649618	-4.979714	-1.756482
H	0.688229	-5.299392	-0.400950
H	-0.818635	-5.895882	-0.840287
H	-3.903507	-3.048686	-1.031028
O	-1.774807	-3.626376	1.378744
H	-1.566348	0.333475	1.153174
H	-0.561594	0.355599	2.580617
O	0.781490	-1.287200	0.208992
H	-0.674613	-4.950207	0.534906
C	-0.011503	-3.976391	-2.613320
H	1.077717	-3.998904	-2.542289
H	-0.289581	-3.099168	-3.198792
H	-0.371092	-4.862609	-3.139359
C	-0.558622	-2.124465	3.242797
H	-1.118477	-2.987296	3.592250
H	-0.520257	-1.373683	4.030613
H	0.458246	-2.436314	2.983593
H	-1.817242	-1.137622	-0.554622
C	-3.770803	-1.072846	0.382903
H	-3.557090	-0.264446	1.079542
H	-4.453963	-1.765453	0.879715
H	-4.264831	-0.653317	-0.494350

AAS_15.15_t-t_O1-ctggtgtt.xyz
34
0 eng= -818.685241548 zpe= -818.394365

N	-0.170924	0.775399	-4.626449
C	-1.081276	-0.299960	-4.254479
C	-0.567286	-0.983482	-3.002003
N	0.637199	-0.675237	-2.588887
C	1.329013	-1.112125	-1.360710
C	1.355758	-2.642565	-1.349746
N	2.499292	-3.315608	-1.476570
C	2.352667	-4.720860	-1.824248
C	1.804504	-4.834104	-3.244521
O	1.697154	-6.107160	-3.603411
H	1.344306	-6.159285	-4.503756
O	-1.330932	-1.842224	-2.448673
H	-0.544847	1.691926	-4.410898
H	0.045478	0.753932	-5.615706
H	1.064371	0.034168	-3.192604
O	0.269493	-3.237127	-1.247191
H	3.316692	-5.224095	-1.762404
H	1.660256	-5.218723	-1.145049
O	1.512501	-3.894662	-3.932599
H	-0.789359	-2.468058	-1.802896
C	3.801096	-2.687015	-1.718449

H	3.876970	-2.325590	-2.747434
H	4.573496	-3.433925	-1.548837
H	3.977876	-1.876780	-1.014430
H	2.330472	-0.699278	-1.443212
C	0.646988	-0.572630	-0.104380
H	1.199454	-0.892926	0.780527
H	-0.374642	-0.943978	-0.018222
H	0.632026	0.516883	-0.132000
H	-2.083460	0.062324	-4.002298
C	-1.193240	-1.363189	-5.352862
H	-0.203282	-1.753514	-5.602302
H	-1.637072	-0.913899	-6.241741
H	-1.823470	-2.189675	-5.027759

AAS_17.32_t-c_O1-ctggcgtt.xyz
34
0 eng= -818.684253507 zpe= -818.393539

N	-2.713079	0.313962	-4.202893
C	-1.754497	0.887351	-3.266456
C	-1.656352	0.003395	-2.035820
N	-2.506289	-0.992720	-1.923042
C	-2.448989	-2.121635	-0.971399
C	-2.487369	-1.605176	0.469532
N	-3.420656	-2.012284	1.326271
C	-4.516961	-2.904710	0.993905
C	-4.070890	-4.356811	0.889385
O	-5.122504	-5.168643	0.881220
H	-4.821026	-6.085567	0.795199
H	-0.738836	0.913383	-3.676358
O	-0.771990	0.292027	-1.170389
H	-2.267061	-0.080312	-5.022279
H	-3.384571	1.003451	-4.518660
H	-3.121981	-1.048630	-2.739201
O	-1.586106	-0.817420	0.816815
H	-5.275606	-2.822471	1.772711
H	-5.009699	-2.620619	0.061516
O	-2.927848	-4.700396	0.795880
H	-0.973683	-0.212606	-0.241631
C	-2.144108	2.300575	-2.823178
H	-2.104181	2.972086	-3.681132
H	-1.460465	2.669189	-2.059676
H	-3.160172	2.304049	-2.419963
C	-3.307876	-1.651219	2.746664
H	-3.259407	-2.567405	3.338025
H	-4.179396	-1.066360	3.043472
H	-2.404866	-1.069519	2.898664
H	-3.314476	-2.737852	-1.200767
C	-1.172166	-2.943957	-1.181215
H	-1.153773	-3.773896	-0.476542
H	-0.283863	-2.330289	-1.026773

H -1.161732 -3.337649 -2.197990

SAA_0.00_NcB.xyz
34
0 eng= -818.69629015 zpe= -818.402478

N	-1.421869	0.701073	0.531505
C	-2.215905	-0.415255	1.106965
C	-3.341727	-0.700036	0.102062
N	-3.877541	-1.936511	0.105231
C	-3.300176	-3.105213	0.769254
C	-1.868194	-3.369712	0.284456
N	-1.640032	-3.224724	-1.046854
C	-0.298575	-3.373374	-1.561598
C	0.600859	-2.275525	-1.008050
O	1.874923	-2.601735	-1.109078
H	2.429825	-1.880762	-0.777509
H	-1.539342	-1.245069	1.305581
H	-2.668885	-0.078734	2.043028
O	-3.710384	0.210971	-0.613075
H	-0.795786	0.268741	-0.166541
H	-4.686362	-2.057215	-0.493980
O	-0.989287	-3.674385	1.063237
H	-2.367161	-2.884907	-1.658683
O	0.218119	-1.212061	-0.567395
H	-2.088788	1.300193	0.023297
C	-0.613614	1.460239	1.524152
H	-1.284710	1.916095	2.249494
H	-0.047324	2.227780	1.001285
H	0.064071	0.766057	2.017607
H	-3.216256	-2.925446	1.843868
C	-4.185379	-4.324139	0.532420
H	-3.744660	-5.194015	1.019090
H	-4.280038	-4.537218	-0.535136
H	-5.178227	-4.157422	0.952367
H	0.118653	-4.321807	-1.215385
C	-0.293337	-3.317375	-3.089127
H	-0.901602	-4.130557	-3.486020
H	0.721907	-3.430405	-3.467368
H	-0.695683	-2.365222	-3.444224

SAA_0.23_c-t_N-tccttgtt.xyz
34
0 eng= -818.69681505 zpe= -818.402391

N	-2.071884	-2.366545	-0.397891
C	-2.725374	-2.317178	-1.735288
C	-3.238031	-0.878598	-1.887737
N	-2.988267	-0.219375	-3.042803
C	-1.847855	-0.488253	-3.901917
C	-0.583448	-0.709795	-3.047169
N	0.456319	-1.268360	-3.698973

C	1.712269	-1.589778	-3.040926
C	1.482881	-2.599603	-1.919781
O	2.630930	-2.910738	-1.332530
H	2.475708	-3.576180	-0.646978
H	-2.007387	-2.677598	-2.468960
H	-3.596476	-2.974119	-1.712411
O	-3.879704	-0.429691	-0.963496
H	-2.625510	-1.748878	0.212215
H	-3.334019	0.733898	-3.029507
H	-2.031757	-1.396777	-4.486408
O	-0.552402	-0.429784	-1.859183
H	0.350817	-1.509923	-4.672856
H	2.330804	-2.111582	-3.776691
O	0.421784	-3.081802	-1.602361
H	-1.141769	-1.936695	-0.517332
C	-1.653015	0.689279	-4.857794
H	-0.836856	0.497717	-5.554123
H	-1.419849	1.596663	-4.296353
H	-2.563342	0.850718	-5.436429
C	2.475246	-0.351949	-2.553127
H	1.902732	0.179609	-1.794209
H	2.649718	0.312764	-3.399056
H	3.436444	-0.650113	-2.137215
C	-1.951439	-3.733313	0.179711
H	-1.427374	-3.664332	1.130683
H	-1.383410	-4.345191	-0.516223
H	-2.952422	-4.133266	0.329701

SAA_4.16_c-t_N-tccttgtt.xyz
34
0 eng= -818.695539933 zpe= -818.400895

N	-1.154548	-0.008021	1.135512
C	-2.106030	-0.049721	-0.010064
C	-1.981509	-1.464902	-0.589462
N	-1.836080	-1.622896	-1.924608
C	-1.260356	-0.636947	-2.830510
C	-0.062443	0.061174	-2.149525
N	0.360858	1.208718	-2.712537
C	1.478204	1.949692	-2.161050
C	1.135441	2.475748	-0.770546
O	2.152258	3.165600	-0.268652
H	1.921389	3.493016	0.612259
H	-3.118400	0.054828	0.383858
H	-1.870348	0.786390	-0.663301
O	-2.057594	-2.379938	0.202008
H	-0.219128	0.123730	0.721428
H	-1.743444	-2.596767	-2.185931
H	-0.822260	-1.213712	-3.650541
O	0.473313	-0.407807	-1.156255
H	-0.131553	1.603773	-3.499063

H	2.318749	1.262934	-2.016116
O	0.099027	2.289269	-0.180445
H	-1.176063	-0.947804	1.556402
C	-2.309804	0.306355	-3.427650
H	-1.912975	0.866195	-4.276944
H	-2.695944	1.017842	-2.694946
H	-3.144556	-0.287651	-3.798389
C	1.885506	3.079376	-3.104814
H	2.163583	2.664695	-4.074124
H	2.740497	3.616525	-2.699200
H	1.065098	3.788198	-3.241169
C	-1.450312	1.042757	2.148231
H	-0.678096	1.015302	2.914296
H	-2.422769	0.830748	2.588417
H	-1.447678	2.006376	1.646020

SAA_5.66_NtA.xyz
34
0 eng= -818.69453072 zpe= -818.400321

N	0.435721	1.811928	-2.941318
C	-0.769365	1.552036	-2.107645
C	-0.459334	0.254672	-1.356326
N	-1.498616	-0.540030	-1.065578
C	-1.278824	-1.961544	-0.741230
C	-0.932047	-2.566340	-2.109918
N	0.392399	-2.638733	-2.404338
C	0.784532	-2.798389	-3.795112
C	0.076287	-1.720316	-4.622644
O	-0.268143	-2.124547	-5.825777
H	-0.700235	-1.400945	-6.302461
H	-0.903302	2.383630	-1.413397
H	-1.632229	1.468046	-2.767893
O	0.706772	0.015420	-1.088570
H	0.467998	1.042580	-3.634770
H	-2.395801	-0.328637	-1.484846
O	-1.797982	-2.812929	-2.924614
H	1.043584	-2.211580	-1.760597
H	1.847093	-2.538994	-3.854398
O	-0.100312	-0.576959	-4.237169
H	1.245026	1.652097	-2.326274
C	0.475409	3.148905	-3.591798
H	-0.400351	3.252970	-4.229275
H	1.381890	3.221475	-4.188740
H	0.471964	3.916338	-2.820097
C	0.585597	-4.217284	-4.318342
H	1.137262	-4.904795	-3.677637
H	0.962429	-4.305723	-5.336545
H	-0.469862	-4.483672	-4.306900
H	-0.433447	-2.012231	-0.054338
C	-2.528908	-2.572553	-0.141899

```
H       -2.345057       -3.617860       0.107321
H       -3.347084       -2.541031       -0.863642
H       -2.813906       -2.040518       0.765918

SAA_6.21_NcA.xyz
34
0 eng= -818.694224523 zpe= -818.400111
N       -2.385923       -2.285682       0.090065
C       -2.072868       -2.057347       -1.358993
C       -3.378064       -1.686116       -2.071706
N       -3.449212       -0.475925       -2.680409
C       -2.582285       0.663248        -2.451196
C       -2.370903       0.894416        -0.943761
N       -1.322467       1.683173        -0.634700
C       -0.919055       1.961484        0.733943
C       -0.496376       0.673396        1.436397
O       -0.124931       0.920614        2.684211
H       0.187703        0.104483        3.100734
H       -1.286467       -1.306802       -1.404146
H       -1.706953       -2.994600       -1.778469
O       -4.284855       -2.488130       -2.073911
H       -3.014770       -1.518306       0.372667
H       -4.360581       -0.291529       -3.083797
H       -1.603075       0.482848        -2.910226
O       -3.088533       0.379028        -0.099634
H       -0.741040       2.031317        -1.382275
H       -0.010452       2.567817        0.677036
O       -0.467922       -0.427234       0.938403
H       -1.526038       -2.108884       0.621682
C       -3.210281       1.902912        -3.092125
H       -2.562334       2.771745        -2.978391
H       -4.170608       2.124128        -2.621381
H       -3.364621       1.730149        -4.157998
C       -1.970552       2.740214        1.533138
H       -2.878517       2.151720        1.655155
H       -2.211139       3.658861        0.998226
H       -1.572908       2.999102        2.513240
C       -2.965524       -3.623329       0.418504
H       -3.172167       -3.651799       1.486450
H       -2.233855       -4.385268       0.156104
H       -3.875447       -3.753924       -0.160928

SAA_9.16_c-t_N-gcggtgtt.xyz
34
0 eng= -818.693064004 zpe= -818.398991
C       -3.080633       -1.004877       -0.675093
H       -2.751257       -1.838279       -0.063100
H       -4.146813       -1.074699       -0.892106
C       -2.341960       -0.803417       -2.000155
O       -2.702189       0.109137        -2.709379
```

N	-2.849104	0.244910	0.106909
H	-3.173605	1.031761	-0.466701
N	-1.314036	-1.636618	-2.298695
H	-0.866311	-1.422675	-3.183215
C	-0.592143	-2.510963	-1.369847
H	-1.296545	-3.192517	-0.889484
C	-0.009747	-1.650129	-0.233370
O	-0.633263	-1.488730	0.807196
N	1.161919	-1.030489	-0.470456
H	1.636281	-1.166463	-1.350509
C	1.744340	-0.105904	0.481508
H	1.745109	-0.578497	1.470164
C	0.872035	1.136545	0.632752
O	1.454105	2.034627	1.413320
H	0.876913	2.806475	1.507177
O	-0.218613	1.299438	0.137482
H	-1.828114	0.383850	0.220852
C	0.430906	-3.329814	-2.144344
H	1.022741	-3.942204	-1.463876
H	1.110579	-2.696589	-2.722236
H	-0.082116	-3.988420	-2.845843
C	3.173667	0.245595	0.074430
H	3.769881	-0.665460	0.017338
H	3.622201	0.909209	0.810471
H	3.190560	0.745749	-0.896955
C	-3.492870	0.237298	1.448775
H	-3.034726	-0.561915	2.028768
H	-4.560665	0.063884	1.328453
H	-3.317228	1.198559	1.926931

SAA_9.24_NcB.xyz
34
0 eng= -818.692922919 zpe= -818.398957

N	-2.491093	-1.486631	-2.505085
C	-2.666925	-1.340480	-1.036315
C	-2.217239	0.085838	-0.692432
N	-1.723212	0.329254	0.538371
C	-1.289080	-0.666093	1.525784
C	-0.320282	-1.675039	0.876596
N	0.646856	-1.143222	0.084735
C	1.626165	-2.008127	-0.552623
C	0.887803	-3.018637	-1.430022
O	1.534511	-4.159584	-1.548693
H	1.049098	-4.747401	-2.145976
H	-3.731759	-1.420891	-0.804597
H	-2.123908	-2.148999	-0.550115
O	-2.363975	0.939104	-1.546224
H	-1.488418	-1.688167	-2.648409
H	-1.571493	1.311441	0.732161
O	-0.440856	-2.872255	1.031967

H	0.718766	-0.143686	-0.025160
H	2.180187	-1.386091	-1.264037
O	-0.150201	-2.785334	-2.014897
H	-2.701035	-0.566743	-2.918196
C	-3.293239	-2.584247	-3.110851
H	-3.008072	-3.516591	-2.627576
H	-3.073410	-2.632542	-4.175127
H	-4.349916	-2.378070	-2.952068
C	2.606637	-2.641401	0.432681
H	3.106270	-1.849996	0.991485
H	3.358634	-3.221747	-0.099824
H	2.082171	-3.297714	1.125972
H	-0.702486	-0.106697	2.259931
C	-2.432655	-1.365368	2.246544
H	-2.033943	-2.035100	3.007661
H	-3.031864	-1.971241	1.565403
H	-3.074754	-0.623615	2.721315

SAA_9.51_NcA.xyz
34
0 eng= -818.693502512 zpe= -818.398856

C	-0.807087	0.571621	-1.931644
H	-1.051279	1.286668	-2.717546
H	0.185672	0.163808	-2.107586
C	-1.946001	-0.450473	-1.847665
O	-3.076636	-0.039840	-1.704589
N	-0.742961	1.313771	-0.630087
H	0.202532	1.705334	-0.555051
N	-1.647143	-1.769111	-1.948594
H	-2.464709	-2.357413	-1.848308
C	-0.345162	-2.407915	-1.833394
H	-0.551957	-3.435659	-1.518721
C	0.445769	-1.802400	-0.657035
O	-0.113912	-1.220918	0.261811
N	1.782507	-1.962011	-0.688375
H	2.224622	-2.390869	-1.486942
C	2.632590	-1.436471	0.361021
H	2.206383	-1.716407	1.329790
C	2.629770	0.089465	0.336011
O	3.416672	0.578895	1.282792
H	3.408975	1.546448	1.249105
O	1.994132	0.778826	-0.426863
H	-0.777645	0.595813	0.110631
C	0.406552	-2.466248	-3.167597
H	1.246407	-3.163247	-3.127647
H	0.778105	-1.487122	-3.478049
H	-0.272715	-2.830234	-3.937718
C	4.048082	-1.996086	0.231945
H	4.673531	-1.624596	1.041102
H	4.499527	-1.699618	-0.718097

H	4.017047	-3.084533	0.286853
C	-1.783028	2.368255	-0.429318
H	-2.762644	1.903110	-0.490108
H	-1.669728	3.112450	-1.215349
H	-1.621298	2.822062	0.546390

SAA_11.70_NtA.xyz
34
0 eng= -818.691910406 zpe= -818.39802

N	-0.611605	1.842565	-2.978681
C	-1.546061	1.240572	-1.989277
C	-0.696406	0.987353	-0.737740
N	-1.345541	1.026462	0.435485
C	-0.648134	1.239840	1.724010
C	-0.147865	2.688235	1.623653
N	1.098862	2.878012	1.115223
C	1.433434	4.202530	0.645214
C	0.432924	4.652281	-0.415606
O	0.304857	5.963776	-0.442857
H	-0.318351	6.222501	-1.136987
H	-2.362778	1.941151	-1.816781
H	-1.932745	0.304140	-2.396042
O	0.496075	0.787585	-0.896881
H	0.248034	1.278047	-2.934049
H	-2.303032	1.350529	0.416452
O	-0.892968	3.614437	1.868190
H	1.580171	2.088302	0.706138
H	1.356707	4.910823	1.471705
O	-0.139681	3.913039	-1.195324
H	-0.339640	2.763747	-2.591100
C	-1.146548	1.955530	-4.361536
H	-0.391836	2.419580	-4.992793
H	-2.043669	2.571131	-4.339435
H	-1.383600	0.959658	-4.730733
C	2.840089	4.226803	0.049394
H	3.562407	3.943410	0.815080
H	3.086392	5.227369	-0.305430
H	2.916625	3.525296	-0.785423
H	-1.433462	1.258378	2.479254
C	0.364119	0.158825	2.069552
H	0.799093	0.380680	3.045137
H	1.162506	0.072525	1.335214
H	-0.141249	-0.805702	2.131935

SAA_13.28_NtB.xyz
34
0 eng= -818.691404325 zpe= -818.39742

N	-1.001083	-2.045366	-3.509882
C	-1.927191	-1.249989	-2.659497
C	-1.795154	-1.834200	-1.251093

N	-2.882467	-1.763871	-0.467584
C	-2.997968	-2.610238	0.733147
C	-3.308613	-3.991828	0.141839
N	-2.235282	-4.795977	-0.088991
C	-2.393161	-5.865562	-1.062433
C	-2.970543	-5.244964	-2.352396
O	-3.940865	-5.865570	-2.986964
H	-4.324694	-6.570120	-2.449117
H	-1.609369	-0.205785	-2.676605
H	-2.933311	-1.344950	-3.067433
O	-0.729418	-2.332473	-0.930177
H	-0.127196	-2.135963	-2.974927
H	-3.761426	-1.489632	-0.889199
H	-2.029054	-2.599708	1.233363
O	-4.426362	-4.280460	-0.236494
H	-1.316138	-4.390887	0.028222
O	-2.500728	-4.226122	-2.815104
H	-1.398961	-3.001487	-3.542669
C	-0.775206	-1.494422	-4.873237
H	-1.732521	-1.437299	-5.387520
H	-0.102659	-2.157692	-5.412655
H	-0.335075	-0.503040	-4.784570
C	-4.105197	-2.109086	1.638039
H	-5.066373	-2.161817	1.123607
H	-3.906190	-1.082834	1.946934
H	-4.167936	-2.736981	2.527135
H	-1.385943	-6.186785	-1.343417
C	-3.160821	-7.064606	-0.514683
H	-2.657879	-7.428924	0.380431
H	-3.170680	-7.886433	-1.235831
H	-4.180347	-6.789720	-0.239932

ASS_0.00_NcA.xyz
34
0 eng= -818.68649851 zpe= -818.392499

C	0.109861	1.245939	-2.238762
H	0.425389	1.382385	-1.207632
C	-0.943125	0.144816	-2.467319
O	-1.158235	-0.158000	-3.624196
N	1.318319	0.709961	-2.955534
H	2.097381	1.369990	-2.925090
H	1.049903	0.491219	-3.922566
N	-1.564720	-0.403288	-1.396744
C	-0.977389	-0.317731	-0.075468
H	-1.577568	-0.928511	0.601580
H	-1.015970	0.703906	0.312963
C	0.477910	-0.817040	-0.070796
O	0.972072	-1.298904	-1.085464
N	1.192740	-0.646620	1.059086
C	2.613867	-0.909616	0.974182

H	3.050318	-0.932448	1.972498
H	2.795040	-1.880152	0.505293
C	3.342148	0.131095	0.136346
O	4.653660	-0.004506	0.262688
H	5.108470	0.653328	-0.283320
O	2.810815	0.953834	-0.570047
H	1.601310	-0.161516	-2.474736
C	-0.332281	2.559234	-2.862015
H	-0.598732	2.415160	-3.910480
H	0.451696	3.314450	-2.777684
H	-1.214366	2.928259	-2.337156
C	-2.474100	-1.522088	-1.660939
H	-3.216338	-1.571066	-0.865501
H	-1.916477	-2.461431	-1.713968
H	-2.971607	-1.351963	-2.611927
C	0.694201	0.054907	2.240894
H	0.879827	1.131208	2.172347
H	1.202374	-0.336623	3.121047
H	-0.370394	-0.123931	2.373743

ASS_11.74_c-c_N-gcgtcgtt.xyz
34
0 eng= -818.681716681 zpe= -818.388026

N	0.298882	-0.811293	-5.644123
C	0.566784	-0.184171	-4.299841
C	-0.839324	0.300765	-3.893198
N	-1.336004	-0.041485	-2.685399
C	-0.804601	-1.172117	-1.953441
C	-0.860824	-2.471375	-2.783750
N	-0.555148	-3.625657	-2.152390
C	0.244376	-3.622891	-0.947097
C	1.683972	-3.211545	-1.246176
O	2.498452	-3.542197	-0.255247
H	3.397848	-3.243698	-0.457837
H	0.990535	-0.968066	-3.674546
O	-1.420193	0.988658	-4.712152
H	-0.281945	-1.651811	-5.481736
H	1.155061	-1.055352	-6.143666
H	-1.399748	-1.289840	-1.046728
H	0.225232	-0.986816	-1.639150
O	-1.176943	-2.432919	-3.967772
H	-0.150370	-2.941237	-0.189311
H	0.237885	-4.616696	-0.499556
O	2.017910	-2.631154	-2.246582
H	-0.252753	-0.135793	-6.191868
C	1.521604	0.988575	-4.447463
H	1.101930	1.745189	-5.112600
H	2.493060	0.659636	-4.821807
H	1.678276	1.446706	-3.469955
C	-2.718562	0.353008	-2.399805

H	-3.416207	-0.400220	-2.777165
H	-2.921729	1.300263	-2.891684
H	-2.839755	0.468021	-1.323547
C	-0.510269	-4.848688	-2.956840
H	-0.638452	-5.704739	-2.296122
H	0.444711	-4.930481	-3.485099
H	-1.319178	-4.827064	-3.681791

ASS_15.22_c-t_N-tcggtgtt.xyz
34
0 eng= -818.680796441 zpe= -818.386701

N	-3.754626	0.948574	-0.057185
C	-2.781743	-0.117430	-0.469763
C	-2.379396	0.294359	-1.897364
N	-1.186908	-0.095183	-2.383389
C	-0.120042	-0.629306	-1.535362
C	0.295346	0.311018	-0.394641
N	0.737800	1.558930	-0.701877
C	0.865314	2.464884	0.414055
C	-0.487858	2.881400	0.961401
O	-0.355472	3.711500	1.979099
H	-1.224401	3.969275	2.318923
H	-1.967129	-0.104812	0.250557
O	-3.216374	0.884723	-2.563881
H	-4.397259	0.628167	0.667614
H	-4.271426	1.217010	-0.912037
H	0.734968	-0.852630	-2.173190
H	-0.417228	-1.562538	-1.052793
O	0.180365	-0.061541	0.759572
H	1.414052	1.997592	1.234651
H	1.407887	3.362353	0.113336
O	-1.555712	2.508209	0.528134
H	-3.223292	1.768664	0.276577
C	-3.475102	-1.474945	-0.511687
H	-2.773057	-2.232546	-0.861944
H	-4.324843	-1.456731	-1.197852
H	-3.811128	-1.770783	0.483947
C	-1.000161	0.072350	-3.830845
H	-0.067172	-0.404635	-4.123356
H	-0.981886	1.128022	-4.104760
H	-1.827740	-0.399948	-4.359569
C	0.808778	2.114275	-2.047151
H	1.616583	2.845077	-2.082282
H	-0.129324	2.600666	-2.333681
H	1.047244	1.334652	-2.767400

ASS_18.50_NcD.xyz
34
0 eng= -818.679244866 zpe= -818.385454

N	-0.629349	-2.056419	-1.616948

C	-1.751929	-1.130173	-1.997669
C	-2.309343	-0.677671	-0.635713
N	-2.407651	0.648462	-0.373730
C	-1.619594	1.610866	-1.115314
C	-0.123697	1.256157	-1.085653
N	0.711802	1.984719	-1.857985
C	2.049906	1.458189	-2.009847
C	2.048247	0.119248	-2.753209
O	3.236157	-0.450689	-2.927183
H	3.962366	0.078781	-2.575733
H	-1.287240	-0.348862	-2.592468
O	-2.644529	-1.560168	0.128746
H	0.058415	-1.507136	-1.075928
H	-0.160358	-2.436305	-2.442567
H	-1.774259	2.594087	-0.666823
H	-1.955915	1.687448	-2.153125
O	0.277483	0.305198	-0.423660
H	2.661813	2.178828	-2.556354
H	2.502627	1.294311	-1.027644
O	1.048416	-0.423797	-3.139447
H	-1.007087	-2.802190	-1.021199
C	-2.804739	-1.881740	-2.795000
H	-2.382995	-2.279824	-3.719902
H	-3.612396	-1.197705	-3.058871
H	-3.229488	-2.692416	-2.200380
C	-2.840420	1.025640	0.974750
H	-1.988017	1.037686	1.659887
H	-3.565604	0.296552	1.326029
H	-3.304166	2.010327	0.935975
C	0.262610	3.006687	-2.802275
H	1.060212	3.736246	-2.936937
H	-0.599498	3.539122	-2.407184
H	0.011633	2.566709	-3.772293

ASS_20.65_c-c_N-gcgtcgtt.xyz
34
0 eng= -818.678118202 zpe= -818.384632

C	-1.251864	-1.260968	-5.121047
H	-1.071062	-0.208665	-4.923126
C	-1.424454	-2.131823	-3.862287
O	-1.375022	-3.334095	-4.049742
N	0.027529	-1.802452	-5.700714
H	0.218704	-1.455101	-6.641197
H	-0.054107	-2.829029	-5.711561
N	-1.640359	-1.552479	-2.662604
C	-1.221549	-0.193141	-2.376402
H	-1.589652	0.047068	-1.383793
H	-1.680723	0.522753	-3.064592
C	0.309515	-0.047919	-2.491152
O	0.896133	-0.704715	-3.351366

N	0.941713	0.840421	-1.704661
C	0.315246	1.478967	-0.564535
H	0.951033	2.295819	-0.223487
H	-0.646658	1.929756	-0.820384
C	0.098486	0.501156	0.585621
O	-0.141076	1.150106	1.720824
H	-0.302707	0.509268	2.429437
O	0.108571	-0.690729	0.459720
H	0.783619	-1.536311	-5.046200
C	-2.404015	-1.468217	-6.089431
H	-2.542470	-2.530326	-6.297726
H	-2.237628	-0.925709	-7.022364
H	-3.322880	-1.089139	-5.640171
C	-1.726999	-2.457466	-1.507624
H	-0.729519	-2.688541	-1.126310
H	-2.220387	-3.373631	-1.819425
H	-2.306985	-1.970819	-0.725173
C	2.404645	0.915905	-1.755074
H	2.838051	0.315398	-0.950666
H	2.711317	1.955477	-1.644058
H	2.748389	0.532332	-2.710344

ASS_20.95_NcA.xyz
34
0 eng= -818.678195937 zpe= -818.38452

N	-3.077953	0.684231	-0.028688
C	-2.919984	-0.685302	-0.632985
C	-3.890164	-0.617249	-1.827621
N	-3.464011	-0.970000	-3.058820
C	-2.061674	-0.924132	-3.432038
C	-1.534420	0.520836	-3.350000
N	-0.603048	0.923446	-4.230348
C	-0.397719	2.356591	-4.323729
C	-1.580700	2.990510	-5.047907
O	-1.410381	4.303736	-5.176742
H	-2.171033	4.676440	-5.646073
H	-1.872582	-0.774348	-0.906837
O	-5.021040	-0.253310	-1.560366
H	-2.627532	0.772973	0.882977
H	-4.088571	0.864414	0.055342
H	-1.990291	-1.319246	-4.442319
H	-1.446596	-1.570341	-2.800075
O	-1.972394	1.267239	-2.472604
H	-0.319028	2.801079	-3.332131
H	0.521368	2.571266	-4.869138
O	-2.531427	2.377904	-5.444707
H	-2.677864	1.354721	-0.707420
C	-3.346009	-1.755463	0.357585
H	-3.249481	-2.736570	-0.109224
H	-4.390389	-1.617925	0.642503

H	-2.714118	-1.742826	1.248009
C	-4.404976	-0.771315	-4.169819
H	-4.226287	-1.538035	-4.922969
H	-4.262226	0.221872	-4.607827
H	-5.419467	-0.857827	-3.792349
C	-0.180562	0.116561	-5.376724
H	0.746034	0.536752	-5.763735
H	-0.935778	0.131395	-6.167527
H	0.031240	-0.906381	-5.072195

ASS_22.81_c-t_O1-tcgttgtt.xyz
34
0 eng= -818.675691852 zpe= -818.383813

N	-3.953563	0.219858	0.295352
C	-3.026908	-0.302888	-0.707260
C	-3.040715	0.740911	-1.823407
N	-2.146904	0.826258	-2.769236
C	-0.856832	0.172150	-2.594232
C	-0.117296	0.835843	-1.413472
N	1.112440	0.356088	-1.116661
C	1.607478	0.714958	0.196495
C	0.800467	-0.000664	1.272379
O	1.287322	0.250226	2.481221
H	0.758443	-0.216947	3.144074
O	-4.063652	1.520401	-1.833705
H	-3.467909	0.717400	1.035171
H	-4.514700	-0.512229	0.715291
H	-0.300867	0.271207	-3.526058
H	-0.981703	-0.894451	-2.395938
O	-0.668781	1.712369	-0.776656
H	2.659259	0.443117	0.289582
H	1.516263	1.789204	0.362432
O	-0.155150	-0.698740	1.053029
H	-4.532212	1.300373	-0.968622
C	-2.214928	1.904634	-3.767373
H	-1.843730	1.519502	-4.715513
H	-1.596610	2.738254	-3.427016
H	-3.243574	2.232217	-3.882030
C	1.658657	-0.859851	-1.713787
H	2.744204	-0.830874	-1.628758
H	1.421082	-0.913638	-2.774468
H	1.283630	-1.754332	-1.205759
H	-2.017919	-0.421433	-0.310151
C	-3.548031	-1.627802	-1.277394
H	-2.949653	-1.970961	-2.121785
H	-4.581542	-1.518445	-1.614683
H	-3.508013	-2.391388	-0.499732

ASS_23.83_t-t_N-gtgttgtt.xyz
34

0 eng= -818.676964183 zpe= -818.383423
N -1.224800 1.562606 0.847644
C -2.050606 1.163847 -0.358143
C -0.982214 0.408995 -1.166007
N -1.006357 -0.944145 -1.215274
C 0.339074 -1.506476 -1.171318
C 0.997386 -0.961276 0.111650
N 2.306223 -0.667747 0.101252
C 2.871203 -0.136080 1.324457
C 2.491484 1.313419 1.587756
O 3.298636 1.840362 2.500970
H 3.032480 2.753578 2.681525
O -0.021294 1.061422 -1.530393
H -0.400110 2.105417 0.541570
H -0.809255 0.719160 1.293013
H 0.887483 -1.242591 -2.071180
H 0.272430 -2.593102 -1.098282
O 0.294840 -0.799108 1.113081
H 2.507997 -0.710709 2.180598
H 3.956561 -0.221703 1.302033
O 1.575035 1.908018 1.079596
H -1.763345 2.102293 1.526236
C -2.113187 -1.764615 -0.730438
H -2.078771 -1.885188 0.356719
H -2.039397 -2.745770 -1.197660
H -3.064348 -1.335922 -1.041524
C 3.087417 -0.582706 -1.138909
H 4.114140 -0.333856 -0.880593
H 2.685871 0.196778 -1.790256
H 3.099460 -1.540771 -1.658011
H -2.847202 0.524728 0.016598
C -2.578720 2.395884 -1.063885
H -3.181787 2.092131 -1.920297
H -1.749664 3.004191 -1.428860
H -3.210910 2.989635 -0.400849

ASS_24.87_c-t_N-gcggtgtt.xyz
34
0 eng= -818.67777472 zpe= -818.383026
N -1.492166 -0.610131 0.606388
C -2.125878 -1.689877 -0.229578
C -2.280272 -1.038241 -1.616261
N -1.651480 -1.576568 -2.691266
C -0.462264 -2.422599 -2.566030
C 0.654911 -1.653271 -1.849957
N 1.442472 -0.800165 -2.534462
C 2.321507 0.041357 -1.746477
C 1.553930 1.092913 -0.962274
O 2.375758 2.022326 -0.496889
H 1.875998 2.672261 0.018658

H	-1.436769	-2.527575	-0.199880
O	-3.001342	-0.062272	-1.681061
H	-1.970113	0.280272	0.415488
H	-0.500770	-0.506297	0.329967
H	-0.188678	-2.771457	-3.558506
H	-0.667505	-3.306009	-1.962968
O	0.753709	-1.768278	-0.628697
H	2.877046	-0.556932	-1.019491
H	3.041186	0.536291	-2.398033
O	0.364318	1.079584	-0.760218
H	-1.551308	-0.822764	1.603633
C	-3.491105	-2.050022	0.333372
H	-3.398782	-2.452552	1.344338
H	-3.946838	-2.818560	-0.292062
H	-4.148894	-1.180041	0.334589
C	-1.948365	-0.940772	-3.978988
H	-1.634267	-1.607691	-4.780494
H	-1.450158	0.027104	-4.072226
H	-3.021572	-0.775682	-4.048435
C	1.384527	-0.507511	-3.963555
H	0.752780	-1.219329	-4.484152
H	2.388797	-0.572500	-4.384926
H	0.991274	0.498827	-4.129594

ASS_24.97_c-c_O1-tcgtcgtt.xyz
34
0 eng= -818.674562107 zpe= -818.382989

N	-0.451692	-0.578482	-6.709624
C	-1.288021	-0.645136	-5.512554
C	-1.226563	-2.110131	-5.081680
N	-1.586329	-2.551742	-3.909289
C	-1.733141	-1.610487	-2.807027
C	-0.341250	-1.032674	-2.454430
N	-0.281935	-0.190323	-1.394529
C	-1.460126	0.465326	-0.874651
C	-1.993103	1.527062	-1.833481
O	-2.755283	2.410571	-1.204373
H	-3.101954	3.052934	-1.841761
O	-0.851200	-2.937369	-5.992600
H	-0.843187	0.031753	-7.417584
H	0.492114	-0.267971	-6.501546
H	-2.159259	-2.150054	-1.960774
H	-2.417041	-0.808069	-3.081908
O	0.620913	-1.357874	-3.119268
H	-2.272354	-0.240758	-0.677742
H	-1.224279	0.930079	0.082598
O	-1.775284	1.528967	-3.017208
H	-0.563313	-2.336887	-6.748556
C	-1.416064	-3.970301	-3.560226
H	-0.451999	-4.094614	-3.062044

```
H       -1.446374       -4.574537       -4.461692
H       -2.227383       -4.261146       -2.895328
C       0.998270        0.469435        -1.126401
H       1.061258        0.696067        -0.062780
H       1.085018        1.393204        -1.706839
H       1.806342        -0.200753       -1.405322
H       -0.927204       -0.003033       -4.708545
C       -2.739787       -0.291109       -5.853387
H       -3.092869       -0.891238       -6.695593
H       -2.793037       0.765026        -6.119485
H       -3.407936       -0.458120       -5.008458

SSS_0.00_NcA.xyz
34
0 eng= -818.678209702 zpe= -818.383537
C       -0.708375       0.604340        -2.043604
H       -1.026612       1.208922        -2.894337
H       0.278147        0.187720        -2.228968
C       -1.853662       -0.363410       -1.706060
O       -2.882634       0.153316        -1.316983
N       -0.598377       1.498933        -0.856855
H       -1.564657       1.665043        -0.541249
N       -1.669613       -1.691599       -1.875405
C       -0.332935       -2.248676       -1.877257
H       0.215549        -1.967821       -2.781193
H       -0.421208       -3.336127       -1.892616
C       0.459555        -1.803075       -0.635705
O       -0.058613       -1.071754       0.200255
N       1.745414        -2.199259       -0.539935
C       2.528463        -1.592200       0.514506
H       2.009454        -1.672933       1.472819
H       3.487744        -2.101082       0.608084
C       2.773882        -0.112918       0.263519
O       3.655326        0.367001        1.129335
H       3.799268        1.310095        0.965141
O       2.230643        0.549609        -0.586971
H       -0.131830       0.940467        -0.125484
C       0.122074        2.776488        -1.108296
H       1.121074        2.537242        -1.464117
H       0.177441        3.334904        -0.176051
H       -0.433824       3.344484        -1.851740
C       -2.764663       -2.567910       -1.449021
H       -2.705182       -3.502437       -2.005127
H       -3.710196       -2.077205       -1.663072
H       -2.700539       -2.763594       -0.374825
C       2.463335        -2.918622       -1.589781
H       3.217120        -3.555176       -1.127847
H       2.955140        -2.225485       -2.279150
H       1.788715        -3.563759       -2.147317
```

SSS_7.43_NcA.xyz
34
0 eng= -818.675417357 zpe= -818.380706

C	-1.675443	0.104227	-1.877829
H	-0.635954	0.301036	-1.629327
H	-1.969665	0.719043	-2.728706
C	-2.695252	0.320392	-0.751060
O	-3.859867	0.083052	-0.998056
N	-1.755876	-1.335639	-2.294631
H	-0.844385	-1.592267	-2.688091
N	-2.250302	0.772428	0.448213
C	-0.856862	0.679054	0.825963
H	-0.766210	1.004880	1.863773
H	-0.235510	1.357831	0.234519
C	-0.322241	-0.754792	0.690702
O	-1.067205	-1.669881	0.358201
N	0.998159	-0.946264	0.896598
C	1.530623	-2.239820	0.529909
H	2.539564	-2.356924	0.925214
H	0.912495	-3.040377	0.943734
C	1.561618	-2.435703	-0.977708
O	2.249016	-3.520179	-1.297597
H	2.256644	-3.637360	-2.258850
O	1.016953	-1.717378	-1.783060
H	-1.819953	-1.880220	-1.420069
C	-2.865531	-1.671956	-3.238011
H	-2.833896	-2.741467	-3.435896
H	-2.709189	-1.113332	-4.159104
H	-3.804650	-1.385199	-2.772781
C	-3.237962	0.866734	1.526918
H	-4.198035	1.143119	1.099812
H	-2.919349	1.632820	2.232395
H	-3.339475	-0.095048	2.037639
C	1.949670	0.136578	1.139689
H	2.332549	0.548728	0.200895
H	2.783261	-0.253892	1.721927
H	1.489692	0.930315	1.723677

SSS_10.81_c-c_N-gcgtcgtt.xyz
34
0 eng= -818.674099862 zpe= -818.379419

C	-2.376349	-3.147825	-1.886402
H	-1.500356	-2.506871	-1.809404
H	-3.158941	-2.647211	-2.458316
C	-2.991093	-3.679053	-0.584642
O	-3.856004	-4.524085	-0.713773
N	-1.978372	-4.380415	-2.629209
H	-2.793901	-5.006681	-2.609914
N	-2.543867	-3.202607	0.597765
C	-1.265920	-2.528629	0.693450

H	-1.092495	-2.291384	1.744300
H	-1.282791	-1.580611	0.150625
C	-0.112227	-3.406649	0.166498
O	-0.353274	-4.476131	-0.381279
N	1.147817	-2.951257	0.344764
C	1.405846	-1.541509	0.536173
H	0.796084	-1.112937	1.335689
H	2.445039	-1.393391	0.829766
C	1.125162	-0.757057	-0.743523
O	1.666540	0.450598	-0.701556
H	1.446929	0.936338	-1.510843
O	0.465853	-1.183805	-1.656851
H	-1.249437	-4.825303	-2.046869
C	-1.488152	-4.136929	-4.011971
H	-1.205517	-5.087588	-4.459001
H	-0.626589	-3.473493	-3.956003
H	-2.284483	-3.670771	-4.588829
C	-3.062518	-3.837460	1.812326
H	-2.452971	-4.706125	2.077858
H	-4.082518	-4.163776	1.628646
H	-3.052129	-3.111857	2.624613
C	2.241474	-3.736851	-0.230207
H	3.160053	-3.501001	0.305109
H	2.366183	-3.508959	-1.293570
H	2.016800	-4.793538	-0.115550

SSS_11.45_c-t_N-tcgttgtt.xyz
34
0 eng= -818.673553464 zpe= -818.379175

C	-1.641420	1.124939	0.012506
H	-1.976068	1.909948	-0.669000
H	-0.558040	1.024809	-0.027327
C	-2.451308	-0.157799	-0.266610
O	-3.458308	-0.322051	0.403144
N	-2.019043	1.505781	1.404333
H	-3.003572	1.192211	1.493234
N	-2.044077	-0.964413	-1.260287
C	-0.689700	-0.886269	-1.764600
H	-0.474443	0.107120	-2.170911
H	-0.596894	-1.587413	-2.594894
C	0.326430	-1.214076	-0.656084
O	-0.057488	-1.448953	0.476373
N	1.639600	-1.162133	-0.989187
C	2.565558	-1.144044	0.120232
H	2.333163	-1.941775	0.828234
H	3.585248	-1.294599	-0.235348
C	2.488011	0.175072	0.873916
O	3.428580	0.243105	1.808192
H	3.370907	1.093261	2.267080
O	1.677214	1.043421	0.665807

H -1.969157 2.515878 1.535554
C -1.197687 0.800143 2.440304
H -1.588397 1.055699 3.422790
H -0.164319 1.119886 2.324221
H -1.276801 -0.268824 2.250413
C -2.755365 -2.231080 -1.450272
H -2.750346 -2.484043 -2.509937
H -3.779781 -2.113609 -1.108158
H -2.270000 -3.022381 -0.872650
C 2.121090 -0.649485 -2.269121
H 3.113039 -1.058042 -2.458044
H 2.181324 0.443977 -2.265244
H 1.476822 -0.976032 -3.082577

SSS_14.86_c-t_N-tcggtgtt.xyz
34
0 eng= -818.672750827 zpe= -818.377877
C -2.329486 -0.097263 0.989496
H -1.421283 -0.540442 1.392089
H -3.203431 -0.603627 1.408013
C -2.472905 -0.099575 -0.539019
O -3.079259 0.833901 -1.041691
N -2.381084 1.333869 1.394182
H -3.006265 1.791863 0.712008
N -2.004061 -1.144188 -1.244039
C -1.136747 -2.169578 -0.661426
H -0.897537 -2.889666 -1.443864
H -1.639321 -2.715056 0.139417
C 0.151496 -1.610554 -0.041807
O 0.375786 -1.795680 1.141451
N 0.990817 -0.878902 -0.821696
C 2.041063 -0.182378 -0.118634
H 2.560584 -0.853256 0.568871
H 2.774157 0.209860 -0.824881
C 1.501786 0.973488 0.704701
O 2.474664 1.594455 1.346780
H 2.119129 2.333091 1.861401
O 0.336051 1.295485 0.770683
H -1.436261 1.711953 1.231151
C -2.803927 1.567897 2.800826
H -2.119639 1.040556 3.462886
H -3.816117 1.189542 2.929127
H -2.776223 2.636410 3.003318
C -2.406108 -1.194514 -2.656008
H -2.058103 -2.129642 -3.089083
H -1.991963 -0.350215 -3.208531
H -3.492559 -1.146895 -2.727473
C 0.743574 -0.525920 -2.213847
H 0.231086 -1.337158 -2.726763
H 1.701292 -0.381615 -2.713106

H 0.151243 0.390718 -2.299634

SSS_17.29_c-c_N-tcgtcgtt.xyz
34
0 eng= -818.670846789 zpe= -818.376951

C	-1.038304	-4.082426	1.505732
H	-1.569615	-4.047830	2.459363
H	-0.217797	-3.367678	1.518704
C	-2.060910	-3.926079	0.363560
O	-2.532550	-4.956656	-0.088655
N	-0.481905	-5.454237	1.321036
H	-1.278825	-5.998947	0.942142
N	-2.419838	-2.685440	-0.006434
C	-1.561345	-1.559078	0.294194
H	-1.401548	-1.471403	1.371177
H	-2.068804	-0.649465	-0.030878
C	-0.207151	-1.703528	-0.433472
O	0.036675	-2.723240	-1.051459
N	0.656219	-0.657832	-0.354320
C	0.569688	0.296752	0.725830
H	1.226046	1.143057	0.521922
H	-0.437064	0.709804	0.834438
C	0.960388	-0.329863	2.061169
O	1.258392	0.593119	2.964250
H	1.473694	0.167364	3.807438
O	0.962025	-1.516432	2.270687
H	-0.190263	-5.855175	2.212478
C	0.639848	-5.504953	0.328875
H	0.941987	-6.542319	0.202508
H	1.462325	-4.903150	0.710151
H	0.275628	-5.083035	-0.605968
C	-3.304102	-2.562891	-1.168603
H	-3.998589	-3.398553	-1.173177
H	-2.715982	-2.578579	-2.090367
H	-3.857652	-1.628147	-1.092306
C	1.977362	-0.829766	-0.959974
H	2.650593	-1.367803	-0.284680
H	2.390586	0.153185	-1.182587
H	1.872702	-1.396557	-1.881039

SSS_17.48_NcD.xyz
34
0 eng= -818.671485041 zpe= -818.376881

C	-2.282946	-1.859225	-1.404238
H	-1.265367	-2.123039	-1.678364
H	-2.985532	-2.577034	-1.830696
C	-2.785529	-0.450403	-1.755109
O	-3.842622	-0.124651	-1.252001
N	-2.380781	-1.946621	0.080504
H	-3.252905	-1.461084	0.333307

N	-2.061177	0.320256	-2.598853
C	-0.642906	0.083523	-2.768892
H	-0.239256	0.881147	-3.394622
H	-0.454684	-0.854504	-3.299539
C	0.083454	0.055904	-1.414217
O	-0.539308	0.215340	-0.371959
N	1.409871	-0.209106	-1.415933
C	1.985790	-0.500041	-0.122733
H	3.066135	-0.621450	-0.226360
H	1.794661	0.324533	0.569812
C	1.381771	-1.771023	0.479129
O	1.817868	-2.110333	1.688768
H	2.489213	-1.505785	2.028182
O	0.540539	-2.428207	-0.072205
H	-1.602956	-1.381349	0.450748
C	-2.344904	-3.332010	0.627438
H	-2.391859	-3.277962	1.713058
H	-1.411703	-3.794672	0.316023
H	-3.204805	-3.875313	0.240496
C	-2.546794	1.684856	-2.823790
H	-2.177484	2.036082	-3.786339
H	-2.205892	2.348896	-2.024239
H	-3.633255	1.673387	-2.833782
C	2.157446	-0.621943	-2.601928
H	2.122813	-1.708007	-2.731775
H	3.194613	-0.306948	-2.492137
H	1.763311	-0.137091	-3.491777

SGG_0.00_NcA.xyz
28
0 eng= -740.081387043 zpe= -739.843501

C	-0.708635	0.563284	-1.985743
H	-1.023026	1.165964	-2.839343
H	0.276852	0.140907	-2.170839
C	-1.849620	-0.412167	-1.663050
O	-2.889159	0.071338	-1.276286
N	-0.600824	1.458497	-0.800042
H	-1.563123	1.607929	-0.465231
N	-1.644482	-1.736542	-1.864553
H	-2.435270	-2.303626	-1.584162
C	-0.334197	-2.344862	-1.818992
H	0.222331	-2.166229	-2.744418
H	-0.459432	-3.425098	-1.729442
C	0.481291	-1.831090	-0.623518
O	-0.014962	-1.122689	0.236171
N	1.785425	-2.169547	-0.617927
H	2.171513	-2.713249	-1.374934
C	2.668275	-1.632974	0.388934
H	2.280429	-1.838955	1.390445
H	3.649970	-2.099337	0.312353

C	2.825048	-0.125308	0.269932
O	3.738133	0.309030	1.125481
H	3.830859	1.269806	1.051540
O	2.200197	0.587960	-0.476208
H	-0.105010	0.917271	-0.075910
C	0.087116	2.753100	-1.063337
H	1.085437	2.536054	-1.434338
H	0.145866	3.311201	-0.131218
H	-0.493441	3.308238	-1.797513

SGG_7.21_NcA.xyz
28
0 eng= -740.079171341 zpe= -739.840754

C	-0.857287	0.481304	-1.974011
H	-1.165160	1.167041	-2.763547
H	0.128843	0.083077	-2.203748
C	-1.964511	-0.563792	-1.786578
O	-3.090158	-0.183914	-1.562138
N	-0.730302	1.263423	-0.700672
H	0.220654	1.648079	-0.671680
N	-1.625041	-1.874572	-1.897602
H	-2.397021	-2.502142	-1.711949
C	-0.284942	-2.404473	-1.829292
H	0.271879	-2.210287	-2.751796
H	-0.352710	-3.488952	-1.724913
C	0.495712	-1.847050	-0.633441
O	-0.048972	-1.231275	0.268099
N	1.828549	-2.045393	-0.669267
H	2.247589	-2.509853	-1.460802
C	2.682443	-1.445322	0.325896
H	2.321969	-1.678152	1.331534
H	3.694695	-1.838969	0.238064
C	2.728533	0.069755	0.204322
O	3.621096	0.573372	1.040105
H	3.647079	1.538037	0.958748
O	2.039956	0.732385	-0.534227
H	-0.745561	0.573289	0.066316
C	-1.755279	2.333201	-0.498624
H	-2.740184	1.875299	-0.533323
H	-1.647254	3.063328	-1.298495
H	-1.573166	2.801325	0.466531

SGG_7.92_NcB.xyz
28
0 eng= -740.077915942 zpe= -739.840483

C	-2.319816	-0.059346	0.862888
H	-1.433676	-0.516689	1.300187
H	-3.216485	-0.533856	1.270038
C	-2.403818	-0.103983	-0.669725
O	-2.916014	0.837004	-1.241411

N	-2.331502	1.380050	1.232533
H	-2.915212	1.854070	0.526641
N	-1.940462	-1.210341	-1.286636
H	-2.124363	-1.254056	-2.281003
C	-1.146966	-2.248701	-0.644186
H	-0.913100	-3.007689	-1.391578
H	-1.690588	-2.740519	0.164094
C	0.153395	-1.704739	-0.043757
O	0.521385	-2.036462	1.061143
N	0.836607	-0.808484	-0.804933
H	0.444207	-0.477066	-1.672368
C	1.973247	-0.137344	-0.235159
H	2.643214	-0.856107	0.241138
H	2.537604	0.381156	-1.011833
C	1.566029	0.877951	0.818689
O	2.620520	1.363598	1.443242
H	2.347974	2.012908	2.107699
O	0.426939	1.215656	1.056458
H	-1.364304	1.713643	1.102197
C	-2.781055	1.661067	2.623826
H	-2.125511	1.130736	3.312111
H	-3.807297	1.317317	2.737234
H	-2.722374	2.732857	2.800071

SGG_10.27_NtA.xyz
28
0 eng= -740.077729228 zpe= -739.839588

C	-0.858171	1.929944	-0.893183
H	-1.886001	2.122943	-0.586628
H	-0.637767	2.459515	-1.821963
C	-0.537827	0.439887	-1.030047
O	0.594398	0.070529	-0.767249
N	0.056437	2.430050	0.168946
H	0.975323	2.010510	-0.030957
N	-1.538207	-0.361402	-1.421445
H	-2.477070	0.013409	-1.447731
C	-1.423972	-1.808073	-1.215197
H	-0.438409	-2.136343	-1.541457
H	-2.198634	-2.311868	-1.787644
C	-1.677456	-2.002668	0.278294
O	-2.801184	-2.014936	0.727874
N	-0.564237	-2.002148	1.066820
H	0.321227	-1.769263	0.638638
C	-0.748929	-1.747386	2.468836
H	-1.505750	-2.414777	2.882260
H	0.181665	-1.918248	3.014038
C	-1.191435	-0.316229	2.731860
O	-1.673099	-0.176413	3.950799
H	-1.945874	0.739895	4.102464
O	-1.092970	0.602191	1.941256

H	-0.251114	1.983424	1.049911
C	0.126815	3.911381	0.292430
H	0.490876	4.324642	-0.646165
H	0.807916	4.163185	1.102409
H	-0.869345	4.292184	0.509203

SGG_14.24_c-t_N-tcgttgtt.xyz
28
0 eng= -740.076187801 zpe= -739.838079

N	0.253867	0.526103	-3.347538
C	0.055783	-0.856678	-2.824754
C	-1.366671	-1.242461	-3.266300
N	-1.991608	-2.230209	-2.596870
C	-1.641173	-2.640800	-1.257504
C	-1.652253	-1.452723	-0.283680
N	-1.204504	-1.729250	0.962404
C	-0.912503	-0.643985	1.864750
C	0.254015	0.198035	1.369364
O	0.541486	1.151788	2.246542
H	1.294690	1.670961	1.930410
H	0.763635	-1.522710	-3.323367
H	0.242337	-0.843686	-1.751881
O	-1.817313	-0.683368	-4.245936
H	1.244234	0.712539	-3.507377
H	-2.933574	-2.406828	-2.923016
H	-0.653084	-3.111888	-1.238376
H	-2.356521	-3.395735	-0.928913
O	-1.990273	-0.341264	-0.635019
H	-0.834091	-2.644997	1.167201
H	-1.777955	0.015368	1.962742
H	-0.676737	-1.030779	2.856009
O	0.836449	0.037770	0.326818
H	-0.245402	0.542248	-4.253290
C	-0.336044	1.579332	-2.456603
H	-1.393418	1.355150	-2.333031
H	-0.192014	2.547739	-2.930662
H	0.167584	1.523082	-1.494006

SGG_14.98_c-t_N-gcggtgtt.xyz
28
0 eng= -740.075752789 zpe= -739.837796

C	-3.035966	-1.011117	-0.694930
H	-2.697405	-1.842364	-0.084864
H	-4.102839	-1.090498	-0.904985
C	-2.308039	-0.806990	-2.025578
O	-2.675073	0.100832	-2.735674
N	-2.809639	0.240941	0.085143
H	-3.163021	1.023017	-0.477671
N	-1.274663	-1.634495	-2.326415
H	-0.841706	-1.433554	-3.219378

C	-0.574714	-2.510882	-1.392700
H	0.205748	-3.029987	-1.948832
H	-1.241546	-3.266453	-0.976711
C	0.008771	-1.695962	-0.234231
O	-0.603280	-1.548647	0.812070
N	1.189726	-1.085033	-0.467262
H	1.648693	-1.193884	-1.358379
C	1.746233	-0.173056	0.501493
H	1.785509	-0.644649	1.488187
H	2.764400	0.094636	0.221661
C	0.926076	1.095932	0.652216
O	1.578245	1.987404	1.377607
H	1.039655	2.785693	1.483431
O	-0.178264	1.277933	0.196722
H	-1.789830	0.403689	0.180751
C	-3.424033	0.218408	1.441724
H	-2.949281	-0.583769	2.003947
H	-4.493616	0.041947	1.343804
H	-3.240757	1.176628	1.922994

SGG_16.97_t-t_N-gtgttgtt.xyz
28
0 eng= -740.075551949 zpe= -739.837037

C	-2.109471	0.701828	-2.609817
H	-2.388497	1.723790	-2.863099
H	-2.808523	-0.010751	-3.046915
C	-1.884902	0.554087	-1.105763
O	-1.117628	1.321497	-0.571821
N	-0.743700	0.431953	-3.166522
H	-0.072639	1.047537	-2.682113
N	-2.369539	-0.562866	-0.495972
H	-2.850836	-1.230103	-1.082118
C	-1.451224	-1.138722	0.476084
H	-1.860671	-2.078564	0.846450
H	-1.314263	-0.454394	1.312206
C	-0.133806	-1.409132	-0.269128
O	-0.155891	-1.783258	-1.437695
N	1.010246	-1.194558	0.395500
H	0.978528	-0.755114	1.303153
C	2.286605	-1.375205	-0.253592
H	3.087474	-1.317615	0.482400
H	2.334976	-2.364473	-0.717588
C	2.555570	-0.361417	-1.356153
O	3.822778	-0.444889	-1.742291
H	3.990468	0.190561	-2.452858
O	1.751924	0.392520	-1.842351
H	-0.446387	-0.511996	-2.853427
C	-0.629401	0.575747	-4.642110
H	0.401852	0.378198	-4.926975
H	-0.907906	1.591019	-4.917853

H   -1.294541   -0.140855   -5.119911

SGG_18.01_NcC.xyz
28
0 eng= -740.074677273 zpe= -739.836642

N	0.676460	-0.166491	-3.824447
C	-0.608410	-0.916692	-3.914748
C	-1.438797	-0.400519	-2.735870
N	-2.013953	-1.288313	-1.891959
C	-1.527876	-2.629736	-1.637694
C	-0.070237	-2.622551	-1.155731
N	0.261930	-3.545618	-0.256929
C	1.615538	-3.680233	0.241842
C	1.620551	-4.786349	1.275072
O	2.834237	-4.966091	1.780483
H	2.801750	-5.676624	2.438083
H	-1.110981	-0.628948	-4.839349
H	-0.381582	-1.978960	-3.916930
O	-1.550387	0.802221	-2.638417
H	1.134586	-0.532140	-2.972673
H	-2.528308	-0.837236	-1.144076
H	-1.582845	-3.245369	-2.540184
H	-2.186976	-3.094698	-0.905440
O	0.738404	-1.818839	-1.619653
H	-0.422837	-4.179364	0.142548
H	2.313156	-3.929259	-0.561802
H	1.959871	-2.749634	0.699899
O	0.642131	-5.407804	1.581960
H	0.424732	0.813316	-3.636191
C	1.560294	-0.281980	-5.016198
H	1.803780	-1.331640	-5.167733
H	2.467910	0.290159	-4.836265
H	1.032347	0.111929	-5.882451

SGG_19.00_NcD.xyz
28
0 eng= -740.074165488 zpe= -739.836263

N	-1.107121	1.485765	-0.660428
C	-0.931005	0.542914	-1.800628
C	-2.344626	0.263298	-2.328739
N	-2.706770	-1.023257	-2.562280
C	-2.128715	-2.144519	-1.857682
C	-2.043862	-1.875549	-0.349350
N	-1.359577	-2.787705	0.375092
C	-1.048406	-2.498293	1.751763
C	-0.192267	-1.235280	1.879784
O	0.081363	-0.844516	3.120103
H	-0.288574	-1.431283	3.790964
H	-0.382265	1.062489	-2.587846
H	-0.352310	-0.304390	-1.440374

```
O       -3.050012       1.222265        -2.544358
H       -1.864640       2.127714        -0.932308
H       -3.668111       -1.108991       -2.869111
H       -1.131047       -2.385110       -2.238334
H       -2.752794       -3.022105       -2.033713
O       -2.524222       -0.875466       0.154755
H       -0.886494       -3.548698       -0.088713
H       -1.970917       -2.341455       2.319527
H       -0.518461       -3.345666       2.190539
O       0.200297        -0.601483       0.938406
H       -1.454947       0.923085        0.129357
C       0.125028        2.232501        -0.275525
H       0.888330        1.507620        -0.004795
H       -0.107073       2.866543        0.577559
H       0.436713        2.839680        -1.123079

GGS_0.00_NcA.xyz
28
0 eng= -740.074907002 zpe= -739.837096
C       -0.764838       0.623173        -1.987669
H       -1.075660       1.280912        -2.798939
H       0.211393        0.196747        -2.200261
C       -1.915776       -0.348398       -1.690607
O       -2.960276       0.138080        -1.323290
N       -0.630381       1.433315        -0.740867
H       -1.550202       1.816048        -0.492197
H       0.066944        2.173398        -0.835440
N       -1.698596       -1.674052       -1.866906
H       -2.485328       -2.245937       -1.585585
C       -0.383565       -2.274469       -1.842358
H       0.161986        -2.058307       -2.765396
H       -0.503832       -3.358036       -1.798244
C       0.436153        -1.786926       -0.634649
O       -0.081849       -1.075734       0.221132
N       1.741426        -2.115181       -0.593679
C       2.545702        -1.460337       0.417267
H       2.071040        -1.545267       1.398379
H       3.525790        -1.932479       0.477846
C       2.723304        0.024508        0.137659
O       3.655282        0.538416        0.924777
H       3.752683        1.485981        0.750031
O       2.086914        0.662656        -0.666414
H       -0.320962       0.780650        0.002246
C       2.434990        -2.855529       -1.647046
H       3.248960        -3.423190       -1.198133
H       2.845781        -2.179437       -2.402814
H       1.765859        -3.568992       -2.122241

GGS_8.31_t-c_O1-ctggcgtt.xyz
28
```

0 eng= -740.068443374 zpe= -739.833932

N	-4.403006	0.779636	-1.508171
C	-3.961435	1.099923	-0.164176
C	-2.786087	0.244349	0.240068
N	-2.340703	-0.639488	-0.615810
C	-1.357884	-1.683022	-0.332146
C	-0.003589	-1.079139	0.024965
N	1.101732	-1.424120	-0.631972
C	1.098240	-2.201673	-1.859961
C	0.400524	-1.475213	-3.004007
O	0.780939	-1.966422	-4.174563
H	0.301009	-1.519289	-4.887992
H	-3.638303	2.138553	-0.053230
H	-4.731261	0.937462	0.596505
O	-2.281233	0.423924	1.396407
H	-4.331206	1.570847	-2.134424
H	-5.357358	0.445081	-1.533903
H	-2.799701	-0.593952	-1.527866
H	-1.343940	-2.354376	-1.182895
H	-1.686058	-2.245568	0.548261
O	0.014749	-0.295902	0.989524
H	2.130024	-2.405305	-2.142854
H	0.619285	-3.176493	-1.730328
O	-0.415139	-0.607517	-2.849007
H	-1.278097	0.076601	1.408083
C	2.383212	-0.810920	-0.254366
H	3.141411	-1.590499	-0.185097
H	2.272825	-0.318268	0.706334
H	2.674890	-0.077826	-1.009646

GGS_10.29_t-c_O2-ctgtcgtt.xyz
28
0 eng= -740.066634451 zpe= -739.833175

N	-3.679276	1.247483	-2.440771
C	-2.943778	1.350787	-1.190102
C	-2.532887	-0.009092	-0.666903
N	-2.860932	-1.065711	-1.413767
C	-2.839259	-2.438210	-0.923719
C	-1.435529	-2.920219	-0.593335
N	-0.949699	-4.062153	-1.032656
C	-1.753092	-5.042760	-1.750500
C	-2.844101	-5.622318	-0.856154
O	-3.357578	-6.717962	-1.395950
H	-4.063859	-7.061519	-0.827482
H	-2.025001	1.934876	-1.285108
H	-3.520736	1.818525	-0.387498
O	-1.907087	-0.111514	0.403513
H	-3.223302	1.745768	-3.194012
H	-4.622280	1.605929	-2.361827
H	-3.380659	-0.823935	-2.256800

H	-3.330886	-3.062839	-1.662441
H	-3.406645	-2.523011	0.009982
O	-0.724876	-2.186839	0.159800
H	-2.213259	-4.617053	-2.645095
H	-1.098741	-5.844685	-2.089651
O	-3.191959	-5.116510	0.172231
H	-1.237059	-1.236828	0.404230
C	0.370335	-4.524864	-0.569119
H	0.927372	-3.687429	-0.162978
H	0.904838	-4.948730	-1.417834
H	0.233032	-5.284496	0.203382

GGS_10.86_OtB.xyz
28
0 eng= -740.066672003 zpe= -739.832961

C	-3.917122	0.753584	0.291908
H	-4.699397	1.226143	-0.307787
H	-3.321328	1.567728	0.715092
C	-3.008787	0.036603	-0.681619
O	-2.474780	0.716347	-1.605999
N	-4.439858	-0.157697	1.293130
H	-5.450497	-0.206595	1.278774
H	-4.153836	0.091611	2.231223
N	-2.841049	-1.256609	-0.508662
H	-3.378092	-1.592500	0.296821
C	-1.837619	-2.144707	-1.098136
H	-2.067937	-3.148427	-0.753011
H	-0.840886	-1.883748	-0.718414
C	-1.732492	-2.078533	-2.612746
O	-1.901832	-0.993126	-3.199088
N	-1.378614	-3.173330	-3.279091
C	-0.945179	-2.969465	-4.654790
H	-0.952440	-3.917969	-5.190545
H	-1.613298	-2.278684	-5.167919
C	0.463272	-2.384806	-4.644914
O	0.911587	-2.210318	-5.881946
H	1.804078	-1.835273	-5.848939
O	1.066198	-2.126879	-3.642088
H	-2.127671	0.048119	-2.385362
C	-0.960496	-4.412019	-2.610107
H	-0.064432	-4.246589	-2.008291
H	-0.729223	-5.147152	-3.376942
H	-1.768239	-4.817298	-2.001821

GGS_11.27_t-c_O1-ctgtcgtt.xyz
28
0 eng= -740.06691987 zpe= -739.832805

N	-2.264252	-0.428066	-5.420103
C	-1.423436	0.420414	-4.594824
C	-0.478894	-0.402020	-3.749661

```
N       -0.552006       -1.711918       -3.850775
C       0.434357        -2.686003       -3.373298
C       0.653142        -2.618631       -1.865685
N       0.692483        -3.724387       -1.128038
C       0.672134        -5.066462       -1.685701
C       1.921428        -5.355495       -2.509571
O       2.048392        -6.657181       -2.731571
H       2.835560        -6.818074       -3.273808
H       -0.793229       1.102457        -5.173307
H       -1.994024       1.044856        -3.902507
O       0.327661        0.204830        -2.985861
H       -2.118583       -0.271555       -6.409296
H       -3.248763       -0.296612       -5.227053
H       -1.272050       -2.003012       -4.516639
H       1.408703        -2.482005       -3.828307
H       0.109245        -3.661854       -3.720332
O       0.823157        -1.493012       -1.357906
H       0.610554        -5.781503       -0.865986
H       -0.208353       -5.240702       -2.309307
O       2.656607        -4.503428       -2.921261
H       0.684152        -0.483227       -2.216768
C       1.068330        -3.622937       0.290389
H       0.950870        -2.596017       0.620127
H       2.109504        -3.929751       0.414426
H       0.418007        -4.274937       0.872262

GGS_11.49_c-t_N-tcggtgtt.xyz
28
0 eng= -740.07087554 zpe= -739.832718
C       -2.650550       -0.724455       -0.422408
H       -2.006913       -1.106523       0.367942
H       -3.588056       -1.283159       -0.447477
C       -2.042230       -0.736269       -1.832187
O       -2.346744       0.166563        -2.587538
N       -2.949866       0.712112        -0.154702
H       -3.689905       0.846362        0.533776
H       -3.208403       1.137466        -1.060845
N       -1.253138       -1.771749       -2.171173
H       -0.942698       -1.749886       -3.134388
C       -0.629591       -2.711875       -1.244715
H       -0.026356       -3.406307       -1.828952
H       -1.377352       -3.298955       -0.710689
C       0.204610        -1.997203       -0.175279
O       -0.118127       -2.085465       0.996138
N       1.241489        -1.215249       -0.577255
C       1.800432        -0.355442       0.438368
H       2.026488        -0.919423       1.346106
H       2.727591        0.096064        0.082740
C       0.844676        0.757000        0.831391
O       1.378421        1.538490        1.750593
```

H	0.764553	2.247017	1.991699
O	-0.263828	0.916882	0.367562
H	-2.077118	1.162062	0.168518
C	1.624937	-0.976736	-1.964411
H	2.696092	-0.780439	-2.002600
H	1.087524	-0.124282	-2.391972
H	1.443387	-1.864106	-2.568461

GGS_12.14_OtB.xyz
28
0 eng= -740.066625229 zpe= -739.832472
N	-0.732455	0.132122	-4.579627
C	-1.314166	1.213038	-3.805292
C	-1.069663	1.019144	-2.326754
N	-0.384755	-0.040408	-1.958598
C	-0.232736	-0.588903	-0.609367
C	0.357533	0.411769	0.376907
N	1.268068	0.021631	1.261327
C	1.905399	1.079559	2.032471
C	2.921237	1.798400	1.151503
O	3.547147	2.746761	1.838448
H	4.184395	3.192581	1.261210
H	-0.901268	2.193263	-4.058082
H	-2.398391	1.297059	-3.924554
O	-1.527767	1.886713	-1.524761
H	-0.028422	0.459866	-5.228399
H	-1.425600	-0.382906	-5.107061
H	-0.091876	-0.600521	-2.763623
H	0.376079	-1.481901	-0.706445
H	-1.215022	-0.883896	-0.222304
O	-0.075376	1.580244	0.370480
H	1.164698	1.801171	2.376548
H	2.404359	0.655586	2.903114
O	3.115070	1.530129	0.000225
H	-0.991697	1.821385	-0.585815
C	1.930077	-1.287645	1.229753
H	2.457882	-1.419931	2.171451
H	2.651174	-1.336241	0.410515
H	1.200120	-2.091150	1.153621

GGS_12.86_c-c_N-gcgtcgtt.xyz
28
0 eng= -740.070094197 zpe= -739.832197
N	-0.624076	-4.638792	-0.441872
C	-1.565287	-3.476193	-0.483142
C	-2.891251	-4.115427	-0.921827
N	-3.566089	-3.564358	-1.953548
C	-2.950037	-2.734466	-2.964522
C	-1.689393	-3.394449	-3.561137
N	-1.097789	-2.768250	-4.599660

C	-1.290305	-1.350766	-4.817857
C	-0.621840	-0.536443	-3.712087
O	-0.470515	0.730011	-4.065978
H	-0.073683	1.229749	-3.336528
H	-1.688512	-3.096360	0.530997
H	-1.142214	-2.709136	-1.129248
O	-3.256368	-5.086655	-0.296720
H	-1.098779	-5.407068	0.053197
H	0.265070	-4.424889	0.011053
H	-4.393065	-4.088783	-2.211842
H	-2.690358	-1.756964	-2.551800
H	-3.684879	-2.566200	-3.752844
O	-1.267372	-4.448936	-3.098150
H	-0.868078	-1.067412	-5.781913
H	-2.347220	-1.074594	-4.851038
O	-0.306128	-0.996349	-2.645001
H	-0.469339	-4.931180	-1.422777
C	0.146580	-3.336845	-5.122209
H	1.004408	-2.997099	-4.533401
H	0.265568	-3.022270	-6.157955
H	0.088315	-4.420654	-5.077298

GGS_15.46_OtB.xyz
28
0 eng= -740.066853149 zpe= -739.831207

C	0.173236	2.267245	-1.240605
H	1.223606	2.268099	-1.545028
H	0.086844	2.986443	-0.421205
C	-0.101078	0.913488	-0.630669
O	0.690517	0.569122	0.321874
N	-0.744937	2.533053	-2.329972
H	-0.272347	2.660691	-3.215372
H	-1.320716	3.347555	-2.159760
N	-1.070084	0.206344	-1.127734
H	-1.560862	0.707243	-1.875485
C	-1.505249	-1.091961	-0.649775
H	-0.691553	-1.813253	-0.770460
H	-2.343501	-1.422323	-1.262921
C	-1.890209	-0.982829	0.835421
O	-1.968341	0.107719	1.359640
N	-2.009347	-2.149802	1.519968
C	-1.747048	-2.027987	2.937936
H	-2.377298	-1.260066	3.389997
H	-1.941756	-2.972128	3.446670
C	-0.293153	-1.624243	3.154035
O	0.049760	-1.675315	4.423880
H	0.969102	-1.386514	4.530497
O	0.463104	-1.294214	2.265744
H	0.456586	-0.199493	0.899131
C	-1.762384	-3.453509	0.910225

H	-0.692051	-3.683797	0.861924
H	-2.192372	-3.493584	-0.089082
H	-2.262726	-4.216075	1.505521

GGS_17.80_NcD.xyz
28
0 eng= -740.067665921 zpe= -739.830317

C	-0.083231	-0.932573	-3.059761
H	-0.312276	-1.484999	-3.970658
H	0.481131	-1.556298	-2.373167
C	-1.382882	-0.305901	-2.538607
O	-1.938919	0.488422	-3.260821
N	0.783992	0.230783	-3.414271
H	1.684618	-0.065087	-3.797733
H	0.962038	0.749424	-2.536579
N	-1.833222	-0.687931	-1.317533
H	-2.659035	-0.181116	-1.023540
C	-0.978640	-1.239202	-0.290011
H	-0.697101	-2.269507	-0.525784
H	-1.545027	-1.277502	0.641959
C	0.290328	-0.392163	-0.098156
O	0.429354	0.667272	-0.700103
N	1.260940	-0.886636	0.698787
C	2.532420	-0.199486	0.644328
H	2.395488	0.863160	0.866256
H	3.204650	-0.624272	1.392545
C	3.172879	-0.305635	-0.742580
O	4.342900	0.305100	-0.892011
H	4.660726	0.717784	-0.079349
O	2.655927	-0.868342	-1.670239
H	0.279501	0.840978	-4.067226
C	1.217522	-2.215392	1.308255
H	1.811026	-2.202781	2.221449
H	1.617744	-2.975042	0.630145
H	0.199282	-2.474429	1.589027

SGS_0.00_NcA.xyz
31
0 eng= -779.378078582 zpe= -779.111932

N	-1.170788	1.348813	2.066525
C	-0.941454	0.623423	0.785741
C	-2.341653	0.315861	0.237919
N	-2.616907	-0.948905	-0.161089
C	-1.929033	-2.108674	0.360108
C	-1.837947	-2.065981	1.895529
N	-1.124968	-3.036496	2.501280
C	-0.867722	-2.861236	3.914621
C	0.076093	-1.699763	4.183397
O	0.473703	-1.703861	5.447533
H	1.064214	-0.953907	5.609784

H	-0.455563	1.308309	0.089125
H	-0.289931	-0.221118	0.998354
O	-3.116373	1.243202	0.166191
H	-1.982111	1.962123	1.906651
H	-3.579007	-1.066769	-0.454164
H	-0.922918	-2.191674	-0.061916
H	-2.473519	-2.998538	0.041344
O	-2.356920	-1.150209	2.523413
H	-1.796925	-2.648188	4.449530
H	-0.441149	-3.772535	4.333046
O	0.404050	-0.867358	3.372750
H	-1.463692	0.629284	2.746405
C	0.003250	2.120148	2.559789
H	-0.259795	2.586638	3.507032
H	0.248947	2.882505	1.822997
H	0.829525	1.426849	2.696244
C	-0.358328	-4.055758	1.787058
H	-0.302752	-4.950344	2.405853
H	0.656263	-3.709376	1.568339
H	-0.855024	-4.332786	0.860080

SGS_6.56_NcA.xyz
31
0 eng= -779.375836221 zpe= -779.109433

N	1.012409	-1.072753	-3.588336
C	-0.084948	-1.686478	-2.771410
C	-1.220513	-0.663214	-2.642674
N	-1.557191	-0.248638	-1.393791
C	-0.725967	-0.380191	-0.221694
C	0.693869	0.153534	-0.464179
N	1.614097	-0.082488	0.493826
C	2.990823	0.206966	0.156926
C	3.544811	-0.766464	-0.871882
O	4.854686	-0.624441	-0.993353
H	5.197376	-1.242605	-1.655419
H	-0.464815	-2.554724	-3.310222
H	0.363597	-2.004918	-1.832494
O	-1.781498	-0.285213	-3.645006
H	1.878696	-1.569732	-3.357284
H	-2.271022	0.468815	-1.394251
H	-0.674081	-1.421842	0.108513
H	-1.193567	0.178940	0.590811
O	0.977035	0.725202	-1.511018
H	3.078218	1.208551	-0.271674
H	3.611968	0.170337	1.051553
O	2.885890	-1.550081	-1.514203
H	1.151876	-0.123734	-3.204559
C	0.781721	-1.046986	-5.065131
H	-0.136135	-0.499148	-5.259612
H	0.690591	-2.073805	-5.414394

H	1.635499	-0.562575	-5.534510
C	1.354727	-0.880721	1.691493
H	1.500921	-1.948035	1.499073
H	0.344068	-0.711291	2.056213
H	2.039423	-0.565005	2.477184

SGS_10.82_c-c_N-gcgtcgtt.xyz
31
0 eng= -779.373907626 zpe= -779.107811

C	0.137262	-3.585950	-3.728011
H	0.786043	-4.074277	-4.456654
H	0.400250	-2.533192	-3.637838
C	0.142302	-4.418228	-2.440789
O	-0.297389	-5.544360	-2.513687
N	-1.267315	-3.674599	-4.223267
H	-1.840371	-3.202680	-3.503398
N	0.637954	-3.858042	-1.313172
H	0.533847	-4.449043	-0.497547
C	0.742142	-2.431519	-1.104283
H	1.530882	-2.009719	-1.731858
H	1.035292	-2.263514	-0.067009
C	-0.590636	-1.708545	-1.387943
O	-1.549794	-2.332857	-1.825457
N	-0.646176	-0.385255	-1.120652
C	0.559503	0.413344	-1.097090
H	0.333535	1.409138	-0.715364
H	1.322392	-0.003631	-0.434860
C	1.157711	0.532789	-2.496769
O	2.044807	1.513086	-2.564255
H	2.426586	1.549048	-3.454110
O	0.870119	-0.199341	-3.409128
H	-1.524479	-4.670002	-4.200392
C	-1.485907	-3.072712	-5.566487
H	-0.867910	-3.599937	-6.290633
H	-1.199276	-2.023413	-5.519687
H	-2.537587	-3.166427	-5.828353
C	-1.882925	0.323309	-1.456090
H	-2.730844	-0.319260	-1.235863
H	-1.901412	0.592035	-2.517174
H	-1.943345	1.225014	-0.848619

SGS_11.35_c-t_N-tcggtgtt.xyz
31
0 eng= -779.373966042 zpe= -779.10761

C	-0.948047	-1.364694	-2.743453
H	-1.798682	-1.957235	-3.090210
H	-0.168372	-2.022671	-2.361922
C	-1.462341	-0.312391	-1.754457
O	-1.794179	0.770772	-2.195843
N	-0.403692	-0.599412	-3.897345

H	0.517672	-0.246078	-3.595702
N	-1.575043	-0.664350	-0.459003
H	-1.980686	0.055529	0.125318
C	-0.952391	-1.824119	0.170879
H	-1.322731	-2.755309	-0.258817
H	-1.225857	-1.820452	1.225789
C	0.568948	-1.835893	-0.019975
O	1.088826	-2.737779	-0.652569
N	1.289535	-0.786625	0.459117
C	2.651152	-0.704031	-0.011517
H	3.203301	0.048547	0.553014
H	3.164796	-1.660317	0.110672
C	2.720279	-0.345866	-1.485687
O	3.973165	-0.250711	-1.892282
H	4.006171	-0.016474	-2.830704
O	1.761324	-0.168034	-2.204244
H	-1.008313	0.229961	-4.000015
C	-0.288365	-1.380620	-5.158425
H	0.342393	-2.247870	-4.972539
H	0.154440	-0.749280	-5.925664
H	-1.283014	-1.699055	-5.464205
C	0.727031	0.378766	1.131751
H	-0.144004	0.095395	1.719783
H	0.450223	1.162837	0.419811
H	1.466874	0.772902	1.828132

SGS_13.01_c-t_N-tcgttgtt.xyz
31
0 eng= -779.372925643 zpe= -779.106975

N	-0.556304	1.751895	-1.033567
C	-0.566097	0.851021	-2.221916
C	-0.755944	-0.560352	-1.640696
N	-0.398872	-1.609567	-2.405075
C	0.555264	-1.523020	-3.487077
C	1.894764	-0.942335	-3.001184
N	2.855391	-0.751106	-3.937233
C	3.965492	0.082080	-3.534060
C	3.530292	1.529929	-3.364321
O	4.575842	2.312875	-3.128048
H	4.282424	3.230813	-3.036946
H	-1.437003	1.092922	-2.835178
H	0.351246	1.016801	-2.784648
O	-1.294331	-0.649396	-0.554988
H	-0.895537	2.681596	-1.281172
H	-0.515417	-2.502175	-1.941675
H	0.164908	-0.901251	-4.299096
H	0.693478	-2.522364	-3.900455
O	2.038825	-0.622716	-1.834110
H	4.371166	-0.253632	-2.577667
H	4.763863	0.032257	-4.274883

O	2.391089	1.921836	-3.421329
H	-1.217009	1.314126	-0.367655
C	0.790713	1.845004	-0.380737
H	1.096900	0.837262	-0.107922
H	0.703597	2.482175	0.496639
H	1.486324	2.256684	-1.109002
C	2.625714	-0.912731	-5.370494
H	3.580501	-1.108215	-5.857110
H	2.179956	-0.013277	-5.808079
H	1.983365	-1.768786	-5.565316

SGS_16.13_c-t_N-gcggtgtt.xyz
31
0 eng= -779.372175984 zpe= -779.105788

N	-1.076473	0.446295	-2.536408
C	-1.221457	-0.965184	-2.076663
C	-2.646788	-1.080563	-1.528922
N	-2.802330	-1.404661	-0.220111
C	-1.757811	-1.328963	0.795965
C	-1.216388	0.101889	0.894871
N	-1.950446	1.025373	1.560970
C	-1.464007	2.387559	1.529710
C	-1.703255	3.063473	0.192563
O	-1.563582	4.373250	0.285979
H	-1.695388	4.783242	-0.582057
H	-1.142513	-1.615864	-2.948066
H	-0.418831	-1.161840	-1.373041
O	-3.562893	-0.856867	-2.287225
H	-1.288180	1.084731	-1.745931
H	-3.769264	-1.461586	0.075228
H	-0.917053	-1.974248	0.544281
H	-2.168096	-1.690938	1.736053
O	-0.171307	0.393245	0.325604
H	-1.941653	2.973420	2.314993
H	-0.384777	2.404639	1.702647
O	-1.969787	2.490280	-0.839497
H	-1.809055	0.621934	-3.233217
C	0.277907	0.765245	-3.066632
H	0.987309	0.639412	-2.250314
H	0.282333	1.795512	-3.416144
H	0.507294	0.086056	-3.885694
C	-3.330701	0.830259	1.998489
H	-3.509773	-0.207214	2.269718
H	-4.036384	1.128424	1.216805
H	-3.507818	1.432903	2.888834

SGS_16.81_t-t_N-gtgttgtt.xyz
31
0 eng= -779.371374277 zpe= -779.105528

C	-2.108395	0.705421	-2.602685

H	-2.364337	1.738997	-2.831605
H	-2.818308	0.019379	-3.064163
C	-1.905774	0.510593	-1.102162
O	-1.150877	1.262651	-0.527281
N	-0.742897	0.420015	-3.151353
H	-0.063254	1.017051	-2.654636
N	-2.389932	-0.630459	-0.542960
H	-2.842522	-1.287754	-1.161819
C	-1.489047	-1.213368	0.442228
H	-1.885607	-2.173962	0.772459
H	-1.408631	-0.549183	1.297551
C	-0.145927	-1.441180	-0.278733
O	-0.167116	-1.809321	-1.455573
N	1.000064	-1.213696	0.380248
C	2.232319	-1.423485	-0.351546
H	3.069008	-1.501252	0.341052
H	2.174967	-2.360305	-0.912557
C	2.528954	-0.333254	-1.369897
O	3.801350	-0.389511	-1.743830
H	3.975470	0.289954	-2.411038
O	1.733707	0.455768	-1.815413
H	-0.462658	-0.533104	-2.845331
C	-0.616731	0.579556	-4.623956
H	0.413881	0.374492	-4.905674
H	-0.883112	1.600693	-4.890106
H	-1.286425	-0.124412	-5.114193
C	1.045667	-0.520992	1.674349
H	0.487036	-1.071900	2.430577
H	0.654318	0.494229	1.578640
H	2.082001	-0.470480	2.000386

SGS_17.19_t-t_N-gtgttgtt.xyz
31
0 eng= -779.371438062 zpe= -779.105383

C	-2.989411	-0.431648	-2.649895
H	-3.493677	0.458876	-3.023032
H	-3.455042	-1.334833	-3.042993
C	-2.847228	-0.387077	-1.129586
O	-2.314882	0.584343	-0.642584
N	-1.561745	-0.395687	-3.104834
H	-1.108148	0.416282	-2.660459
N	-3.114932	-1.524749	-0.431886
H	-3.364292	-2.337655	-0.977419
C	-2.162599	-1.769908	0.641037
H	-2.394030	-2.716966	1.130048
H	-2.232470	-0.967424	1.369918
C	-0.769615	-1.849055	-0.017743
O	-0.682073	-2.285592	-1.167942
N	0.301644	-1.419745	0.665233
C	1.575187	-1.446530	-0.025171

H	2.387998	-1.318641	0.687846
H	1.711387	-2.413464	-0.517705
C	1.699212	-0.390874	-1.113871
O	2.973383	-0.238606	-1.457054
H	3.043627	0.416160	-2.166467
O	0.788653	0.204990	-1.630969
H	-1.067635	-1.199081	-2.667515
C	-1.375813	-0.375255	-4.579233
H	-1.804848	-1.282323	-5.000382
H	-0.309701	-0.332603	-4.791653
H	-1.875721	0.502133	-4.985124
C	0.198206	-0.717701	1.949004
H	1.193793	-0.636445	2.379357
H	-0.415641	-1.283889	2.647987
H	-0.215431	0.284491	1.813697

SGS_18.39_NcD.xyz
31
0 eng= -779.37139563 zpe= -779.104926

C	-1.039894	-0.797176	-3.001114
H	-0.639484	-0.068374	-2.300736
H	-1.619386	-0.295657	-3.777850
C	-1.904585	-1.927359	-2.427579
O	-2.197025	-2.830027	-3.179266
N	0.115166	-1.477325	-3.651948
H	-0.269098	-2.307807	-4.122983
N	-2.317589	-1.838731	-1.139433
H	-2.836638	-2.654359	-0.838088
C	-1.610045	-1.085953	-0.128880
H	-2.038685	-1.333318	0.843281
H	-1.752218	-0.009671	-0.266822
C	-0.105737	-1.401551	-0.142505
O	0.352156	-2.222042	-0.928244
N	0.688341	-0.698974	0.696030
C	2.107662	-0.814383	0.448976
H	2.656163	-0.278562	1.226425
H	2.410240	-1.865237	0.473037
C	2.476386	-0.250218	-0.925449
O	3.759329	-0.333705	-1.266984
H	4.306955	-0.719934	-0.572653
O	1.671583	0.215996	-1.685440
H	0.710605	-1.818510	-2.882184
C	0.889092	-0.623294	-4.597726
H	1.716220	-1.208108	-4.994906
H	1.265952	0.234991	-4.046579
H	0.225586	-0.315432	-5.403518
C	0.222598	0.432946	1.495555
H	-0.787360	0.256167	1.858055
H	0.248789	1.362061	0.918030
H	0.866529	0.535154	2.368128

SGS_18.62_c-c_N-gcggcgtt.xyz
31
0 eng= -779.371786044 zpe= -779.104841

N	-1.238047	-2.402403	3.992855
C	-2.533182	-1.768604	3.612760
C	-2.904356	-2.424914	2.278157
N	-3.213376	-1.645544	1.215873
C	-2.674357	-0.315177	0.997532
C	-1.146118	-0.392681	0.864723
N	-0.532363	0.143111	-0.203473
C	-1.239797	0.579983	-1.393308
C	-1.937178	-0.569169	-2.111525
O	-2.212294	-0.245366	-3.367909
H	-2.691138	-0.972928	-3.792709
H	-3.283897	-2.047009	4.353562
H	-2.385758	-0.693105	3.597444
O	-2.913650	-3.637419	2.261881
H	-1.350666	-3.411594	3.829114
H	-3.347671	-2.185111	0.364784
H	-2.896584	0.337170	1.844181
H	-3.179894	0.106999	0.135612
O	-0.527745	-0.941221	1.779549
H	-1.988474	1.345722	-1.172948
H	-0.526412	1.042079	-2.074641
O	-2.232340	-1.609807	-1.589303
H	-0.562458	-2.050913	3.291907
C	-0.783354	-2.120495	5.380685
H	0.169975	-2.617804	5.545793
H	-1.529804	-2.496510	6.077571
H	-0.668684	-1.044419	5.494708
C	0.919121	-0.015008	-0.338728
H	1.345823	-0.212884	0.639314
H	1.341964	0.903352	-0.744779
H	1.145352	-0.850259	-1.006801

GSS_0.00_NcA.xyz
31
0 eng= -779.37506577 zpe= -779.109064

N	-1.177449	-0.612726	0.739942
C	-1.702071	0.536450	-0.057233
C	-3.222190	0.311800	-0.075649
N	-3.868024	0.290403	-1.262455
C	-3.142644	0.051665	-2.493345
C	-2.275982	-1.216241	-2.400172
N	-1.426695	-1.466224	-3.415856
C	-0.464714	-2.529282	-3.212755
C	0.582325	-2.166955	-2.170162
O	1.570950	-3.047527	-2.177845
H	2.226895	-2.816740	-1.503683

H	-1.500879	1.457605	0.488894
H	-1.171372	0.534631	-1.004528
O	-3.744944	0.162411	1.010499
H	-1.698287	-0.659704	1.624109
H	-0.170865	-0.547205	0.898409
H	-2.515155	0.907666	-2.757926
H	-3.871230	-0.058568	-3.298746
O	-2.324987	-1.930992	-1.403613
H	-0.964720	-3.435256	-2.860429
H	0.035049	-2.765540	-4.151827
O	0.518698	-1.225796	-1.416074
H	-1.369159	-1.466991	0.187691
C	-5.295545	-0.040730	-1.241540
H	-5.784185	0.443613	-2.085944
H	-5.725711	0.325397	-0.313355
H	-5.434577	-1.124021	-1.297201
C	-1.233948	-0.573795	-4.557926
H	-0.927675	-1.166916	-5.418498
H	-0.465543	0.177307	-4.351264
H	-2.165539	-0.078063	-4.820652

GSS_12.54_c-c_N-gcgtcgtt.xyz
31
0 eng= -779.370403531 zpe= -779.104288

N	-1.303968	-3.781392	0.453889
C	-1.425453	-2.614699	-0.476176
C	-2.890283	-2.183411	-0.296186
N	-3.172973	-0.887900	-0.052289
C	-2.160644	0.006025	0.471408
C	-1.532359	-0.538765	1.770890
N	-0.703249	0.280660	2.451129
C	-0.040081	1.382559	1.788967
C	1.003164	0.872990	0.797013
O	1.856300	1.826742	0.457226
H	2.489214	1.484037	-0.191804
H	-0.647226	-1.895828	-0.229672
H	-1.300535	-2.977282	-1.496487
O	-3.712622	-3.074860	-0.389340
H	-0.442851	-4.314118	0.328582
H	-2.125037	-4.383735	0.297029
H	-2.638012	0.966540	0.670519
H	-1.375214	0.185107	-0.266428
O	-1.795777	-1.674360	2.152962
H	-0.740058	2.021984	1.245209
H	0.444794	2.018039	2.529899
O	1.017245	-0.250051	0.363104
H	-1.360071	-3.403732	1.415235
C	-4.573634	-0.550852	0.218938
H	-4.757624	0.476013	-0.093772
H	-4.793332	-0.664520	1.284431

H	-5.211596	-1.223979	-0.347135
C	-0.031441	-0.260156	3.635084
H	0.257768	0.568360	4.279738
H	0.856618	-0.832286	3.348760
H	-0.720012	-0.910164	4.167549

GSS_13.88_c-t_O1-tcgttgtt.xyz
31
0 eng= -779.367698625 zpe= -779.103779

N	-0.873412	0.509079	-2.679330
C	-1.758236	-0.581689	-2.294029
C	-2.817628	0.026199	-1.382256
N	-3.784079	-0.653246	-0.834815
C	-3.741499	-2.109033	-0.865446
C	-2.622352	-2.595662	0.077402
N	-2.423123	-3.932439	0.151110
C	-1.151899	-4.328246	0.721287
C	-0.016832	-3.960373	-0.226002
O	1.150619	-4.386335	0.237790
H	1.852780	-4.137549	-0.380746
H	-2.259903	-1.025049	-3.158320
H	-1.235953	-1.382291	-1.763334
O	-2.727585	1.293725	-1.197412
H	0.070523	0.379720	-2.335262
H	-0.833707	0.654769	-3.680335
H	-3.553013	-2.465250	-1.880908
H	-4.718763	-2.482845	-0.560464
O	-1.947428	-1.780055	0.673251
H	-0.979519	-3.818148	1.669956
H	-1.139309	-5.402991	0.904826
O	-0.162666	-3.358662	-1.258722
H	-1.914503	1.552931	-1.736727
C	-4.684271	-0.024560	0.143727
H	-5.671461	-0.471150	0.039916
H	-4.741960	1.042725	-0.048646
H	-4.287671	-0.202015	1.145598
C	-3.061761	-4.883253	-0.754903
H	-3.061626	-5.865470	-0.284018
H	-2.527300	-4.944010	-1.708663
H	-4.100994	-4.612901	-0.932842

GSS_14.73_c-t_N-tcggtgtt.xyz
31
0 eng= -779.369748915 zpe= -779.103453

N	-1.121870	-0.738003	-3.433376
C	-2.421290	-1.016286	-2.754671
C	-3.113566	0.353121	-2.666533
N	-4.053223	0.557340	-1.729349
C	-4.254844	-0.350338	-0.597487
C	-2.987765	-0.582988	0.237725

```
N       -2.361613       0.487796        0.793088
C       -1.056028       0.223758        1.348389
C       -0.024744       -0.049953       0.268642
O       1.161374        -0.291854       0.793249
H       1.812306        -0.460374       0.096886
H       -3.025480       -1.653051       -3.404101
H       -2.227728       -1.523890       -1.813227
O       -2.817287       1.176714        -3.518680
H       -1.292526       0.066714        -4.062707
H       -0.761546       -1.536684       -3.954502
H       -4.580224       -1.338869       -0.926881
H       -5.048773       0.062385        0.024817
O       -2.544535       -1.712485       0.348649
H       -0.716820       1.074805        1.940709
H       -1.079921       -0.652193       2.000165
O       -0.249882       -0.046407       -0.922158
H       -0.440275       -0.450392       -2.712305
C       -4.858447       1.776647        -1.883618
H       -5.275578       1.809297        -2.889739
H       -4.249168       2.669752        -1.739132
H       -5.668891       1.756071        -1.158370
C       -2.763861       1.876837        0.612593
H       -2.439763       2.450665        1.480308
H       -3.847932       1.955363        0.560411
H       -2.322993       2.310337        -0.291067

GSS_17.46_c-c_O1-tcgtcgtt.xyz
31
0 eng= -779.366013909 zpe= -779.102414
N       0.223224        -1.745516       -5.128873
C       -0.305963       -2.046665       -3.806309
C       -1.809866       -2.228471       -3.974939
N       -2.621674       -2.506377       -2.996682
C       -2.141545       -2.428984       -1.623491
C       -1.960860       -0.941475       -1.238490
N       -1.572931       -0.682210       0.034401
C       -0.944155       -1.689492       0.857812
C       0.460610        -2.028879       0.366170
O       1.194460        -2.574099       1.325920
H       2.067993        -2.805599       0.976111
H       0.117002        -2.964580       -3.390327
H       -0.122500       -1.253162       -3.079706
O       -2.251362       -2.115913       -5.176137
H       0.617705        -0.814389       -5.187226
H       0.920711        -2.410536       -5.439072
H       -1.200180       -2.970425       -1.525723
H       -2.880951       -2.910094       -0.982017
O       -2.189788       -0.086478       -2.068062
H       -0.892187       -1.336993       1.887748
H       -1.519998       -2.619503       0.883433
```

```
O       0.829942        -1.855556       -0.766074
H       -1.422470       -1.919204       -5.715501
C       -4.077459       -2.520757       -3.202485
H       -4.295911       -2.713414       -4.248462
H       -4.477259       -1.547276       -2.910935
H       -4.505335       -3.308546       -2.584836
C       -1.276200       0.711257        0.376144
H       -0.240815       0.958915        0.122588
H       -1.437373       0.852602        1.444070
H       -1.942265       1.362205        -0.182984

GSS_18.76_NcD.xyz
31
0 eng= -779.367824401 zpe= -779.101918
N       -0.934632       1.232077        1.181734
C       -1.605497       1.525580        -0.121155
C       -3.103090       1.527459        0.218541
N       -3.939998       0.746277        -0.502714
C       -3.430642       -0.377916       -1.260675
C       -2.573625       -1.305035       -0.382960
N       -1.916230       -2.316039       -0.990586
C       -0.924694       -2.996650       -0.187729
C       0.228004        -2.064819       0.196328
O       1.182658        -2.600253       0.949039
H       1.033445        -3.534802       1.137906
H       -1.324947       2.530664        -0.434741
H       -1.244234       0.793047        -0.835952
O       -3.436595       2.248771        1.136479
H       -1.235580       0.284889        1.466174
H       -1.260588       1.909046        1.881258
H       -2.844989       -0.045274       -2.122479
H       -4.282591       -0.931081       -1.660599
O       -2.451089       -1.088035       0.817926
H       -1.377736       -3.365229       0.737371
H       -0.533736       -3.850491       -0.744863
O       0.278147        -0.906555       -0.120826
H       0.084470        1.231986        1.100494
C       -5.339248       0.691695        -0.070201
H       -5.968759       0.477408        -0.932820
H       -5.613251       1.655938        0.349061
H       -5.471772       -0.079950       0.693447
C       -1.885296       -2.514054       -2.438966
H       -2.834954       -2.227256       -2.884372
H       -1.737109       -3.573123       -2.646159
H       -1.074959       -1.941190       -2.899804

GSS_21.42_c-c_N-gcgtcgtt.xyz
31
0 eng= -779.366734537 zpe= -779.100904
N       -0.788199       -4.621458       -0.143300
```

C	-2.232428	-4.664619	-0.529008
C	-2.922583	-3.943923	0.638081
N	-3.793536	-2.943451	0.399487
C	-3.757849	-2.170303	-0.828209
C	-2.408863	-1.438645	-0.982940
N	-2.381542	-0.247390	-1.601487
C	-3.574063	0.503102	-1.941438
C	-4.237641	1.104142	-0.706762
O	-5.089843	2.065312	-1.047837
H	-5.521676	2.410313	-0.251958
H	-2.330514	-4.225082	-1.515317
H	-2.558993	-5.704402	-0.533337
O	-2.620464	-4.353993	1.743784
H	-0.192272	-5.225612	-0.709402
H	-0.731040	-4.891709	0.849368
H	-4.601481	-1.488618	-0.794782
H	-3.899655	-2.806950	-1.706487
O	-1.389206	-2.006341	-0.587759
H	-3.301865	1.305398	-2.627277
H	-4.311822	-0.107986	-2.467367
O	-4.035312	0.723620	0.411430
H	-0.500124	-3.630610	-0.232770
C	-4.343232	-2.265822	1.582467
H	-4.521388	-3.006687	2.357029
H	-5.279391	-1.784592	1.305278
H	-3.645056	-1.511114	1.952014
C	-1.121865	0.497786	-1.676220
H	-0.301915	-0.163197	-1.415540
H	-1.147385	1.334264	-0.973076
H	-0.984605	0.875589	-2.689599

GSS_22.41_c-t_O1-tcgttgtt.xyz
31
0 eng= -779.364244242 zpe= -779.100529

N	-2.660045	-2.285890	-3.745322
C	-2.178560	-2.179529	-2.376319
C	-2.425036	-0.739591	-1.935473
N	-2.128339	-0.277613	-0.758201
C	-1.324940	-1.057787	0.175104
C	0.153713	-0.921456	-0.249465
N	1.100586	-1.086834	0.701168
C	2.392477	-0.518125	0.369355
C	2.285855	1.003806	0.363780
O	3.460036	1.565421	0.099804
H	3.359133	2.528568	0.098162
H	-2.711579	-2.852081	-1.699813
H	-1.108035	-2.373766	-2.291324
O	-2.984161	0.017501	-2.811746
H	-1.920846	-2.492332	-4.406126
H	-3.397791	-2.969708	-3.859025

H	-1.629911	-2.106336	0.179462
H	-1.503769	-0.652032	1.169223
O	0.402279	-0.663163	-1.411972
H	3.142001	-0.830908	1.096601
H	2.716511	-0.844015	-0.619054
O	1.267637	1.606208	0.570825
H	-3.114214	-0.587437	-3.605062
C	-2.237901	1.157040	-0.439351
H	-2.765611	1.671160	-1.236068
H	-1.226139	1.557300	-0.332072
H	-2.783384	1.262288	0.498060
C	0.786142	-1.228940	2.120833
H	-0.044526	-1.918583	2.263101
H	0.555915	-0.261434	2.578535
H	1.650496	-1.662725	2.621430

GSS_22.54_NcA.xyz
31
0 eng= -779.366720433 zpe= -779.100478

N	0.888087	-0.791008	-2.962670
C	-0.470987	-1.262509	-2.554446
C	-0.722730	-0.494299	-1.249015
N	-1.130697	-1.157168	-0.148331
C	-0.878736	-2.576105	0.031215
C	0.636449	-2.848607	0.067696
N	1.105819	-3.807500	0.881503
C	2.537658	-3.794339	1.116733
C	2.888869	-2.623709	2.028845
O	4.195041	-2.593399	2.279409
H	4.387808	-1.848808	2.867833
H	-1.193431	-0.928471	-3.298832
H	-0.447753	-2.344982	-2.495343
O	-0.526456	0.704994	-1.312566
H	1.146095	-1.052023	-3.914521
H	1.548176	-1.198225	-2.277087
H	-1.333739	-3.176876	-0.760665
H	-1.363642	-2.867636	0.959164
O	1.378963	-2.185746	-0.660525
H	2.850564	-4.727525	1.584854
H	3.083015	-3.682682	0.180379
O	2.086977	-1.843236	2.457561
H	0.898602	0.234080	-2.857380
C	-1.230928	-0.380145	1.094836
H	-1.468305	0.650570	0.848700
H	-0.280291	-0.415938	1.636699
H	-2.025301	-0.802906	1.708758
C	0.275492	-4.499381	1.870176
H	0.817823	-5.379139	2.211647
H	-0.652557	-4.846741	1.420518
H	0.067649	-3.854698	2.728620

GSS_22.97_t-t_O1-ttgttgtt.xyz
31
0 eng= -779.364326958 zpe= -779.100315

N	-4.455843	-0.078195	-3.771189
C	-3.048513	-0.390182	-3.576275
C	-2.968281	-1.346983	-2.394681
N	-1.864209	-1.846428	-1.926281
C	-1.889613	-2.455164	-0.599640
C	-1.945433	-1.290956	0.415152
N	-1.724652	-1.600079	1.712681
C	-1.369823	-0.475578	2.554617
C	0.020608	0.024679	2.180061
O	0.392534	1.034571	2.961475
H	1.276043	1.329658	2.697721
H	-2.600169	-0.858324	-4.454904
H	-2.455469	0.492559	-3.324504
O	-4.092343	-1.654915	-1.849521
H	-4.665708	0.901754	-3.625523
H	-4.802007	-0.351361	-4.682465
H	-0.977741	-3.038815	-0.476985
H	-2.753605	-3.112361	-0.499807
O	-2.158829	-0.163180	0.013532
H	-2.077700	0.343001	2.422204
H	-1.382047	-0.772148	3.603793
O	0.686482	-0.436047	1.293662
H	-4.771704	-1.149229	-2.389602
C	-0.543541	-1.412278	-2.396758
H	0.117714	-2.277224	-2.405409
H	-0.158499	-0.655218	-1.708896
H	-0.603603	-1.011198	-3.404886
C	-1.256222	-2.916844	2.135326
H	-1.469685	-3.036042	3.196630
H	-0.179329	-3.027103	1.971704
H	-1.795222	-3.703478	1.609525

GSG_0.00_NcA.xyz
28
0 eng= -740.078294491 zpe= -739.840588

C	-0.654605	0.543114	-2.078866
H	-0.907019	1.147426	-2.949853
H	0.327572	0.097277	-2.206117
C	-1.844446	-0.383841	-1.783150
O	-2.883511	0.171646	-1.489018
N	-0.583645	1.440795	-0.887306
H	-1.521240	1.824999	-0.716585
H	0.111264	2.180723	-0.999827
N	-1.673782	-1.722310	-1.868714
C	-0.351047	-2.306520	-1.781050
H	0.220442	-2.157566	-2.702716

H	-0.463731	-3.384601	-1.650379
C	0.437903	-1.746700	-0.589247
O	-0.091820	-1.051616	0.263850
N	1.756210	-2.023223	-0.578217
H	2.164919	-2.561934	-1.327230
C	2.620504	-1.427709	0.412691
H	2.227913	-1.601484	1.418391
H	3.611629	-1.877244	0.362475
C	2.746781	0.077935	0.233815
O	3.670631	0.561847	1.047899
H	3.741389	1.521546	0.939357
O	2.087384	0.748741	-0.522789
H	-0.304195	0.856913	-0.081304
C	-2.816754	-2.565985	-1.505320
H	-2.750527	-3.504880	-2.053130
H	-3.732838	-2.049230	-1.778171
H	-2.821704	-2.757221	-0.428791

GSG_8.70_NcB.xyz
28
0 eng= -740.074542577 zpe= -739.837275

C	-2.265932	-0.107488	0.973748
H	-1.356410	-0.543400	1.379354
H	-3.138526	-0.619452	1.383975
C	-2.386120	-0.101803	-0.562926
O	-2.913914	0.873447	-1.071169
N	-2.312090	1.329044	1.368378
H	-2.854538	1.815604	0.631182
H	-1.344620	1.690254	1.361579
N	-1.949774	-1.185534	-1.227909
C	-1.137819	-2.206172	-0.572041
H	-0.931687	-2.991498	-1.301056
H	-1.655947	-2.672813	0.267699
C	0.186855	-1.656746	-0.033660
O	0.613719	-1.997934	1.046408
N	0.825212	-0.746172	-0.818972
H	0.375087	-0.391621	-1.648552
C	1.962112	-0.046945	-0.282863
H	2.668336	-0.749998	0.162613
H	2.484060	0.493515	-1.074185
C	1.561091	0.949050	0.791549
O	2.619085	1.462162	1.385312
H	2.351230	2.100618	2.062462
O	0.419373	1.250723	1.064179
H	-2.729735	1.481692	2.285939
C	-2.188798	-1.296234	-2.669521
H	-2.720606	-2.225481	-2.878023
H	-1.242002	-1.299583	-3.215981
H	-2.788771	-0.449842	-2.992737

GSG_13.78_NtA.xyz
28
0 eng= -740.07343606 zpe= -739.835339

N	-0.909732	-0.043513	-3.782393
C	-2.194538	-0.010146	-3.024352
C	-1.916253	-0.867789	-1.781471
N	-2.960586	-1.412468	-1.147701
C	-2.665115	-2.567284	-0.286134
C	-2.456664	-3.717252	-1.269979
N	-1.160770	-3.981575	-1.600930
C	-0.920810	-4.739633	-2.797762
C	-1.266768	-3.950549	-4.050444
O	-1.338561	-4.732222	-5.108932
H	-1.548842	-4.212355	-5.898063
H	-2.434105	1.021431	-2.763366
H	-2.972274	-0.434543	-3.655679
O	-0.742325	-1.040317	-1.474313
H	-0.148887	-0.004679	-3.084923
H	-0.823781	0.701257	-4.473461
H	-3.530021	-2.771238	0.340944
H	-1.793785	-2.352034	0.329435
O	-3.396242	-4.256164	-1.813953
H	-0.448917	-3.343578	-1.272924
H	0.130023	-5.029595	-2.862757
H	-1.519517	-5.651249	-2.798132
O	-1.430227	-2.746357	-4.094956
H	-0.840193	-0.973134	-4.229808
C	-4.345158	-1.255737	-1.597612
H	-4.606861	-2.018562	-2.335713
H	-4.504750	-0.255410	-1.997770
H	-4.997614	-1.368934	-0.733235

GSG_15.99_c-t_O1-tcgttgtt.xyz
28
0 eng= -740.069947073 zpe= -739.834498

N	-1.569494	2.555955	1.340824
C	-1.935605	1.276986	0.748036
C	-1.259143	1.230261	-0.617931
N	-1.345941	0.222999	-1.439792
C	-1.900949	-1.037300	-0.967444
C	-0.921995	-1.681390	0.031522
N	-1.342730	-2.840906	0.585514
C	-0.677544	-3.304341	1.781434
C	-0.897237	-2.330825	2.932590
O	-0.294835	-2.762472	4.032775
H	-0.443462	-2.131341	4.751727
H	-1.611124	0.424215	1.350660
H	-3.015501	1.190928	0.599789
O	-0.601711	2.282949	-0.944221
H	-2.369224	3.135274	1.564125

H   -1.008415   2.446013   2.177182
H   -2.063385   -1.686148   -1.828643
H   -2.867755   -0.876144   -0.484832
O   0.127292   -1.143442   0.304145
H   -2.290646   -3.146278   0.420249
H   -1.049302   -4.289462   2.062138
H   0.396861   -3.388066   1.608470
O   -1.528740   -1.310154   2.853868
H   -0.709264   2.884108   -0.138413
C   -0.563484   0.190713   -2.685202
H   -1.165377   -0.284483   -3.457892
H   0.349794   -0.380747   -2.507219
H   -0.313629   1.204191   -2.984596

GSG_17.40_c-t_N-gcggtgtt.xyz
28
0 eng= -740.071690217 zpe= -739.83396
N   -0.135849   -0.858943   -3.747149
C   -1.557731   -1.175471   -3.407475
C   -2.223943   0.196043   -3.231054
N   -2.856620   0.487311   -2.069126
C   -2.541946   -0.216255   -0.822799
C   -1.052524   -0.052965   -0.516479
N   -0.683407   1.122340   0.033685
C   0.711724   1.474522   0.143225
C   1.368416   1.652800   -1.215448
O   2.568293   2.193725   -1.084728
H   2.986769   2.283350   -1.953625
H   -2.017286   -1.648278   -4.274901
H   -1.554042   -1.842071   -2.552894
O   -2.141453   0.955056   -4.175034
H   0.376556   -1.675044   -4.083672
H   0.338565   -0.472639   -2.911963
H   -2.770764   -1.279015   -0.890069
H   -3.165938   0.206485   -0.035664
O   -0.237658   -0.899903   -0.851339
H   -1.383067   1.804712   0.281953
H   0.821366   2.394018   0.716693
H   1.265383   0.688180   0.664995
O   0.884048   1.328033   -2.272859
H   -0.131013   -0.121755   -4.465020
C   -3.460716   1.819012   -1.979492
H   -3.931704   2.058105   -2.930095
H   -2.704251   2.583411   -1.775226
H   -4.213860   1.811090   -1.193219

GSG_18.37_NcC.xyz
28
0 eng= -740.071261448 zpe= -739.833591
N   -1.654945   -1.359656   -3.807107

C	-2.850868	-1.262982	-2.915520
C	-2.381191	-0.330198	-1.790341
N	-2.562685	-0.685151	-0.499977
C	-2.657736	-2.079001	-0.101269
C	-1.379569	-2.848178	-0.465079
N	-0.938098	-3.730134	0.426878
C	0.226789	-4.556594	0.182475
C	0.487872	-5.369150	1.432956
O	1.549789	-6.150270	1.284857
H	1.689341	-6.655212	2.099836
H	-3.651264	-0.762686	-3.460627
H	-3.144424	-2.268605	-2.636287
O	-1.870920	0.710354	-2.159462
H	-1.285435	-0.406158	-3.931727
H	-1.854092	-1.780413	-4.715217
H	-3.502710	-2.578188	-0.581580
H	-2.848965	-2.110298	0.971049
O	-0.828962	-2.662104	-1.551320
H	-1.378529	-3.841560	1.334488
H	0.066541	-5.231298	-0.662431
H	1.101643	-3.943270	-0.046812
O	-0.192046	-5.300704	2.418398
H	-0.956829	-1.921545	-3.288083
C	-2.059223	0.246109	0.516321
H	-2.150495	1.260927	0.139533
H	-1.006742	0.044929	0.738067
H	-2.656744	0.134665	1.419934

GSG_18.92_NcD.xyz
28
0 eng= -740.070492462 zpe= -739.83338

N	0.141420	0.950025	-0.217877
C	-0.141241	0.514663	-1.619174
C	-0.649172	-0.927598	-1.477190
N	-0.069695	-1.905296	-2.212851
C	1.260855	-1.731920	-2.757785
C	2.250530	-1.244840	-1.691437
N	3.454400	-0.834643	-2.145014
C	4.364899	-0.171341	-1.245331
C	3.779907	1.140670	-0.713229
O	4.534144	1.800941	0.157050
H	5.379717	1.369367	0.330794
H	-0.956402	1.123868	-2.008965
H	0.761131	0.686864	-2.197371
O	-1.563224	-1.092516	-0.695560
H	-0.696154	0.786420	0.352984
H	0.447849	1.924489	-0.170999
H	1.265954	-1.038553	-3.604773
H	1.601735	-2.696547	-3.138950
O	1.949355	-1.187015	-0.510194

H   3.636882   -0.809766   -3.137119
H   4.581484   -0.816838   -0.388505
H   5.303250   0.031361   -1.764909
O   2.697943   1.558282   -1.025708
H   0.911294   0.360873   0.138391
C   -0.537274   -3.275729   -1.981484
H   -0.380753   -3.859775   -2.887182
H   -1.597718   -3.247479   -1.745988
H   0.001748   -3.726579   -1.143641

GSG_19.68_c-t_N-gcggtgtt.xyz
28
0 eng= -740.071694521 zpe= -739.833093
N   -0.247601   -0.178247   -2.626661
C   -1.449777   -1.015254   -2.321787
C   -2.648853   -0.094480   -2.590518
N   -3.531150   0.168263   -1.594449
C   -3.157244   0.046121   -0.182884
C   -1.966396   0.964172   0.089676
N   -2.247830   2.267700   0.277229
C   -1.195637   3.256173   0.253577
C   -0.530889   3.346208   -1.111371
O   0.275709   4.394710   -1.165346
H   0.705438   4.436030   -2.032336
H   -1.499063   -1.823539   -3.050321
H   -1.334662   -1.401555   -1.315148
O   -2.737062   0.351857   -3.715100
H   -0.095850   0.472617   -1.837120
H   -0.442227   0.386780   -3.464830
H   -2.880460   -0.975032   0.075221
H   -4.026066   0.315225   0.417311
O   -0.816946   0.547006   0.025262
H   -3.208379   2.574566   0.300490
H   -1.595257   4.232837   0.523499
H   -0.416059   3.003265   0.977771
O   -0.689538   2.563214   -2.015207
H   0.591918   -0.739474   -2.775069
C   -4.628068   1.076374   -1.940687
H   -5.016407   0.805362   -2.919714
H   -4.278721   2.112639   -1.991272
H   -5.417035   0.974662   -1.197230

GSG_19.99_t-t_O1-ttggtgtt.xyz
28
0 eng= -740.069218872 zpe= -739.832974
N   0.053729   1.548280   -3.326179
C   -1.082902   1.790762   -2.450346
C   -0.740161   1.106248   -1.135536
N   -1.576569   0.925250   -0.158062
C   -1.240004   -0.044189   0.907220

C	-1.592246	-1.396198	0.277749
N	-0.549094	-2.092517	-0.247380
C	-0.839318	-3.042879	-1.290904
C	-1.255701	-2.331614	-2.572328
O	-1.653964	-3.198129	-3.488239
H	-1.917686	-2.732252	-4.294646
H	-1.225298	2.859589	-2.273342
H	-2.015847	1.367351	-2.825452
O	0.470693	0.672656	-1.045506
H	-0.138463	0.791578	-3.974392
H	0.348042	2.369583	-3.838928
H	-1.884913	0.147966	1.761364
H	-0.194709	0.065009	1.189508
O	-2.749370	-1.724104	0.150086
H	0.362978	-1.660514	-0.238961
H	0.033822	-3.662173	-1.501205
H	-1.652623	-3.702338	-0.985622
O	-1.215506	-1.134234	-2.742931
H	0.853514	0.898209	-1.959056
C	-2.978507	1.362138	-0.234505
H	-3.603702	0.513840	-0.518511
H	-3.081069	2.178593	-0.944684
H	-3.273508	1.718058	0.751336

SSG_0.00_NcA.xyz
31
0 eng= -779.381551461 zpe= -779.114996

C	-0.685092	0.565212	-2.013786
H	-0.998737	1.159949	-2.873283
H	0.303093	0.148732	-2.192788
C	-1.832581	-0.401582	-1.679871
O	-2.862138	0.115798	-1.294028
N	-0.582144	1.474494	-0.837417
H	-1.548432	1.621596	-0.511756
N	-1.651386	-1.729280	-1.858356
C	-0.316400	-2.289589	-1.846281
H	0.232144	-2.052871	-2.763345
H	-0.402532	-3.376936	-1.803354
C	0.484266	-1.802290	-0.630177
O	-0.024550	-1.118142	0.241766
N	1.791716	-2.132080	-0.619812
H	2.188340	-2.654057	-1.386494
C	2.662754	-1.607181	0.403133
H	2.270124	-1.834586	1.398072
H	3.648960	-2.063979	0.325270
C	2.808286	-0.096579	0.313216
O	3.706856	0.330852	1.187300
H	3.791516	1.293594	1.131468
O	2.187029	0.624166	-0.428848
H	-0.086400	0.944735	-0.104601

C	0.103453	2.766586	-1.114454
H	1.101422	2.546740	-1.484998
H	0.164395	3.334466	-0.188433
H	-0.479225	3.314167	-1.852750
C	-2.759353	-2.607837	-1.470395
H	-2.698865	-3.527307	-2.050831
H	-3.697247	-2.102785	-1.684560
H	-2.712416	-2.833153	-0.401294

SSG_7.14_NcA.xyz
31
0 eng= -779.378655006 zpe= -779.112277

N	-1.926667	-1.764245	-2.863580
C	-2.680229	-1.034672	-1.788326
C	-3.847552	-0.295876	-2.457274
N	-3.931245	1.051022	-2.314422
C	-2.791380	1.846936	-1.914400
C	-1.553194	1.551614	-2.768873
N	-0.398948	2.090849	-2.326146
C	0.852405	1.747548	-2.954385
C	1.232810	0.293918	-2.719973
O	2.439909	0.048234	-3.201265
H	2.677613	-0.877908	-3.047811
H	-3.093919	-1.777086	-1.105564
H	-1.959398	-0.418818	-1.256546
O	-4.647910	-0.964645	-3.078055
H	-0.952076	-1.849511	-2.553288
H	-2.547023	1.705228	-0.856686
H	-3.053331	2.899851	-2.035419
O	-1.604192	0.839842	-3.758493
H	-0.388880	2.621739	-1.468184
H	0.791535	1.895347	-4.035934
H	1.649951	2.388111	-2.578709
O	0.534147	-0.533451	-2.185137
H	-1.879680	-1.124441	-3.670659
C	-2.491753	-3.092480	-3.251393
H	-1.890645	-3.496388	-4.063555
H	-3.522055	-2.944147	-3.563138
H	-2.443055	-3.748870	-2.384486
C	-5.013419	1.730096	-3.033270
H	-5.871381	1.065476	-3.083484
H	-4.697607	1.981656	-4.049548
H	-5.284605	2.635239	-2.491505

SSG_8.17_NcB.xyz
31
0 eng= -779.37775258 zpe= -779.111886

C	-2.326398	-0.077328	0.879374
H	-1.444605	-0.536970	1.321985
H	-3.226280	-0.541942	1.290800

C	-2.422394	-0.118014	-0.655579
O	-2.951321	0.834753	-1.202333
N	-2.323443	1.361633	1.251337
H	-2.923333	1.838104	0.561527
N	-1.959783	-1.214791	-1.285884
C	-1.149282	-2.208693	-0.591202
H	-0.912054	-3.004880	-1.298951
H	-1.678958	-2.668807	0.244987
C	0.156854	-1.635143	-0.035179
O	0.599547	-1.998158	1.031227
N	0.765000	-0.678642	-0.788885
H	0.323888	-0.328409	-1.624835
C	1.920779	-0.009793	-0.254825
H	2.649708	-0.734011	0.113573
H	2.404199	0.587870	-1.029525
C	1.559667	0.911674	0.897443
O	2.637707	1.329656	1.529719
H	2.392617	1.925084	2.252977
O	0.433295	1.243482	1.196324
H	-1.356716	1.689514	1.100598
C	-2.729565	1.637535	2.656310
H	-2.055862	1.098673	3.320075
H	-3.754257	1.299970	2.798969
H	-2.657509	2.707702	2.837486
C	-2.167965	-1.362276	-2.728827
H	-2.673064	-2.308420	-2.927417
H	-1.211074	-1.353968	-3.257450
H	-2.782015	-0.537675	-3.080683

SSG_11.61_c-t_N-tcgttgtt.xyz
31
0 eng= -779.3767337 zpe= -779.110573

C	-0.743554	0.604797	-1.831450
H	-0.806320	0.915549	-2.876521
H	0.275647	0.312186	-1.584219
C	-1.841136	-0.443810	-1.557970
O	-2.906543	-0.020997	-1.140580
N	-1.109827	1.762922	-0.965602
H	-2.145904	1.748572	-0.943006
N	-1.588326	-1.729793	-1.857155
C	-0.227673	-2.193132	-2.015639
H	0.276348	-1.673627	-2.836917
H	-0.250310	-3.251068	-2.281679
C	0.582124	-1.990952	-0.726022
O	0.092643	-1.473498	0.257113
N	1.879517	-2.370065	-0.793167
H	2.257941	-2.683827	-1.674339
C	2.790700	-1.936577	0.236110
H	2.416273	-2.221671	1.221721
H	3.764155	-2.408344	0.102904

C	2.963493	-0.424809	0.226958
O	3.840830	-0.052178	1.151012
H	3.956739	0.908168	1.122338
O	2.385482	0.337158	-0.505758
H	-0.790315	2.640532	-1.375583
C	-0.598293	1.623418	0.436807
H	-0.958840	2.467713	1.020395
H	0.488713	1.597896	0.396442
H	-0.974551	0.682084	0.832973
C	-2.606120	-2.723000	-1.501634
H	-2.455985	-3.064449	-0.474008
H	-2.532682	-3.563573	-2.190188
H	-3.588378	-2.266586	-1.587316

SSG_12.62_NtA.xyz
31
0 eng= -779.376628454 zpe= -779.110191

C	-0.892184	1.926147	-0.796506
H	-1.870783	2.086185	-0.347165
H	-0.807271	2.493971	-1.725504
C	-0.570819	0.441209	-1.006206
O	0.569349	0.073442	-0.746714
N	0.137234	2.411795	0.162670
H	1.023925	1.974445	-0.126313
N	-1.557599	-0.366291	-1.415399
C	-1.353233	-1.805609	-1.195563
H	-0.343353	-2.080642	-1.494006
H	-2.084765	-2.360936	-1.778042
C	-1.627039	-1.996328	0.294388
O	-2.758905	-2.028308	0.727337
N	-0.527546	-1.968979	1.101395
H	0.355394	-1.709990	0.682854
C	-0.738588	-1.706197	2.498355
H	-1.495925	-2.378451	2.902699
H	0.184348	-1.863977	3.060438
C	-1.197793	-0.277406	2.745606
O	-1.698621	-0.134296	3.957172
H	-1.975338	0.782396	4.099475
O	-1.096674	0.638118	1.952264
H	-0.093878	1.964925	1.065193
C	0.235601	3.890345	0.287732
H	0.519841	4.306455	-0.676801
H	0.988717	4.130194	1.035181
H	-0.732747	4.281014	0.594268
C	-2.935811	0.088130	-1.609551
H	-3.421185	-0.590184	-2.309188
H	-2.948890	1.082238	-2.054345
H	-3.491125	0.073701	-0.667778

SSG_13.56_c-t_N-gcggtgtt.xyz

31
0 eng= -779.375927994 zpe= -779.10983

C	-3.044422	-1.027460	-0.642889
H	-2.719129	-1.866613	-0.037974
H	-4.107665	-1.103530	-0.872674
C	-2.298209	-0.784797	-1.957690
O	-2.649962	0.175595	-2.612606
N	-2.835830	0.212310	0.161729
H	-3.162006	1.001671	-0.407831
N	-1.286431	-1.617626	-2.308418
C	-0.637127	-2.509199	-1.346119
H	0.119843	-3.081207	-1.882581
H	-1.341646	-3.219419	-0.914552
C	-0.016289	-1.692796	-0.209912
O	-0.601767	-1.519946	0.847865
N	1.164673	-1.096883	-0.483167
H	1.610617	-1.245920	-1.375062
C	1.744243	-0.159727	0.447490
H	1.807885	-0.603887	1.445515
H	2.754934	0.101251	0.136168
C	0.923360	1.111030	0.580739
O	1.587460	2.024112	1.268973
H	1.048200	2.823356	1.362536
O	-0.190069	1.276269	0.142622
H	-1.818094	0.361208	0.284688
C	-3.494031	0.179809	1.495995
H	-3.042756	-0.629992	2.066400
H	-4.560635	0.009890	1.360995
H	-3.322636	1.131919	1.993544
C	-0.584841	-1.314104	-3.557007
H	-0.145091	-2.230853	-3.947299
H	0.192127	-0.558895	-3.401100
H	-1.299286	-0.919324	-4.275395

SSG_16.28_NcC.xyz
31
0 eng= -779.374694238 zpe= -779.108795

N	0.806070	-1.170528	-3.516429
C	-0.439439	-1.103658	-2.699127
C	-0.608141	0.389412	-2.391724
N	-0.825758	0.799887	-1.122896
C	-0.410766	0.006286	0.019652
C	1.110578	-0.201228	0.034205
N	1.705908	-0.156862	1.223982
C	3.125372	-0.396771	1.384337
C	3.462670	-0.215436	2.848741
O	4.753512	-0.425108	3.074448
H	4.934707	-0.299814	4.017772
H	-1.276213	-1.420563	-3.323527
H	-0.319604	-1.771891	-1.852543

```
O       -0.542309       1.130735        -3.354292
H       1.563260        -0.916071       -2.860215
H       -0.886407       -0.977671       0.018016
H       -0.750647       0.510782        0.923782
O       1.719326        -0.432759       -1.010310
H       1.196619        0.073148        2.071020
H       3.395988        -1.408565       1.071784
H       3.713117        0.301691        0.783396
O       2.651859        0.078305        3.682041
H       0.733816        -0.408340       -4.204428
C       1.064927        -2.480814       -4.171135
H       1.992677        -2.413506       -4.735186
H       0.235234        -2.709624       -4.837024
H       1.151134        -3.243725       -3.399953
C       -0.921227       2.248440        -0.909969
H       -1.510346       2.433208        -0.013074
H       -1.411175       2.699352        -1.768389
H       0.073742        2.690327        -0.800292
```

```
SSG_18.21_c-t_N-gcggtgtt.xyz
31
0 eng= -779.374409867 zpe= -779.108059
C       -1.876481       -1.580036       -2.950724
H       -2.709940       -1.642921       -3.651598
H       -1.607765       -2.565948       -2.585982
C       -2.208951       -0.508886       -1.902804
O       -2.326398       0.621046        -2.337794
N       -0.695860       -1.027706       -3.676327
H       0.071417        -1.049611       -2.983630
N       -2.351215       -0.851647       -0.602754
C       -1.721205       -2.042607       -0.057035
H       -2.003038       -2.939203       -0.610860
H       -2.080770       -2.176226       0.963177
C       -0.197773       -1.883085       -0.110357
O       0.349559        -1.555122       -1.159258
N       0.487143        -2.115799       1.018425
H       -0.027359       -2.235067       1.878439
C       1.875447        -1.721716       1.101857
H       2.325191        -2.122868       2.009982
H       2.430338        -2.111309       0.247829
C       1.988658        -0.201271       1.108916
O       3.265958        0.171537        1.135989
H       3.312587        1.138372        1.161361
O       1.053373        0.546615        1.096481
H       -0.912236       -0.038234       -3.858776
C       -0.328779       -1.750253       -4.923595
H       -0.109193       -2.786222       -4.672712
H       0.549105        -1.277485       -5.358575
H       -1.166094       -1.699704       -5.616874
C       -2.545813       0.247344        0.353444
```

H	-3.091048	1.048271	-0.137315
H	-1.577353	0.626274	0.696422
H	-3.124491	-0.124562	1.198393

SSG_18.98_NcD.xyz
31
0 eng= -779.374238318 zpe= -779.107767

C	0.008891	-0.980327	-3.003531
H	-0.233541	-1.588760	-3.876476
H	0.604243	-1.558400	-2.301850
C	-1.300712	-0.352587	-2.501145
O	-1.821024	0.454145	-3.245695
N	0.845748	0.165247	-3.460836
H	1.224245	0.607331	-2.611027
N	-1.807995	-0.742998	-1.308740
C	-0.938563	-1.300128	-0.294604
H	-0.608629	-2.311490	-0.551950
H	-1.504460	-1.382388	0.635208
C	0.288832	-0.409828	-0.060697
O	0.426392	0.659215	-0.629031
N	1.231380	-0.907950	0.771048
H	1.134511	-1.841611	1.140796
C	2.505041	-0.241240	0.864834
H	2.366039	0.787759	1.210733
H	3.135538	-0.761715	1.588166
C	3.215772	-0.195536	-0.490496
O	4.360819	0.477747	-0.522647
H	4.605416	0.847065	0.334827
O	2.780035	-0.707567	-1.485442
H	0.193388	0.836253	-3.891043
C	1.946496	-0.204576	-4.394918
H	2.585243	-0.927870	-3.894629
H	2.515160	0.691634	-4.633677
H	1.504277	-0.622483	-5.297275
C	-3.026476	-0.064858	-0.855565
H	-3.561198	-0.722413	-0.171695
H	-3.650509	0.147384	-1.719336
H	-2.778083	0.876319	-0.356864

SSG_19.33_c-c_N-gcgtcgtt.xyz
31
0 eng= -779.374017472 zpe= -779.107634

C	0.103553	-3.540695	-3.750748
H	0.750843	-4.017982	-4.488003
H	0.347988	-2.483054	-3.668227
C	0.127873	-4.386815	-2.469920
O	-0.324317	-5.511146	-2.571234
N	-1.305128	-3.655109	-4.233095
H	-1.883122	-3.190935	-3.513656
N	0.628183	-3.853312	-1.334174

C	0.755890	-2.419782	-1.180538
H	1.510233	-2.013837	-1.858727
H	1.100168	-2.219072	-0.164250
C	-0.582556	-1.696101	-1.410241
O	-1.567541	-2.301736	-1.804173
N	-0.615613	-0.371594	-1.139267
H	-1.506260	0.061336	-1.351571
C	0.547026	0.484223	-1.137662
H	0.265685	1.483223	-0.805638
H	1.309592	0.132897	-0.436758
C	1.181324	0.573096	-2.522572
O	2.068357	1.554451	-2.580357
H	2.475318	1.577345	-3.459365
O	0.922979	-0.175555	-3.429181
H	-1.539723	-4.656248	-4.195125
C	-1.549073	-3.073321	-5.580433
H	-0.923724	-3.593572	-6.303300
H	-1.288872	-2.016823	-5.549608
H	-2.599990	-3.196988	-5.832876
C	0.515138	-4.654148	-0.111875
H	1.340100	-4.404492	0.554083
H	0.567265	-5.706255	-0.377570
H	-0.439758	-4.459188	0.384664
